# Tuberculosis screening among ambulatory people living with HIV: a systematic review and individual participant data meta-analysis

**DOI:** 10.1016/S1473-3099(21)00387-X

**Published:** 2022-04

**Authors:** Ashar Dhana, Yohhei Hamada, Andre P Kengne, Andrew D Kerkhoff, Molebogeng X Rangaka, Tamara Kredo, Annabel Baddeley, Cecily Miller, Satvinder Singh, Yasmeen Hanifa, Alison D Grant, Katherine Fielding, Dissou Affolabi, Corinne S Merle, Ablo Prudence Wachinou, Christina Yoon, Adithya Cattamanchi, Christopher J Hoffmann, Neil Martinson, Eyongetah Tabenyang Mbu, Melissa S Sander, Taye T Balcha, Sten Skogmar, Byron W P Reeve, Grant Theron, Gcobisa Ndlangalavu, Surbhi Modi, Joseph Cavanaugh, Susan Swindells, Richard E Chaisson, Faiz Ahmad Khan, Andrea A Howard, Robin Wood, Swe Swe Thit, Mar Mar Kyi, Josh Hanson, Paul K Drain, Adrienne E Shapiro, Tendesayi Kufa, Gavin Churchyard, Duc T Nguyen, Edward A Graviss, Stephanie Bjerrum, Isik S Johansen, Jill K Gersh, David J Horne, Sylvia M LaCourse, Haider Abdulrazzaq Abed Al-Darraji, Adeeba Kamarulzaman, Russell R Kempker, Nestani Tukvadze, David A Barr, Graeme Meintjes, Gary Maartens

**Affiliations:** a Department of Medicine, University of Cape Town, Cape Town, South Africa; b Wellcome Centre for Infectious Diseases Research in Africa, Institute of Infectious Disease and Molecular Medicine, University of Cape Town, Cape Town, South Africa; c Faculty of Health Sciences, University of Cape Town, Cape Town, South Africa; d Centre for International Cooperation and Global Tuberculosis Information, The Research Institute of Tuberculosis, Japan Anti-Tuberculosis Association, Tokyo, Japan; e Institute for Global Health, University College London, London, UK; f Non-communicable Diseases Research Unit, South African Medical Research Council, Cape Town, South Africa; g Cochrane South Africa, South African Medical Research Council, Cape Town, South Africa; h Division of HIV, Infectious Diseases and Global Medicine, Zuckerberg San Francisco General Hospital and Trauma Center, University of California, San Francisco, CA, USA; i Department of Medicine, Division of Pulmonary and Critical Care Medicine, Center for Tuberculosis, University of California, San Francisco, CA, USA; j Division of Clinical Pharmacology, Department of Medicine, Faculty of Medicine and Health Sciences, Stellenbosch University, Stellenbosch, South Africa; k Global Tuberculosis Programme, World Health Organization, Geneva, Switzerland; l Global HIV, Hepatitis and STIs Programme, World Health Organization, Geneva, Switzerland; m TB Centre, London School of Hygiene & Tropical Medicine, London, UK; n Africa Health Research Institute, School of Laboratory Medicine and Medical Sciences, College of Health Sciences, University of KwaZulu-Natal, Durban, South Africa; o School of Public Health, University of the Witwatersrand, Johannesburg, South Africa; p Perinatal HIV Research Unit, University of the Witwatersrand, Johannesburg, South Africa; q Laboratoire de Référence des Mycobactéries, Cotonou, Benin; r UNICEF/UNDP/World Bank/WHO Special Programme for Research and Training in Tropical Diseases, Geneva, Switzerland; s National Hospital for Tuberculosis and Pulmonary Diseases, Cotonou, Benin; t Johns Hopkins University School of Medicine, Baltimore, MD, USA; u Johns Hopkins University Center for Tuberculosis Research, Baltimore, MD, USA; v Bamenda Regional Hospital HIV Treatment Center, Bamenda, Cameroon; w Tuberculosis Reference Laboratory Bamenda, Bamenda, Cameroon; x Clinical Infection Medicine, Lund University, Malmö, Sweden; y Department of Translational Medicine, Lund University, Malmö, Sweden; z DSI-NRF Centre of Excellence for Biomedical Tuberculosis Research, South African Medical Research Council Centre for Tuberculosis Research, Division of Molecular Biology and Human Genetics, Faculty of Medicine and Health Sciences, Stellenbosch University, Cape Town, Western Cape, South Africa; aa US Centers for Disease Control and Prevention, Atlanta, GA, USA; ab University of Nebraska Medical Center, Omaha, NE, USA; ac McGill International Tuberculosis Centre, Research Institute of the McGill University Health Centre, Montreal, QC, Canada; ad ICAP at Columbia University, New York, NY, USA; ae Department of Epidemiology, Mailman School of Public Health, Columbia University, New York, NY, USA; af Department of Medicine, University of Medicine 2, Yangon, Yangon Division, Myanmar; ag The Kirby Institute, University of New South Wales, Sydney, NSW, Australia; ah Department of Global Health, University of Washington, Seattle, WA, USA; ai Department of Medicine, University of Washington, Seattle, WA, USA; aj Department of Epidemiology, University of Washington, Seattle, WA, USA; ak Centre for HIV and STIs, National Institute for Communicable Diseases, Johannesburg, South Africa; al The Aurum Institute, Parktown, South Africa; am Department of Pathology and Genomic Medicine, Houston Methodist Research Institute, Houston, TX, USA; an Department of Clinical Research, Infectious Diseases, University of Southern Denmark, Odense, Denmark; ao Research Unit for Infectious Diseases, Odense University Hospital, University of Southern Denmark, Odense, Denmark; ap Department of Medicine, Division of Infectious Diseases, University of Washington, Seattle, WA, USA; aq Department of Global Health, Division of Infectious Diseases, University of Washington, Seattle, WA, USA; ar Centre of Excellence for Research in AIDS, University of Malaya, Kuala Lumpur, Malaysia; as Department of Medicine, Division of Infectious Diseases, Emory University School of Medicine, Atlanta, GA, USA; at National Center for Tuberculosis and Lung Diseases, Tbilisi, Georgia; au Institute of Infection and Global Health, University of Liverpool, Liverpool, UK; au University of Washington, Seattle, WA, USA

## Abstract

**Background:**

The WHO-recommended tuberculosis screening and diagnostic algorithm in ambulatory people living with HIV is a four-symptom screen (known as the WHO-recommended four symptom screen [W4SS]) followed by a WHO-recommended molecular rapid diagnostic test (eg Xpert MTB/RIF [hereafter referred to as Xpert]) if W4SS is positive. To inform updated WHO guidelines, we aimed to assess the diagnostic accuracy of alternative screening tests and strategies for tuberculosis in this population.

**Methods:**

In this systematic review and individual participant data meta-analysis, we updated a search of PubMed (MEDLINE), Embase, the Cochrane Library, and conference abstracts for publications from Jan 1, 2011, to March 12, 2018, done in a previous systematic review to include the period up to Aug 2, 2019. We screened the reference lists of identified pieces and contacted experts in the field. We included prospective cross-sectional, observational studies and randomised trials among adult and adolescent (age ≥10 years) ambulatory people living with HIV, irrespective of signs and symptoms of tuberculosis. We extracted study-level data using a standardised data extraction form, and we requested individual participant data from study authors. We aimed to compare the W4SS with alternative screening tests and strategies and the WHO-recommended algorithm (ie, W4SS followed by Xpert) with Xpert for all in terms of diagnostic accuracy (sensitivity and specificity), overall and in key subgroups (eg, by antiretroviral therapy [ART] status). The reference standard was culture. This study is registered with PROSPERO, CRD42020155895.

**Findings:**

We identified 25 studies, and obtained data from 22 studies (including 15 666 participants; 4347 [27·7%] of 15 663 participants with data were on ART). W4SS sensitivity was 82% (95% CI 72–89) and specificity was 42% (29–57). C-reactive protein (≥10 mg/L) had similar sensitivity to (77% [61–88]), but higher specificity (74% [61–83]; n=3571) than, W4SS. Cough (lasting ≥2 weeks), haemoglobin (<10 g/dL), body-mass index (<18·5 kg/m^2^), and lymphadenopathy had high specificities (80–90%) but low sensitivities (29–43%). The WHO-recommended algorithm had a sensitivity of 58% (50–66) and a specificity of 99% (98–100); Xpert for all had a sensitivity of 68% (57–76) and a specificity of 99% (98–99). In the one study that assessed both, the sensitivity of sputum Xpert Ultra was higher than sputum Xpert (73% [62–81] *vs* 57% [47–67]) and specificities were similar (98% [96–98] *vs* 99% [98–100]). Among outpatients on ART (4309 [99·1%] of 4347 people on ART), W4SS sensitivity was 53% (35–71) and specificity was 71% (51–85). In this population, a parallel strategy (two tests done at the same time) of W4SS with any chest x-ray abnormality had higher sensitivity (89% [70–97]) and lower specificity (33% [17–54]; n=2670) than W4SS alone; at a tuberculosis prevalence of 5%, this strategy would require 379 more rapid diagnostic tests per 1000 people living with HIV than W4SS but detect 18 more tuberculosis cases. Among outpatients not on ART (11 160 [71·8%] of 15 541 outpatients), W4SS sensitivity was 85% (76–91) and specificity was 37% (25–51). C-reactive protein (≥10 mg/L) alone had a similar sensitivity to (83% [79–86]), but higher specificity (67% [60–73]; n=3187) than, W4SS and a sequential strategy (both test positive) of W4SS then C-reactive protein (≥5 mg/L) had a similar sensitivity to (84% [75–90]), but higher specificity than (64% [57–71]; n=3187), W4SS alone; at 10% tuberculosis prevalence, these strategies would require 272 and 244 fewer rapid diagnostic tests per 1000 people living with HIV than W4SS but miss two and one more tuberculosis cases, respectively.

**Interpretation:**

C-reactive protein reduces the need for further rapid diagnostic tests without compromising sensitivity and has been included in the updated WHO tuberculosis screening guidelines. However, C-reactive protein data were scarce for outpatients on ART, necessitating future research regarding the utility of C-reactive protein in this group. Chest x-ray can be useful in outpatients on ART when combined with W4SS. The WHO-recommended algorithm has suboptimal sensitivity; Xpert for all offers slight sensitivity gains and would have major resource implications.

**Funding:**

World Health Organization.

## Introduction

Tuberculosis is the leading cause of death among people living with HIV and often goes undiagnosed.^1^, ^2^ One approach to reduce this tuberculosis burden involves systematic screening as part of an intensified case-finding strategy. WHO recommends a tuberculosis screening and diagnostic algorithm in people living with HIV at each clinical encounter using the WHO-recommended four-symptom screen (W4SS; comprising any one of current cough, fever, night sweats, or weight loss) followed by confirmatory testing using a WHO-recommended molecular rapid diagnostic test such as Xpert MTB/RIF (referred to hereon as Xpert) or Xpert MTB/RIF Ultra (referred to hereon as Xpert Ultra) for those with a positive W4SS.^3^, ^4^ However, the W4SS has low specificity, meaning many people require unnecessary and expensive confirmatory testing with a rapid diagnostic test.^3^, ^5^ Furthermore, the W4SS has reduced sensitivity in specific subgroups (eg, those who are on antiretroviral therapy [ART], are pregnant, or have high CD4 counts).^3^, ^5^, ^6^ The entire algorithm might also have low sensitivity,^7^ because overall sensitivity depends on the combined sensitivity of the W4SS and the rapid diagnostic test.

**Research in context**

**Evidence before this study**

Tuberculosis is common and often goes undiagnosed in people living with HIV. The WHO-recommended four-symptom screen (W4SS; comprising any one of current cough, fever, night sweats, or weight loss) was developed after a 2011 individual participant data meta-analysis to rule out active tuberculosis before initiating tuberculosis preventive therapy. WHO recommends that ambulatory people living with HIV be screened for tuberculosis at each clinical encounter with the W4SS followed by a WHO-recommended molecular rapid diagnostic test (eg, Xpert MTB/RIF [Xpert] or Xpert MTB/RIF Ultra [Xpert Ultra]) for those with a positive W4SS. In 2018, WHO commissioned an updated systematic review and meta-analysis because the earlier meta-analysis primarily comprised people living with HIV not on antiretroviral therapy (ART). The updated review showed that the W4SS had a specificity of only 27% in people living with HIV not on ART. Thus, large numbers of people ultimately undergo unnecessary and expensive WHO-recommended rapid diagnostic testing. The updated review also found that the W4SS had a sensitivity of only 51% in people living with HIV on ART (*vs* 89% for those not on ART). Furthermore, the entire WHO-recommended algorithm (W4SS then Xpert) might be suboptimal because its sensitivity depends on both the W4SS and Xpert. Alternative screening tests to the W4SS need to be explored. Several recent studies have shown that C-reactive protein might have improved diagnostic accuracy compared with W4SS. The 2018 WHO-commissioned systematic review assessed the addition of chest x-ray to W4SS, but identified few studies, particularly among those on ART. Other screening tests (eg, haemoglobin and body-mass index [BMI]) are also known to be associated with tuberculosis, but their diagnostic accuracy is unclear. Finally, some experts have argued for an Xpert for all strategy (as opposed to the WHO-recommended algorithm) to improve sensitivity.

**Added value of this study**

To inform an update to WHO guidelines on tuberculosis screening among ambulatory people living with HIV regardless of signs and symptoms of tuberculosis, we did an individual participant data meta-analysis of 22 studies and did pre-specified subgroup analyses, notably by ART status. We found that the W4SS had a specificity of 37% in people not on ART, and a sensitivity of 53% in people on ART (*vs* 85% in people not on ART). We found that C-reactive protein (≥10 mg/L cutoff) had comparable sensitivity to W4SS but higher specificity, leading to fewer rapid diagnostic tests being needed. Chest x-ray had lower sensitivity than W4SS in studies that directly compared both tests, making it unsuitable as a standalone screening test. Cough (lasting ≥2 weeks), haemoglobin (<10 g/dL), body-mass index (<18·5 kg/m^2^), and lymphadenopathy had high specificities, but their low sensitivities also made them unsuitable as screening tests. Xpert for all slightly improved sensitivity compared with the WHO-recommended algorithm (W4SS followed by Xpert). In one study, Xpert Ultra improved sensitivity over Xpert (73% *vs* 57%). ART status had a major effect on the diagnostic accuracy of W4SS and C-reactive protein, both of which had lower sensitivities but higher specificities among outpatients on ART. Among outpatients on ART, the best performing screening strategy to improve sensitivity was a parallel strategy (two screening tests offered at the same time) of W4SS with any chest x-ray abnormality. Among outpatients not on ART, the best performing screening strategy to improve specificity was C-reactive protein (≥10 mg/L) as a standalone test or a sequential strategy (second screening test offered only if first screening test is positive) of W4SS then C-reactive protein (≥5 mg/L).

**Implications of all the available evidence**

Compared with W4SS, C-reactive protein reduces the need for further rapid diagnostic tests without compromising sensitivity, and it has been included in the updated WHO tuberculosis screening guidelines. However, data on use of C-reactive protein in outpatients on ART were scarce. In outpatients on ART, chest x-ray could be used in parallel with W4SS, depending on available resources, because this strategy detects more tuberculosis cases than W4SS alone. The current WHO-recommended algorithm (W4SS followed by Xpert) is insufficiently sensitive to identify all tuberculosis cases. Xpert for all would offer slight gains over this strategy in terms of sensitivity, but would be resource intensive. Future research is needed to assess the utility of C-reactive protein in outpatients on ART and Xpert Ultra in all people living with HIV.

Alternative screening tests to the W4SS need to be explored. According to WHO, a screening test should have a sensitivity of more than 90% and a specificity of more than 70%.^8^ Several studies have shown that C-reactive protein has improved diagnostic accuracy compared with W4SS.^9^, ^10^, ^11^ C-reactive protein assays as point-of-care assays are easy to use, inexpensive (approximately US$2 per test), and provide rapid results (<3 min). One study among people living with HIV initiating ART found that replacing the W4SS with C-reactive protein (10 mg/L) could halve the number of Xpert tests performed.^7^ Chest x-ray might also be useful for tuberculosis screening, especially when combined with the W4SS in people living with HIV on ART;^5^ however, it is often unavailable and resource intensive. Haemoglobin, body-mass index (BMI), and lymphadenopathy are other predictors of tuberculosis,^12^, ^13^ but their diagnostic accuracy is unclear. The authors of some studies among people living with HIV initiating ART have argued that Xpert for all, rather than Xpert only for those who are positive on the W4SS, should be the preferred strategy.^14^, ^15^ This approach could optimise diagnostic yield, but cost and capacity issues could restrict its implementation in resource-poor settings. The Alere Determine TB-LAM (AlereLAM) Ag lateral flow urine assay for screening outpatients living with HIV has been recently reviewed and has a sensitivity of 31% and specificity of 95%;^16^ next-generation assays based on detection of lipoarabinomannan (eg, Fujifilm SILVAMP TB-LAM) have higher sensitivity (eg, 71%).^17^ WHO recommends the AlereLAM assay if an outpatient has a positive W4SS, CD4 count of 100 cells per μL or lower, is WHO clinical stage 3 or 4, or has a WHO-defined danger sign.^18^

We did a systematic review and individual participant data meta-analysis to provide a more detailed and precise analysis of the accuracy of different tuberculosis screening tests and strategies compared with W4SS among ambulatory people living with HIV, including key subgroups. We also assessed the accuracy of the WHO-recommended screening and diagnostic algorithm (W4SS followed by Xpert) and compared its accuracy with Xpert for all as the first screening test.

## Methods

### Search strategy and selection criteria

In this systematic review and individual participant data meta-analysis, we updated the systematic review done by Hamada and colleagues,^5^ who searched PubMed (MEDLINE), Embase, the Cochrane Library, and conference abstracts (from the Conference on Retroviruses and Opportunistic Infections, AIDS/International AIDS Society, and International Union Against TB and Lung Diseases conferences) without language or geographical restrictions from Jan 1, 2011, to March 12, 2018. The start date restrictions correspond to the year WHO issued recommendations on the W4SS. We rescreened all potential full texts identified via Hamada and colleagues' search to identify eligible studies. Additionally, we applied the same search strategy to the same databases for publications between March 12, 2018, and Aug 2, 2019. We also screened reference lists of reviews and included articles and contacted field experts. Detailed search terms are in the appendix (p 2).

Two authors (AD and YHam) independently screened titles and abstracts from the search and subsequently screened the full texts of potentially eligible articles. For abstracts that were not in English, we used Google Translate to translate the abstracts before screening. We included prospective cross-sectional studies, prospective observational studies, and randomised trials that collected at least one sputum sample for tuberculosis culture from adult and adolescent (ie, aged ≥10 years) ambulatory people living with HIV regardless of signs and symptoms of tuberculosis. We excluded case-control studies, general community or household contact-screening studies, and studies that involved people living with HIV who were already on tuberculosis treatment or had a current tuberculosis diagnosis.

The target condition was active tuberculosis (ie, we exlcuded articles on latent tuberculosis infections). The reference standard for confirmed tuberculosis was bacteriological confirmation of *Mycobacterium tuberculosis* using culture of a sputum sample or other samples, or both.

We included primary datasets that had sufficient data to allow us to compare the W4SS with alternative screening tests or strategies and the WHO-recommended algorithm (W4SS followed by Xpert) with Xpert for all. We examined several systematically performed screening tests: C-reactive protein, chest x-ray, Xpert or Xpert Ultra, haemoglobin, BMI, lymphadenopathy (on examination), and cough (lasting ≥2 weeks). A positive chest x-ray was defined by the authors of the included studies and categorised as any abnormality or abnormality suggestive of tuberculosis. We were primarily interested in any abnormality on chest x-ray because identification of features suggestive of tuberculosis on chest x-ray requires a skilled reader. For C-reactive protein, we primarily focused on the 10 mg/L threshold, which is considered the upper limit of normal.^19^, ^20^ We also explored a 5 mg/L threshold to maximise sensitivity and an 8 mg/L threshold because a previous study found that this cutoff met WHO's minimum sensitivity (≥90%) and specificity (≥70%) targets.^8^, ^11^ Finally, we examined several parallel strategies (two screening tests offered at the same time) to improve sensitivity and sequential strategies (second screening test offered only if first screening test is positive) to improve specificity.

We have reported our findings according to the PRISMA-IPD and PRISMA-DTA statements.^21^, ^22^ This study was registered with PROSPERO (CRD42020155895).

### Data extraction, study quality, and individual participant data synthesis

Using a standardised data extraction form, two authors (AD and YHam) independently extracted study-level information on first author, publication year, study period, country, setting (eg, HIV clinic, hospital clinic, prison clinic), exclusion criteria, study design, type of participants (eg, all people living with HIV, only pregnant people), and method of tuberculosis diagnosis. Two authors independently (AD and YHam) assessed study quality using the Quality Assessment of Diagnostic Accuracy Studies-2 (QUADAS-2) tool.^23^

We invited authors of eligible datasets by email to contribute individual participant data. We prespecified variables to be collected after consultation with WHO and our study group (appendix p 3). We standardised individual participant data, then synthesised a single dataset with study-level data. Study participants younger than 10 years were excluded, and contaminated cultures were considered negative. To ensure integrity of the individual participant data, we checked information against study publications and did checks on each dataset for missing, duplicate, invalid, and implausible items.^24^, ^25^ We resolved discrepancies by contacting the corresponding author.

### Statistical analysis

We did analyses overall and in key subgroups, comprising outpatient clinic attendees (on ART *vs* not on ART), CD4 count (≤200 *vs* >200 cells per μL), and pregnancy. To analyse individual participant data we used a two-stage approach. Individual participant data were first analysed separately in each study using an appropriate statistical method (accounting for the design of data collection) and reduced to aggregate data, which were then synthesised using meta-analytical techniques.

In the first stage, we estimated tuberculosis prevalence, positivity rate (proportion of screen-positive participants), and measures of diagnostic performance (including sensitivity and specificity) by screening test or strategy. In the second stage, we pooled tuberculosis prevalence and positivity rates using a generalised linear mixed model with logit transformation^26^ in preference to the protocol specified DerSimonian and Laird random effects model for proportions with variance stabilisation by applying the Freeman-Tukey double arcsine transformation. We assessed heterogeneity using Cochran's Q test and the *I*^2^ statistic.^27^ We pooled absolute accuracy measures (sensitivity, specificity) in a bivariate generalised linear mixed model.^28^ In the case of non-convergence, we assumed no correlation between measures of sensitivity and specificity to simplify the model.^29^ When data were sparse, we did not do a meta-analysis (eg, for C-reactive protein [n=62] and lymphadenopathy [n=34] in pregnant participants). We illustrated the absolute pooled sensitivity and specificity using summary receiver-operating characteristic (ROC) curves.^30^ To compare the accuracy of screening tests and strategies, we did both indirect and direct comparisons. Direct comparisons were based on studies that assessed both tests of interest; indirect comparisons were based on all studies that assessed at least one test of interest. We did a bivariate meta-regression with test type as a covariate and used likelihood ratio tests to assess the significance of differences in sensitivity and specificity. We explored study-level characteristics (tuberculosis prevalence and reference standard) as potential sources of heterogeneity. Accounting for the variation of tuberculosis prevalence across studies and their pooled values, we applied pooled accuracy estimates to a hypothetical cohort of 1000 individuals to show the consequences of using each screening test and strategy, which included calculating negative and positive predictive values using Bayes' theorem. We also calculated predictive values using a trivariate generalised linear mixed model that jointly models predictive values and test prevalence.^31^

We did several sensitivity analyses. We assessed diagnostic accuracy using a prespecified second reference standard of culture or Xpert. This analysis included one additional study of outpatients living with HIV (not on ART and on ART) that did not meet our primary reference standard criterion.^13^ We also assessed diagnostic accuracy using a reference standard of Xpert alone because it is one of the molecular rapid diagnostic tests recommended by WHO. Finally, we did a direct comparison of the accuracy of W4SS followed by Xpert with the accuracy of C-reactive protein (≥10 mg/L) followed by Xpert.

We assessed publication bias with funnel plots (for analyses with ten or more studies) and applied Egger's test. Although Deeks' test might be more appropriate, most methods to test for publication bias in studies of test accuracy have limitations.^32^ Therefore, we also applied the trim-and-fill method to provide bias-adjusted estimates.^33^

We selected a p value threshold of 0·05 to characterise statistically significant findings. We did all meta-analyses using *lme, altmeta, meta, metafor,* and *mada* packages in R (version 3.6.1). The substantive protocol deviations were that we did not perform a leave-one-out sensitivity analysis and did not compare individual participant data results with aggregate data for which individual participant data were not obtained because we obtained more than 90% of requested data.

### Role of the funding source

The funder had a role in the study design, data collection, data analysis, data interpretation, and writing of the report.

## Results

**Figure 1:**
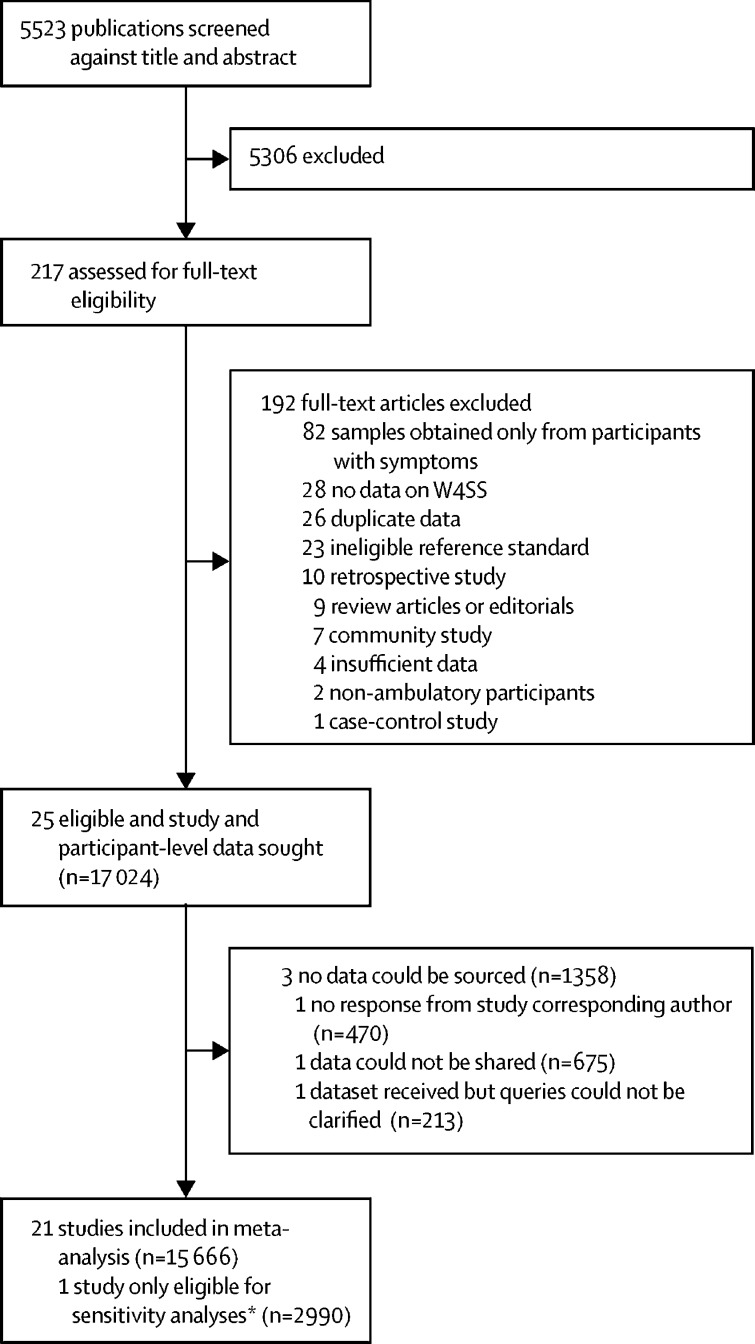
Study selection W4SS=WHO-recommended four symptom screen. *One study (Hanifa and colleagues^13^) was incorporated into sensitivity analyses because the study's reference standard made it ineligible for the main analyses.

Of 5523 potentially eligible publications, 25 were eligible (figure 1). Individual participant data were provided for 22 studies (including one study^13^ that was eligible only for sensitivity analyses).^6^, ^10^, ^11^, ^12^, ^34^, ^35^, ^36^, ^37^, ^38^, ^39^, ^40^, ^41^, ^42^, ^43^, ^44^, ^45^, ^46^, ^47^, ^48^, ^49^, ^50^ Individual participant data were not provided for three studies.^51^, ^52^, ^53^ Hence, we obtained individual participant data for 15 666 (92%) of 17 024 participants identified. The characteristics of included studies are shown in the appendix (pp 4–5). The studies collected data from 2007 to 2020. 18 studies were done in sub-Saharan Africa. Two studies included only pregnant women, and one study included only people living in prison. Overall, we judged studies as low risk of bias in most QUADAS-2 domains (appendix pp 54–55), but six studies had high applicability concerns for participant selection (eg, selected only people living with HIV with advanced immunosuppression). Missing data by study are shown in the appendix (p 6).

Participant characteristics overall are shown in table 1 and by study are shown in the appendix (pp 7–10). 10 388 (66·3%) of 15 666 participants were female, and 4347 (27·8%) of 15 663 with available data were on ART. W4SS was positive in 8028 (51·3%) of 15 625 participants, and C-reactive protein was elevated (≥10 mg/L) in 1259 (35·1%) of 3582 participants. C-reactive protein was measured with a point-of-care assay (2695 participants) or laboratory assay (887 participants) in five studies. The median CD4 count was 269 cells per μL (IQR 142–439; in 15 281 participants).

**Table 1:** Summary of main characteristics for all participants

		**All participants (n=15 666)**
Clinical setting		
	Outpatient	15 541 (99·2%)
	Other setting*	125 (0·8%)
Age, years		34 (28–42)
Sex		
	Female	10 388 (66·3%)
	Male	5278 (33·7%)
ART status		
	On ART	4347/15 663 (27·8%)
	Outpatients on ART	4328/15 538 (27·9%)
	Not on ART	11 316/15 663 (72·2%)
	Outpatients not on ART	11 210/15 538 (72·1%)
CD4 count, cells per μL		
	n	15 281
	Median	269 (142–439)
History of tuberculosis		1955/11 148 (17·5%)
W4SS		8028/15 652 (51·3%)
	Cough	4629/15 623 (29·6%)
	Fever	3391/15 631 (21·7%)
	Weight loss	5575/15 602 (35·7%)
	Night sweats	3270/15 630 (20·9%)
Cough lasting ≥2 weeks		2205/10 919 (20·2%)
Lymphadenopathy		374/2394 (15·6%)
Chest x-ray		
	Suggestive of tuberculosis	1296/6177 (21·0%)
	Any abnormality	2158/6222 (34·7%)
Xpert positive†		616/8625 (7·1%)
Body-mass index, kg/m^2^		
	n	12 704
	Median	22 (19–26)
C-reactive protein, mg/L‡		
	n	3582
	Median	4 (2–21)
	≥10 mg/L	1259 (35·1%)
Haemoglobin, g/dL		
	n	5118
	Median	12 (10–13)
	<10 g/dL	1093 (21·4%)

Data are median (IQR), n (%), or n/N (%). If data were not available for the full cohort, the revised denominator or count is provided. ART=antiretroviral therapy. W4SS=WHO four-symptom screening.

* One study was among people living in prison.

† Sputum or non-sputum sample Xpert result, or both.

‡ Measured with a point-of-care assay (n=2695) or laboratory assay (n=887).

The pooled tuberculosis prevalence was 7·7% (95% CI 5·7–10·4) using culture as a reference standard (table 2). The pooled prevalence of tuberculosis in outpatients not on ART was 9·3% (7·0–12·1) compared with 3·3% (2·2–4·8) among outpatients on ART. For participants with a CD4 count of 200 cells per μL or less, the prevalence of tuberculosis was 13·7% (11·1–16·7) and among those with a CD4 count of more than 200 cells per μL it was 4·9% (3·6–6·6; table 2). Heterogeneity of tuberculosis prevalence was high. The pooled tuberculosis prevalences were slightly higher using a reference standard of either culture or Xpert than with a reference standard of culture alone, but subgroup comparisons remained qualitatively similar (appendix p 11).

**Table 2:** Prevalence of tuberculosis in all participants and by subgroup (using culture as a reference standard)

		**Number of studies**	**Number of participants**	**Number of tuberculosis cases**	Prevalence (95% CI)*	**Heterogeneity**		**p value for publication bias**†	**p value for between-subgroup heterogeneity**‡
						*I^2^* (95% CI)	p value		
All		21	15 611	1347	7·7% (5·7–10·4)	95 (94–96)	<0·0001	0·024	..
Setting and ART status		21	15 608	1347	7·7% (5·7–10·4)	95 (94–96)	<0·0001	0·025	..
	Outpatients (on ART)§	9	4309	137	3·3% (2·2–4·8)	81 (65–90)	<0·0001	0·79	<0·0001
	Outpatients (not on ART)	20	11 174	1195	9·3% (7·0–12·1)	92 (89–94)	<0·0001	0·050	..
	Other setting¶	1	125	15	12·0% (7·4–19·0)	..	..	..	..
CD4 count		21	15 227	1320	7·8% (5·8–10·4)	95 (94–96)	<0·0001	0·024	..
	≤200 cells per μL	21	5622	866	13·7% (11·1–16·7)	84 (77–89)	<0·0001	0·035	<0·0001
	>200 cells per μL	21	9605	454	4·9% (3·6–6·6)	88 (84–92)	<0·0001	0·22	..
Pregnancy status‖		21	10 351	701	6·4% (4·7–8·7)	91 (88–94)	<0·0001	0·15	..
	Pregnant	8	1938	53	2·7% (2·1–3·6)	0 (0–60)	<0·0001	0·038	<0·0001
	Not pregnant	19	8413	648	7·3% (5·4–9·8)	90 (85–93)	<0·0001	0·21	..

ART=antiretroviral therapy.

* Calculated using meta-analysis of proportions.

† Egger's test.

‡ Cochran's Q test (based on random effects model).

§ p value for between-subgroup heterogeneity compares outpatients (on ART) with outpatients (not on ART).

¶ One study was among a prison population.

‖ Pregnancy status was unavailable for some studies, and so female participants in those studies were categorised as not pregnant.

**Figure 2:**
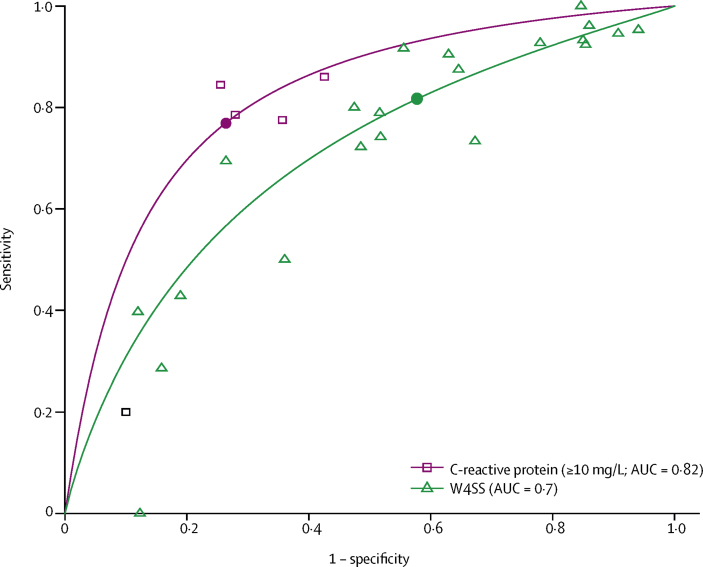
Summary ROC curves comparing C-reactive protein (≥10 mg/L) with W4SS in all participants* AUC=area under the ROC. ROC=receiver operating characteristic. W4SS=WHO-recommended four-symptom screen. *Data were extrapolated beyond observed datapoints.

Plots of sensitivity and specificity for each test in all participants and each subgroup are shown in the appendix (pp 56–61). Indirect comparisons between each test and W4SS in all participants are shown in table 3 and each subgroup are shown in the appendix (pp 12–16). Among 15 597 participants with available culture results, the sensitivity of W4SS was 82% (95% CI 72–89) and specificity was 42% (29–57; table 3; appendix p 56). The sensitivity of C-reactive protein (≥10 mg/L) was similar to, and its specificity was higher than, that of W4SS (sensitivity 77% [95% CI 61–88; p=0·71], specificity 74% [61–83; p=0·041]; table 3; figure 2). The sensitivity of chest x-ray (with any abnormality) was 72% (65–78) and specificity was 62% (51–71; table 3; appendix p 56). Cough (lasting ≥2 weeks), haemoglobin (<10 g/dL), BMI (<18·5 kg/m^2^), and lymphadenopathy had high specificities but low sensitivities, making them unsuitable to be explored further as screening tests.

**Table 3:** Indirect comparisons between each test and W4SS for the detection of tuberculosis in all participants (using culture as a reference standard)

		**Number of studies**	**Number of participants**	**Sensitivity (95% CI)**	**Specificity (95% CI)**	Difference from W4SS*	
						Sensitivity (p value)	Specificity (p value)
W4SS		21	15 597	82% (72–89)	42% (29–57)	..	..
C-reactive protein							
	≥10 mg/L	5	3571	77% (61–88)	74% (61–83)	0·71	0·041
	≥8 mg/L	5	3571	81% (68–89)	70% (57–81)	0·91	0·071
	≥5 mg/L	5	3571	87% (77–93)	60% (48–71)	0·51	0·27
Chest x-ray							
	With any abnormality	8	6195	72% (65–78)	62% (51–71)	0·26	0·13
	Suggestive of tuberculosis	8	6150	63% (57–70)	78% (67–86)	0·071	0·0049
Cough							
	Any	21	15 568	56% (48–63)	72% (65–79)	<0·0001	0·0006
	Lasting ≥2 weeks	17	10 906	38% (29–49)	84% (77–90)	<0·0001	<0·0001
Haemoglobin							
	<10 g/dL	9	5116	43% (33–54)	80% (73–85)	0·0006	0·0013
	<8 g/dL	9	5116	12% (9–16)	96% (93–97)	<0·0001	<0·0001
BMI (<18·5 kg/m^2^)		18	12 650	29% (22–38)	89% (84–92)	<0·0001	<0·0001
Lymphadenopathy		4	2391	31% (14–55)	90% (75–96)	0·0023	0·0018
Parallel strategies†							
	W4SS and C-reactive protein (≥10 mg/L)	5	3571	88% (63–97)	31% (13–57)	0·358	0·46
	W4SS and chest x-ray with abnormal findings	8	6186	94% (89–97)	20% (10–37)	0·0077	0·066
Sequential strategies†							
	W4SS then C-reactive protein (≥5 mg/L)	5	3571	70% (31–92)	75% (53–88)	0·55	0·0405
	W4SS then Xpert‡§	12	8557	58% (50–66)	99% (98–100)	..	..
Xpert for all‡§		12	8570	68% (57–76)	99% (98–99)	0·094¶	0·40¶

Indirect comparisons are based on all studies that assessed at least one of the W4SS or relevant screening tests. BMI=body-mass index. W4SS=WHO-recommended four-symptom screen.

* For Xpert for all, the comparator is W4SS then Xpert.

† For parallel strategies, two screening tests are offered at the same time; for sequential strategies, a second screening test is offered only if the first screening test is positive.

‡ Accuracy measures for entire algorithm using total Xpert (sputum or non-sputum sample Xpert result, or both); alternative algorithms are W4SS then single sputum Xpert (12 studies; 8556 participants; sensitivity 55% [95% CI 48–63], specificity 99% [99–100]) and single sputum Xpert alone (12 studies; 8569 participants; sensitivity 64% [53–74], specificity 99% [98–99]).

§ One study assessed Xpert and Xpert Ultra among 733 participants; sputum Xpert sensitivity was 57% (95% CI 47–67) and specificity was 99% (98–100), sputum Xpert Ultra sensitivity was 73% (62–81) and specificity was 98% (96–98), urine Xpert Ultra sensitivity was 27% (19–38) and specificity was 98% (96–99), and sputum and urine Xpert Ultra sensitivity was 75% (65–83) and specificity was 95% (94–97).

¶ Bivariate model did not converge; results from a model assuming no correlation between sensitivity and specificity.

Parallel strategies that combined W4SS with either chest x-ray (with any abnormality) or C-reactive protein (≥10 mg/L) had higher sensitivities and lower specificities than W4SS alone (table 3). A sequential strategy of W4SS followed by C-reactive protein (≥5 mg/L) had a lower sensitivity but higher specificity than W4SS alone. A sequential strategy of W4SS followed by chest x-ray (with any abnormality) had a sensitivity of 63% (54–71) and specificity of 73% (62–82); we did not assess this strategy further because of reduced sensitivity compared with W4SS alone.

The sensitivity of W4SS followed by Xpert was 58% (95% CI 50–66; table 3). The sensitivity of Xpert for all was 68% (95% CI 57–76). The specificities of both strategies—W4SS followed by Xpert and Xpert for all—were 99% (table 3). The sensitivity of sputum Xpert Ultra was higher than that of sputum Xpert (73% [95% CI 62–81] *vs* 57% [47–67]) and specificities were similar (98% [96–98] and 99% [98–100]) in the only study (unpublished) that compared both tests.^50^

Direct and indirect comparisons of individual tests were largely similar (appendix p 17); however, the lower sensitivity and higher specificity of chest x-ray (with any abnormality) than with W4SS were more pronounced in the direct comparison. Forest plots and summary ROC curves for all tests and screening strategies are provided in the appendix (pp 62–146). The point estimates for the specificities of C-reactive protein (≥10 mg/L cutoff) were numerically higher than those of W4SS in each individual study that had these data (appendix pp 62–63). Additional diagnostic accuracy measures are shown in the appendix (pp 23–28).

**Figure 3:**
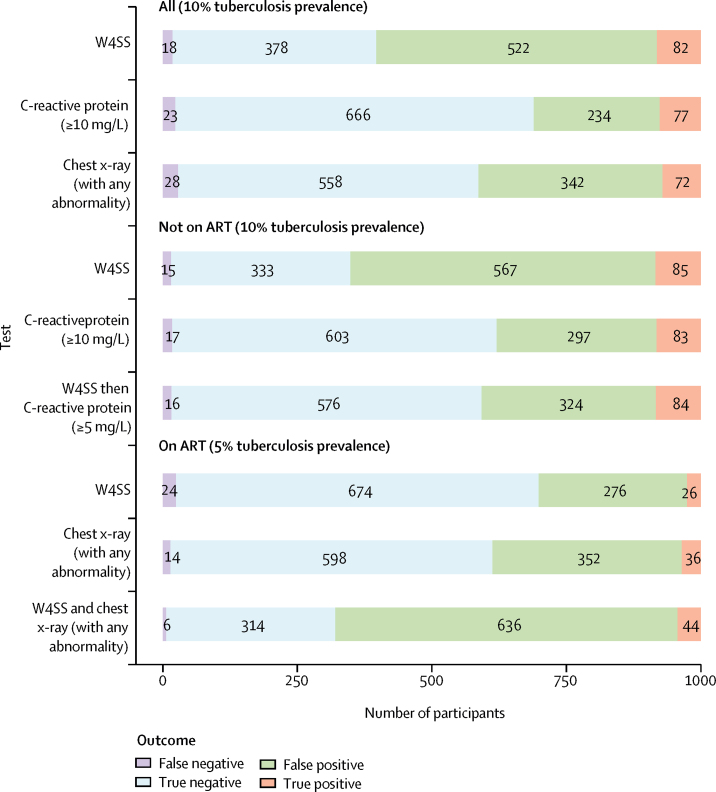
Screening outcomes for selected screening tests and strategies in a hypothetical cohort of 1000 people living with HIV at 10% (all and not on ART) and 5% (on ART) tuberculosis prevalence ART=antiretroviral therapy. W4SS=WHO-recommended four-symptom screen.

We assessed how estimates for each test or strategy affected detection rates in a hypothetical cohort of 1000 people living with HIV at different tuberculosis prevalences (appendix pp 29–46). At a tuberculosis prevalence of 10%, the W4SS would result in 604 rapid diagnostic tests being needed; C-reactive protein (≥10 mg/L) would reduce the number of rapid diagnostic tests needed by 293 but miss five additional tuberculosis cases, and chest x-ray (with any abnormality) would reduce the number of rapid diagnostic tests needed by 190, but miss ten additional tuberculosis cases (figure 3; appendix pp 29–31). At 10% prevalence, the WHO-recommended algorithm (W4SS followed by Xpert) would result in 604 Xpert tests, and Xpert for all would increase the number of Xpert tests needed by 396 (ie, because all 1000 people would receive an Xpert test), but it would detect ten additional tuberculosis cases (appendix pp 29–31).

Indirect comparisons by ART status are shown in the appendix (pp 12–13, 57–58). Most tests, except chest x-ray and haemoglobin, had lower sensitivity and higher specificity in outpatients on ART than in outpatients not on ART. In outpatients on ART, a parallel strategy of W4SS and chest x-ray (with any abnormality) had higher sensitivity than W4SS alone (89% [95% CI 70–97] *vs* 53% [35–71]) but lower specificity (33% [17–54] *vs* 71% [51–85]; appendix p 12). In a hypothetical cohort of 1000 outpatients on ART with 5% tuberculosis prevalence, this strategy would increase the number of rapid diagnostic tests needed by 378 compared with W4SS alone but detect 18 additional tuberculosis cases (figure 3; appendix pp 32–34).

In outpatients not on ART, sensitivities for C-reactive protein (≥10 mg/L) alone (83% [95% CI 79–86]) and a sequential strategy of W4SS then C-reactive protein (≥5 mg/L; 84% [75–90]) were similar to the sensitivity of W4SS alone (85% [76–91]), but their specificities were higher (67% [60–73] for C-reactive protein alone; 64% [57–71] for sequential strategy) than with W4SS alone (37% [25–51]; appendix p 13). In a hypothetical cohort of 1000 outpatients not on ART with 10% tuberculosis prevalence, compared with use of W4SS alone, use of C-reactive protein (≥10 mg/L) would reduce the number of rapid diagnostic tests needed by 272 but miss two additional tuberculosis cases, and use of the sequential strategy of W4SS then C-reactive protein (≥5 mg/L) would reduce the number of rapid diagnostic tests needed by 244 but miss one additional tuberculosis case (figure 3; appendix pp 35–37).

Indirect comparisons between each test and W4SS by CD4 cell count are shown in the appendix (pp 14–15, 59–60). Most tests, except chest x-ray, had lower sensitivity and higher specificity in participants with CD4 counts of more than 200 cells per μL than those with CD4 counts of 200 cells per μL or lower. Similarly, most tests had lower sensitivity and higher specificity in pregnant women living with HIV than in the overall population (appendix pp 16, 61); however, these estimates had suboptimal precision.

Indirect and direct comparisons for the subgroups were largely similar (appendix pp 12–16, 18–22). However, among outpatients on ART, the slightly higher sensitivity of chest x-ray (both with any abnormality and suggestive of tuberculosis) than of W4SS alone in indirect comparisons was attenuated in direct comparisons (appendix p 18). Only one study (n=381) among outpatients on ART assessed C-reactive protein (≥10 mg/L), for which there was a similar sensitivity and specificity compared with W4SS alone (appendix p 18).^49^

We did sensitivity analyses using two alternative reference standards: culture or Xpert, and Xpert alone (appendix pp 47–52). Results were largely similar to the main analyses, although sensitivities were slightly higher for the reference standard of Xpert alone than for the main reference standard of culture. In sensitivity analyses directly comparing W4SS followed by Xpert with C-reactive protein (≥10 mg) followed by Xpert, both strategies had similar sensitivities and specificities (appendix p 53).

Egger's test and meta-regression results are provided in the appendix (pp 23–28), as well as funnel plots (pp 147–154). We found no evidence of publication bias (Egger's test p>0·05) for most tests. Meta-regression showed that prevalence explained some heterogeneity in the analyses for several tests, but reference standard type generally did not.

## Discussion

In this systematic review and individual participant data meta-analysis, we found that the sensitivity of C-reactive protein (≥10 mg/L) was similar to that of W4SS alone, but its specificity was higher (74% *vs* 42%). Chest x-ray (with any abnormality) had lower sensitivity than W4SS alone in direct comparisons, making it less suitable than a standalone screening test. Cough (lasting ≥2 weeks), haemoglobin (<10 g/dL), BMI (<18·5 kg/m^2^), and lymphadenopathy had high specificities (>80%), but their low sensitivities also made them less suitable as screening tests than W4SS. The WHO-recommended algorithm of W4SS then Xpert had a sensitivity of only 58% (95% CI 50–66), and Xpert for all had a slightly higher sensitivity of 68% (57–76). In one unpublished study, Xpert Ultra improved sensitivity over Xpert (73% [62–81] *vs* 57% [47–67]).^50^

Among outpatients on ART, the sensitivity of a parallel strategy of W4SS with chest x-ray (any abnormality) was higher than that of W4SS alone, but its specificity was lower. At 5% tuberculosis prevalence, this strategy was estimated to require more than double the number of rapid diagnostic tests needed compared with W4SS alone but would detect 70% more tuberculosis cases. Among outpatients not on ART, the sensitivities of C-reactive protein (≥10 mg/L) and a sequential strategy of W4SS then C-reactive protein (≥5 mg/L) were similar to W4SS alone, but specificities were higher. At 10% tuberculosis prevalence, these strategies would reduce the number of rapid diagnostic tests needed by 42% for C-reactive protein (≥10 mg/L) and 37% for W4SS then C-reactive protein (≥5 mg/L) compared with W4SS alone, but would miss a similar number of tuberculosis cases.

We found that C-reactive protein (≥10 mg/L) approached the WHO-defined minimum thresholds for a screening test (with 83% sensitivity and 67% specificity *vs* WHO's thresholds of 90% sensitivity and 70% specificity) for outpatients not on ART.^8^ Efforts to scale-up of access to WHO-recommended, molecular, rapid diagnostic tests have been slow, particularly in decentralised locations.^54^, ^55^ C-reactive protein testing could allow for broader implementation of rapid diagnostic tests because its greater specificity means that screening using C-reactive protein would require fewer subsequent rapid diagnostic tests than screening with W4SS. The need for fewer tests could also reduce laboratory processing time; Xpert can provide a result in less than 2 h, but a result often takes several days in the real world.^56^ The high specificity of C-reactive protein would reduce the time to start tuberculosis preventive therapy in people living with HIV. Current C-reactive protein point-of-care assays have differing complexities, ranging from qualitative lateral-flow assays that do not require a power source or refrigeration to quantitative assays that require a small machine.^57^ C-reactive protein point-of-care assays can cost approximately US$2 per test, provide results in less than 3 min, and be performed easily with minimal expertise (blood collected by finger prick). Thus, available point-of-care assays have the potential for affordable scale-up.

The sensitivity of a parallel strategy incorporating W4SS and chest x-ray was higher than the sensitivity of other tests or strategies in those on ART; however, the higher number of rapid diagnostic tests needed might pose a substantial cost burden. Furthermore, a 2016 survey of 14 countries with high HIV-associated tuberculosis burdens found that chest x-ray as a screening tool was available at only 14% of primary health-care centres.^55^ We found that the sensitivity of chest x-ray was not increased in those not on ART and at lower CD4 cell counts of 200 per μL or lower; the most likely explanation for these findings is that normal chest x-ray images in patients with pulmonary and extra-pulmonary tuberculosis occur more frequently in those with advanced immunosuppression than in other people living with HIV.^58^, ^59^

The low sensitivities of haemoglobin, BMI, and lymphadenopathy make them unsuitable as screening tests. However, haemoglobin levels below 10 g/dL, a BMI of less than 18·5 kg/m^2^, and lymphadenopathy in ambulatory people living with HIV should prompt a thorough search for tuberculosis, given their high specificities and known association with mortality.^60^, ^61^

We found that the WHO-recommended strategy (W4SS followed by Xpert) would miss approximately 40% of tuberculosis cases. The low yield is a result of the inadequate sensitivities of both the W4SS and Xpert. Approximately 20% of people living with HIV with tuberculosis will be missed with W4SS and thus have subclinical tuberculosis, 56–75% of whom will probably progress to symptomatic disease.^62^, ^63^ Although Xpert for all would still miss approximately 33% of tuberculosis cases, Xpert Ultra showed improved sensitivity over Xpert in one study.^50^ Xpert Ultra costs the same as Xpert, and the point-of-care GeneXpert Omni platform might allow its use at decentralised locations. Further research is needed to assess this approach.

Our study has limitations. First, we did not have adequate precision in some analyses for outpatients on ART and pregnant people living with HIV. Specifically, we had little data on C-reactive protein in people living with HIV on ART. Furthermore, there was a paucity of data on countries other than South Africa, where almost half of all included studies were done, and which might be more urbanised than other low-income and middle-income countries. Second, we largely excluded participants who were unable to produce a sputum sample, meaning our findings might not generalise to this group. Few studies also systematically included extra-pulmonary tuberculosis samples, meaning our results are more applicable to pulmonary tuberculosis. However, pulmonary tuberculosis probably comprises most tuberculosis cases in an ambulatory screening setting. Third, we used an imperfect reference standard, because sputum culture, which was all that was done in most of the included studies, should ideally comprise multiple samples collected in the early morning to maximise sensitivity, but this was not done in any of our included studies. Fourth, although direct comparison minimises confounding, these analyses involved fewer studies and reduced precision. Fifth, we were unable to obtain individual participant data from three studies. However, these studies comprised only approximately 8% of data. Sixth, only one study assessed Xpert Ultra,^50^ and we did not assess non-Xpert nucleic acid amplification tests. Seventh, our study findings might not be generalisable to children with HIV and they might not be generalisable to all settings because most included studies were done in settings with high tuberculosis prevalence. Test performance might also vary in the context of regular screening. Finally, although calculations based on a hypothetical cohort give insight into consequences of testing, they were often based on heterogenous results.

Findings from this study have informed the updated 2021 WHO tuberculosis screening guidelines in people living with HIV.^64^ Compared with W4SS, C-reactive protein reduces the need for additional rapid diagnostic tests without compromising sensitivity, but there was a paucity of data for outpatients on ART. In outpatients not on ART, C-reactive protein assays could be used as a standalone screening test or combined with W4SS in a sequential strategy. In outpatients on ART, chest x-ray could be used in parallel with W4SS, depending on available resources, because this strategy detects more tuberculosis cases than does W4SS alone. Overall, the WHO-recommended screening and diagnostic algorithm (W4SS followed by Xpert) has suboptimal sensitivity; Xpert for all offers small improvements in sensitivity and would be resource intensive. Future research is needed to assess the utility of C-reactive protein screening in outpatients on ART and Xpert Ultra in all people living with HIV, and to investigate the cost-effectiveness of different screening tests and strategies. Because no test or strategy met both WHO-defined minimum sensitivity and specificity thresholds, improved screening tests for tuberculosis need to be developed for this population.

## Data sharing

The aggregate datasets and analysis code are available online. The study investigators of the original studies retain ownership of their data. Any requests for access to individual participant data should be made directly to study investigators of the original studies.

## Declaration of interests

AC reports grants from National Institutes of Health (NIH), Global Health Labs, and Stop TB Partnership, and consulting fees from the US Centers for Disease Control and Prevention (CDC). AK reports grants from Sanofi. FAK reports grants from WHO, Canadian Institutes of Health Research, Fonds de Recherche Quebec, and McGill Interdisciplinary Initiative on Infection and Immunity. GT reports receipt of consumables and equipment from Boditech and Cepheid. NM reports grants from Pfizer and Roche. REC reports grants from NIH, CDC, and Unitaid, and consulting fees from Sanofi. SML reports grants from NIH and CDC. SSk reports grants from Swedish Heart-Lung Foundation. TKr reports consulting fees from WHO. All other authors declare no competing interests.
