# Correction to *Lancet Infect Dis* 2022; 22: 507–18

**DOI:** 10.1016/S1473-3099(23)00263-3

**Published:** 2023-06

*Dhana A, Hamada Y, Kengne AP, et al. Tuberculosis screening among ambulatory people living with HIV: a systematic review and individual participant data meta-analysis.* Lancet Infect Dis *2022; **22:** 507–18*—In the Acknowledgments section of this Article, the authors have added the statement: “We acknowledge the provision of data by the AIDS Clinical Trials Group”. This correction has been made to the online version as of April 17, 2023.

