# Supplementary material for: Tuberculosis screening among ambulatory people living with HIV: a systematic review and individual participant data meta-analysis
Source: Lancet Infect Dis. 2022 Apr;22(4):507–18. doi: 10.1016/S1473-3099(21)00387-X (PMC8942858; doi:10.1016/S1473-3099(21)00387-X)
Supplement: Supplementary appendix [file mmc1.pdf]

THE LANCET
Infectious Diseases

Supplementary appendix

This appendix formed part of the original submission and has been peer reviewed. We post it as supplied by the authors.

Supplement to: Dhana A, Hamada Y, Kengne AP, et al. Tuberculosis screening among ambulatory people living with HIV: a systematic review and individual participant data meta-analysis. *Lancet Infect Dis* 2021; published online Nov 17. https://doi.org/10.1016/S1473-3099(21)00387-X.

Supplementary Figures and Tables

Table of Contents

Table S1 - Search terms 2
Table S2 - Variables sought 3
Table S3 - Study-level characteristics 4
Table S4 - Percentage of missing data for each variable by study†§ 6
Table S5 - Summary of main characteristics for participants overall and by each study 7
Table S6 - Prevalence of tuberculosis in all participants and by different subgroups (using culture or Xpert as reference standard) 11
Table S7 - Indirect comparisons between each test and WHO four-symptom screen for the detection of tuberculosis in subgroups 12
Table S8 - Direct comparisons between each test and WHO four-symptom screen for the detection of tuberculosis in all participants and subgroups 17
Table S9 - Additional diagnostic accuracy estimates 23
Table S10 - Yield of different screening and diagnostic algorithms when screening a population of 1000 persons 29
Table S11 - Sensitivity analyses using different reference standards 47
Table S12 - Sensitivity analyses comparing W4SS followed by Xpert with CRP (≥10mg) followed by Xpert in all participants and by subgroups† 53
Figure S1 - Risk of bias and applicability results on the QUADAS-2 criteria tool 54
Figure S2 - Plots of sensitivity and specificity for each screening test for the detection of tuberculosis 56
Figure S3 - Forest plots of sensitivity and specificity estimates in all participants and subgroups 62
Figure S4 - Summary receiver operating characteristics curves in all participants and subgroups (for tests/strategies with >=2 studies available) 122
Figure S5 - Funnel plots (for tests/strategies with >=10 studies available) 147
References 155

Table S1 - Search terms

Pubmed

#1.	“HIV Infections” [MeSH] OR “HIV”[MeSH] OR “hiv”[tw] OR hiv infect*[tw] OR “human immunodeficiency virus”[tw] OR “human immunedeficiency virus”[tw] OR “human immuno-deficiency virus”[tw] OR “human immune-deficiency virus”[tw] OR ((human immun*) AND (“deficiency virus”[tw])) OR “acquired immunodeficiency syndrome”[tw] OR “acquired immunedeficiency syndrome”[tw] OR “acquired immuno-deficiency syndrome”[tw] OR “acquired immune-deficiency syndrome”[tw] OR ((acquired immun*) AND (“deficiency syndrome”[tw]))
#2.	"Tuberculosis"[Mesh] OR tuberculosis [TW] OR "Mycobacterium tuberculosis"[Mesh] OR TB [Ti]
#3	Screening* OR algorithm* OR “case finding” [TIAB] OR “case findings” [TIAB] OR sensitivit* OR specificit* OR predictor* OR "Sensitivity and Specificity"[MeSH Terms] OR "Tuberculosis/diagnosis"[Mesh] OR "Mass Screening"[Mesh:NoExp]
#4.	("animals"[MeSH Terms] NOT ("humans"[MeSH Terms] AND "animals"[MeSH Terms])) OR case reports[Publication Type]
#5	#1 AND #2 AND #3 NOT #4
Limit: publication date from 2011/01/01	

Embase

#1	'human immunodeficiency virus infection'/exp OR 'human immunodeficiency virus'/exp OR 'hiv':ti,ab OR 'human immunodeficiency virus':ti,ab OR 'human immuno-deficiency virus':ti,ab OR 'human immunedeficiency virus':ti,ab OR 'human immune-deficiency virus':ti,ab OR 'acquired immune-deficiency syndrome':ti,ab OR 'acquired immunedeficiency syndrome':ti,ab OR 'acquired immunodeficiency syndrome':ti,ab OR 'acquired immuno-deficiency syndrome':ti,ab
#2	’tuberculosis‘/exp OR 'tuberculosis':ab,ti OR 'TB':ti OR 'Mycobacterium tuberculosis'/exp
#3	‘Screen’:ti,ab OR ‘Screening’:ti,ab OR ‘algorithm’:ti,ab OR ‘case finding’:ti,ab OR ‘case findings’:ti,ab OR sensitivit*:ti,ab OR specificit*:ti,ab OR predictor*:ti,ab OR ‘sensitivity and specificity’/exp OR ‘case finding’/exp OR ‘Mass Screening’/exp OR ‘screening’/exp
#4	([animals]/lim NOT [humans]/lim)
#5	#1 AND #2 AND #3 NOT #4 AND [2011-]/py

Cochrane

#1.	“HIV Infections” [MeSH] OR “HIV”[MeSH] OR hiv OR hiv infect* OR “human immunodeficiency virus” OR “human immunedeficiency virus”OR “human immuno-deficiency virus” OR “human immune-deficiency virus” OR ((human immun*) AND (“deficiency virus”)) OR “acquired immunodeficiency syndrome” OR “acquired immunedeficiency syndrome” OR “acquired immuno-deficiency syndrome” OR “acquired immune-deficiency syndrome” OR ((acquired immun*) AND (“deficiency syndrome”))
#2.	"Tuberculosis"[Mesh] OR tuberculosis OR "Mycobacterium tuberculosis"[Mesh]
#3	Screening* OR algorithm* OR “case finding” OR “case findings” OR sensitivit* OR specificit* OR predictor* OR "Sensitivity and Specificity"[MeSH Terms] OR "Tuberculosis/diagnosis"[Mesh] OR "Mass Screening"[Mesh:NoExp]
#4.	("animals"[MeSH Terms] NOT ("humans"[MeSH Terms] AND "animals"[MeSH Terms])) OR case reports[Mesh]
#5	#1 AND #2 AND #3 NOT #4
Limit: publication year from 2011-	

Table S2 - Variables sought

Variable	Description
country	country where the study took place, or if multisite, country individual patient was recruited from
clinical setting	from {inpatient, outpatient, other, NA}
age	patient's age in years
sex	patient's sex {female, male, NA}
hiv status	from {positive, negative, NA}
art status	from {on art, not on art, NA}
history of tuberculosis	from {history of tuberculosis, no history of tuberculosis, NA}
current smoking status	from {currently smoking, not currently smoking, NA}
pregnancy status	from {pregnant, not pregnant, NA}
tuberculosis treatment status	from {currently on tuberculosis treatment, not currently on tuberculosis treatment, NA}
current ipt status	from {yes, no, NA}
current cough	from {yes, no, NA}
cough (more than 2 weeks)	from {yes, no, NA}
fever	from {yes, no, NA}
weight loss	from {yes, no, NA}
night sweats	from {yes, no, NA}
w4ss	number of w4ss symptoms {0, 1, 2, 3, 4, NA}
body mass index	numerical value (weight/height^2)
lymphadenopathy	from {yes, no, NA}
cd4 count	numerical value (in cells/µL)
c-reactive protein level	numerical value (in mg/L)
haemoglobin	numerical value (in g/dl)
chest x-ray suggestive of tuberculosis	from {yes, no, NA}
chest x-ray abnormal	from {yes, no, NA}
sputum xpert result	{positive, negative, NA}, indeterminate = negative
sputum xpert ultra result	{positive, negative, NA}, indeterminate = negative
sputum culture result	{positive, negative, NA}, contaminated culture = negative
non-sputum xpert result	{positive, negative, NA}, indeterminate = negative
non-sputum xpert ultra result	{positive, negative, NA}, indeterminate = negative
non-sputum culture result	{positive, negative, NA}, contaminated culture = negative

Definition of abbreviations: ART = antiretroviral therapy, IPT = Isoniazid preventive therapy, W4SS = WHO four-symptom screen

Table S3 - Study-level characteristics

Author, year	Country	Study period	Study population	Study setting	Exclusion criteria	Sputum culture	Sputum Xpert	Non-sputum culture/Xpert	Liquid or solid culture
Abed Al-Darraji, 2013[1]	Malaysia	2012-2012	PLHIV who are inmates	1 prison in Malaysia	Receiving ATT, anticipated release within 24 hours	2 spot samples	1 morning sample	No	Liquid
Affolabi, 2018[2]	Multi-country*	2015-2018	PLHIV of any age	3 HIV clinics	On ATT for TB	1 spot and 1 early morning samples	1 spot and 1 early morning samples	No	Solid
Ahmad, 2014[3]	South Africa	2011-2012	PLHIV aged ≥18 years	2 HIV clinics	On ATT currently or awaiting results of TB investigations	1 morning sample	No	No	Liquid
Balcha, 2014[4]	Ethiopia	2011-2013	ART-naive PLHIV aged ≥18 years with WHO stage 4 or CD4 cell count <350 per µL	5 public health centers	Unable to submit ≥ 1 pair of sputum samples, previous ART, ATT >2 weeks	1 morning sample	1 morning sample	FNA sample of LN >1cm for culture and Xpert	Liquid
Bjerrum, 2015[5]	Ghana	2013-2014	ART-naive PLHIV aged ≥18 years with WHO stage 3/4 or CD4 cell count ≤350 per µL or pregnant	1 public hospital (out- and inpatient departments)	On ATT > 2 days <3 months before or unable to produce sputum or urine samples	1 spot and 1 early morning samples	1 or 2 samples	No	Both
Gersh, 2018[6]	Kenya	2017-2018	PLHIV aged ≥18 and ≤70 years	2 HIV clinics	Currently on treatment for TB or LTBI, pregnant, incarcerated	1 spot sample, induced or early morning if necessary	1 spot sample, induced or early morning if necessary	No	Liquid
Hanifa, 2012[7]	South Africa	2007-2008	ART-naive PLHIV aged >17 years with WHO stage 4 or CD4 cell count ≤200 per µL	1 public HIV clinic	On ATT currently or <3 months before	2 spot samples	No	No	Liquid
Heidebrecht, 2016[8]	South Africa	2013-2013	Inpatient PLHIV admitted to medical wards	1 hospital (5 medical wards)	≥3 doses of ATT	1 spot sample	1 spot sample	No	Both
Hoffman, 2013[9]	South Africa	2010-2011	Pregnant PLHIV aged ≥18 years	16 PHC centre prenatal clinics and 1 regional hospital	-	1 spot sample	No	No	Liquid
Kempker, 2019[10]	Georgia	2014-2015	Newly diagnosed ART-naive PLHIV aged ≥18 years	1 national reference center for HIV diagnosis and treatment	ATT <90 days before	1 spot and 1 early morning samples	1 spot and 1 early morning samples for 1 test	No	Solid
Kerkhoff, 2013[11]	South Africa	2010-2011	ART-naive PLHIV aged ≥18 years	1 community-based ART clinic	No current TB diagnosis	2 spot samples with ≥ 1 induced	2 spot samples with ≥ 1 induced	No	Liquid
Kufa, 2012[12]	South Africa	2009-2010	PLHIV aged ≥18 years	1 HIV clinic in PHC centre	On TB treatment, completed TB treatment <3 months before, dialysis, prisoners	2 spot samples, induced if necessary	No	Blood culture	Liquid
LaCourse, 2016[13]	Kenya	2013-2014	Pregnant PLHIV aged ≥16 years	2 antenatal care clinics	On ATT for TB or LTBI, or were treated for TB or LTBI <1 year before	1 spot and 1 early morning samples	1 spot sample, on 2nd sample if no spot sample or if 2nd sample culture positive for TB	No	Liquid
Mbu, 2018[14]	Cameroon	2012-2013	ART-naive PLHIV aged ≥18 years	1 regional hospital (HIV testing and ART treatment center)	First diagnosis of HIV <1 month before, currently on ATT	1 spot and 1 early morning samples	No	No	Both
Modi, 2016[15]	Kenya	2011-2012	ART-naïve PLHIV aged ≥7 years	15 public HIV care and treatment clinics	Receipt of any HIV-related care <2 years before, ATT <1 year before	1 spot and 1 early morning samples	1 spot and 1 early morning samples	No	Liquid

Author, year	Country	Study period	Study population	Study setting	Exclusion criteria	Sputum culture	Sputum Xpert	Non-sputum culture/Xpert	Liquid or solid culture
Nguyen, 2016[16]	Vietnam	2009-2010	PLHIV aged ≥15 years	1 outpatient urban HIV clinic	Screened for TB in past month, received TB treatment <1 year before	1 spot and 1 early morning samples	No	No	Solid
Rangaka, 2012[17]	South Africa	2007-2009	PLHIV aged ≥18 years	1 HIV clinic	-	1 spot sample, induced if necessary	No	No	Both
Reeve, 2019[18]	South Africa	2017-2020	ART-naive PLHIV aged ≥18 years	1 outpatient clinic	On ATT <60 days before or has unknown treatment status	2 spot samples, majority induced	1 spot sample and Xpert Ultra on 1 spot sample	Urine Xpert Ultra	Liquid
Shapiro, 2018[19]	South Africa	2014-2015	ART-naive PLHIV aged ≥18 years	1 urban HIV clinic	-	2 samples, induced if necessary	No	No	Liquid
Swindells, 2013[20]	Multi-country**	2010-2010	ART-naive PLHIV aged ≥13 years	11 outpatient clinics	ART or diagnosis of TB <90 days before and current or recent receipt of ATT	3 spot samples, induced if necessary	No	No	Both
Thit, 2017[21]	Myanmar	2015-2015	Inpatient or outpatient PLHIV	1 tertiary referral hospital	-	1 spot sample	1 spot sample	No	Solid
Yoon, 2018[22]	Uganda	2013-2016	ART-naive PLHIV aged ≥18 years with CD4 cell count ≤350 per μL	2 HIV clinics in Kampala	Diagnosis of active tuberculosis, taking ATT (anti-TB or TB preventive therapy, fluoroquinolones) ≤3 days before	2 spot samples, 2nd induced if necessary	1 spot sample, induced if necessary	No	Both

*Benin, Guinea, and Senegal

**Botswana, Malawi, South Africa, Zimbabwe, India, Brazil and Peru

Definition of abbreviations: ART = antiretroviral therapy, ATT = anti-tuberculosis treatment, FNA = fine needle aspiration, LN = Lymph node, LTBI = latent tuberculosis infection, PHC = primary health care, PLHIV = people living with HIV, TB = tuberculosis

Table S4 - Percentage of missing data for each variable by study†§

Variable	Affolabi	Ahmad	Al_Darraji	Balcha	Bjerrum	Gersh	Hanifa	Hoffmann	Kemper	Kerkhoff	Kufa	LaCourse	Mbu	Modi	Nguyen	Rangaka	Reeve	Shapiro	Swindells	Thit	Yoon
**Clinical setting**	0	0	0	0	0	0	0	0	0	0	0	0	0	0	0	0	0	0	0	0	0
**Age**	0	0	0	0	0	0	0	0	0	0	0	0	0	0	0	0	0	0	0	0	0
**Sex**	0	0	0	0	0	0	0	0	0	0	0	0	0	0	0	0	0	0	0	0	0
**ART status**	0	0	0	0	0	1	0	0	0	0	0	0	0	0	0	0	0	0	0	0	0
**History of tuberculosis**	100	0	0	1	1	1	0	0	0	0	0	6	100	100	0	0	0	0	0	0	0
**Currently smoking**	0	100	0	0	24	0	0	0	0	0	100	100	100	100	1	100	0	0	100	0	8
**Pregnancy***	100	100	100	100	0	0	100	0	100	1	0	0	100	4	0	100	100	100	0	100	0
**Currently on IPT**	1	0	100	0	100	0	100	100	100	100	100	0	100	100	100	100	100	100	0	0	0
**W4SS****	0	0	0	0	0	0	0	0	0	0	0	0	0	2	0	0	0	0	0	0	0
Cough	0	0	0	0	0	0	0	0	0	0	0	0	0	5	0	0	0	0	0	0	0
Fever	0	0	0	0	2	0	0	0	0	0	0	0	0	4	0	0	0	0	0	0	0
Weight loss	0	0	0	0	1	0	0	0	0	0	0	0	0	3	0	0	0	0	4	0	0
Night sweats	0	0	0	0	1	0	0	0	0	0	0	0	0	4	0	0	0	0	0	0	0
**Cough >=2 weeks**	100	100	0	100	0	0	0	0	0	0	5	0	0	4	0	0	0	0	0	100	0
**Body mass index**	1	6	0	1	1	1	1	100	2	0	3	0	100	13	100	0	0	0	1	0	0
**Lymphadenopathy**	100	100	100	0	0	100	100	100	100	100	100	100	100	100	100	100	100	100	0	0	100
**CD4 cell count**	3	0	0	1	2	18	1	2	0	0	0	17	0	7	0	0	10	3	0	0	0
**CRP**	100	100	100	100	100	1	100	100	100	4	100	100	100	100	100	100	8	0	100	100	0
**Hb**	100	100	100	6	9	5	1	8	100	6	0	100	100	100	100	100	100	100	0	21	100
**CXR (any abnormality)**	3	3	100	100	100	100	1	100	100	9	7	100	100	23	100	100	100	100	4	7	100
**CXR (suggests tuberculosis)**	3	3	100	100	100	100	1	100	100	9	8	100	100	23	0	100	100	100	4	100	100
**Total Xpert*****	1	100	0	1	65	1	100	100	1	0	100	1	100	0	100	100	6	100	100	0	0
**Total culture**	1	0	0	0	0	0	0	0	1	0	0	0	0	0	0	0	1	0	0	0	0
**Total (culture±Xpert)**	0	0	0	0	0	0	0	0	0	0	0	0	0	0	0	0	0	0	0	0	0

†<5% missing (green), 5-95% missing (yellow), and >95% missing (red)

§Some datasets received in which some participants with missing data were already excluded

*Pregnancy could be ascertained by testing or interview; missing percentages based on available data for females in the study

**Regarded as missing only if a subject had all four symptoms missing

***Study by Bjerrum et al has a high missing value because Xpert only became available after study enrollment began

Definition of abbreviations: ART = antiretroviral therapy, CRP = C-reactive protein, CXR = chest X-ray, Hb = haemoglobin, IPT = Isoniazid preventive therapy, W4SS = WHO four-symptom screen

Table S5 - Summary of main characteristics for participants overall and by each study

Variable	All	Ahmad	Al Darraji	Balcha	Bjerrum	Gersh	Hanifa	Hoffmann	Kemper	Kerkhoff	Kufa
**Participants**	15666 (100)	611 (3.9)	125 (0.8)	812 (5.2)	395 (2.5)	387 (2.5)	351 (2.2)	1404 (9)	103 (0.7)	523 (3.3)	415 (2.6)
**Clinical setting**											
**Outpatient**	15541 (99.2)	611 (100)	0 (0)	812 (100)	395 (100)	387 (100)	351 (100)	1404 (100)	103 (100)	523 (100)	415 (100)
**Other setting***	125 (0.8)	0 (0)	125 (100)	0 (0)	0 (0)	0 (0)	0 (0)	0 (0)	0 (0)	0 (0)	0 (0)
**N**	15666	611	125	812	395	387	351	1404	103	523	415
**Age (years)**	34 (28-42)	37 (31-45)	37 (33-42)	32 (28-40)	38 (31-45)	37 (31-45)	38 (32-46)	27 (23-32)	42 (35-49)	34 (28-41)	37 (31-44)
**N**	15666	611	125	812	395	387	351	1404	103	523	415
**Female**	10388 (66.3)	461 (75.5)	12 (9.6)	476 (58.6)	264 (66.8)	225 (58.1)	232 (66.1)	1404 (100.0)	26 (25.2)	335 (64.1)	274 (66.0)
**N**	15666	611	125	812	395	387	351	1404	103	523	415
**On ART**	4347 (27.8)	423 (69.2)	19 (15.2)	0 (0.0)	0 (0.0)	383 (99.7)	0 (0.0)	2 (0.1)	0 (0.0)	0 (0.0)	210 (50.6)
**N**	15663	611	125	812	395	384	351	1404	103	523	415
**CD4 count (cells/μL)**	269 (142-439)	313 (178-472)	338 (150-492)	208 (117-320)	140 (45-268)	404 (276-558)	119 (72-168)	395 (271-533)	120 (26-272)	169 (96-232)	216 (108-350)
**N**	15281	611	125	807	387	317	349	1377	103	521	415
**History of tuberculosis**	1955 (17.5)	235 (38.5)	36 (28.8)	51 (6.4)	23 (5.9)	58 (15.1)	99 (28.2)	110 (7.9)	3 (2.9)	141 (27.0)	109 (26.3)
**N**	11148	611	125	800	392	384	351	1401	103	523	415
**Current Smoker**	1191 (11.6)	-	117 (93.6)	35 (4.3)	11 (3.7)	11 (2.8)	40 (11.4)	40 (2.8)	64 (62.1)	122 (23.4)	-
**N**	10301		125	812	299	387	351	1404	103	522	
**Pregnant**	1938 (35.1)	-	-	-	24 (6.1)	0 (0.0)	-	1404 (100.0)	-	20 (3.8)	11 (2.7)
**N**	5519				395	387		1404		521	415
**On IPT**	41 (0.5)	3 (0.5)	-	19 (2.3)	-	0 (0.0)	-	-	-	-	-
**N**	7593	609		810		387					
**W4SS**	8028 (51.3)	331 (54.2)	85 (68.0)	651 (80.2)	359 (91.1)	47 (12.1)	331 (94.3)	227 (16.2)	62 (60.2)	452 (86.4)	355 (85.5)
**N**	15652	611	125	812	394	387	351	1404	103	523	415
**Cough**	4629 (29.6)	223 (36.5)	62 (49.6)	326 (40.2)	172 (43.7)	36 (9.3)	161 (45.9)	110 (7.8)	29 (28.2)	260 (49.7)	201 (48.4)
**N**	15623	611	125	811	394	387	351	1404	103	523	415
**Fever**	3391 (21.7)	57 (9.3)	37 (29.6)	389 (48.0)	189 (48.6)	5 (1.3)	100 (28.5)	52 (3.7)	55 (53.4)	152 (29.1)	163 (39.3)
**N**	15631	611	125	811	389	387	351	1404	103	523	415
**Weight loss**	5575 (35.7)	170 (27.8)	19 (15.2)	514 (63.6)	323 (82.2)	9 (2.3)	316 (90.3)	103 (7.3)	43 (41.7)	355 (68.0)	254 (61.2)
**N**	15602	611	125	808	393	387	350	1404	103	522	415
**Night sweats**	3270 (20.9)	144 (23.6)	30 (24.0)	396 (48.8)	131 (33.5)	18 (4.7)	147 (41.9)	49 (3.5)	26 (25.2)	210 (40.2)	190 (45.8)
**N**	15630	611	125	811	391	387	351	1404	103	523	415
**Cough >= 2 weeks**	2205 (20.2)	-	14 (11.2)	-	130 (33.0)	8 (2.1)	103 (29.3)	54 (3.8)	28 (27.2)	107 (20.5)	151 (38.1)
**N**	10919		125		394	387	351	1404	103	523	396
**Lymphadenopathy**	374 (15.6)	-	-	28 (3.5)	76 (19.2)	-	-	-	-	-	-
**N**	2394			810	395						
**CXR (suggests tuberculosis)**	1296 (21.0)	265 (44.8)	-	-	-	-	177 (50.9)	-	-	196 (41.3)	72 (18.8)
**N**	6177	591					348			475	383
**CXR (any abnormality)**	2158 (34.7)	354 (59.9)	-	-	-	-	222 (63.8)	-	-	239 (50.1)	145 (37.8)
**N**	6222	591					348			477	384
**Total Xpert positive****	616 (7.1)	-	8 (6.4)	96 (11.9)	21 (15.1)	4 (1.0)	-	-	12 (11.8)	70 (13.4)	-
**N**	8625		125	804	139	384			102	523	

Variable	All	Ahmad	Al Darraji	Balcha	Bjerrum	Gersh	Hanifa	Hoffmann	Kemper	Kerkhoff	Kufa
**Total culture positive*** **	1347 (8.6)	57 (9.3)	15 (12.0)	124 (15.3)	37 (9.4)	5 (1.3)	64 (18.2)	35 (2.5)	12 (11.8)	89 (17.0)	24 (5.8)
**N**	15611	611	125	809	395	387	351	1404	102	522	415
**Total Xpert & culture positive**	1453 (9.3)	57 (9.3)	15 (12.0)	137 (16.9)	40 (10.1)	8 (2.1)	64 (18.2)	35 (2.5)	15 (14.6)	95 (18.2)	24 (5.8)
**N**	15666	611	125	812	395	387	351	1404	103	523	415
**BMI (kg/m²)**	22 (19-26)	24 (21-28)	22 (21-25)	19 (17-21)	20 (18-23)	22 (20-25)	20 (18-24)	-	22 (21-24)	23 (21-27)	24 (21-28)
**N**	12704	575	125	801	390	383	349	-	101	522	403
**CRP (mg/L)**	4 (2-21)	-	-	-	-	1 (1-4)	-	-	-	10 (2-32)	-
**N**	3582	-	-	-	-	385	-	-	-	502	-
**CRP (>=10 mg/L)**	1259 (35.1)	-	-	-	-	39 (10.1)	-	-	-	251 (50.0)	-
**N**	3582					385				502	
**Hb (g/dL)**	12 (10-13)	-	-	12 (10-13)	10 (9-11)	13 (11-14)	11 (10-13)	11 (10-12)	-	12 (11-13)	12 (11-14)
**N**	5118	-	-	762	360	366	348	1287	-	490	415
**Hb (<10 g/dL)**	1093 (21.4)	-	-	165 (21.7)	184 (51.1)	53 (14.5)	91 (26.1)	204 (15.9)	-	86 (17.6)	64 (15.4)
**N**	5118			762	360	366	348	1287		490	415

†Data are median (25th-75th percentiles) or count (%)

*One study among a prison population

**Sputum and/or non-sputum Xpert result

***Sputum and/or non-sputum culture result

Definition of abbreviations: ART = antiretroviral therapy, BMI = body mass index, CRP = C-reactive protein, CXR = chest X-ray, Hb = haemoglobin, IPT = Isoniazid preventive therapy, W4SS = WHO four-symptom screen

Variable	LaCourse	Mbu	Affolabi	Modi	Nguyen	Rangaka	Reeve	Shapiro	Swindells	Thit	Yoon
**Participants**	292 (1.9)	940 (6)	2805 (17.9)	731 (4.7)	397 (2.5)	1429 (9.1)	807 (5.2)	425 (2.7)	726 (4.6)	463 (3)	1525 (9.7)
**Clinical setting**											
**Outpatient**	292 (100)	940 (100)	2805 (100)	731 (100)	397 (100)	1429 (100)	807 (100)	425 (100)	726 (100)	463 (100)	1525 (100)
**Other setting***	0 (0)	0 (0)	0 (0)	0 (0)	0 (0)	0 (0)	0 (0)	0 (0)	0 (0)	0 (0)	0 (0)
**N**	292	940	2805	731	397	1429	807	425	726	463	1525
**Age (years)**	25 (22-30)	35 (28-42)	40 (33-49)	30 (24-39)	30 (27-34)	34 (30-40)	32 (26-39)	32 (27-39)	33 (28-39)	34 (30-40)	33 (27-40)
**N**	292	940	2805	731	397	1429	807	425	726	463	1525
**Female**	292 (100.0)	641 (68.2)	1878 (67.0)	481 (65.8)	113 (28.5)	1053 (73.7)	478 (59.2)	248 (58.4)	458 (63.1)	231 (49.9)	806 (52.9)
**N**	292	940	2805	731	397	1429	807	425	726	463	1525
**On ART**	159 (54.5)	0 (0.0)	1808 (64.5)	0 (0.0)	230 (57.9)	775 (54.2)	0 (0.0)	0 (0.0)	0 (0.0)	338 (73.0)	0 (0.0)
**N**	292	940	2805	731	397	1429	807	425	726	463	1525
**CD4 count (cells/μL)**	437 (345-560)	291 (116-496)	376 (197-551)	342 (168-512)	336 (217-489)	209 (145-331)	306 (174-489)	306 (176-468)	273 (167-435)	286 (145-466)	160 (70-265)
**N**	242	940	2731	683	397	1429	726	411	723	462	1525
**History of tuberculosis**	25 (9.1)	-	-	-	178 (44.8)	552 (38.6)	115 (14.3)	19 (4.5)	53 (7.3)	94 (20.3)	54 (3.5)
**N**	274				397	1429	806	425	725	463	1524
**Current Smoker**	-	-	102 (3.6)	-	42 (10.7)	-	295 (36.6)	105 (24.8)	-	137 (29.7)	70 (5.0)
**N**			2805		393		807	424		462	1407
**Pregnant**	292 (100.0)	-	-	134 (29.1)	0 (0.0)	-	-	-	10 (1.4)	-	43 (5.3)
**N**	292			461	113				726		805
**On IPT**	0 (0.0)	-	7 (0.3)	-	-	-	-	-	0 (0.0)	12 (2.6)	0 (0.0)
**N**	292		2781						726	463	1525
**W4SS**	57 (19.5)	812 (86.4)	780 (27.8)	366 (50.9)	147 (37.0)	206 (14.4)	442 (54.8)	279 (65.6)	481 (66.3)	223 (48.2)	1335 (87.5)
**N**	292	940	2805	719	397	1429	807	425	725	463	1525
**Cough**	44 (15.1)	448 (47.7)	567 (20.2)	171 (24.6)	105 (26.4)	125 (8.7)	249 (30.9)	191 (44.9)	311 (43.0)	74 (16.0)	764 (50.1)
**N**	292	940	2805	694	397	1429	807	425	723	462	1525
**Fever**	14 (4.8)	220 (23.4)	444 (15.8)	236 (33.5)	26 (6.5)	9 (0.6)	55 (6.8)	129 (30.4)	222 (30.7)	61 (13.2)	776 (50.9)
**N**	292	940	2805	705	397	1429	807	425	724	463	1525
**Weight loss**	3 (1.0)	592 (63.0)	395 (14.1)	241 (34.0)	79 (19.9)	112 (7.8)	334 (41.4)	146 (34.4)	265 (38.2)	193 (41.7)	1109 (72.7)
**N**	292	940	2805	708	397	1429	806	425	694	463	1525
**Night sweats**	20 (6.8)	395 (42.0)	140 (5.0)	182 (26.0)	11 (2.8)	71 (5.0)	191 (23.7)	139 (32.7)	210 (29.0)	40 (8.6)	530 (34.8)
**N**	292	940	2805	701	397	1429	807	425	725	463	1525
**Cough >= 2 weeks**	14 (4.8)	228 (24.3)	-	138 (19.7)	105 (26.4)	82 (5.7)	166 (20.6)	129 (30.5)	229 (31.6)	-	519 (34.0)
**N**	292	940		699	397	1429	807	423	724		1525
**Lymphadenopathy**	-	-	-	-	-	-	-	-	238 (32.8)	32 (6.9)	-
**N**									726	463	
**CXR (suggests tuberculosis)**	-	-	251 (9.2)	99 (17.6)	127 (32.0)	-	-	-	109 (15.6)	-	-
**N**			2724	561	397				698		
**CXR (any abnormality)**	-	-	635 (23.3)	248 (43.8)	-	-	-	-	159 (22.8)	156 (36.1)	-
**N**			2726	566					698	432	
**Total Xpert positive****	4 (1.4)	-	111 (4.0)	61 (8.4)	-	-	61 (8.0)	-	-	25 (5.4)	143 (9.4)
**N**	289		2781	728			762			463	1525
**Total culture positive*****	7 (2.4)	131 (13.9)	85 (3.1)	75 (10.3)	28 (7.1)	126 (8.8)	93 (11.7)	42 (9.9)	56 (7.7)	10 (2.2)	232 (15.2)

Variable	LaCourse	Mbu	Affolabi	Modi	Nguyen	Rangaka	Reeve	Shapiro	Swindells	Thit	Yoon
**N**	292	940	2766	731	397	1429	796	425	726	463	1525
**Total Xpert & culture positive**	8 (2.7)	131 (13.9)	118 (4.2)	82 (11.2)	28 (7.1)	126 (8.8)	100 (12.4)	42 (9.9)	56 (7.7)	30 (6.5)	242 (15.9)
**N**	292	940	2805	731	397	1429	807	425	726	463	1525
**BMI (kg/m²)**	24 (22-26)	-	22 (19-25)	21 (19-22)	-	25 (22-29)	24 (21-29)	24 (21-29)	22 (20-26)	21 (19-23)	21 (19-24)
**N**	292	-	2769	634	-	1429	803	425	719	461	1523
**CRP (mg/L)**	-	-	-	-	-	-	6 (2-28)	4 (1-17)	-	-	4 (2-23)
**N**	-	-	-	-	-	-	745	425	-	-	1525
**CRP (>=10 mg/L)**	-	-	-	-	-	-	303 (40.7)	140 (32.9)	-	-	526 (34.5)
**N**							745	425			1525
**Hb (g/dL)**	-	-	-	-	-	-	-	-	12 (11-14)	11 (9-12)	-
**N**	-	-	-	-	-	-	-	-	724	366	-
**Hb (<10 g/dL)**	-	-	-	-	-	-	-	-	113 (15.6)	133 (36.3)	-
**N**									724	366	

†Data are median (25th-75th percentiles) or Count (%)

*One study among a prison population

**Sputum and/or non-sputum Xpert result

***Sputum and/or non-sputum culture result

Definition of abbreviations: ART = antiretroviral therapy, BMI = body mass index, CRP = C-reactive protein, CXR = chest X-ray, Hb = haemoglobin, IPT = Isoniazid preventive therapy, W4SS = WHO four-symptom screen

Table S6 - Prevalence of tuberculosis in all participants and by different subgroups (using culture or Xpert as reference standard)

					Heterogeneity			
**Subgroup§**	**No studies**	**N**	**No tuberculosis**	**Prevalence % (95% CI)†**	**I² (95% CI)**	**P-value**	**Egger's test (p-value)**	**Subgroup analysis (p-value)††**
**All**	**21**	**15,666**	**1,453**	**8.7 (6.7-11.3)**	**95 (94-96)**	**<0.0001**	**0.06**	**-**
**All (setting and ART status)**	**21**	**15,663**	**1,453**	**8.7 (6.7-11.3)**	**95 (94-96)**	**<0.0001**	**0.06**	**-**
Outpatients (on ART)*	9	4,328	169	4.2 (3.1-5.6)	75 (52-87)	<0.0001	0.78	<0.0001
Outpatients (not on ART)	20	11,210	1,269	10.2 (8.0-12.9)	92 (89-94)	<0.0001	0.06	-
Other setting**	1	125	15	12.0 (7.4-19.0)	- (-)	-	-	-
**All (CD4 count)**	**21**	**15,281**	**1,423**	**8.7 (6.7-11.2)**	**95 (93-96)**	**<0.0001**	**0.06**	**-**
CD4 count <=200 cells/µL	21	5,641	922	15.1 (12.8-17.7)	82 (73-88)	<0.0001	0.05	<0.0001
CD4 count >200 cells/µL	21	9,640	501	5.5 (4.2-7.1)	88 (84-92)	<0.0001	0.42	-
**All (pregnancy status)*** **	**21**	**10,388**	**746**	**7.0 (5.3-9.2)**	**91 (88-94)**	**<0.0001**	**0.22**	**-**
Pregnant	8	1,938	56	2.9 (2.2-3.7)	16 (0-59)	<0.0001	0.09	<0.0001
Non-pregnant	19	8,450	690	8.0 (6.2-10.3)	89 (85-92)	<0.0001	0.32	-

§Subgroup in bold is the overall comparator. For example, all (setting and ART status) contains combined subgroups outpatients (on ART), outpatients (not on ART), and other setting

†Calculated using meta-analysis of proportions. We used a generalized linear mixed model with logit transformation in preference to protocol specified DerSimonian and Laird random effects model for proportions with variance stabilization by applying the Freeman-Tukey double arcsine transformation

††P-value of between subgroups heterogeneity statistic Q (based on random effects model)

*P(subgroup) compares outpatients (on ART) with outpatients (not on ART)

**One study among a prison population

***Pregnancy status unavailable for some studies, female participants in those studies categorized as non-pregnant

Definition of abbreviations: ART = antiretroviral therapy

Table S7 - Indirect comparisons between each test and WHO four-symptom screen for the detection of tuberculosis in subgroups

Table S7A - Indirect comparisons between each test and WHO four-symptom screen for the detection of tuberculosis in outpatients (on ART)†

					Difference from W4SS††	
**Test**	**No studies**	**N**	**Sensitivity (95% CI)**	**Specificity (95% CI)**	**Sensitivity (p-value)**	**Specificity (p-value)**
**W4SS**	9	4309	53 (35-71)	71 (51-85)	-	-
**CRP (>=10 mg/L)**	1	381	20 (3-69)	90 (93-87)	0.296	0.29
**CRP (>=8 mg/L)**	1	381	40 (10-80)	89 (92-86)	0.689	0.342
**CRP (>=5 mg/L)**	1	381	40 (10-80)	80 (84-75)	0.689	0.697
**CXR (abnormal)**	4	2670	73 (60-83)	63 (50-75)	0.142	0.59
**CXR (suggests tuberculosis)**	4	2581	70 (55-82)	78 (62-89)	0.188	0.571
**Cough (any)**	9	4309	40 (24-58)	83 (73-90)	0.286	0.206
**Cough (>=2 weeks)**	6	1746	19 (5-52)	93 (79-98)	0.054	0.031
**Hb (<10 g/dL)**	4	844	55 (34-75)	82 (70-91)	0.996	0.315
**Hb (<8 g/dL)**	4	844	18 (5-49)	94 (91-96)	0.057	0.013
**BMI (<18.5 kg/m²)**	7	4036	16 (8-30)	93 (88-96)	0.004	0.007
**Lymphadenopathy**	1	338	22 (6-58)	93 (95-89)	0.278	0.204
**W4SS with CRP (>=10 mg/L)¶**	1	381	20 (3-69)	80 (83-75)	0.296	0.706
**W4SS with CXR (abnormal)¶**	4	2670	89 (70-97)	33 (17-54)	0.014	0.036
**W4SS then CRP (>=5 mg/L)¶**	1	381	8 (1-62)	96 (97-93)	0.04	0.09
**W4SS then Xpert***	4	2645	37 (25-52)	100 (96-100)	-	-
**Xpert alone***	4	2645	53 (22-83)	99 (97-99)	0.219	0.306

†Using culture as a reference standard. Indirect comparisons are based on all studies that evaluated at least one of the W4SS or relevant screening tests

††For Xpert alone, the comparator is W4SS then Xpert

¶For parallel strategies, two screening tests are offered at the same time. For sequential strategies, a second screening test is offered only if the first screening test is positive

*Accuracy measures for entire algorithm using total Xpert (sputum and/or non-sputum Xpert result). Alternative algorithms are W4SS then single sputum Xpert (4 studies; 2645 participants; sensitivity 37 (25-52), specificity 100 (96-100) and single sputum Xpert alone (4 studies; 2645 participants; sensitivity 53 (22-83), specificity 99 (97-99).

Definition of abbreviations: BMI = body mass index, CRP = C-reactive protein, CXR = chest X-ray, Hb = haemoglobin, W4SS = WHO four-symptom screen

Table S7B - Indirect comparisons between each test and WHO four-symptom screen for the detection of tuberculosis in outpatients (not on ART)†

					Difference from W4SS††	
**Test**	**No studies**	**N**	**Sensitivity (95% CI)**	**Specificity (95% CI)**	**Sensitivity (p-value)**	**Specificity (p-value)**
**W4SS**	20	11160	85 (76-91)	37 (25-51)	-	-
**CRP (>=10 mg/L)**	5	3187	83 (79-86)	67 (60-73)	0.953	0.085
**CRP (>=8 mg/L)**	5	3187	85 (81-88)	63 (56-70)	0.8	0.135
**CRP (>=5 mg/L)**	5	3187	89 (86-92)	53 (46-61)	0.318	0.375
**CXR (abnormal)**	8	3525	71 (64-78)	62 (51-72)	0.102	0.034
**CXR (suggests tuberculosis)**	8	3569	62 (55-68)	79 (67-87)	0.01	0.001
**Cough (any)**	20	11131	58 (50-65)	70 (63-76)	<0.0001	<0.0001
**Cough (>=2 weeks)**	16	9032	43 (34-52)	82 (75-87)	<0.0001	<0.0001
**Hb (<10 g/dL)**	9	4269	44 (32-56)	78 (70-85)	<0.0001	<0.0001
**Hb (<8 g/dL)**	9	4269	11 (8-15)	96 (93-98)	<0.0001	<0.0001
**BMI (<18.5 kg/m²)**	17	8486	31 (23-41)	87 (82-91)	<0.0001	<0.0001
**Lymphadenopathy**	4	2053	30 (13-55)	91 (76-97)	0.001	<0.0001
**W4SS with CRP (>=10 mg/L)¶**	5	3187	94 (87-97)	20 (12-33)	0.113	0.155
**W4SS with CXR (abnormal)¶**	8	3516	95 (91-97)	18 (9-33)	0.007	0.064
**W4SS then CRP (>=5 mg/L)¶**	5	3187	84 (75-90)	64 (57-71)	0.793	0.056
**W4SS then Xpert*§**	11	5784	64 (57-71)	99 (98-99)	-	-
**Xpert alone*§**	11	5797	74 (64-82)	99 (98-99)	0.199	0.59

†Using culture as a reference standard. Indirect comparisons are based on all studies that evaluated at least one of the W4SS or relevant screening tests

††For Xpert alone, the comparator is W4SS then Xpert

¶For parallel strategies, two screening tests are offered at the same time. For sequential strategies, a second screening test is offered only if the first screening test is positive

*Accuracy measures for entire algorithm using total Xpert (sputum and/or non-sputum Xpert result). Alternative algorithms are W4SS then single sputum Xpert (11 studies; 5783 participants; sensitivity 61 (53-69), specificity 99 (98-99) and single sputum Xpert alone (11 studies; 5796 participants; sensitivity 70 (58-80), specificity 99 (98-99).

§One study assessed Xpert and Xpert Ultra among 733 participants. The accuracy of sputum Xpert was: sensitivity 57 (47-67), specificity 99 (98-100); sputum Xpert Ultra: sensitivity 73 (62-81), specificity 98 (96-98); urine Xpert Ultra: sensitivity 27 (19-38), specificity 98 (96-99); sputum and urine Xpert Ultra: sensitivity 75 (65-83), specificity 95 (94-97)

Definition of abbreviations: BMI = body mass index, CRP = C-reactive protein, CXR = chest X-ray, Hb = haemoglobin, W4SS = WHO four-symptom screen

Table S7C - Indirect comparisons between each test and WHO four-symptom screen for the detection of tuberculosis in participants with CD4 cell count <=200 cells/µL†

					Difference from W4SS††	
**Test**	**No studies**	**N**	**Sensitivity (95% CI)**	**Specificity (95% CI)**	**Sensitivity (p-value)**	**Specificity (p-value)**
**W4SS**	21	5617	87 (77-93)	33 (20-48)	-	-
**CRP (>=10 mg/L)**	5	1593	88 (82-92)	62 (50-73)	0.923	0.07
**CRP (>=8 mg/L)**	5	1593	90 (86-93)	58 (45-70)	0.62	0.113
**CRP (>=5 mg/L)**	5	1593	92 (88-95)	48 (34-62)	0.385	0.319
**CXR (abnormal)**	8	2201	73 (65-80)	58 (48-67)	0.127	0.063
**CXR (suggests tuberculosis)**	8	2134	64 (57-71)	74 (64-82)	0.026	0.003
**Cough (any)**	21	5607	59 (51-66)	66 (58-73)	<0.0001	<0.0001
**Cough (>=2 weeks)**	17	4197	42 (33-51)	78 (70-85)	<0.0001	<0.0001
**Hb (<10 g/dL)**	9	1970	54 (41-65)	74 (64-81)	0.004	0.002
**Hb (<8 g/dL)**	9	1970	14 (9-19)	95 (91-97)	<0.0001	<0.0001
**BMI (<18.5 kg/m²)**	18	4950	35 (27-44)	83 (77-88)	<0.0001	<0.0001
**Lymphadenopathy**	4	1001	33 (15-58)	89 (73-96)	0.004	0.002
**W4SS with CRP (>=10 mg/L)¶**	5	1593	93 (57-99)	23 (9-47)	0.211	0.548
**W4SS with CXR (abnormal)¶**	8	2199	96 (91-98)	14 (7-25)	0.022	0.06
**W4SS then CRP (>=5 mg/L)¶**	5	1593	80 (49-94)	66 (37-86)	0.579	0.066
**W4SS then Xpert*§**	12	3110	69 (61-76)	98 (97-99)	-	-
**Xpert alone*§**	12	3115	77 (68-84)	98 (97-99)	0.167	0.879

†Using culture as a reference standard. Indirect comparisons are based on all studies that evaluated at least one of the W4SS or relevant screening tests

††For Xpert alone, the comparator is W4SS then Xpert

¶For parallel strategies, two screening tests are offered at the same time. For sequential strategies, a second screening test is offered only if the first screening test is positive

*Accuracy measures for entire algorithm using total Xpert (sputum and/or non-sputum Xpert result). Alternative algorithms are W4SS then single sputum Xpert (12 studies; 3110 participants; sensitivity 65 (57-72), specificity 98 (97-99) and single sputum Xpert alone (12 studies; 3115 participants; sensitivity 72 (62-81), specificity 98 (97-99).

§One study assessed Xpert and Xpert Ultra among 191 participants. The accuracy of sputum Xpert was: sensitivity 69 (54-81), specificity 98 (94-99); sputum Xpert Ultra: sensitivity 83 (69-92), specificity 95 (90-97); urine Xpert Ultra: sensitivity 40 (27-56), specificity 95 (90-98); sputum and urine Xpert Ultra: sensitivity 88 (74-95), specificity 91 (86-95)

Definition of abbreviations: BMI = body mass index, CRP = C-reactive protein, CXR = chest X-ray, Hb = haemoglobin, W4SS = WHO four-symptom screen

Table S7D - Indirect comparisons between each test and WHO four-symptom screen for the detection of tuberculosis in participants with CD4 cell count >200 cells/µL†

					Difference from W4SS††	
**Test**	**No studies**	**N**	**Sensitivity (95% CI)**	**Specificity (95% CI)**	**Sensitivity (p-value)**	**Specificity (p-value)**
**W4SS**	21	9598	71 (59-81)	49 (36-63)	-	-
**CRP (>=10 mg/L)**	5	1822	65 (52-76)	79 (68-87)	0.543	0.03
**CRP (>=8 mg/L)**	5	1822	67 (53-79)	76 (64-85)	0.693	0.058
**CRP (>=5 mg/L)**	5	1822	78 (65-87)	65 (54-75)	0.469	0.274
**CXR (abnormal)**	8	3892	66 (57-74)	64 (53-74)	0.777	0.23
**CXR (suggests tuberculosis)**	8	3915	59 (49-69)	81 (70-89)	0.341	0.006
**Cough (any)**	21	9581	51 (41-61)	76 (68-82)	0.019	0.002
**Cough (>=2 weeks)**	17	6409	34 (23-48)	87 (80-91)	<0.0001	<0.0001
**Hb (<10 g/dL)**	9	3047	25 (17-37)	84 (78-89)	<0.0001	0.001
**Hb (<8 g/dL)**	9	3047	6 (3-15)	96 (94-98)	<0.0001	<0.0001
**BMI (<18.5 kg/m²)**	18	7362	20 (13-29)	92 (88-95)	<0.0001	<0.0001
**Lymphadenopathy**	4	1373	30 (10-61)	90 (73-97)	0.017	0.004
**W4SS with CRP (>=10 mg/L)¶**	5	1822	78 (53-92)	35 (15-61)	0.496	0.349
**W4SS with CXR (abnormal)¶**	8	3886	91 (81-96)	28 (16-44)	0.008	0.073
**W4SS then CRP (>=5 mg/L)¶**	5	1822	57 (15-91)	80 (62-91)	0.639	0.03
**W4SS then Xpert*§**	12	5128	46 (39-52)	99 (99-100)	-	-
**Xpert alone*§**	12	5135	57 (47-67)	99 (99-99)	0.052	0.218

†Using culture as a reference standard. Indirect comparisons are based on all studies that evaluated at least one of the W4SS or relevant screening tests

††For Xpert alone, the comparator is W4SS then Xpert

¶For parallel strategies, two screening tests are offered at the same time. For sequential strategies, a second screening test is offered only if the first screening test is positive

*Accuracy measures for entire algorithm using total Xpert (sputum and/or non-sputum Xpert result). Alternative algorithms are W4SS then single sputum Xpert (12 studies; 5127 participants; sensitivity 44 (38-51), specificity 100 (99-100) and single sputum Xpert alone (12 studies; 5134 participants; sensitivity 54 (44-64), specificity 99 (99-99).

§One study assessed Xpert and Xpert Ultra among 472 participants. The accuracy of sputum Xpert was: sensitivity 42 (28-58), specificity 99 (98-100); sputum Xpert Ultra: sensitivity 62 (47-76), specificity 98 (96-99); urine Xpert Ultra: sensitivity 8 (2-21), specificity 99 (97-99); sputum and urine Xpert Ultra: sensitivity 62 (47-76), specificity 97 (95-98)

Definition of abbreviations: BMI = body mass index, CRP = C-reactive protein, CXR = chest X-ray, Hb = haemoglobin, W4SS = WHO four-symptom screen

Table S7E - Indirect comparisons between each test and WHO four-symptom screen for the detection of tuberculosis in pregnant participants†§

					Difference from W4SS††	
**Test**	**No studies**	**N**	**Sensitivity (95% CI)**	**Specificity (95% CI)**	**Sensitivity (p-value)**	**Specificity (p-value)**
**W4SS**	8	1935	84 (24-99)	58 (39-75)	-	-
**CRP (>=10 mg/L)#**	-	-	-	-	-	-
**CRP (>=8 mg/L)#**	-	-	-	-	-	-
**CRP (>=5 mg/L)#**	-	-	-	-	-	-
**CXR (abnormal)**	1	8	75 (11-99)	69 (91-33)	0.538	0.619
**CXR (suggests tuberculosis)**	1	7	75 (11-99)	93 (100-42)	0.456	0.041
**Cough (any)**	8	1933	67 (26-92)	81 (71-88)	0.445	0.038
**Cough (>=2 weeks)**	8	1933	47 (18-78)	92 (86-95)	0.203	0.001
**Hb (<10 g/dL)**	5	1350	20 (10-36)	75 (61-85)	0.132	0.227
**Hb (<8 g/dL)**	5	1350	0 (0-100)	98 (97-99)	<0.0001	<0.0001
**BMI (<18.5 kg/m²)**	7	472	0 (0-100)	96 (94-98)	0.003	<0.0001
**Lymphadenopathy#**	-	-	-	-	-	-
**W4SS with CRP (>=10 mg/L)¶#**	-	-	-	-	-	-
**W4SS with CXR (abnormal)¶**	1	8	75 (11-99)	56 (84-24)	0.541	0.987
**W4SS then CRP (>=5 mg/L)¶#**	-	-	-	-	-	-
**W4SS then Xpert***	5	489	36 (16-62)	100 (0-100)	-	-
**Xpert alone***	5	492	53 (29-76)	99 (98-100)	0.339	0.05

†Using culture as a reference standard. Indirect comparisons are based on all studies that evaluated at least one of the W4SS or relevant screening tests

§For some analyses, all studies had 0% or 100% sensitivity/specificity; therefore, models may have given unreliable estimates such as 95% CIs that range from 0 to 100

††For Xpert alone, the comparator is W4SS then Xpert

¶For parallel strategies, two screening tests are offered at the same time. For sequential strategies, a second screening test is offered only if the first screening test is positive

#Insufficient data to perform meta-analysis

*Accuracy measures for entire algorithm using total Xpert (sputum and/or non-sputum Xpert result). Alternative algorithms are W4SS then single sputum Xpert (5 studies; 489 participants; sensitivity 36 (16-62), specificity 100 (0-100) and single sputum Xpert alone (5 studies; 492 participants; sensitivity 47 (24-71), specificity 99 (98-100).

Definition of abbreviations: BMI = body mass index, CRP = C-reactive protein, CXR = chest X-ray, Hb = haemoglobin, W4SS = WHO four-symptom screen

Table S8 - Direct comparisons between each test and WHO four-symptom screen for the detection of tuberculosis in all participants and subgroups

Table S8A - Direct comparisons between each test and WHO four-symptom screen for the detection of tuberculosis in all participants†

	Index Test				W4SS				Difference from W4SS	
	No of studies	N	Sensitivity (95% CI)	Specificity (95% CI)	No of studies	N	Sensitivity (95% CI)	Specificity (95% CI)	Sensitivity (p-value)	Specificity (p-value)
**CRP (>=10 mg/L)**	5	3571	77 (50-92)	74 (52-88)	5	3571	82 (58-94)	39 (20-63)	0.7	0.052
**CRP (>=8 mg/L)**	5	3571	79 (54-93)	70 (48-86)	5	3571	82 (58-94)	39 (20-63)	0.833	0.079
**CRP (>=5 mg/L)**	5	3571	86 (67-95)	60 (37-79)	5	3571	82 (59-94)	39 (20-62)	0.706	0.231
**CXR (abnormal)**	8	6186	72 (62-80)	62 (46-75)	8	6186	86 (80-91)	32 (20-47)	0.011	0.017
**CXR (suggests tuberculosis)**	8	6141	64 (53-73)	78 (63-88)	8	6141	84 (76-89)	33 (19-51)	0.004	0.001
**Cough (any)**	21	15568	56 (44-66)	72 (62-81)	21	15568	82 (74-88)	42 (31-54)	<0.0001	0.001
**Cough (>=2 weeks)**	17	10906	38 (25-52)	85 (75-91)	17	10906	81 (70-88)	41 (28-56)	<0.0001	<0.0001
**Hb (<10 g/dL)**	9	5115	44 (25-65)	80 (65-89)	9	5115	85 (70-93)	33 (18-51)	0.006	0.001
**Hb (<8 g/dL)**	9	5115	12 (5-25)	96 (91-98)	9	5115	85 (69-93)	33 (18-52)	<0.0001	<0.0001
**BMI (<18.5 kg/m²)**	18	12639	29 (20-40)	89 (82-93)	18	12639	84 (76-90)	41 (29-54)	<0.0001	<0.0001
**Lymphadenopathy**	4	2389	31 (16-52)	90 (77-96)	4	2389	91 (79-96)	27 (12-49)	0.001	0.001
**W4SS with CRP (>=10 mg/L)¶**	5	3571	89 (63-97)	31 (12-58)	5	3571	82 (50-95)	39 (17-67)	0.617	0.65
**W4SS with CXR (abnormal)¶**	8	6186	94 (89-97)	20 (10-37)	8	6186	87 (78-92)	32 (17-52)	0.05	0.32
**W4SS then CRP (>=5 mg/L)¶**	5	3571	72 (36-92)	75 (50-90)	5	3571	81 (49-95)	39 (18-66)	0.62	0.074

†Using culture as a reference standard. Direct comparisons are based on all studies that evaluated both the W4SS and relevant screening test

¶For parallel strategies, two screening tests are offered at the same time. For sequential strategies, a second screening test is offered only if the first screening test is positive

Definition of abbreviations: BMI = body mass index, CRP = C-reactive protein, CXR = chest X-ray, Hb = haemoglobin, W4SS = WHO four-symptom screen

Table S8B - Direct comparisons between each test and WHO four-symptom screen for the detection of tuberculosis in outpatients (on ART)†

	Index Test				W4SS				Difference from W4SS	
	**No of studies**	**N**	**Sensitivity (95% CI)**	**Specificity (95% CI)**	**No of studies**	**N**	**Sensitivity (95% CI)**	**Specificity (95% CI)**	**Sensitivity (p-value)**	**Specificity (p-value)**
**CRP (>=10 mg/L)**	1	381	20 (3-69)	90 (87-93)	1	381	8 (1-62)	88 (84-91)	0.458	0.246
**CRP (>=8 mg/L)**	1	381	40 (10-80)	89 (86-92)	1	381	8 (1-62)	88 (84-91)	0.223	0.571
**CRP (>=5 mg/L)**	1	381	40 (10-80)	80 (75-84)	1	381	8 (1-62)	88 (84-91)	0.223	0.003
**CXR (abnormal)**	4	2670	74 (55-87)	63 (42-80)	4	2670	70 (50-85)	52 (31-72)	0.786	0.469
**CXR (suggests tuberculosis)**	4	2581	71 (52-84)	78 (57-91)	4	2581	64 (44-80)	54 (31-76)	0.587	0.144
**Cough (any)**	9	4309	40 (23-58)	83 (70-91)	9	4309	54 (36-72)	71 (54-84)	0.286	0.206
**Cough (>=2 weeks)**	6	1746	20 (6-50)	93 (79-98)	6	1746	52 (23-80)	74 (45-91)	0.136	0.09
**Hb (<10 g/dL)**	4	844	52 (15-87)	84 (60-94)	4	844	65 (21-93)	54 (26-79)	0.689	0.107
**Hb (<8 g/dL)**	4	844	15 (1-78)	95 (84-98)	4	844	67 (9-98)	54 (27-78)	0.285	0.008
**BMI (<18.5 kg/m²)**	7	4036	16 (8-32)	93 (85-97)	7	4036	53 (33-72)	73 (54-86)	0.009	0.017
**Lymphadenopathy**	1	338	22 (6-58)	93 (89-95)	1	338	78 (42-94)	55 (50-60)	0.027	<0.0001
**W4SS with CRP (>=10 mg/L)¶**	1	381	20 (3-69)	80 (75-83)	1	381	8 (1-62)	88 (84-91)	0.458	0.003
**W4SS with CXR (abnormal)¶**	4	2670	89 (71-96)	33 (15-57)	4	2670	73 (47-89)	52 (28-75)	0.187	0.291
**W4SS then CRP (>=5 mg/L)¶**	1	381	8 (1-62)	96 (93-97)	1	381	8 (1-62)	88 (84-91)	1	<0.0001

†Using culture as a reference standard. Direct comparisons are based on all studies that evaluated both the W4SS and relevant screening test

¶For parallel strategies, two screening tests are offered at the same time. For sequential strategies, a second screening test is offered only if the first screening test is positive

Definition of abbreviations: BMI = body mass index, CRP = C-reactive protein, CXR = chest X-ray, Hb = haemoglobin, W4SS = WHO four-symptom screen

Table S8C - Direct comparisons between each test and WHO four-symptom screen for the detection of tuberculosis in outpatients (not on ART)†

	Index Test				W4SS				Difference from W4SS	
	**No of studies**	**N**	**Sensitivity (95% CI)**	**Specificity (95% CI)**	**No of studies**	**N**	**Sensitivity (95% CI)**	**Specificity (95% CI)**	**Sensitivity (p-value)**	**Specificity (p-value)**
**CRP (>=10 mg/L)**	5	3187	84 (74-90)	66 (53-78)	5	3187	90 (83-94)	27 (17-40)	0.236	0.005
**CRP (>=8 mg/L)**	5	3187	85 (75-92)	63 (49-75)	5	3187	90 (82-95)	27 (17-40)	0.349	0.008
**CRP (>=5 mg/L)**	5	3187	90 (83-95)	53 (39-66)	5	3187	90 (83-94)	27 (18-40)	0.952	0.031
**CXR (abnormal)**	8	3516	71 (62-79)	62 (48-74)	8	3516	89 (83-93)	27 (17-40)	0.001	0.002
**CXR (suggests tuberculosis)**	8	3560	62 (52-71)	79 (65-88)	8	3560	87 (81-92)	28 (17-44)	<0.0001	<0.0001
**Cough (any)**	20	11131	58 (47-68)	70 (60-78)	20	11131	85 (78-90)	37 (27-48)	<0.0001	<0.0001
**Cough (>=2 weeks)**	16	9032	42 (29-55)	82 (72-89)	16	9032	83 (74-90)	37 (25-50)	<0.0001	<0.0001
**Hb (<10 g/dL)**	9	4268	44 (27-63)	79 (65-88)	9	4268	89 (78-95)	26 (15-41)	0.001	<0.0001
**Hb (<8 g/dL)**	9	4268	11 (5-21)	96 (92-98)	9	4268	88 (78-94)	26 (15-42)	<0.0001	<0.0001
**BMI (<18.5 kg/m²)**	17	8475	31 (22-42)	87 (81-92)	17	8475	87 (81-92)	34 (24-46)	<0.0001	<0.0001
**Lymphadenopathy**	4	2051	30 (15-51)	91 (78-96)	4	2051	91 (80-96)	25 (11-48)	0.001	0.001
**W4SS with CRP (>=10 mg/L)¶**	5	3187	94 (87-97)	20 (11-34)	5	3187	90 (79-95)	28 (16-43)	0.351	0.407
**W4SS with CXR (abnormal)¶**	8	3516	95 (91-97)	18 (9-32)	8	3516	89 (82-93)	27 (15-44)	0.045	0.364
**W4SS then CRP (>=5 mg/L)¶**	5	3187	83 (72-91)	65 (51-76)	5	3187	90 (82-95)	27 (18-40)	0.254	0.004

†Using culture as a reference standard. Direct comparisons are based on all studies that evaluated both the W4SS and relevant screening test

¶For parallel strategies, two screening tests are offered at the same time. For sequential strategies, a second screening test is offered only if the first screening test is positive

Definition of abbreviations: BMI = body mass index, CRP = C-reactive protein, CXR = chest X-ray, Hb = haemoglobin, W4SS = WHO four-symptom screen

Table S8D - Direct comparisons between each test and WHO four-symptom screen for the detection of tuberculosis in participants with CD4 cell count <=200 cells/μL†

	Index Test				W4SS				Difference from W4SS	
	**No of studies**	**N**	**Sensitivity (95% CI)**	**Specificity (95% CI)**	**No of studies**	**N**	**Sensitivity (95% CI)**	**Specificity (95% CI)**	**Sensitivity (p-value)**	**Specificity (p-value)**
**CRP (>=10 mg/L)**	5	1593	87 (59-97)	64 (39-83)	5	1593	86 (58-97)	32 (15-56)	0.992	0.094
**CRP (>=8 mg/L)**	5	1593	89 (69-97)	60 (35-80)	5	1593	88 (64-96)	32 (15-57)	0.851	0.145
**CRP (>=5 mg/L)**	5	1593	92 (74-98)	49 (26-73)	5	1593	87 (63-97)	32 (14-57)	0.655	0.351
**CXR (abnormal)**	8	2199	74 (64-81)	58 (44-70)	8	2199	90 (84-94)	22 (14-32)	0.002	0.001
**CXR (suggests tuberculosis)**	8	2132	65 (55-73)	74 (60-84)	8	2132	88 (82-92)	24 (14-37)	<0.0001	<0.0001
**Cough (any)**	21	5607	57 (45-69)	67 (55-77)	21	5607	87 (79-92)	32 (22-44)	<0.0001	<0.0001
**Cough (>=2 weeks)**	17	4197	39 (25-55)	80 (67-88)	17	4197	85 (75-92)	31 (19-47)	<0.0001	<0.0001
**Hb (<10 g/dL)**	9	1969	54 (33-73)	74 (56-86)	9	1969	88 (75-95)	26 (13-43)	0.013	0.002
**Hb (<8 g/dL)**	9	1969	15 (7-30)	95 (89-98)	9	1969	89 (76-95)	25 (13-44)	<0.0001	<0.0001
**BMI (<18.5 kg/m²)**	18	4947	34 (23-47)	84 (75-90)	18	4947	88 (80-93)	31 (21-44)	<0.0001	<0.0001
**Lymphadenopathy**	4	1000	33 (16-56)	89 (75-95)	4	1000	94 (84-98)	18 (8-37)	0.001	0.001
**W4SS with CRP (>=10 mg/L)¶**	5	1593	94 (62-99)	23 (8-50)	5	1593	83 (36-98)	32 (12-61)	0.489	0.614
**W4SS with CXR (abnormal)¶**	8	2199	96 (92-98)	14 (7-24)	8	2199	90 (83-94)	22 (12-36)	0.042	0.267
**W4SS then CRP (>=5 mg/L)¶**	5	1593	77 (35-95)	66 (36-87)	5	1593	86 (50-98)	32 (12-63)	0.631	0.144

†Using culture as a reference standard. Direct comparisons are based on all studies that evaluated both the W4SS and relevant screening test

¶For parallel strategies, two screening tests are offered at the same time. For sequential strategies, a second screening test is offered only if the first screening test is positive

Definition of abbreviations: BMI = body mass index, CRP = C-reactive protein, CXR = chest X-ray, Hb = haemoglobin, W4SS = WHO four-symptom screen

Table S8E - Direct comparisons between each test and WHO four-symptom screen for the detection of tuberculosis in participants with CD4 cell count >200 cells/µL†

	Index Test				W4SS				Difference from W4SS	
	**No of studies**	**N**	**Sensitivity (95% CI)**	**Specificity (95% CI)**	**No of studies**	**N**	**Sensitivity (95% CI)**	**Specificity (95% CI)**	**Sensitivity (p-value)**	**Specificity (p-value)**
**CRP (>=10 mg/L)**	5	1822	62 (35-83)	79 (60-91)	5	1822	72 (45-89)	43 (23-66)	0.581	0.035
**CRP (>=8 mg/L)**	5	1822	65 (37-86)	76 (55-89)	5	1822	72 (44-90)	43 (23-66)	0.694	0.055
**CRP (>=5 mg/L)**	5	1822	78 (53-92)	65 (43-82)	5	1822	70 (43-88)	43 (23-65)	0.626	0.182
**CXR (abnormal)**	8	3886	67 (54-78)	64 (49-77)	8	3886	79 (68-87)	41 (27-57)	0.111	0.048
**CXR (suggests tuberculosis)**	8	3909	60 (47-72)	81 (68-90)	8	3909	77 (65-86)	41 (25-59)	0.058	0.002
**Cough (any)**	21	9581	52 (40-63)	75 (66-83)	21	9581	71 (60-80)	50 (38-61)	0.018	0.002
**Cough (>=2 weeks)**	17	6409	34 (22-49)	87 (78-92)	17	6409	70 (56-81)	48 (34-62)	0.001	<0.0001
**Hb (<10 g/dL)**	9	3047	26 (12-48)	84 (72-91)	9	3047	79 (58-91)	41 (25-58)	0.004	0.001
**Hb (<8 g/dL)**	9	3047	6 (2-18)	97 (93-98)	9	3047	79 (56-92)	41 (24-59)	<0.0001	<0.0001
**BMI (<18.5 kg/m²)**	18	7356	20 (13-30)	92 (87-95)	18	7356	74 (64-83)	49 (36-62)	<0.0001	<0.0001
**Lymphadenopathy**	4	1373	30 (12-56)	90 (77-96)	4	1373	85 (65-95)	34 (16-58)	0.008	0.003
**W4SS with CRP (>=10 mg/L)¶**	5	1822	77 (48-93)	35 (15-62)	5	1822	71 (39-90)	43 (20-70)	0.707	0.665
**W4SS with CXR (abnormal)¶**	8	3886	91 (81-96)	28 (16-45)	8	3886	82 (67-91)	41 (25-59)	0.159	0.293
**W4SS then CRP (>=5 mg/L)¶**	5	1822	61 (21-90)	80 (58-92)	5	1822	71 (29-93)	43 (21-69)	0.731	0.049

†Using culture as a reference standard. Direct comparisons are based on all studies that evaluated both the W4SS and relevant screening test

¶For parallel strategies, two screening tests are offered at the same time. For sequential strategies, a second screening test is offered only if the first screening test is positive

Definition of abbreviations: BMI = body mass index, CRP = C-reactive protein, CXR = chest X-ray, Hb = haemoglobin, W4SS = WHO four-symptom screen

Table S8F - Direct comparisons between each test and WHO four-symptom screen for the detection of tuberculosis in pregnant participants†§

	Index Test				W4SS				Difference from W4SS	
	No of studies	N	Sensitivity (95% CI)	Specificity (95% CI)	No of studies	N	Sensitivity (95% CI)	Specificity (95% CI)	Sensitivity (p-value)	Specificity (p-value)
**CRP (>=10 mg/L)#**	-	-	-	-	-	-	-	-	-	-
**CRP (>=8 mg/L)#**	-	-	-	-	-	-	-	-	-	-
**CRP (>=5 mg/L)#**	-	-	-	-	-	-	-	-	-	-
**CXR (abnormal)**	1	8	75 (11-99)	69 (33-91)	1	8	75 (11-99)	81 (42-96)	1	0.567
**CXR (suggests tuberculosis)**	1	7	75 (11-99)	93 (42-100)	1	7	75 (11-99)	79 (38-96)	1	0.465
**Cough (any)**	8	1933	69 (28-93)	80 (68-89)	8	1933	83 (39-97)	59 (42-74)	0.448	0.04
**Cough (>=2 weeks)**	8	1933	53 (20-83)	92 (84-96)	8	1933	78 (36-95)	59 (43-74)	0.215	0.001
**Hb (<10 g/dL)**	5	1350	53 (6-95)	74 (58-86)	5	1350	92 (7-100)	59 (41-75)	0.125	0.184
**Hb (<8 g/dL)**	5	1350	0 (0-100)	98 (94-99)	5	1350	95 (1-100)	59 (40-75)	0.001	<0.0001
**BMI (<18.5 kg/m²)**	7	471	0 (0-98)	97 (93-99)	7	471	100 (0-100)	54 (38-69)	<0.0001	<0.0001
**Lymphadenopathy#**	-	-	-	-	-	-	-	-	-	-
**W4SS with CRP (>=10 mg/L)¶#**	-	-	-	-	-	-	-	-	-	-
**W4SS with CXR (abnormal)¶**	1	8	75 (11-99)	56 (24-84)	1	8	75 (11-99)	81 (42-96)	1	0.292
**W4SS then CRP (>=5 mg/L)¶#**	-	-	-	-	-	-	-	-	-	-

†Using culture as a reference standard. Direct comparisons are based on all studies that evaluated both the W4SS and relevant screening test

§For some analyses, all studies had 0% or 100% sensitivity/specificity; therefore, models may have given unreliable estimates such as 95% CIs that range from 0 to 100

¶For parallel strategies, two screening tests are offered at the same time. For sequential strategies, a second screening test is offered only if the first screening test is positive

#Insufficient data to perform meta-analysis

Definition of abbreviations: BMI = body mass index, CRP = C-reactive protein, CXR = chest X-ray, Hb = haemoglobin, W4SS = WHO four-symptom screen

Table S9 - Additional diagnostic accuracy estimates

Table S9A - Additional diagnostic accuracy estimates in all participants

									Metaregression†	
	Univariate diagnostic odds ratio				Positive screen (%)††		Trivariate GLMM†††		Reference standard	Prevalence
Test	Estimate (95% CI)	I² (95% CI)	Trim-and-fill estimate (95% CI)	Egger's test (p-value)	Estimate (95% CI)	I² (95% CI)	PPV (95% CI)	NPV (95% CI)	P-value	P-value
**W4SS**	3.21 (2.61-3.95)	27 (0-57)	3.39 (2.74-4.20)	0.16	60 (45-73)	100 (99-100)	11.6 (9.3-14.3)	96.2 (94.9-97.2)	0.63	0.19
**CRP (>=10 mg/L)**	9.06 (5.55-14.78)	63 (3-86)	13.83 (8.18-23.36)	0.21	32 (20-47)	97 (95-98)	20.7 (10.4-37.1)	96.8 (94.7-98.1)	-	0.23
**CRP (>=8 mg/L)**	8.93 (5.11-15.63)	70 (25-88)	14.15 (7.88-25.38)	0.38	35 (22-50)	97 (96-98)	20.5 (11.5-33.9)	97.1 (94.9-98.3)	-	0.35
**CRP (>=5 mg/L)**	8.79 (5.06-15.26)	60 (0-85)	12.33 (6.80-22.33)	0.3	45 (32-59)	98 (96-98)	16.7 (8.4-30.6)	97.5 (95.6-98.6)	-	0.28
**CXR (abnormal)**	4.07 (2.97-5.58)	45 (0-76)	4.07 (2.97-5.58)	0.84	41 (32-52)	99 (98-99)	14.0 (9.5-20.2)	96.1 (93.4-97.7)	0.08	0.27
**CXR (suggests tuberculosis)**	6.37 (3.89-10.43)	81 (63-90)	6.37 (3.92-10.35)	0.95	26 (17-38)	99 (99-99)	22.6 (17.6-28.5)	95.4 (93.1-96.9)	0.12	0.11
**Cough (any)**	3.23 (2.76-3.77)	28 (0-58)	3.23 (2.76-3.77)	0.98	30 (23-38)	99 (98-99)	15.1 (12.4-18.2)	95.3 (93.5-96.5)	0.84	<0.0001
**Cough (>=2 weeks)**	3.32 (2.62-4.20)	55 (22-74)	2.95 (2.31-3.76)	0.41	18 (12-25)	98 (97-98)	19.2 (15.9-22.9)	94.1 (91.7-95.8)	0.4	<0.0001
**Hb (<10 g/dL)**	3.03 (2.31-3.97)	22 (0-63)	2.96 (2.25-3.89)	0.96	22 (16-30)	97 (96-98)	12.5 (6.6-22.4)	95.2 (91.3-97.4)	0.4	0.93
**Hb (<8 g/dL)**	3.25 (1.73-6.09)	60 (18-81)	4.33 (2.22-8.44)	0.54	5 (3-8)	94 (90-96)	15.5 (7.2-30.3)	93.9 (89.1-96.7)	0.12	0.63
**BMI (<18.5 kg/m²)**	3.11 (2.65-3.64)	9 (0-45)	3.45 (2.83-4.20)	0.72	13 (9-18)	98 (97-98)	18.3 (13.4-24.5)	93.6 (91.1-95.5)	0.65	0.26
**Lymphadenopathy**	3.96 (2.72-5.77)	0 (0-83)	4.81 (3.24-7.15)	0.93	12 (5-26)	99 (98-99)	18.3 (8.1-36.3)	94.5 (89.0-97.3)	0.21	0.32
**W4SS with CRP (>=10 mg/L)**	3.61 (2.41-5.40)	0 (0-71)	3.61 (2.41-5.40)	0.9	71 (45-88)	99 (99-100)	12.6 (8.1-19.0)	96.5 (93.7-98.1)	-	0.28
**W4SS with CXR (abnormal)**	5.22 (3.61-7.54)	0 (0-62)	5.44 (3.79-7.83)	0.38	81 (65-91)	99 (99-99)	10.0 (6.9-14.4)	97.8 (94.6-99.2)	0.91	0.04
**W4SS then CRP (>=5 mg/L)**	8.49 (4.69-15.37)	72 (30-89)	8.95 (5.04-15.90)	0.66	30 (13-54)	98 (96-99)	21.9 (13.9-33.0)	96.7 (94.6-98.0)	-	0.61
**W4SS then Xpert***	142.47 (95.22-213.16)	32 (0-65)	142.49 (95.27-213.13)	0.37	5 (3-9)	93 (90-95)	87.9 (75.4-94.5)	96.6 (94.6-97.9)	0.66	0.54
**Xpert alone***	165.89 (95.97-286.77)	62 (29-80)	273.62 (157.19-476.31)	0.56	7 (4-10)	92 (88-95)	81.1 (66.8-90.1)	97.5 (95.5-98.6)	0.65	0.78

†For the meta-regressions, the outcome variable was the diagnostic odds ratio

††Calculated using meta-analysis of proportions. We used a generalized linear mixed model with logit transformation in preference to protocol specified DerSimonian and Laird random effects model for proportions with variance stabilization by applying the Freeman-Tukey double arcsine transformation

†††Pooled using a trivariate generalized linear mixed model that jointly models diagnostic test prevalence and predictive values

*Estimates for entire algorithm using total Xpert (sputum and/or non-sputum Xpert result)

Definition of abbreviations: BMI = body mass index, CRP = C-reactive protein, CXR = chest X-ray, GLMM = generalized linear mixed model, Hb = haemoglobin, NPV = negative predictive value, PPV = positive predictive value, W4SS = WHO four-symptom screen

Table S9B - Additional diagnostic accuracy estimates in outpatients (On ART)

									Metaregression†	
	Univariate diagnostic odds ratio				Positive screen (%)††		Trivariate GLMM†††		Reference standard	Prevalence
**Test**	**Estimate (95% CI)**	**I² (95% CI)**	**Trim-and-fill estimate (95% CI)**	**Egger's test (p-value)**	**Estimate (95% CI)**	**I² (95% CI)**	**PPV (95% CI)**	**NPV (95% CI)**	**P-value**	**P-value**
**W4SS**	3.07 (2.09-4.52)	0 (0-63)	3.80 (2.50-5.79)	0.38	30 (16-50)	99 (98-99)	6.0 (3.9-9.1)	97.9 (96.7-98.7)	0.93	0.41
**CRP (>=10 mg/L)**	2.36 (0.26-21.70)	-	-	-	10 (7-13)	-	2.7 (0.1-13.8)	98.8 (97.0-99.5)	-	-
**CRP (>=8 mg/L)**	5.45 (0.88-33.56)	-	-	-	11 (8-15)	-	4.7 (1.3-15.5)	99.1 (97.4-99.7)	-	-
**CRP (>=5 mg/L)**	2.63 (0.43-16.03)	-	-	-	20 (17-25)	-	2.6 (0.7-8.9)	99.0 (97.1-99.7)	-	-
**CXR (abnormal)**	4.31 (2.21-8.39)	22 (0-88)	4.31 (2.22-8.35)	0.64	38 (26-52)	98 (97-99)	5.9 (4.3-8.0)	98.7 (97.4-99.3)	0.15	0.44
**CXR (suggests tuberculosis)**	8.84 (4.39-17.80)	37 (0-78)	8.84 (4.44-17.58)	0.8	24 (12-41)	99 (98-99)	11.3 (8.7-14.5)	98.5 (97.3-99.2)	0.16	0.12
**Cough (any)**	3.16 (2.11-4.74)	0 (0-65)	3.16 (2.11-4.74)	0.74	18 (11-28)	97 (96-98)	7.1 (4.9-10.1)	97.8 (96.4-98.6)	0.21	0.21
**Cough (>=2 weeks)**	3.23 (1.53-6.82)	0 (0-72)	2.27 (1.16-4.46)	0.12	7 (2-22)	98 (96-98)	9.6 (5.9-15.2)	97.4 (95.0-98.6)	0.19	0.2
**Hb (<10 g/dL)**	5.42 (2.19-13.45)	0 (0-87)	5.42 (2.19-13.45)	0.76	19 (10-31)	94 (88-97)	7.2 (3.4-14.7)	98.7 (97.3-99.3)	0.59	0.81
**Hb (<8 g/dL)**	5.23 (1.62-16.91)	0 (0-83)	8.22 (3.08-21.89)	0.15	6 (4-9)	54 (0-85)	6.7 (1.7-22.7)	98.0 (96.3-98.9)	0.96	0.46
**BMI (<18.5 kg/m²)**	2.61 (1.56-4.37)	0 (0-0)	2.61 (1.56-4.37)	0.44	7 (4-13)	95 (91-97)	6.6 (3.5-12.1)	97.4 (96.1-98.2)	0.35	0.65
**Lymphadenopathy**	3.63 (0.71-18.45)	-	-	-	8 (5-11)	-	7.7 (2.1-24.1)	97.8 (95.4-98.9)	-	-
**W4SS with CRP (>=10 mg/L)**	0.97 (0.11-8.81)	-	-	-	20 (17-25)	-	1.3 (0.1-6.9)	98.7 (96.7-99.5)	-	-
**W4SS with CXR (abnormal)**	3.69 (1.96-6.93)	0 (0-61)	3.69 (1.96-6.93)	0.96	68 (46-84)	99 (99-99)	4.7 (3.4-6.4)	98.8 (96.9-99.5)	0.79	0.41
**W4SS then CRP (>=5 mg/L)**	2.12 (0.11-40.08)	-	-	-	4 (2-6)	-	0.0 (0.0-20.4)	98.6 (96.8-99.4)	-	-
**W4SS then Xpert***	82.01 (14.71-457.12)	65 (0-88)	230.45 (35.98-1476.17)	0.82	1 (0-4)	88 (71-95)	78.3 (22.7-97.8)	98.9 (98.4-99.2)	-	0.34
**Xpert alone***	88.54 (10.76-728.44)	81 (51-93)	442.60 (45.58-4298.07)	0.46	2 (1-4)	82 (54-93)	42.7 (20.7-68.2)	99.2 (98.2-99.7)	-	0.85

†For the meta-regressions, the outcome variable was the diagnostic odds ratio

††Calculated using meta-analysis of proportions. We used a generalized linear mixed model with logit transformation in preference to protocol specified DerSimonian and Laird random effects model for proportions with variance stabilization by applying the Freeman-Tukey double arcsine transformation

†††Pooled using a trivariate generalized linear mixed model that jointly models diagnostic test prevalence and predictive values

*Estimates for entire algorithm using total Xpert (sputum and/or non-sputum Xpert result)

Definition of abbreviations: BMI = body mass index, CRP = C-reactive protein, CXR = chest X-ray, GLMM = generalized linear mixed model, Hb = haemoglobin, NPV = negative predictive value, PPV = positive predictive value, W4SS = WHO four-symptom screen

Table S9C - Additional diagnostic accuracy estimates in outpatients (Not on ART)

									Metaregression†	
	Univariate diagnostic odds ratio				Positive screen (%)††		Trivariate GLMM†††		Reference standard	Prevalence
Test	Estimate (95% CI)	I² (95% CI)	Trim-and-fill estimate (95% CI)	Egger's test (p-value)	Estimate (95% CI)	I² (95% CI)	PPV (95% CI)	NPV (95% CI)	P-value	P-value
**W4SS**	3.14 (2.61-3.78)	0 (0-46)	3.11 (2.59-3.75)	0.72	65 (52-76)	99 (99-99)	12.8 (10.5-15.6)	95.8 (94.4-96.8)	0.63	0.78
**CRP (>=10 mg/L)**	9.66 (5.97-15.65)	67 (3-89)	13.79 (8.17-23.28)	0.35	40 (33-46)	91 (82-96)	28.4 (22.9-34.6)	95.9 (94.6-96.9)	-	0.66
**CRP (>=8 mg/L)**	9.30 (5.10-16.95)	77 (37-92)	14.05 (7.45-26.52)	0.39	43 (36-50)	92 (84-96)	26.6 (21.1-32.9)	96.1 (94.8-97.1)	-	0.47
**CRP (>=5 mg/L)**	9.72 (5.65-16.70)	62 (0-87)	14.97 (8.02-27.93)	0.61	52 (46-59)	92 (86-96)	23.3 (18.8-28.4)	96.8 (95.3-97.8)	-	0.95
**CXR (abnormal)**	4.08 (3.02-5.51)	28 (0-68)	4.08 (3.02-5.50)	0.93	42 (31-52)	98 (97-98)	19.0 (15.3-23.2)	95.0 (91.8-96.9)	0.44	0.01
**CXR (suggests tuberculosis)**	5.98 (3.42-10.46)	81 (64-90)	5.49 (3.23-9.32)	0.99	26 (17-38)	98 (97-99)	28.3 (24.6-32.4)	93.7 (91.2-95.6)	0.29	<0.0001
**Cough (any)**	3.12 (2.74-3.56)	0 (0-41)	2.95 (2.57-3.39)	0.29	33 (27-40)	98 (97-98)	16.9 (14.0-20.1)	94.4 (92.5-95.9)	0.46	0.06
**Cough (>=2 weeks)**	3.26 (2.61-4.06)	48 (5-71)	2.92 (2.34-3.65)	0.17	21 (15-28)	97 (96-98)	20.6 (17.6-23.9)	93.3 (90.9-95.1)	0.72	0.01
**Hb (<10 g/dL)**	2.88 (2.19-3.80)	24 (0-66)	2.88 (2.19-3.80)	0.41	24 (17-32)	96 (95-98)	13.3 (6.2-26.5)	94.2 (89.5-96.9)	0.55	0.44
**Hb (<8 g/dL)**	3.11 (1.48-6.52)	69 (31-86)	3.11 (1.50-6.43)	0.74	5 (3-8)	93 (90-96)	16.6 (6.3-37.0)	92.6 (86.9-95.9)	0.08	0.91
**BMI (<18.5 kg/m²)**	3.05 (2.54-3.66)	17 (0-53)	2.86 (2.37-3.46)	0.7	15 (10-21)	97 (97-98)	21.8 (16.7-28.0)	92.1 (89.5-94.1)	0.59	0.34
**Lymphadenopathy**	4.01 (2.73-5.89)	0 (0-83)	3.43 (2.37-4.98)	0.85	11 (4-26)	98 (97-99)	19.5 (7.1-43.6)	94.8 (87.7-97.9)	0.22	0.35
**W4SS with CRP (>=10 mg/L)**	3.78 (2.51-5.70)	0 (0-67)	3.44 (2.36-5.00)	0.17	82 (70-90)	98 (97-99)	16.3 (14.9-17.7)	95.5 (93.3-97.0)	-	0.79
**W4SS with CXR (abnormal)**	4.85 (3.07-7.67)	0 (0-66)	5.73 (3.69-8.90)	0.22	83 (69-92)	98 (97-98)	12.5 (9.1-16.8)	97.2 (92.8-99.0)	0.82	0.1
**W4SS then CRP (>=5 mg/L)**	8.94 (4.86-16.45)	78 (39-92)	8.94 (4.88-16.38)	0.9	42 (35-50)	93 (86-96)	27.0 (23.3-31.0)	96.0 (94.3-97.2)	-	0.88
**W4SS then Xpert***	154.61 (111.63-214.14)	0 (0-44)	154.61 (111.63-214.14)	0.21	8 (6-10)	82 (68-89)	83.7 (71.7-91.2)	96.6 (94.2-98.0)	0.32	0.85
**Xpert alone***	184.61 (120.87-281.97)	30 (0-66)	184.60 (120.90-281.86)	0.99	9 (7-11)	74 (53-86)	84.0 (74.7-90.3)	97.7 (95.1-99.0)	0.29	0.36

†For the meta-regressions, the outcome variable was the diagnostic odds ratio

††Calculated using meta-analysis of proportions. We used a generalized linear mixed model with logit transformation in preference to protocol specified DerSimonian and Laird random effects model for proportions with variance stabilization by applying the Freeman-Tukey double arcsine transformation

†††Pooled using a trivariate generalized linear mixed model that jointly models diagnostic test prevalence and predictive values

*Estimates for entire algorithm using total Xpert (sputum and/or non-sputum Xpert result)

Definition of abbreviations: BMI = body mass index, CRP = C-reactive protein, CXR = chest X-ray, GLMM = generalized linear mixed model, Hb = haemoglobin, NPV = negative predictive value, PPV = positive predictive value, W4SS = WHO four-symptom screen

Table S9D - Additional diagnostic accuracy estimates in participants with a CD4 cell count <=200 cells/µL

									Metaregression†	
	Univariate diagnostic odds ratio				Positive screen (%)††		Trivariate GLMM†††		Reference standard	Prevalence
**Test**	**Estimate (95% CI)**	**I² (95% CI)**	**Trim-and-fill estimate (95% CI)**	**Egger's test (p-value)**	**Estimate (95% CI)**	**I² (95% CI)**	**PPV (95% CI)**	**NPV (95% CI)**	**P-value**	**P-value**
**W4SS**	2.98 (2.33-3.81)	0 (0-42)	3.28 (2.54-4.24)	0.29	70 (55-82)	98 (98-99)	17.5 (14.7-20.7)	94.0 (92.0-95.5)	0.29	0.46
**CRP (>=10 mg/L)**	12.06 (7.99-18.21)	9 (0-81)	14.72 (9.32-23.24)	0.02	46 (32-60)	91 (82-96)	37.5 (31.7-43.7)	95.4 (93.7-96.6)	-	0.1
**CRP (>=8 mg/L)**	13.34 (8.96-19.87)	1 (0-80)	15.50 (10.22-23.51)	0.04	50 (35-64)	92 (84-96)	36.0 (29.8-42.8)	96.0 (94.0-97.3)	-	0.59
**CRP (>=5 mg/L)**	9.69 (5.15-18.26)	35 (0-76)	12.41 (6.74-22.87)	0.26	58 (43-72)	94 (88-97)	31.7 (26.4-37.5)	96.1 (93.6-97.6)	-	0.55
**CXR (abnormal)**	3.78 (2.83-5.05)	0 (0-66)	4.12 (3.08-5.50)	0.44	46 (37-56)	96 (94-98)	20.4 (15.7-26.2)	93.7 (90.6-95.9)	0.23	0.4
**CXR (suggests tuberculosis)**	4.95 (3.15-7.78)	61 (16-82)	5.52 (3.53-8.64)	0.7	32 (23-42)	96 (95-98)	29.7 (25.8-33.9)	92.3 (89.7-94.3)	0.24	0.1
**Cough (any)**	2.63 (2.24-3.09)	0 (0-40)	2.65 (2.26-3.11)	0.98	37 (30-45)	96 (95-97)	21.8 (18.6-25.3)	91.4 (88.9-93.5)	0.77	0.01
**Cough (>=2 weeks)**	2.46 (1.93-3.13)	34 (0-64)	2.29 (1.78-2.96)	0.41	25 (18-33)	94 (92-96)	27.1 (23.1-31.4)	89.0 (85.9-91.4)	0.1	<0.0001
**Hb (<10 g/dL)**	3.25 (2.21-4.79)	37 (0-71)	3.13 (2.09-4.69)	0.71	30 (22-39)	94 (90-96)	20.4 (13.1-30.3)	92.3 (88.1-95.0)	0.97	0.64
**Hb (<8 g/dL)**	3.49 (1.55-7.87)	64 (27-82)	4.65 (1.93-11.16)	0.7	7 (5-10)	83 (70-91)	26.6 (14.0-44.7)	89.9 (85.1-93.3)	0.3	0.9
**BMI (<18.5 kg/m²)**	2.57 (2.15-3.09)	0 (0-43)	2.41 (2.03-2.87)	0.61	19 (14-26)	96 (95-97)	24.6 (19.1-31.0)	89.1 (86.3-91.4)	0.99	0.91
**Lymphadenopathy#**	3.97 (2.36-6.68)	0 (0-80)	3.81 (2.31-6.28)	0.59	14 (6-30)	97 (95-98)	-	-	0.32	0.52
**W4SS with CRP (>=10 mg/L)**	5.14 (2.11-12.54)	14 (0-82)	5.15 (2.22-11.96)	0.63	80 (56-92)	96 (93-98)	21.3 (17.0-26.4)	95.8 (91.6-98.0)	-	0.15
**W4SS with CXR (abnormal)**	3.90 (2.25-6.79)	0 (0-36)	5.11 (3.14-8.31)	0.33	88 (77-94)	97 (96-98)	14.4 (11.0-18.7)	96.7 (90.5-98.9)	0.82	0.29
**W4SS then CRP (>=5 mg/L)**	7.50 (3.88-14.48)	57 (0-84)	12.80 (6.37-25.74)	0.39	41 (17-70)	85 (67-93)	32.5 (27.0-38.6)	94.7 (91.5-96.8)	-	0.88
**W4SS then Xpert***	127.84 (86.56-188.80)	0 (0-52)	142.03 (90.33-223.30)	0.11	12 (9-14)	68 (42-82)	85.4 (73.1-92.6)	95.4 (92.6-97.1)	0.47	0.29
**Xpert alone***	167.10 (100.59-277.58)	25 (0-63)	218.91 (123.79-387.11)	0.41	13 (10-16)	63 (31-80)	86.0 (74.3-92.9)	96.6 (94.0-98.1)	0.73	0.63

†For the meta-regressions, the outcome variable was the diagnostic odds ratio

††Calculated using meta-analysis of proportions. We used a generalized linear mixed model with logit transformation in preference to protocol specified DerSimonian and Laird random effects model for proportions with variance stabilization by applying the Freeman-Tukey double arcsine transformation

†††Pooled using a trivariate generalized linear mixed model that jointly models diagnostic test prevalence and predictive values

#Trivariate random-effects model did not converge

*Estimates for entire algorithm using total Xpert (sputum and/or non-sputum Xpert result)

Definition of abbreviations: BMI = body mass index, CRP = C-reactive protein, CXR = chest X-ray, GLMM = generalized linear mixed model, Hb = haemoglobin, NPV = negative predictive value, PPV = positive predictive value, W4SS = WHO four-symptom screen

Table S9E - Additional diagnostic accuracy estimates in participants with a CD4 cell count >200 cells/µL

									Metaregression†	
	Univariate diagnostic odds ratio				Positive screen (%)††		Trivariate GLMM†††		Reference standard	Prevalence
Test	Estimate (95% CI)	I² (95% CI)	Trim-and-fill estimate (95% CI)	Egger's test (p-value)	Estimate (95% CI)	I² (95% CI)	PPV (95% CI)	NPV (95% CI)	P-value	P-value
W4SS	2.33 (1.73-3.12)	26 (0-57)	2.26 (1.69-3.02)	0.8	52 (38-65)	99 (99-99)	7.3 (5.8-9.1)	96.8 (95.5-97.8)	0.94	0.06
CRP (>=10 mg/L)	6.23 (3.64-10.65)	36 (0-76)	6.23 (3.64-10.65)	0.97	23 (14-37)	95 (92-97)	16.8 (11.2-24.3)	97.2 (94.7-98.5)	-	0.81
CRP (>=8 mg/L)	5.81 (3.27-10.31)	41 (0-78)	5.81 (3.27-10.31)	0.99	27 (16-40)	96 (92-98)	15.1 (9.7-22.8)	97.2 (94.9-98.5)	-	0.93
CRP (>=5 mg/L)	6.56 (4.14-10.42)	0 (0-71)	6.56 (4.14-10.42)	0.8	37 (26-50)	95 (92-97)	12.8 (8.0-20.0)	97.8 (96.2-98.7)	-	0.59
CXR (abnormal)	3.60 (2.33-5.56)	23 (0-65)	3.60 (2.33-5.56)	0.91	37 (28-48)	98 (96-98)	8.3 (5.0-13.5)	97.5 (95.0-98.7)	0.25	0.11
CXR (suggests tuberculosis)	6.51 (3.79-11.16)	52 (0-78)	6.50 (3.82-11.06)	0.82	22 (13-33)	98 (98-99)	15.3 (10.6-21.7)	97.1 (95.2-98.3)	0.13	0.12
Cough (any)	3.16 (2.51-3.99)	13 (0-47)	3.16 (2.51-3.99)	0.94	26 (20-34)	98 (97-98)	10.1 (8.1-12.4)	96.9 (95.7-97.8)	0.52	0.02
Cough (>=2 weeks)	3.45 (2.48-4.81)	29 (0-60)	3.45 (2.48-4.81)	0.98	15 (10-22)	96 (94-97)	13.0 (10.2-16.4)	96.1 (94.6-97.3)	0.57	0.01
Hb (<10 g/dL)	1.99 (1.29-3.07)	0 (0-64)	1.99 (1.29-3.07)	0.99	16 (12-22)	92 (86-95)	6.7 (3.2-13.5)	96.1 (92.7-98.0)	0.31	0.74
Hb (<8 g/dL)	2.87 (1.40-5.89)	0 (0-30)	3.48 (1.76-6.85)	0.51	4 (2-6)	86 (75-92)	7.4 (2.7-18.6)	95.9 (92.2-97.9)	0.45	0.59
BMI (<18.5 kg/m²)	2.86 (2.17-3.75)	0 (0-0)	2.90 (2.21-3.81)	0.55	9 (6-13)	95 (93-96)	11.3 (7.4-16.8)	95.7 (94.0-96.9)	0.47	0.72
Lymphadenopathy	4.02 (2.26-7.16)	0 (0-0)	4.02 (2.26-7.16)	0.73	11 (4-29)	97 (96-98)	12.5 (4.3-31.3)	96.2 (92.1-98.2)	0.69	0.55
W4SS with CRP (>=10 mg/L)	1.66 (1.01-2.72)	0 (0-74)	1.42 (0.87-2.31)	0.17	66 (40-85)	99 (98-99)	7.3 (4.4-11.9)	96.0 (91.8-98.1)	-	0.98
W4SS with CXR (abnormal)	3.56 (1.94-6.54)	19 (0-62)	4.02 (1.85-8.71)	0.33	73 (58-84)	99 (98-99)	6.2 (3.9-9.9)	98.4 (96.2-99.4)	0.62	0.13
W4SS then CRP (>=5 mg/L)	7.56 (3.48-16.40)	57 (0-84)	5.24 (2.61-10.50)	0.52	22 (10-43)	96 (93-98)	15.0 (8.9-24.2)	97.7 (95.7-98.8)	-	0.81
W4SS then Xpert*	128.84 (74.31-223.38)	10 (0-50)	149.81 (83.48-268.82)	0.55	3 (2-5)	83 (72-90)	82.5 (67.5-91.5)	97.1 (95.3-98.2)	0.05	0.09
Xpert alone*	145.18 (78.32-269.13)	36 (0-68)	151.58 (83.62-274.78)	0.78	4 (3-6)	78 (62-87)	77.0 (66.0-85.2)	97.9 (96.0-98.9)	0.02	0.03

†For the meta-regressions, the outcome variable was the diagnostic odds ratio

††Calculated using meta-analysis of proportions. We used a generalized linear mixed model with logit transformation in preference to protocol specified DerSimonian and Laird random effects model for proportions with variance stabilization by applying the Freeman-Tukey double arcsine transformation

†††Pooled using a trivariate generalized linear mixed model that jointly models diagnostic test prevalence and predictive values

*Estimates for entire algorithm using total Xpert (sputum and/or non-sputum Xpert result)

Definition of abbreviations: BMI = body mass index, CRP = C-reactive protein, CXR = chest X-ray, GLMM = generalized linear mixed model, Hb = haemoglobin, NPV = negative predictive value, PPV = positive predictive value, W4SS = WHO four-symptom screen

Table S9F - Additional diagnostic accuracy estimates in pregnant participants

									Metaregression†	
	Univariate diagnostic odds ratio				Positive screen (%)††		Trivariate GLMM†††		Reference standard	Prevalence
Test	Estimate (95% CI)	I² (95% CI)	Trim-and-fill estimate (95% CI)	Egger's test (p-value)	Estimate (95% CI)	I² (95% CI)	PPV (95% CI)	NPV (95% CI)	P-value	P-value
**W4SS**	2.22 (1.23-4.00)	0 (0-0)	2.27 (1.27-4.04)	0.89	43 (26-62)	94 (90-96)	5.8 (3.4-9.8)	98.7 (92.8-99.8)	0.86	0.88
**CRP (>=10 mg/L)#**	-	-	-	-	-	-	-	-	-	-
**CRP (>=8 mg/L)#**	-	-	-	-	-	-	-	-	-	-
**CRP (>=5 mg/L)#**	-	-	-	-	-	-	-	-	-	-
**CXR (abnormal)**	6.60 (0.19-225.79)	-	-	-	38 (12-72)	-	33.3 (1.7-79.2)	100.0 (56.6-100.0)	-	-
**CXR (suggests tuberculosis)**	39.00 (0.53-2883.60)	-	-	-	14 (2-58)	-	100.0 (5.1-100.0)	100.0 (61.0-100.0)	-	-
**Cough (any)**	4.05 (2.15-7.61)	0 (0-0)	3.73 (2.03-6.85)	0.18	20 (13-32)	90 (82-94)	10.0 (5.5-17.5)	98.6 (96.1-99.5)	0.79	0.57
**Cough (>=2 weeks)**	7.25 (3.68-14.27)	0 (0-0)	6.89 (3.55-13.39)	0.64	9 (5-16)	82 (67-91)	17.0 (9.0-29.8)	98.0 (95.6-99.1)	0.9	0.96
**Hb (<10 g/dL)**	1.33 (0.57-3.09)	0 (0-85)	1.33 (0.57-3.09)	0.57	26 (16-40)	78 (47-91)	3.1 (1.5-6.3)	98.2 (89.9-99.7)	0.24	0.51
**Hb (<8 g/dL)##**	1.98 (0.25-15.58)	0 (0-83)	0.69 (0.12-4.11)	0.02	2 (1-3)	6 (0-80)	-	-	0.51	0.29
**BMI (<18.5 kg/m²)**	5.68 (1.39-23.17)	0 (0-69)	5.68 (1.39-23.17)	0.85	4 (2-6)	32 (0-71)	1.3 (0.0-93.6)	97.5 (95.3-98.7)	0.96	0.76
**Lymphadenopathy#**	-	-	-	-	-	-	-	-	-	-
**W4SS with CRP (>=10 mg/L)¶#**	-	-	-	-	-	-	-	-	-	-
**W4SS with CXR (abnormal)**	3.86 (0.12-126.73)	-	-	-	50 (20-80)	-	25.0 (1.3-69.9)	100.0 (51.0-100.0)	-	-
**W4SS then CRP (>=5 mg/L)¶#**	-	-	-	-	-	-	-	-	-	-
**W4SS then Xpert***	165.04 (24.50-1111.61)	0 (0-6)	165.04 (24.50-1111.61)	0.12	1 (0-2)	0 (0-29)	100.0 (0.0-100.0)	98.1 (96.5-99.0)	-	0.67
**Xpert alone***	141.49 (31.79-629.82)	0 (0-0)	158.18 (39.84-627.97)	0.64	2 (1-4)	0 (0-76)	73.4 (40.5-91.8)	98.5 (97.0-99.3)	-	0.66

†For the meta-regressions, the outcome variable was the diagnostic odds ratio

††Calculated using meta-analysis of proportions. We used a generalized linear mixed model with logit transformation in preference to protocol specified DerSimonian and Laird random effects model for proportions with variance stabilization by applying the Freeman-Tukey double arcsine transformation

†††Pooled using a trivariate generalized linear mixed model that jointly models diagnostic test prevalence and predictive values

#Insufficient data to perform meta-analysis

##Trivariate random-effects model did not converge

*Estimates for entire algorithm using total Xpert (sputum and/or non-sputum Xpert result)

Definition of abbreviations: BMI = body mass index, CRP = C-reactive protein, CXR = chest X-ray, GLMM = generalized linear mixed model, Hb = haemoglobin, NPV = negative predictive value, PPV = positive predictive value, W4SS = WHO four-symptom screen

Table S10 - Yield of different screening and diagnostic algorithms when screening a population of 1000 persons

Table S10A - Yield of different screening and diagnostic algorithms at different prevalences when screening a population of 1000 participants

		Outcome of screening§							Outcome of screening then diagnosis§						
**Test**	**Total TB**	**TP+FP‡**	**TP**	**FP**	**TN**	**FN**	**PPV**	**NPV**	**TP**	**TN**	**FP**	**FN**	**PPV**	**NPV**	**NNS**
	1% prevalence														
**W4SS**	10	582	8	574	416	2	1.4	99.6	-	-	-	-	-	-	-
**CRP (>=10 mg/L)**	10	265	8	257	733	2	2.9	99.7	-	-	-	-	-	-	-
**CRP (>=8 mg/L)**	10	305	8	297	693	2	2.7	99.7	-	-	-	-	-	-	-
**CRP (>=5 mg/L)**	10	405	9	396	594	1	2.1	99.8	-	-	-	-	-	-	-
**CXR (abnormal)**	10	383	7	376	614	3	1.9	99.5	-	-	-	-	-	-	-
**CXR (suggests tuberculosis)**	10	224	6	218	772	4	2.8	99.5	-	-	-	-	-	-	-
**Cough (any)**	10	283	6	277	713	4	2	99.4	-	-	-	-	-	-	-
**Cough (>=2 weeks)**	10	162	4	158	832	6	2.3	99.3	-	-	-	-	-	-	-
**Hb (<10 g/dL)**	10	202	4	198	792	6	2.1	99.3	-	-	-	-	-	-	-
**Hb (<8 g/dL)**	10	41	1	40	950	9	2.9	99.1	-	-	-	-	-	-	-
**BMI (<18.5 kg/m²)**	10	112	3	109	881	7	2.6	99.2	-	-	-	-	-	-	-
**Lymphadenopathy**	10	102	3	99	891	7	3	99.2	-	-	-	-	-	-	-
**W4SS with CRP (>=10 mg/L)¶**	10	692	9	683	307	1	1.3	99.6	-	-	-	-	-	-	-
**W4SS with CXR (abnormal)¶**	10	801	9	792	198	1	1.2	99.7	-	-	-	-	-	-	-
**W4SS then CRP (>=5 mg/L)¶**	10	255	7	248	742	3	2.8	99.6	-	-	-	-	-	-	-
**Xpert alone*†**	10	-	-	-	-	-	-	-	7	980	10	3	40.7	99.7	143
**WHO screen then Xpert*†**	10	-	-	-	-	-	-	-	6	980	10	4	36.9	99.6	167
	5% prevalence														
**W4SS**	50	592	41	551	399	9	6.9	97.8	-	-	-	-	-	-	-
**CRP (>=10 mg/L)**	50	285	38	247	703	12	13.5	98.4	-	-	-	-	-	-	-
**CRP (>=8 mg/L)**	50	325	40	285	665	10	12.4	98.6	-	-	-	-	-	-	-
**CRP (>=5 mg/L)**	50	424	44	380	570	6	10.3	98.9	-	-	-	-	-	-	-
**CXR (abnormal)**	50	397	36	361	589	14	9.1	97.7	-	-	-	-	-	-	-
**CXR (suggests tuberculosis)**	50	241	32	209	741	18	13.1	97.6	-	-	-	-	-	-	-
**Cough (any)**	50	294	28	266	684	22	9.5	96.9	-	-	-	-	-	-	-
**Cough (>=2 weeks)**	50	171	19	152	798	31	11.1	96.3	-	-	-	-	-	-	-
**Hb (<10 g/dL)**	50	212	22	190	760	28	10.2	96.4	-	-	-	-	-	-	-
**Hb (<8 g/dL)**	50	44	6	38	912	44	13.6	95.4	-	-	-	-	-	-	-
**BMI (<18.5 kg/m²)**	50	118	14	104	846	36	12.2	96	-	-	-	-	-	-	-
**Lymphadenopathy**	50	111	16	95	855	34	14	96.1	-	-	-	-	-	-	-
**W4SS with CRP (>=10 mg/L)¶**	50	700	44	656	294	6	6.3	98	-	-	-	-	-	-	-
**W4SS with CXR (abnormal)¶**	50	807	47	760	190	3	5.8	98.4	-	-	-	-	-	-	-
**W4SS then CRP (>=5 mg/L)¶**	50	273	35	238	712	15	12.8	97.9	-	-	-	-	-	-	-
**Xpert alone*†**	50	-	-	-	-	-	-	-	34	940	10	16	78.2	98.3	29
**WHO screen then Xpert*†**	50	-	-	-	-	-	-	-	29	940	10	21	75.3	97.8	34

		Outcome of screening§							Outcome of screening then diagnosis§						
**Test**	**Total TB**	**TP+FP‡**	**TP**	**FP**	**TN**	**FN**	**PPV**	**NPV**	**TP**	**TN**	**FP**	**FN**	**PPV**	**NPV**	**NNS**
	10% prevalence														
**W4SS**	100	604	82	522	378	18	13.6	95.5	-	-	-	-	-	-	-
**CRP (>=10 mg/L)**	100	311	77	234	666	23	24.8	96.7	-	-	-	-	-	-	-
**CRP (>=8 mg/L)**	100	351	81	270	630	19	23.1	97.1	-	-	-	-	-	-	-
**CRP (>=5 mg/L)**	100	447	87	360	540	13	19.5	97.6	-	-	-	-	-	-	-
**CXR (abnormal)**	100	414	72	342	558	28	17.4	95.2	-	-	-	-	-	-	-
**CXR (suggests tuberculosis)**	100	261	63	198	702	37	24.1	95	-	-	-	-	-	-	-
**Cough (any)**	100	308	56	252	648	44	18.2	93.6	-	-	-	-	-	-	-
**Cough (>=2 weeks)**	100	182	38	144	756	62	20.9	92.4	-	-	-	-	-	-	-
**Hb (<10 g/dL)**	100	223	43	180	720	57	19.3	92.7	-	-	-	-	-	-	-
**Hb (<8 g/dL)**	100	48	12	36	864	88	25	90.8	-	-	-	-	-	-	-
**BMI (<18.5 kg/m²)**	100	128	29	99	801	71	22.7	91.9	-	-	-	-	-	-	-
**Lymphadenopathy**	100	121	31	90	810	69	25.6	92.2	-	-	-	-	-	-	-
**W4SS with CRP (>=10 mg/L)¶**	100	709	88	621	279	12	12.4	95.9	-	-	-	-	-	-	-
**W4SS with CXR (abnormal)¶**	100	814	94	720	180	6	11.5	96.8	-	-	-	-	-	-	-
**W4SS then CRP (>=5 mg/L)¶**	100	295	70	225	675	30	23.7	95.7	-	-	-	-	-	-	-
**Xpert alone*†**	100	-	-	-	-	-	-	-	68	891	9	32	88.3	96.5	15
**WHO screen then Xpert*†**	100	-	-	-	-	-	-	-	58	891	9	42	86.6	95.5	17
	20% prevalence														
**W4SS**	200	628	164	464	336	36	26.1	90.3	-	-	-	-	-	-	-
**CRP (>=10 mg/L)**	200	362	154	208	592	46	42.5	92.8	-	-	-	-	-	-	-
**CRP (>=8 mg/L)**	200	402	162	240	560	38	40.3	93.6	-	-	-	-	-	-	-
**CRP (>=5 mg/L)**	200	494	174	320	480	26	35.2	94.9	-	-	-	-	-	-	-
**CXR (abnormal)**	200	448	144	304	496	56	32.1	89.9	-	-	-	-	-	-	-
**CXR (suggests tuberculosis)**	200	302	126	176	624	74	41.7	89.4	-	-	-	-	-	-	-
**Cough (any)**	200	336	112	224	576	88	33.3	86.7	-	-	-	-	-	-	-
**Cough (>=2 weeks)**	200	204	76	128	672	124	37.3	84.4	-	-	-	-	-	-	-
**Hb (<10 g/dL)**	200	246	86	160	640	114	35	84.9	-	-	-	-	-	-	-
**Hb (<8 g/dL)**	200	56	24	32	768	176	42.9	81.4	-	-	-	-	-	-	-
**BMI (<18.5 kg/m²)**	200	146	58	88	712	142	39.7	83.4	-	-	-	-	-	-	-
**Lymphadenopathy**	200	142	62	80	720	138	43.7	83.9	-	-	-	-	-	-	-
**W4SS with CRP (>=10 mg/L)¶**	200	728	176	552	248	24	24.2	91.2	-	-	-	-	-	-	-
**W4SS with CXR (abnormal)¶**	200	828	188	640	160	12	22.7	93	-	-	-	-	-	-	-
**W4SS then CRP (>=5 mg/L)¶**	200	340	140	200	600	60	41.2	90.9	-	-	-	-	-	-	-
**Xpert alone*†**	200	-	-	-	-	-	-	-	136	792	8	64	94.4	92.5	7
**WHO screen then Xpert*†**	200	-	-	-	-	-	-	-	116	792	8	84	93.5	90.4	9

§Estimated using the pooled point estimates for sensitivity and specificity for different tests/strategies

‡TP+FP is the number of participants who screen positive (i.e. the number who need subsequent Xpert testing)

		Outcome of screening§							Outcome of screening then diagnosis§						
Test	Total TB	TP+FP‡	TP	FP	TN	FN	PPV	NPV	TP	TN	FP	FN	PPV	NPV	NNS

¶For parallel strategies, two screening tests are offered at the same time. For sequential strategies, a second screening test is offered only if the first screening test is positive

*Accuracy measures for entire algorithm using total Xpert (sputum and/or non-sputum Xpert result)

†The test accuracy of Xpert in those who were W4SS positive was: 12 studies; 4558 participants; sensitivity 0.74 (0.65-0.81), specificity 0.98 (0.97-0.99).

Definition of abbreviations: BMI = body mass index, CRP = C-reactive protein, CXR = chest X-ray, FN= false negative, FP = false positive, Hb = haemoglobin, NNS = number needed to screen, NPV = negative predictive value, PPV = positive predictive value, TB = tuberculosis, TN = true negative, TP = true positive, W4SS = WHO four-symptom screen

Table S10B - Yield of different screening and diagnostic algorithms at different prevalences when screening a population of 1000 outpatients (on ART)

		Outcome of screening§							Outcome of screening then diagnosis§						
**Test**	**Total TB**	**TP+FP‡**	**TP**	**FP**	**TN**	**FN**	**PPV**	**NPV**	**TP**	**TN**	**FP**	**FN**	**PPV**	**NPV**	**NNS**
	1% prevalence														
**W4SS**	10	292	5	287	703	5	1.8	99.3	-	-	-	-	-	-	-
**CRP (>=10 mg/L)**	10	101	2	99	891	8	2	99.1	-	-	-	-	-	-	-
**CRP (>=8 mg/L)**	10	113	4	109	881	6	3.5	99.3	-	-	-	-	-	-	-
**CRP (>=5 mg/L)**	10	202	4	198	792	6	2	99.2	-	-	-	-	-	-	-
**CXR (abnormal)**	10	373	7	366	624	3	2	99.6	-	-	-	-	-	-	-
**CXR (suggests tuberculosis)**	10	225	7	218	772	3	3.1	99.6	-	-	-	-	-	-	-
**Cough (any)**	10	172	4	168	822	6	2.3	99.3	-	-	-	-	-	-	-
**Cough (>=2 weeks)**	10	71	2	69	921	8	2.7	99.1	-	-	-	-	-	-	-
**Hb (<10 g/dL)**	10	184	6	178	812	4	3	99.4	-	-	-	-	-	-	-
**Hb (<8 g/dL)**	10	61	2	59	931	8	2.9	99.1	-	-	-	-	-	-	-
**BMI (<18.5 kg/m²)**	10	71	2	69	921	8	2.3	99.1	-	-	-	-	-	-	-
**Lymphadenopathy**	10	71	2	69	921	8	3.1	99.2	-	-	-	-	-	-	-
**W4SS with CRP (>=10 mg/L)¶**	10	200	2	198	792	8	1	99	-	-	-	-	-	-	-
**W4SS with CXR (abnormal)¶**	10	672	9	663	327	1	1.3	99.7	-	-	-	-	-	-	-
**W4SS then CRP (>=5 mg/L)¶**	10	41	1	40	950	9	2	99	-	-	-	-	-	-	-
**Xpert alone*†**	10	-	-	-	-	-	-	-	5	980	10	5	34.9	99.5	200
**WHO screen then Xpert*†**	10	-	-	-	-	-	-	-	4	990	0	6	100	99.4	250
	5% prevalence														
**W4SS**	50	302	26	276	674	24	8.8	96.6	-	-	-	-	-	-	-
**CRP (>=10 mg/L)**	50	105	10	95	855	40	9.5	95.5	-	-	-	-	-	-	-
**CRP (>=8 mg/L)**	50	124	20	104	846	30	16.1	96.6	-	-	-	-	-	-	-
**CRP (>=5 mg/L)**	50	210	20	190	760	30	9.5	96.2	-	-	-	-	-	-	-
**CXR (abnormal)**	50	388	36	352	598	14	9.4	97.8	-	-	-	-	-	-	-
**CXR (suggests tuberculosis)**	50	244	35	209	741	15	14.3	98	-	-	-	-	-	-	-
**Cough (any)**	50	181	20	161	789	30	11	96.3	-	-	-	-	-	-	-
**Cough (>=2 weeks)**	50	76	10	66	884	40	12.5	95.6	-	-	-	-	-	-	-
**Hb (<10 g/dL)**	50	199	28	171	779	22	13.9	97.2	-	-	-	-	-	-	-
**Hb (<8 g/dL)**	50	66	9	57	893	41	13.6	95.6	-	-	-	-	-	-	-
**BMI (<18.5 kg/m²)**	50	74	8	66	884	42	10.7	95.5	-	-	-	-	-	-	-
**Lymphadenopathy**	50	77	11	66	884	39	14.2	95.8	-	-	-	-	-	-	-
**W4SS with CRP (>=10 mg/L)¶**	50	200	10	190	760	40	5	95	-	-	-	-	-	-	-
**W4SS with CXR (abnormal)¶**	50	680	44	636	314	6	6.5	98.3	-	-	-	-	-	-	-
**W4SS then CRP (>=5 mg/L)¶**	50	42	4	38	912	46	9.5	95.2	-	-	-	-	-	-	-
**Xpert alone*†**	50	-	-	-	-	-	-	-	26	940	10	24	73.6	97.6	38
**WHO screen then Xpert*†**	50	-	-	-	-	-	-	-	18	950	0	32	100	96.8	56
	10% prevalence														

Test	Total TB	Outcome of screening§							Outcome of screening then diagnosis§						
		TP+FP‡	TP	FP	TN	FN	PPV	NPV	TP	TN	FP	FN	PPV	NPV	NNS
**W4SS**	100	314	53	261	639	47	16.9	93.1	-	-	-	-	-	-	-
**CRP (>=10 mg/L)**	100	110	20	90	810	80	18.2	91	-	-	-	-	-	-	-
**CRP (>=8 mg/L)**	100	139	40	99	801	60	28.8	93	-	-	-	-	-	-	-
**CRP (>=5 mg/L)**	100	220	40	180	720	60	18.2	92.3	-	-	-	-	-	-	-
**CXR (abnormal)**	100	406	73	333	567	27	18	95.5	-	-	-	-	-	-	-
**CXR (suggests tuberculosis)**	100	268	70	198	702	30	26.1	95.9	-	-	-	-	-	-	-
**Cough (any)**	100	193	40	153	747	60	20.7	92.6	-	-	-	-	-	-	-
**Cough (>=2 weeks)**	100	82	19	63	837	81	23.2	91.2	-	-	-	-	-	-	-
**Hb (<10 g/dL)**	100	217	55	162	738	45	25.3	94.3	-	-	-	-	-	-	-
**Hb (<8 g/dL)**	100	72	18	54	846	82	25	91.2	-	-	-	-	-	-	-
**BMI (<18.5 kg/m²)**	100	79	16	63	837	84	20.3	90.9	-	-	-	-	-	-	-
**Lymphadenopathy**	100	85	22	63	837	78	25.9	91.5	-	-	-	-	-	-	-
**W4SS with CRP (>=10 mg/L)¶**	100	200	20	180	720	80	10	90	-	-	-	-	-	-	-
**W4SS with CXR (abnormal)¶**	100	692	89	603	297	11	12.9	96.4	-	-	-	-	-	-	-
**W4SS then CRP (>=5 mg/L)¶**	100	44	8	36	864	92	18.2	90.4	-	-	-	-	-	-	-
**Xpert alone*†**	100	-	-	-	-	-	-	-	53	891	9	47	85.5	95	19
**WHO screen then Xpert*†**	100	-	-	-	-	-	-	-	37	900	0	63	100	93.5	27
	20% prevalence														
**W4SS**	200	338	106	232	568	94	31.4	85.8	-	-	-	-	-	-	-
**CRP (>=10 mg/L)**	200	120	40	80	720	160	33.3	81.8	-	-	-	-	-	-	-
**CRP (>=8 mg/L)**	200	168	80	88	712	120	47.6	85.6	-	-	-	-	-	-	-
**CRP (>=5 mg/L)**	200	240	80	160	640	120	33.3	84.2	-	-	-	-	-	-	-
**CXR (abnormal)**	200	442	146	296	504	54	33	90.3	-	-	-	-	-	-	-
**CXR (suggests tuberculosis)**	200	316	140	176	624	60	44.3	91.2	-	-	-	-	-	-	-
**Cough (any)**	200	216	80	136	664	120	37	84.7	-	-	-	-	-	-	-
**Cough (>=2 weeks)**	200	94	38	56	744	162	40.4	82.1	-	-	-	-	-	-	-
**Hb (<10 g/dL)**	200	254	110	144	656	90	43.3	87.9	-	-	-	-	-	-	-
**Hb (<8 g/dL)**	200	84	36	48	752	164	42.9	82.1	-	-	-	-	-	-	-
**BMI (<18.5 kg/m²)**	200	88	32	56	744	168	36.4	81.6	-	-	-	-	-	-	-
**Lymphadenopathy**	200	100	44	56	744	156	44	82.7	-	-	-	-	-	-	-
**W4SS with CRP (>=10 mg/L)¶**	200	200	40	160	640	160	20	80	-	-	-	-	-	-	-
**W4SS with CXR (abnormal)¶**	200	714	178	536	264	22	24.9	92.3	-	-	-	-	-	-	-
**W4SS then CRP (>=5 mg/L)¶**	200	48	16	32	768	184	33.3	80.7	-	-	-	-	-	-	-
**Xpert alone*†**	200	-	-	-	-	-	-	-	106	792	8	94	93	89.4	9
**WHO screen then Xpert*†**	200	-	-	-	-	-	-	-	74	800	0	126	100	86.4	14

§Estimated using the pooled point estimates for sensitivity and specificity for different tests/strategies

‡TP+FP is the number of participants who screen positive (i.e. the number who need subsequent Xpert testing)

¶For parallel strategies, two screening tests are offered at the same time. For sequential strategies, a second screening test is offered only if the first screening test is positive

*Accuracy measures for entire algorithm using total Xpert (sputum and/or non-sputum Xpert result)

		Outcome of screening§						Outcome of screening then diagnosis§						
Test	Total TB	TP+FP‡	TP	FP	TN	FN	PPV	NPV	TP	TN	FP	FN	PPV	NPV	NNS

†The test accuracy of Xpert in those who were W4SS positive was: 4 studies; 564 participants; sensitivity 0.91 (0.42-0.99), specificity 0.98 (0.91-1).

Definition of abbreviations: BMI = body mass index, CRP = C-reactive protein, CXR = chest X-ray, FN= false negative, FP = false positive, Hb = haemoglobin, NNS = number needed to screen, NPV = negative predictive value, PPV = positive predictive value, TB = tuberculosis, TN = true negative, TP = true positive, W4SS = WHO four-symptom screen

Table S10C - Yield of different screening and diagnostic algorithms at different prevalences when screening a population of 1000 outpatients (not on ART)

		Outcome of screening§							Outcome of screening then diagnosis§						
**Test**	**Total TB**	**TP+FP‡**	**TP**	**FP**	**TN**	**FN**	**PPV**	**NPV**	**TP**	**TN**	**FP**	**FN**	**PPV**	**NPV**	**NNS**
	1% prevalence														
**W4SS**	10	632	8	624	366	2	1.3	99.6	-	-	-	-	-	-	-
**CRP (>=10 mg/L)**	10	335	8	327	663	2	2.5	99.7	-	-	-	-	-	-	-
**CRP (>=8 mg/L)**	10	374	8	366	624	2	2.3	99.8	-	-	-	-	-	-	-
**CRP (>=5 mg/L)**	10	474	9	465	525	1	1.9	99.8	-	-	-	-	-	-	-
**CXR (abnormal)**	10	383	7	376	614	3	1.9	99.5	-	-	-	-	-	-	-
**CXR (suggests tuberculosis)**	10	214	6	208	782	4	2.9	99.5	-	-	-	-	-	-	-
**Cough (any)**	10	303	6	297	693	4	1.9	99.4	-	-	-	-	-	-	-
**Cough (>=2 weeks)**	10	182	4	178	812	6	2.4	99.3	-	-	-	-	-	-	-
**Hb (<10 g/dL)**	10	222	4	218	772	6	2	99.3	-	-	-	-	-	-	-
**Hb (<8 g/dL)**	10	41	1	40	950	9	2.7	99.1	-	-	-	-	-	-	-
**BMI (<18.5 kg/m²)**	10	132	3	129	861	7	2.4	99.2	-	-	-	-	-	-	-
**Lymphadenopathy**	10	92	3	89	901	7	3.3	99.2	-	-	-	-	-	-	-
**W4SS with CRP (>=10 mg/L)¶**	10	801	9	792	198	1	1.2	99.7	-	-	-	-	-	-	-
**W4SS with CXR (abnormal)¶**	10	822	10	812	178	0	1.2	99.7	-	-	-	-	-	-	-
**W4SS then CRP (>=5 mg/L)¶**	10	364	8	356	634	2	2.3	99.7	-	-	-	-	-	-	-
**Xpert alone*†**	10	-	-	-	-	-	-	-	7	980	10	3	42.8	99.7	143
**WHO screen then Xpert*†**	10	-	-	-	-	-	-	-	6	980	10	4	39.3	99.6	167
	5% prevalence														
**W4SS**	50	640	42	598	352	8	6.6	97.9	-	-	-	-	-	-	-
**CRP (>=10 mg/L)**	50	355	42	313	637	8	11.7	98.7	-	-	-	-	-	-	-
**CRP (>=8 mg/L)**	50	394	42	352	598	8	10.8	98.8	-	-	-	-	-	-	-
**CRP (>=5 mg/L)**	50	490	44	446	504	6	9.1	98.9	-	-	-	-	-	-	-
**CXR (abnormal)**	50	397	36	361	589	14	9	97.6	-	-	-	-	-	-	-
**CXR (suggests tuberculosis)**	50	230	31	199	751	19	13.4	97.5	-	-	-	-	-	-	-
**Cough (any)**	50	314	29	285	665	21	9.2	96.9	-	-	-	-	-	-	-
**Cough (>=2 weeks)**	50	193	22	171	779	28	11.2	96.5	-	-	-	-	-	-	-
**Hb (<10 g/dL)**	50	231	22	209	741	28	9.5	96.4	-	-	-	-	-	-	-
**Hb (<8 g/dL)**	50	44	6	38	912	44	12.6	95.3	-	-	-	-	-	-	-
**BMI (<18.5 kg/m²)**	50	140	16	124	826	34	11.2	96	-	-	-	-	-	-	-
**Lymphadenopathy**	50	100	15	85	865	35	14.9	96.1	-	-	-	-	-	-	-
**W4SS with CRP (>=10 mg/L)¶**	50	807	47	760	190	3	5.8	98.4	-	-	-	-	-	-	-
**W4SS with CXR (abnormal)¶**	50	827	48	779	171	2	5.7	98.6	-	-	-	-	-	-	-
**W4SS then CRP (>=5 mg/L)¶**	50	384	42	342	608	8	10.9	98.7	-	-	-	-	-	-	-
**Xpert alone*†**	50	-	-	-	-	-	-	-	37	940	10	13	79.6	98.6	27
**WHO screen then Xpert*†**	50	-	-	-	-	-	-	-	32	940	10	18	77.1	98.1	31
	10% prevalence														

		Outcome of screening§							Outcome of screening then diagnosis§						
**Test**	**Total TB**	**TP+FP‡**	**TP**	**FP**	**TN**	**FN**	**PPV**	**NPV**	**TP**	**TN**	**FP**	**FN**	**PPV**	**NPV**	**NNS**
**W4SS**	100	652	85	567	333	15	13	95.7	-	-	-	-	-	-	-
**CRP (>=10 mg/L)**	100	380	83	297	603	17	21.8	97.3	-	-	-	-	-	-	-
**CRP (>=8 mg/L)**	100	418	85	333	567	15	20.3	97.4	-	-	-	-	-	-	-
**CRP (>=5 mg/L)**	100	512	89	423	477	11	17.4	97.7	-	-	-	-	-	-	-
**CXR (abnormal)**	100	413	71	342	558	29	17.2	95.1	-	-	-	-	-	-	-
**CXR (suggests tuberculosis)**	100	251	62	189	711	38	24.7	94.9	-	-	-	-	-	-	-
**Cough (any)**	100	328	58	270	630	42	17.7	93.8	-	-	-	-	-	-	-
**Cough (>=2 weeks)**	100	205	43	162	738	57	21	92.8	-	-	-	-	-	-	-
**Hb (<10 g/dL)**	100	242	44	198	702	56	18.2	92.6	-	-	-	-	-	-	-
**Hb (<8 g/dL)**	100	47	11	36	864	89	23.4	90.7	-	-	-	-	-	-	-
**BMI (<18.5 kg/m²)**	100	148	31	117	783	69	20.9	91.9	-	-	-	-	-	-	-
**Lymphadenopathy**	100	111	30	81	819	70	27	92.1	-	-	-	-	-	-	-
**W4SS with CRP (>=10 mg/L)¶**	100	814	94	720	180	6	11.5	96.8	-	-	-	-	-	-	-
**W4SS with CXR (abnormal)¶**	100	833	95	738	162	5	11.4	97	-	-	-	-	-	-	-
**W4SS then CRP (>=5 mg/L)¶**	100	408	84	324	576	16	20.6	97.3	-	-	-	-	-	-	-
**Xpert alone*†**	100	-	-	-	-	-	-	-	74	891	9	26	89.2	97.2	14
**WHO screen then Xpert*†**	100	-	-	-	-	-	-	-	64	891	9	36	87.7	96.1	16
	20% prevalence														
**W4SS**	200	674	170	504	296	30	25.2	90.8	-	-	-	-	-	-	-
**CRP (>=10 mg/L)**	200	430	166	264	536	34	38.6	94	-	-	-	-	-	-	-
**CRP (>=8 mg/L)**	200	466	170	296	504	30	36.5	94.4	-	-	-	-	-	-	-
**CRP (>=5 mg/L)**	200	554	178	376	424	22	32.1	95.1	-	-	-	-	-	-	-
**CXR (abnormal)**	200	446	142	304	496	58	31.8	89.5	-	-	-	-	-	-	-
**CXR (suggests tuberculosis)**	200	292	124	168	632	76	42.5	89.3	-	-	-	-	-	-	-
**Cough (any)**	200	356	116	240	560	84	32.6	87	-	-	-	-	-	-	-
**Cough (>=2 weeks)**	200	230	86	144	656	114	37.4	85.2	-	-	-	-	-	-	-
**Hb (<10 g/dL)**	200	264	88	176	624	112	33.3	84.8	-	-	-	-	-	-	-
**Hb (<8 g/dL)**	200	54	22	32	768	178	40.7	81.2	-	-	-	-	-	-	-
**BMI (<18.5 kg/m²)**	200	166	62	104	696	138	37.3	83.5	-	-	-	-	-	-	-
**Lymphadenopathy**	200	132	60	72	728	140	45.5	83.9	-	-	-	-	-	-	-
**W4SS with CRP (>=10 mg/L)¶**	200	828	188	640	160	12	22.7	93	-	-	-	-	-	-	-
**W4SS with CXR (abnormal)¶**	200	846	190	656	144	10	22.5	93.5	-	-	-	-	-	-	-
**W4SS then CRP (>=5 mg/L)¶**	200	456	168	288	512	32	36.8	94.1	-	-	-	-	-	-	-
**Xpert alone*†**	200	-	-	-	-	-	-	-	148	792	8	52	94.9	93.8	7
**WHO screen then Xpert*†**	200	-	-	-	-	-	-	-	128	792	8	72	94.1	91.7	8

§Estimated using the pooled point estimates for sensitivity and specificity for different tests/strategies

‡TP+FP is the number of participants who screen positive (i.e. the number who need subsequent Xpert testing)

¶For parallel strategies, two screening tests are offered at the same time. For sequential strategies, a second screening test is offered only if the first screening test is positive

*Accuracy measures for entire algorithm using total Xpert (sputum and/or non-sputum Xpert result)

		Outcome of screening§						Outcome of screening then diagnosis§						
Test	Total TB	TP+FP‡	TP	FP	TN	FN	PPV	NPV	TP	TN	FP	FN	PPV	NPV	NNS

†The test accuracy of Xpert in those who were W4SS positive was: 10 studies; 3909 participants; sensitivity 0.76 (0.67-0.83), specificity 0.98 (0.97-0.99).

Definition of abbreviations: BMI = body mass index, CRP = C-reactive protein, CXR = chest X-ray, FN= false negative, FP = false positive, Hb = haemoglobin, NNS = number needed to screen, NPV = negative predictive value, PPV = positive predictive value, TB = tuberculosis, TN = true negative, TP = true positive, W4SS = WHO four-symptom screen

Table S10D - Yield of different screening and diagnostic algorithms at different prevalences when screening a population of 1000 participants with a CD4 cell count <=200 cells/µL

		**Outcome of screening§**							**Outcome of screening then diagnosis§**						
**Test**	**Total TB**	**TP+FP‡**	**TP**	**FP**	**TN**	**FN**	**PPV**	**NPV**	**TP**	**TN**	**FP**	**FN**	**PPV**	**NPV**	**NNS**
	1% prevalence														
**W4SS**	10	680	44	636	314	6	6.4	98	-	-	-	-	-	-	-
**CRP (>=10 mg/L)**	10	405	44	361	589	6	10.9	99	-	-	-	-	-	-	-
**CRP (>=8 mg/L)**	10	444	45	399	551	5	10.1	99.1	-	-	-	-	-	-	-
**CRP (>=5 mg/L)**	10	540	46	494	456	4	8.5	99.1	-	-	-	-	-	-	-
**CXR (abnormal)**	10	435	36	399	551	14	8.4	97.6	-	-	-	-	-	-	-
**CXR (suggests tuberculosis)**	10	279	32	247	703	18	11.5	97.5	-	-	-	-	-	-	-
**Cough (any)**	10	353	30	323	627	20	8.4	96.8	-	-	-	-	-	-	-
**Cough (>=2 weeks)**	10	230	21	209	741	29	9.1	96.2	-	-	-	-	-	-	-
**Hb (<10 g/dL)**	10	274	27	247	703	23	9.9	96.8	-	-	-	-	-	-	-
**Hb (<8 g/dL)**	10	54	7	47	903	43	12.8	95.5	-	-	-	-	-	-	-
**BMI (<18.5 kg/m²)**	10	179	18	161	789	32	9.8	96	-	-	-	-	-	-	-
**Lymphadenopathy**	10	120	16	104	846	34	13.6	96.2	-	-	-	-	-	-	-
**W4SS with CRP (>=10 mg/L)¶**	10	778	46	732	218	4	6	98.4	-	-	-	-	-	-	-
**W4SS with CXR (abnormal)¶**	10	865	48	817	133	2	5.5	98.5	-	-	-	-	-	-	-
**W4SS then CRP (>=5 mg/L)¶**	10	363	40	323	627	10	11	98.4	-	-	-	-	-	-	-
**Xpert alone*†**	10	-	-	-	-	-	-	-	38	931	19	12	67	98.8	26
**WHO screen then Xpert*†**	10	-	-	-	-	-	-	-	34	931	19	16	64.5	98.4	29
	5% prevalence														
**W4SS**	50	690	87	603	297	13	12.6	95.8	-	-	-	-	-	-	-
**CRP (>=10 mg/L)**	50	430	88	342	558	12	20.5	97.9	-	-	-	-	-	-	-
**CRP (>=8 mg/L)**	50	468	90	378	522	10	19.2	98.1	-	-	-	-	-	-	-
**CRP (>=5 mg/L)**	50	560	92	468	432	8	16.4	98.2	-	-	-	-	-	-	-
**CXR (abnormal)**	50	451	73	378	522	27	16.2	95.1	-	-	-	-	-	-	-
**CXR (suggests tuberculosis)**	50	298	64	234	666	36	21.5	94.9	-	-	-	-	-	-	-
**Cough (any)**	50	365	59	306	594	41	16.2	93.5	-	-	-	-	-	-	-
**Cough (>=2 weeks)**	50	240	42	198	702	58	17.5	92.4	-	-	-	-	-	-	-
**Hb (<10 g/dL)**	50	288	54	234	666	46	18.8	93.5	-	-	-	-	-	-	-
**Hb (<8 g/dL)**	50	59	14	45	855	86	23.7	90.9	-	-	-	-	-	-	-
**BMI (<18.5 kg/m²)**	50	188	35	153	747	65	18.6	92	-	-	-	-	-	-	-
**Lymphadenopathy**	50	132	33	99	801	67	25	92.3	-	-	-	-	-	-	-
**W4SS with CRP (>=10 mg/L)¶**	50	786	93	693	207	7	11.8	96.7	-	-	-	-	-	-	-
**W4SS with CXR (abnormal)¶**	50	870	96	774	126	4	11	96.9	-	-	-	-	-	-	-
**W4SS then CRP (>=5 mg/L)¶**	50	386	80	306	594	20	20.7	96.7	-	-	-	-	-	-	-
**Xpert alone*†**	50	-	-	-	-	-	-	-	77	882	18	23	81.1	97.5	13
**WHO screen then Xpert*†**	50	-	-	-	-	-	-	-	69	882	18	31	79.3	96.6	14
	10% prevalence														

		Outcome of screening§							Outcome of screening then diagnosis§						
**Test**	**Total TB**	**TP+FP‡**	**TP**	**FP**	**TN**	**FN**	**PPV**	**NPV**	**TP**	**TN**	**FP**	**FN**	**PPV**	**NPV**	**NNS**
**W4SS**	100	710	174	536	264	26	24.5	91	-	-	-	-	-	-	-
**CRP (>=10 mg/L)**	100	480	176	304	496	24	36.7	95.4	-	-	-	-	-	-	-
**CRP (>=8 mg/L)**	100	516	180	336	464	20	34.9	95.9	-	-	-	-	-	-	-
**CRP (>=5 mg/L)**	100	600	184	416	384	16	30.7	96	-	-	-	-	-	-	-
**CXR (abnormal)**	100	482	146	336	464	54	30.3	89.6	-	-	-	-	-	-	-
**CXR (suggests tuberculosis)**	100	336	128	208	592	72	38.1	89.2	-	-	-	-	-	-	-
**Cough (any)**	100	390	118	272	528	82	30.3	86.6	-	-	-	-	-	-	-
**Cough (>=2 weeks)**	100	260	84	176	624	116	32.3	84.3	-	-	-	-	-	-	-
**Hb (<10 g/dL)**	100	316	108	208	592	92	34.2	86.5	-	-	-	-	-	-	-
**Hb (<8 g/dL)**	100	68	28	40	760	172	41.2	81.5	-	-	-	-	-	-	-
**BMI (<18.5 kg/m²)**	100	206	70	136	664	130	34	83.6	-	-	-	-	-	-	-
**Lymphadenopathy**	100	154	66	88	712	134	42.9	84.2	-	-	-	-	-	-	-
**W4SS with CRP (>=10 mg/L)¶**	100	802	186	616	184	14	23.2	92.9	-	-	-	-	-	-	-
**W4SS with CXR (abnormal)¶**	100	880	192	688	112	8	21.8	93.3	-	-	-	-	-	-	-
**W4SS then CRP (>=5 mg/L)¶**	100	432	160	272	528	40	37	93	-	-	-	-	-	-	-
**Xpert alone*†**	100	-	-	-	-	-	-	-	154	784	16	46	90.6	94.5	6
**WHO screen then Xpert*†**	100	-	-	-	-	-	-	-	138	784	16	62	89.6	92.7	7
	20% prevalence														
**W4SS**	200	730	261	469	231	39	35.8	85.6	-	-	-	-	-	-	-
**CRP (>=10 mg/L)**	200	530	264	266	434	36	49.8	92.3	-	-	-	-	-	-	-
**CRP (>=8 mg/L)**	200	564	270	294	406	30	47.9	93.1	-	-	-	-	-	-	-
**CRP (>=5 mg/L)**	200	640	276	364	336	24	43.1	93.3	-	-	-	-	-	-	-
**CXR (abnormal)**	200	513	219	294	406	81	42.7	83.4	-	-	-	-	-	-	-
**CXR (suggests tuberculosis)**	200	374	192	182	518	108	51.3	82.7	-	-	-	-	-	-	-
**Cough (any)**	200	415	177	238	462	123	42.7	79	-	-	-	-	-	-	-
**Cough (>=2 weeks)**	200	280	126	154	546	174	45	75.8	-	-	-	-	-	-	-
**Hb (<10 g/dL)**	200	344	162	182	518	138	47.1	79	-	-	-	-	-	-	-
**Hb (<8 g/dL)**	200	77	42	35	665	258	54.5	72	-	-	-	-	-	-	-
**BMI (<18.5 kg/m²)**	200	224	105	119	581	195	46.9	74.9	-	-	-	-	-	-	-
**Lymphadenopathy**	200	176	99	77	623	201	56.3	75.6	-	-	-	-	-	-	-
**W4SS with CRP (>=10 mg/L)¶**	200	818	279	539	161	21	34.1	88.5	-	-	-	-	-	-	-
**W4SS with CXR (abnormal)¶**	200	890	288	602	98	12	32.4	89.1	-	-	-	-	-	-	-
**W4SS then CRP (>=5 mg/L)¶**	200	478	240	238	462	60	50.2	88.5	-	-	-	-	-	-	-
**Xpert alone*†**	200	-	-	-	-	-	-	-	231	686	14	69	94.3	90.9	4
**WHO screen then Xpert*†**	200	-	-	-	-	-	-	-	207	686	14	93	93.7	88.1	5

§Estimated using the pooled point estimates for sensitivity and specificity for different tests/strategies

‡TP+FP is the number of participants who screen positive (i.e. the number who need subsequent Xpert testing)

¶For parallel strategies, two screening tests are offered at the same time. For sequential strategies, a second screening test is offered only if the first screening test is positive

*Accuracy measures for entire algorithm using total Xpert (sputum and/or non-sputum Xpert result)

		Outcome of screening§							Outcome of screening then diagnosis§						
Test	Total TB	TP+FP‡	TP	FP	TN	FN	PPV	NPV	TP	TN	FP	FN	PPV	NPV	NNS

†The test accuracy of Xpert in those who were W4SS positive was: 12 studies; 2315 participants; sensitivity 0.79 (0.7-0.86), specificity 0.97 (0.95-0.98).

Definition of abbreviations: BMI = body mass index, CRP = C-reactive protein, CXR = chest X-ray, FN= false negative, FP = false positive, Hb = haemoglobin, NNS = number needed to screen, NPV = negative predictive value, PPV = positive predictive value, TB = tuberculosis, TN = true negative, TP = true positive, W4SS = WHO four-symptom screen

Table S10E - Yield of different screening and diagnostic algorithms at different prevalences when screening a population of 1000 participants with a CD4 cell count >200 cells/µL

		Outcome of screening§							Outcome of screening then diagnosis§						
**Test**	**Total TB**	**TP+FP‡**	**TP**	**FP**	**TN**	**FN**	**PPV**	**NPV**	**TP**	**TN**	**FP**	**FN**	**PPV**	**NPV**	**NNS**
	1% prevalence														
**W4SS**	10	512	7	505	485	3	1.4	99.4	-	-	-	-	-	-	-
**CRP (>=10 mg/L)**	10	214	6	208	782	4	3	99.6	-	-	-	-	-	-	-
**CRP (>=8 mg/L)**	10	245	7	238	752	3	2.7	99.6	-	-	-	-	-	-	-
**CRP (>=5 mg/L)**	10	354	8	346	644	2	2.2	99.7	-	-	-	-	-	-	-
**CXR (abnormal)**	10	363	7	356	634	3	1.8	99.5	-	-	-	-	-	-	-
**CXR (suggests tuberculosis)**	10	194	6	188	802	4	3	99.5	-	-	-	-	-	-	-
**Cough (any)**	10	243	5	238	752	5	2.1	99.4	-	-	-	-	-	-	-
**Cough (>=2 weeks)**	10	132	3	129	861	7	2.6	99.2	-	-	-	-	-	-	-
**Hb (<10 g/dL)**	10	160	2	158	832	8	1.6	99.1	-	-	-	-	-	-	-
**Hb (<8 g/dL)**	10	41	1	40	950	9	1.5	99	-	-	-	-	-	-	-
**BMI (<18.5 kg/m²)**	10	81	2	79	911	8	2.5	99.1	-	-	-	-	-	-	-
**Lymphadenopathy**	10	102	3	99	891	7	2.9	99.2	-	-	-	-	-	-	-
**W4SS with CRP (>=10 mg/L)¶**	10	651	8	643	347	2	1.2	99.4	-	-	-	-	-	-	-
**W4SS with CXR (abnormal)¶**	10	722	9	713	277	1	1.3	99.7	-	-	-	-	-	-	-
**W4SS then CRP (>=5 mg/L)¶**	10	204	6	198	792	4	2.8	99.5	-	-	-	-	-	-	-
**Xpert alone*†**	10	-	-	-	-	-	-	-	6	980	10	4	36.5	99.6	167
**WHO screen then Xpert*†**	10	-	-	-	-	-	-	-	5	980	10	5	31.7	99.5	200
	5% prevalence														
**W4SS**	50	520	36	484	466	14	6.8	97	-	-	-	-	-	-	-
**CRP (>=10 mg/L)**	50	231	32	199	751	18	14	97.7	-	-	-	-	-	-	-
**CRP (>=8 mg/L)**	50	262	34	228	722	16	12.8	97.8	-	-	-	-	-	-	-
**CRP (>=5 mg/L)**	50	371	39	332	618	11	10.5	98.2	-	-	-	-	-	-	-
**CXR (abnormal)**	50	375	33	342	608	17	8.8	97.3	-	-	-	-	-	-	-
**CXR (suggests tuberculosis)**	50	210	30	180	770	20	14	97.4	-	-	-	-	-	-	-
**Cough (any)**	50	254	26	228	722	24	10.1	96.7	-	-	-	-	-	-	-
**Cough (>=2 weeks)**	50	141	17	124	826	33	12.1	96.2	-	-	-	-	-	-	-
**Hb (<10 g/dL)**	50	164	12	152	798	38	7.6	95.5	-	-	-	-	-	-	-
**Hb (<8 g/dL)**	50	41	3	38	912	47	7.3	95.1	-	-	-	-	-	-	-
**BMI (<18.5 kg/m²)**	50	86	10	76	874	40	11.6	95.6	-	-	-	-	-	-	-
**Lymphadenopathy**	50	110	15	95	855	35	13.6	96.1	-	-	-	-	-	-	-
**W4SS with CRP (>=10 mg/L)¶**	50	656	39	617	333	11	5.9	96.8	-	-	-	-	-	-	-
**W4SS with CXR (abnormal)¶**	50	730	46	684	266	4	6.2	98.3	-	-	-	-	-	-	-
**W4SS then CRP (>=5 mg/L)¶**	50	219	29	190	760	21	13	97.2	-	-	-	-	-	-	-
**Xpert alone*†**	50	-	-	-	-	-	-	-	29	940	10	21	75	97.8	34
**WHO screen then Xpert*†**	50	-	-	-	-	-	-	-	23	940	10	27	70.8	97.2	43
	10% prevalence														

		Outcome of screening§							Outcome of screening then diagnosis§						
**Test**	**Total TB**	**TP+FP‡**	**TP**	**FP**	**TN**	**FN**	**PPV**	**NPV**	**TP**	**TN**	**FP**	**FN**	**PPV**	**NPV**	**NNS**
**W4SS**	100	530	71	459	441	29	13.4	93.8	-	-	-	-	-	-	-
**CRP (>=10 mg/L)**	100	254	65	189	711	35	25.6	95.3	-	-	-	-	-	-	-
**CRP (>=8 mg/L)**	100	283	67	216	684	33	23.7	95.4	-	-	-	-	-	-	-
**CRP (>=5 mg/L)**	100	393	78	315	585	22	19.8	96.4	-	-	-	-	-	-	-
**CXR (abnormal)**	100	390	66	324	576	34	16.9	94.4	-	-	-	-	-	-	-
**CXR (suggests tuberculosis)**	100	230	59	171	729	41	25.7	94.7	-	-	-	-	-	-	-
**Cough (any)**	100	267	51	216	684	49	19.1	93.3	-	-	-	-	-	-	-
**Cough (>=2 weeks)**	100	151	34	117	783	66	22.5	92.2	-	-	-	-	-	-	-
**Hb (<10 g/dL)**	100	169	25	144	756	75	14.8	91	-	-	-	-	-	-	-
**Hb (<8 g/dL)**	100	42	6	36	864	94	14.3	90.2	-	-	-	-	-	-	-
**BMI (<18.5 kg/m²)**	100	92	20	72	828	80	21.7	91.2	-	-	-	-	-	-	-
**Lymphadenopathy**	100	120	30	90	810	70	25	92	-	-	-	-	-	-	-
**W4SS with CRP (>=10 mg/L)¶**	100	663	78	585	315	22	11.8	93.5	-	-	-	-	-	-	-
**W4SS with CXR (abnormal)¶**	100	739	91	648	252	9	12.3	96.6	-	-	-	-	-	-	-
**W4SS then CRP (>=5 mg/L)¶**	100	237	57	180	720	43	24.1	94.4	-	-	-	-	-	-	-
**Xpert alone*†**	100	-	-	-	-	-	-	-	57	891	9	43	86.4	95.4	18
**WHO screen then Xpert*†**	100	-	-	-	-	-	-	-	46	891	9	54	83.6	94.3	22
	20% prevalence														
**W4SS**	200	550	142	408	392	58	25.8	87.1	-	-	-	-	-	-	-
**CRP (>=10 mg/L)**	200	298	130	168	632	70	43.6	90	-	-	-	-	-	-	-
**CRP (>=8 mg/L)**	200	326	134	192	608	66	41.1	90.2	-	-	-	-	-	-	-
**CRP (>=5 mg/L)**	200	436	156	280	520	44	35.8	92.2	-	-	-	-	-	-	-
**CXR (abnormal)**	200	420	132	288	512	68	31.4	88.3	-	-	-	-	-	-	-
**CXR (suggests tuberculosis)**	200	270	118	152	648	82	43.7	88.8	-	-	-	-	-	-	-
**Cough (any)**	200	294	102	192	608	98	34.7	86.1	-	-	-	-	-	-	-
**Cough (>=2 weeks)**	200	172	68	104	696	132	39.5	84.1	-	-	-	-	-	-	-
**Hb (<10 g/dL)**	200	178	50	128	672	150	28.1	81.8	-	-	-	-	-	-	-
**Hb (<8 g/dL)**	200	44	12	32	768	188	27.3	80.3	-	-	-	-	-	-	-
**BMI (<18.5 kg/m²)**	200	104	40	64	736	160	38.5	82.1	-	-	-	-	-	-	-
**Lymphadenopathy**	200	140	60	80	720	140	42.9	83.7	-	-	-	-	-	-	-
**W4SS with CRP (>=10 mg/L)¶**	200	676	156	520	280	44	23.1	86.4	-	-	-	-	-	-	-
**W4SS with CXR (abnormal)¶**	200	758	182	576	224	18	24	92.6	-	-	-	-	-	-	-
**W4SS then CRP (>=5 mg/L)¶**	200	274	114	160	640	86	41.6	88.2	-	-	-	-	-	-	-
**Xpert alone*†**	200	-	-	-	-	-	-	-	114	792	8	86	93.4	90.2	9
**WHO screen then Xpert*†**	200	-	-	-	-	-	-	-	92	792	8	108	92	88	11

§Estimated using the pooled point estimates for sensitivity and specificity for different tests/strategies

‡TP+FP is the number of participants who screen positive (i.e. the number who need subsequent Xpert testing)

¶For parallel strategies, two screening tests are offered at the same time. For sequential strategies, a second screening test is offered only if the first screening test is positive

*Accuracy measures for entire algorithm using total Xpert (sputum and/or non-sputum Xpert result)

		Outcome of screening§							Outcome of screening then diagnosis§						
Test	Total TB	TP+FP‡	TP	FP	TN	FN	PPV	NPV	TP	TN	FP	FN	PPV	NPV	NNS

†The test accuracy of Xpert in those who were W4SS positive was: 12 studies; 2121 participants; sensitivity 0.64 (0.54-0.74), specificity 0.99 (0.98-0.99).

Definition of abbreviations: BMI = body mass index, CRP = C-reactive protein, CXR = chest X-ray, FN= false negative, FP = false positive, Hb = haemoglobin, NNS = number needed to screen, NPV = negative predictive value, PPV = positive predictive value, TB = tuberculosis, TN = true negative, TP = true positive, W4SS = WHO four-symptom screen

Table S10F - Yield of different screening and diagnostic algorithms at different prevalences when screening a population of 1000 pregnant participants#

		Outcome of screening§							Outcome of screening then diagnosis§						
**Test**	**Total TB**	**TP+FP‡**	**TP**	**FP**	**TN**	**FN**	**PPV**	**NPV**	**TP**	**TN**	**FP**	**FN**	**PPV**	**NPV**	**NNS**
	1% prevalence														
**W4SS**	10	424	8	416	574	2	2	99.7	-	-	-	-	-	-	-
**CRP (>=10 mg/L)**	10	-	-	-	-	-	-	-	-	-	-	-	-	-	-
**CRP (>=8 mg/L)**	10	-	-	-	-	-	-	-	-	-	-	-	-	-	-
**CRP (>=5 mg/L)**	10	-	-	-	-	-	-	-	-	-	-	-	-	-	-
**CXR (abnormal)**	10	315	8	307	683	2	2.4	99.6	-	-	-	-	-	-	-
**CXR (suggests tuberculosis)**	10	77	8	69	921	2	9.8	99.7	-	-	-	-	-	-	-
**Cough (any)**	10	195	7	188	802	3	3.4	99.6	-	-	-	-	-	-	-
**Cough (>=2 weeks)**	10	84	5	79	911	5	5.6	99.4	-	-	-	-	-	-	-
**Hb (<10 g/dL)**	10	250	2	248	742	8	0.8	98.9	-	-	-	-	-	-	-
**Hb (<8 g/dL)**	10	20	0	20	970	10	0	99	-	-	-	-	-	-	-
**BMI (<18.5 kg/m²)**	10	40	0	40	950	10	0	99	-	-	-	-	-	-	-
**Lymphadenopathy**	10	-	-	-	-	-	-	-	-	-	-	-	-	-	-
**W4SS with CRP (>=10 mg/L)¶**	10	-	-	-	-	-	-	-	-	-	-	-	-	-	-
**W4SS with CXR (abnormal)¶**	10	444	8	436	554	2	1.7	99.6	-	-	-	-	-	-	-
**W4SS then CRP (>=5 mg/L)¶**	10	-	-	-	-	-	-	-	-	-	-	-	-	-	-
**Xpert alone*†**	10	-	-	-	-	-	-	-	5	980	10	5	34.9	99.5	200
**WHO screen then Xpert*†**	10	-	-	-	-	-	-	-	4	990	0	6	100	99.4	250
	5% prevalence														
**W4SS**	50	441	42	399	551	8	9.5	98.6	-	-	-	-	-	-	-
**CRP (>=10 mg/L)**	50	-	-	-	-	-	-	-	-	-	-	-	-	-	-
**CRP (>=8 mg/L)**	50	-	-	-	-	-	-	-	-	-	-	-	-	-	-
**CRP (>=5 mg/L)**	50	-	-	-	-	-	-	-	-	-	-	-	-	-	-
**CXR (abnormal)**	50	332	38	294	656	12	11.3	98.1	-	-	-	-	-	-	-
**CXR (suggests tuberculosis)**	50	104	38	66	884	12	36.1	98.6	-	-	-	-	-	-	-
**Cough (any)**	50	214	34	180	770	16	15.7	97.9	-	-	-	-	-	-	-
**Cough (>=2 weeks)**	50	100	24	76	874	26	23.6	97.1	-	-	-	-	-	-	-
**Hb (<10 g/dL)**	50	248	10	238	712	40	4	94.7	-	-	-	-	-	-	-
**Hb (<8 g/dL)**	50	19	0	19	931	50	0	94.9	-	-	-	-	-	-	-
**BMI (<18.5 kg/m²)**	50	38	0	38	912	50	0	94.8	-	-	-	-	-	-	-
**Lymphadenopathy**	50	-	-	-	-	-	-	-	-	-	-	-	-	-	-
**W4SS with CRP (>=10 mg/L)¶**	50	-	-	-	-	-	-	-	-	-	-	-	-	-	-
**W4SS with CXR (abnormal)¶**	50	456	38	418	532	12	8.2	97.7	-	-	-	-	-	-	-
**W4SS then CRP (>=5 mg/L)¶**	50	-	-	-	-	-	-	-	-	-	-	-	-	-	-
**Xpert alone*†**	50	-	-	-	-	-	-	-	26	940	10	24	73.6	97.6	38
**WHO screen then Xpert*†**	50	-	-	-	-	-	-	-	18	950	0	32	100	96.7	56
	10% prevalence														

		Outcome of screening§							Outcome of screening then diagnosis§						
**Test**	**Total TB**	**TP+FP‡**	**TP**	**FP**	**TN**	**FN**	**PPV**	**NPV**	**TP**	**TN**	**FP**	**FN**	**PPV**	**NPV**	**NNS**
**W4SS**	100	462	84	378	522	16	18.2	97	-	-	-	-	-	-	-
**CRP (>=10 mg/L)**	100	-	-	-	-	-	-	-	-	-	-	-	-	-	-
**CRP (>=8 mg/L)**	100	-	-	-	-	-	-	-	-	-	-	-	-	-	-
**CRP (>=5 mg/L)**	100	-	-	-	-	-	-	-	-	-	-	-	-	-	-
**CXR (abnormal)**	100	354	75	279	621	25	21.2	96.1	-	-	-	-	-	-	-
**CXR (suggests tuberculosis)**	100	138	75	63	837	25	54.3	97.1	-	-	-	-	-	-	-
**Cough (any)**	100	238	67	171	729	33	28.2	95.7	-	-	-	-	-	-	-
**Cough (>=2 weeks)**	100	119	47	72	828	53	39.5	94	-	-	-	-	-	-	-
**Hb (<10 g/dL)**	100	245	20	225	675	80	8.2	89.4	-	-	-	-	-	-	-
**Hb (<8 g/dL)**	100	18	0	18	882	100	0	89.8	-	-	-	-	-	-	-
**BMI (<18.5 kg/m²)**	100	36	0	36	864	100	0	89.6	-	-	-	-	-	-	-
**Lymphadenopathy**	100	-	-	-	-	-	-	-	-	-	-	-	-	-	-
**W4SS with CRP (>=10 mg/L)¶**	100	-	-	-	-	-	-	-	-	-	-	-	-	-	-
**W4SS with CXR (abnormal)¶**	100	471	75	396	504	25	15.9	95.3	-	-	-	-	-	-	-
**W4SS then CRP (>=5 mg/L)¶**	100	-	-	-	-	-	-	-	-	-	-	-	-	-	-
**Xpert alone*†**	100	-	-	-	-	-	-	-	53	891	9	47	85.5	95	19
**WHO screen then Xpert*†**	100	-	-	-	-	-	-	-	36	900	0	64	100	93.4	28
	20% prevalence														
**W4SS**	200	504	168	336	464	32	33.3	93.5	-	-	-	-	-	-	-
**CRP (>=10 mg/L)**	200	-	-	-	-	-	-	-	-	-	-	-	-	-	-
**CRP (>=8 mg/L)**	200	-	-	-	-	-	-	-	-	-	-	-	-	-	-
**CRP (>=5 mg/L)**	200	-	-	-	-	-	-	-	-	-	-	-	-	-	-
**CXR (abnormal)**	200	398	150	248	552	50	37.7	91.7	-	-	-	-	-	-	-
**CXR (suggests tuberculosis)**	200	206	150	56	744	50	72.8	93.7	-	-	-	-	-	-	-
**Cough (any)**	200	286	134	152	648	66	46.9	90.8	-	-	-	-	-	-	-
**Cough (>=2 weeks)**	200	158	94	64	736	106	59.5	87.4	-	-	-	-	-	-	-
**Hb (<10 g/dL)**	200	240	40	200	600	160	16.7	78.9	-	-	-	-	-	-	-
**Hb (<8 g/dL)**	200	16	0	16	784	200	0	79.7	-	-	-	-	-	-	-
**BMI (<18.5 kg/m²)**	200	32	0	32	768	200	0	79.3	-	-	-	-	-	-	-
**Lymphadenopathy**	200	-	-	-	-	-	-	-	-	-	-	-	-	-	-
**W4SS with CRP (>=10 mg/L)¶**	200	-	-	-	-	-	-	-	-	-	-	-	-	-	-
**W4SS with CXR (abnormal)¶**	200	502	150	352	448	50	29.9	90	-	-	-	-	-	-	-
**W4SS then CRP (>=5 mg/L)¶**	200	-	-	-	-	-	-	-	-	-	-	-	-	-	-
**Xpert alone*†**	200	-	-	-	-	-	-	-	106	792	8	94	93	89.4	9
**WHO screen then Xpert*†**	200	-	-	-	-	-	-	-	72	800	0	128	100	86.2	14

#For lymphadenopathy and strategies containing CRP, there were insufficient data to perform meta-analysis

§Estimated using the pooled point estimates for sensitivity and specificity for different tests/strategies

‡TP+FP is the number of participants who screen positive (i.e. the number who need subsequent Xpert testing)

¶For parallel strategies, two screening tests are offered at the same time. For sequential strategies, a second screening test is offered only if the first screening test is positive

		Outcome of screening§						Outcome of screening then diagnosis§						
Test	Total TB	TP+FP‡	TP	FP	TN	FN	PPV	NPV	TP	TN	FP	FN	PPV	NPV	NNS

*Accuracy measures for entire algorithm using total Xpert (sputum and/or non-sputum Xpert result)

†The test accuracy of Xpert in those who were W4SS positive was: 5 studies; 137 participants; sensitivity 0.71 (0.33-0.93), specificity 1 (0-1).

Definition of abbreviations: BMI = body mass index, CRP = C-reactive protein, CXR = chest X-ray, FN= false negative, FP = false positive, Hb = haemoglobin, NNS = number needed to screen, NPV = negative predictive value, PPV = positive predictive value, TB = tuberculosis, TN = true negative, TP = true positive, W4SS = WHO four-symptom screen

Table S11 - Sensitivity analyses using different reference standards

Table S11A - Sensitivity analyses using different reference standards in all participants

	Sensitivity analyses 1*				Sensitivity analyses 2†			
	No of studies	N	Sensitivity (95% CI)	Specificity (95% CI)	No of studies	N	Sensitivity (95% CI)	Specificity (95% CI)
**W4SS**	21	15652	82 (71-89)	42 (29-57)	12	8612	88 (76-94)	43 (27-61)
**CRP (>=10 mg/L)**	5	3582	73 (49-88)	74 (62-83)	4	3110	77 (35-95)	72 (56-84)
**CRP (>=8 mg/L)**	5	3582	77 (58-89)	70 (57-81)	4	3110	78 (31-96)	69 (52-82)
**CRP (>=5 mg/L)**	5	3582	85 (72-93)	60 (48-71)	4	3110	88 (62-97)	59 (43-72)
**CXR (abnormal)**	8	6222	72 (65-78)	62 (52-71)	4	4190	73 (65-79)	65 (55-74)
**CXR (suggests tuberculosis)**	8	6177	63 (56-70)	78 (67-86)	3	3749	64 (52-74)	84 (68-93)
**Cough (any)**	21	15623	56 (48-63)	73 (65-79)	12	8586	63 (55-71)	73 (64-80)
**Cough (>=2 weeks)**	17	10919	38 (28-49)	84 (77-90)	9	4545	38 (21-58)	86 (76-92)
**Hb (<10 g/dL)**	9	5118	43 (33-53)	80 (73-85)	5	2098	57 (41-72)	76 (63-86)
**Hb (<8 g/dL)**	9	5118	12 (9-15)	96 (93-97)	5	2098	13 (9-19)	94 (89-97)
**BMI (<18.5 kg/m²)**	18	12704	29 (21-37)	89 (84-92)	12	8464	38 (26-51)	87 (80-91)
**Lymphadenopathy**	4	2394	30 (14-54)	91 (75-97)	3	1404	23 (13-38)	94 (84-98)
**W4SS with CRP (>=10 mg/L)¶**	5	3582	87 (56-97)	31 (13-56)	4	3110	94 (46-100)	30 (10-63)
**W4SS with CXR (abnormal)¶**	8	6213	95 (90-97)	21 (10-38)	4	4182	95 (87-99)	31 (15-55)
**W4SS then CRP (>=5 mg/L)¶**	5	3582	66 (25-92)	75 (54-88)	4	3110	72 (24-96)	75 (47-91)

*Diagnostic accuracy estimates using culture or Xpert of sputum and/or other specimens as a reference standard

†Diagnostic accuracy estimates using only Xpert of sputum and/or other specimens as a reference standard

¶For parallel strategies, two screening tests are offered at the same time. For sequential strategies, a second screening test is offered only if the first screening test is positive

Definition of abbreviations: BMI = body mass index, CRP = C-reactive protein, CXR = chest X-ray, Hb = haemoglobin, W4SS = WHO four-symptom screen

Table S11B - Sensitivity analyses using different reference standards in outpatients (on ART)

	**Sensitivity analyses 1***				**Sensitivity analyses 2†**			
	**No of studies**	**N**	**Sensitivity (95% CI)**	**Specificity (95% CI)**	**No of studies**	**N**	**Sensitivity (95% CI)**	**Specificity (95% CI)**
**W4SS**	9	4328	56 (32-77)	71 (51-85)	4	2664	51 (20-82)	80 (67-88)
**CRP (>=10 mg/L)**	1	381	12 (2-54)	90 (93-87)	1	378	10 (1-67)	90 (93-87)
**CRP (>=8 mg/L)**	1	381	25 (6-62)	89 (92-85)	1	378	10 (1-67)	88 (91-85)
**CRP (>=5 mg/L)**	1	381	38 (13-72)	80 (84-76)	1	378	25 (3-76)	79 (83-75)
**CXR (abnormal)**	4	2679	72 (61-82)	64 (50-75)	2	2063	69 (56-80)	72 (64-79)
**CXR (suggests tuberculosis)**	4	2590	69 (52-82)	78 (62-89)	1	1745	55 (40-69)	92 (93-91)
**Cough (any)**	9	4328	41 (22-62)	83 (73-90)	4	2664	43 (29-58)	88 (85-91)
**Cough (>=2 weeks)**	6	1746	18 (4-51)	93 (79-98)	2	536	13 (0-86)	97 (94-98)
**Hb (<10 g/dL)**	4	844	49 (30-68)	83 (71-90)	2	629	18 (0-93)	77 (61-88)
**Hb (<8 g/dL)**	4	844	13 (4-31)	94 (91-96)	2	629	10 (2-37)	93 (90-94)
**BMI (<18.5 kg/m²)**	7	4054	17 (8-32)	93 (88-96)	4	2646	14 (1-76)	89 (82-94)
**Lymphadenopathy**	1	338	17 (7-38)	93 (95-90)	1	338	11 (3-34)	92 (95-89)
**W4SS with CRP (>=10 mg/L)¶**	1	381	12 (2-54)	79 (83-75)	1	378	10 (1-67)	79 (83-75)
**W4SS with CXR (abnormal)¶**	4	2679	90 (73-97)	33 (17-55)	2	2063	87 (62-97)	51 (33-70)
**W4SS then CRP (>=5 mg/L)¶**	1	381	6 (0-50)	96 (97-93)	1	378	10 (1-67)	96 (97-93)

*Diagnostic accuracy estimates using culture or Xpert of sputum and/or other specimens as a reference standard

†Diagnostic accuracy estimates using only Xpert of sputum and/or other specimens as a reference standard

¶For parallel strategies, two screening tests are offered at the same time. For sequential strategies, a second screening test is offered only if the first screening test is positive

Definition of abbreviations: BMI = body mass index, CRP = C-reactive protein, CXR = chest X-ray, Hb = haemoglobin, W4SS = WHO four-symptom screen

Table S11C - Sensitivity analyses using different reference standards in outpatients (not on ART)

	**Sensitivity analyses 1***				**Sensitivity analyses 2†**			
	**No of studies**	**N**	**Sensitivity (95% CI)**	**Specificity (95% CI)**	**No of studies**	**N**	**Sensitivity (95% CI)**	**Specificity (95% CI)**
**W4SS**	20	11196	85 (76-91)	37 (26-51)	11	5820	90 (80-96)	37 (24-53)
**CRP (>=10 mg/L)**	5	3198	82 (77-86)	67 (61-73)	4	2729	89 (85-92)	63 (56-70)
**CRP (>=8 mg/L)**	5	3198	84 (81-87)	64 (57-70)	4	2729	90 (86-94)	59 (51-67)
**CRP (>=5 mg/L)**	5	3198	89 (85-91)	54 (46-61)	4	2729	93 (89-96)	50 (42-59)
**CXR (abnormal)**	8	3543	72 (65-78)	62 (52-72)	4	2127	73 (64-80)	66 (54-76)
**CXR (suggests tuberculosis)**	8	3587	62 (55-69)	79 (67-87)	3	2004	64 (52-74)	85 (67-94)
**Cough (any)**	20	11167	58 (51-65)	70 (63-76)	11	5794	66 (59-73)	69 (62-75)
**Cough (>=2 weeks)**	16	9045	43 (34-52)	82 (75-87)	8	3881	48 (33-63)	81 (72-87)
**Hb (<10 g/dL)**	9	4271	44 (33-56)	79 (71-85)	5	1466	62 (43-78)	74 (58-85)
**Hb (<8 g/dL)**	9	4271	11 (8-15)	96 (93-98)	5	1466	14 (10-20)	94 (87-97)
**BMI (<18.5 kg/m²)**	17	8522	31 (23-40)	87 (82-92)	11	5690	42 (29-56)	85 (76-91)
**Lymphadenopathy**	4	2056	30 (13-54)	91 (76-97)	3	1066	25 (13-43)	95 (86-98)
**W4SS with CRP (>=10 mg/L)¶**	5	3198	94 (86-98)	20 (12-33)	4	2729	98 (93-100)	17 (9-30)
**W4SS with CXR (abnormal)¶**	8	3534	95 (92-98)	18 (9-34)	4	2119	96 (91-99)	29 (15-50)
**W4SS then CRP (>=5 mg/L)¶**	5	3198	83 (74-89)	64 (57-70)	4	2729	89 (84-92)	60 (52-68)

*Diagnostic accuracy estimates using culture or Xpert of sputum and/or other specimens as a reference standard

†Diagnostic accuracy estimates using only Xpert of sputum and/or other specimens as a reference standard

¶For parallel strategies, two screening tests are offered at the same time. For sequential strategies, a second screening test is offered only if the first screening test is positive

Definition of abbreviations: BMI = body mass index, CRP = C-reactive protein, CXR = chest X-ray, Hb = haemoglobin, W4SS = WHO four-symptom screen

Table S11D - Sensitivity analyses using different reference standards in participants with CD4 cell count <=200 cells/µL

	Sensitivity analyses 1*				Sensitivity analyses 2†			
	No of studies	N	Sensitivity (95% CI)	Specificity (95% CI)	No of studies	N	Sensitivity (95% CI)	Specificity (95% CI)
W4SS	21	5636	88 (78-93)	33 (20-49)	12	3129	90 (78-96)	35 (19-55)
CRP (>=10 mg/L)	5	1597	88 (82-92)	63 (50-73)	4	1460	91 (84-95)	62 (48-75)
CRP (>=8 mg/L)	5	1597	90 (86-93)	59 (46-71)	4	1460	93 (86-97)	58 (43-72)
CRP (>=5 mg/L)	5	1597	92 (88-95)	48 (34-63)	4	1460	94 (88-97)	49 (33-65)
CXR (abnormal)	8	2210	74 (65-80)	58 (49-67)	4	1311	72 (62-80)	61 (49-71)
CXR (suggests tuberculosis)	8	2143	64 (56-71)	74 (64-82)	3	1157	61 (49-71)	81 (63-91)
Cough (any)	21	5626	59 (52-66)	67 (59-74)	12	3119	65 (56-73)	67 (58-75)
Cough (>=2 weeks)	17	4203	42 (33-50)	78 (70-85)	9	1889	38 (22-58)	80 (69-88)
Hb (<10 g/dL)	9	1971	53 (42-63)	74 (65-81)	5	916	65 (51-77)	71 (53-84)
Hb (<8 g/dL)	9	1971	14 (10-19)	95 (91-97)	5	916	15 (9-25)	93 (86-96)
BMI (<18.5 kg/m²)	18	4969	34 (26-43)	84 (77-88)	12	3098	42 (31-54)	80 (72-86)
Lymphadenopathy	4	1003	31 (13-56)	89 (73-96)	3	627	21 (11-35)	94 (87-97)
W4SS with CRP (>=10 mg/L)¶	5	1597	92 (52-99)	23 (10-47)	4	1460	97 (76-100)	24 (8-55)
W4SS with CXR (abnormal)¶	8	2208	97 (92-99)	14 (7-25)	4	1309	97 (88-99)	21 (11-36)
W4SS then CRP (>=5 mg/L)¶	5	1597	80 (51-94)	66 (37-86)	4	1460	95 (66-99)	69 (35-90)

*Diagnostic accuracy estimates using culture or Xpert of sputum and/or other specimens as a reference standard

†Diagnostic accuracy estimates using only Xpert of sputum and/or other specimens as a reference standard

¶For parallel strategies, two screening tests are offered at the same time. For sequential strategies, a second screening test is offered only if the first screening test is positive

Definition of abbreviations: BMI = body mass index, CRP = C-reactive protein, CXR = chest X-ray, Hb = haemoglobin, W4SS = WHO four-symptom screen

Table S11E - Sensitivity analyses using different reference standards in participants with CD4 cell count >200 cells/µL

	Sensitivity analyses 1*				Sensitivity analyses 2†			
	No of studies	N	Sensitivity (95% CI)	Specificity (95% CI)	No of studies	N	Sensitivity (95% CI)	Specificity (95% CI)
**W4SS**	21	9633	71 (58-81)	50 (36-63)	12	5163	81 (63-91)	50 (33-67)
**CRP (>=10 mg/L)**	5	1829	61 (45-75)	79 (68-87)	4	1515	72 (45-89)	78 (62-88)
**CRP (>=8 mg/L)##**	5	1829	64 (47-78)	76 (64-85)	4	1515	78 (67-87)	74 (58-86)
**CRP (>=5 mg/L)**	5	1829	75 (59-87)	65 (54-75)	4	1515	86 (58-97)	64 (50-77)
**CXR (abnormal)**	8	3909	66 (59-73)	65 (53-74)	4	2784	73 (60-83)	67 (56-76)
**CXR (suggests tuberculosis)**	8	3932	60 (51-69)	81 (70-89)	3	2498	66 (46-82)	86 (69-94)
**Cough (any)**	21	9616	50 (41-60)	76 (68-82)	12	5149	61 (48-71)	76 (67-83)
**Cough (>=2 weeks)**	17	6416	34 (22-48)	87 (80-91)	9	2415	38 (16-65)	88 (80-93)
**Hb (<10 g/dL)**	9	3048	27 (18-38)	84 (79-89)	5	1112	38 (20-60)	82 (73-88)
**Hb (<8 g/dL)**	9	3048	6 (2-13)	97 (94-98)	5	1112	7 (2-25)	95 (91-97)
**BMI (<18.5 kg/m²)**	18	7396	20 (13-30)	92 (88-95)	12	5063	32 (20-47)	91 (85-94)
**Lymphadenopathy**	4	1374	31 (12-59)	91 (73-97)	3	770	27 (12-49)	94 (77-99)
**W4SS with CRP (>=10 mg/L)¶**	5	1829	76 (47-92)	35 (15-61)	4	1515	93 (4-100)	34 (12-66)
**W4SS with CXR (abnormal)¶**	8	3903	91 (82-96)	28 (16-44)	4	2778	93 (75-98)	35 (15-63)
**W4SS then CRP (>=5 mg/L)¶**	5	1829	53 (12-90)	80 (62-91)	4	1515	56 (5-97)	79 (55-92)

*Diagnostic accuracy estimates using culture or Xpert of sputum and/or other specimens as a reference standard

†Diagnostic accuracy estimates using only Xpert of sputum and/or other specimens as a reference standard

##For sensitivity analyses 2, the bivariate model did not converge; results from model assuming no correlation between sensitivity and specificity

¶For parallel strategies, two screening tests are offered at the same time. For sequential strategies, a second screening test is offered only if the first screening test is positive

Definition of abbreviations: BMI = body mass index, CRP = C-reactive protein, CXR = chest X-ray, Hb = haemoglobin, W4SS = WHO four-symptom screen

Table S11F - Sensitivity analyses using different reference standards in pregnant participants§

	Sensitivity analyses 1*				Sensitivity analyses 2†			
	No of studies	N	Sensitivity (95% CI)	Specificity (95% CI)	No of studies	N	Sensitivity (95% CI)	Specificity (95% CI)
**W4SS**	8	1935	77 (25-97)	58 (39-75)	5	489	71 (10-98)	55 (31-77)
**CRP (>=10 mg/L)#**	-	-	-	-	-	-	-	-
**CRP (>=8 mg/L)#**	-	-	-	-	-	-	-	-
**CRP (>=5 mg/L)#**	-	-	-	-	-	-	-	-
**CXR (abnormal)**	1	8	75 (11-99)	69 (91-33)	1	8	75 (11-99)	69 (91-33)
**CXR (suggests tuberculosis)**	1	7	75 (11-99)	93 (100-42)	1	7	75 (11-99)	93 (100-42)
**Cough (any)**	8	1933	62 (24-89)	81 (70-88)	5	487	53 (12-90)	81 (70-89)
**Cough (>=2 weeks)**	8	1933	43 (17-74)	92 (86-95)	5	488	17 (3-61)	92 (87-96)
**Hb (<10 g/dL)#**	5	1350	20 (10-36)	75 (61-85)	-	-	-	-
**Hb (<8 g/dL)#**	5	1350	0 (0-100)	98 (97-99)	-	-	-	-
**BMI (<18.5 kg/m²)##**	7	472	0 (0-98)	96 (94-98)	5	431	0 (0-100)	97 (94-98)
**Lymphadenopathy#**	-	-	-	-	-	-	-	-
**W4SS with CRP (>=10 mg/L)¶#**	-	-	-	-	-	-	-	-
**W4SS with CXR (abnormal)¶**	1	8	75 (11-99)	56 (84-24)	1	8	75 (11-99)	56 (84-24)
**W4SS then CRP (>=5 mg/L)¶#**	-	-	-	-	-	-	-	-

§For some analyses, all studies had 0% or 100% sensitivity/specificity; therefore, models may have given unreliable estimates such as 95% CIs that range from 0 to 100

*Diagnostic accuracy estimates using culture or Xpert of sputum and/or other specimens as a reference standard

†Diagnostic accuracy estimates using only Xpert of sputum and/or other specimens as a reference standard

#Insufficient data to perform meta-analysis

##Bivariate model did not converge; results from model assuming no correlation between sensitivity and specificity

¶For parallel strategies, two screening tests are offered at the same time. For sequential strategies, a second screening test is offered only if the first screening test is positive

Definition of abbreviations: BMI = body mass index, CRP = C-reactive protein, CXR = chest X-ray, Hb = haemoglobin, W4SS = WHO four-symptom screen

Table S12 - Sensitivity analyses comparing W4SS followed by Xpert with CRP (≥10mg) followed by Xpert in all participants and by subgroups†

									Number of Xpert tests per 1000 PLHIV*							
	CRP (>=10 mg/L) then Xpert				W4SS then Xpert				1% prevalence		5% prevalence		10% prevalence		20% prevalence	
**Subgroup**	**No of studies**	**N**	**Sensitivity (95% CI)**	**Specificity (95% CI)**	**No of studies**	**N**	**Sensitivity (95% CI)**	**Specificity (95% CI)**	**W4SS**	**CRP**	**W4SS**	**CRP**	**W4SS**	**CRP**	**W4SS**	**CRP**
**All participants**	4	3099	54 (45-63)	100 (99-100)	4	3099	50 (28-71)	100 (98-100)	612	265	620	286	631	311	652	362
**Outpatients (Not on ART)**	4	2718	56 (51-60)	99 (99-100)	4	2718	59 (51-66)	99 (99-100)	732	345	738	365	747	390	764	440
**Outpatients (On ART)**	1	378	8 (1-62)	100 (100-98)	1	378	8 (1-62)	100 (100-98)	120	101	118	105	116	110	112	120
**CD4 >200 cells/μL**	4	1508	43 (34-53)	100 (7-100)	4	1508	44 (32-58)	100 (99-100)	572	214	578	230	585	251	600	292
**CD4 <=200 cells/μL**	4	1456	61 (51-71)	99 (98-100)	4	1456	63 (53-71)	99 (98-99)	682	365	689	386	698	411	716	462
**Pregnant participants#**	-	-	-	-	-	-	-	-	-	-	-	-	-	-	-	-

†Using culture as a reference standard; accuracy measures for entire algorithm using total Xpert (sputum and/or non-sputum Xpert result)

*Calculated using direct comparison estimates

#Insufficient data to perform meta-analysis

Definition of abbreviations: ART = antiretroviral therapy, CRP = C-reactive protein, PLHIV = people living with HIV, W4SS = WHO four-symptom screen

Figure S1 - Risk of bias and applicability results on the QUADAS-2 criteria tool

Figure S1A - Risk of bias and applicability results on the QUADAS-2 criteria tool by study†

Study	Risk of bias domains D1	D2	D3	D4	D5	D6	D7
Abed Al-Darraji, 2013	+	+	+	+	+	+	+
Affolabi, 2018	+	+	+	+	+	+	+
Ahmad, 2014	+	+	+	+	+	+	+
Balcha, 2014	+	+	+	+	X	+	+
Bjerrum, 2015	+	+	+	+	X	+	+
Gersh, 2018	+	+	+	+	+	+	+
Hanifa, 2012	+	+	+	+	X	+	+
Hoffmann, 2013	+	+	+	+	X	+	+
Kempker, 2019	+	+	+	+	+	+	+
Kerkhoff, 2013	+	+	+	+	+	+	+
Kufa, 2012	+	+	+	+	+	+	+
LaCourse, 2016	+	+	+	+	X	+	+
Mbu, 2018	+	+	+	+	+	+	+
Modi, 2016¶	+	+	+	+	+	+	+
Nguyen, 2016	+	+	+	+	+	+	+
Rangaka, 2012	+	+	+	X	+	+	+
Reeve, unpublished	+	+	+	+	+	+	+
Shapiro, 2018	+	+	+	+	+	+	+
Swindells, 2013	+	+	+	+	+	+	+
Thit, 2017§	+	+	+	+	+	+	+
Yoon, 2018	+	+	+	+	X	+	+

D1: Patient selection (Risk of Bias)
D2: Index test (Risk of Bias)
D3: Reference test (Risk of Bias)
D4: Flow and timing (Risk of Bias)
D5: Patient selection (Applicability)
D6: Index test (Applicability)
D7: Reference test (Applicability)

†In general, domains were assessed for all participants. Green = low risk of bias, red = high risk of bias
¶For the domain D4 (flow and timing), the risk of bias was judged high for chest X-ray as the index test (>20% missing data).
§For the domain D4 (flow and timing), the risk of bias was judged high for haemoglobin as the index test (>20% missing data).

Figure S1B - Clustered bar graphs of risk of bias and applicability results on the QUADAS-2 criteria tool

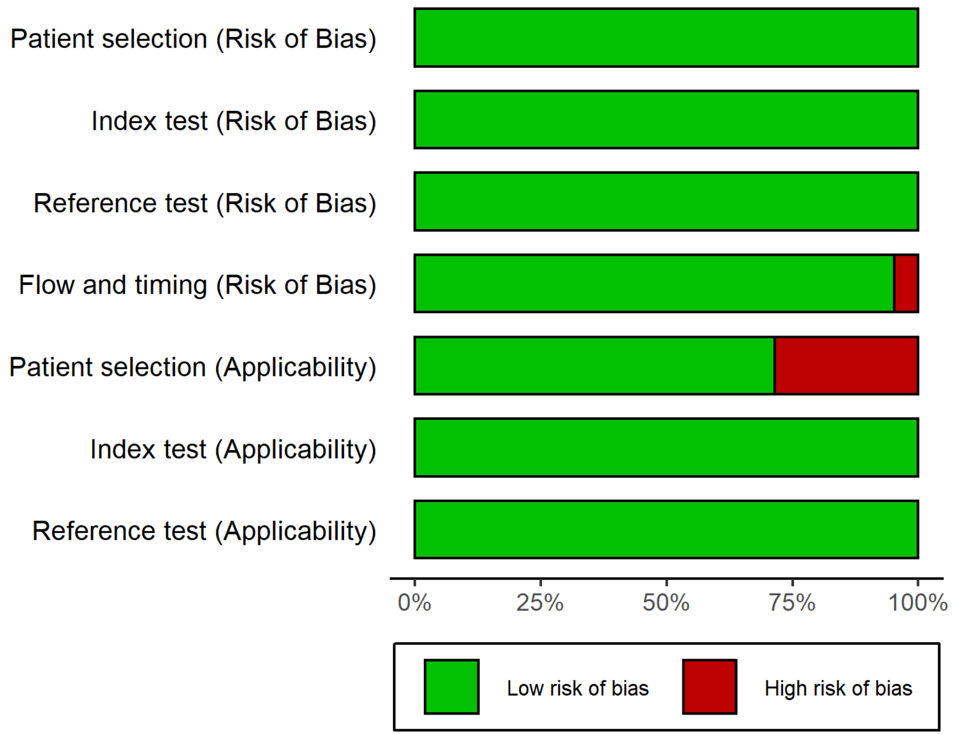

Figure S2 - Plots of sensitivity and specificity for each screening test for the detection of tuberculosis

Figure S2A - Plots of sensitivity and specificity for each screening test for the detection of tuberculosis in all participants†

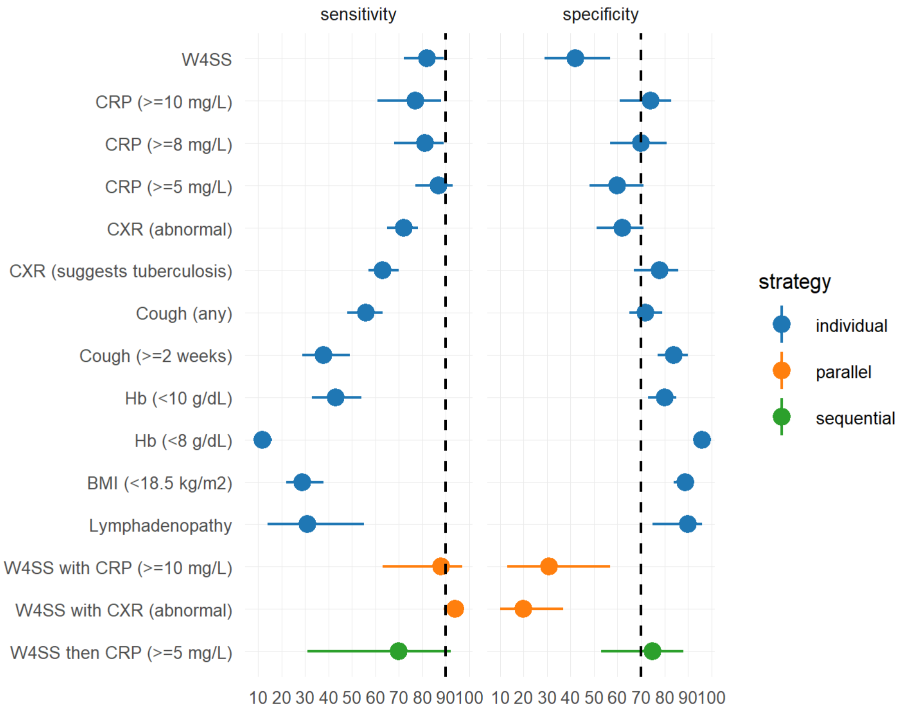

†Dashed lines indicate WHO's minimum requirements for a tuberculosis screening test (90% sensitivity and 70% specificity)

Definition of abbreviations: BMI = body mass index, CRP = C-reactive protein, CXR = chest X-ray, Hb = haemoglobin, W4SS = WHO four-symptom screen

Figure S2B - Plots of sensitivity and specificity for each screening test for the detection of tuberculosis in outpatients (on ART)†

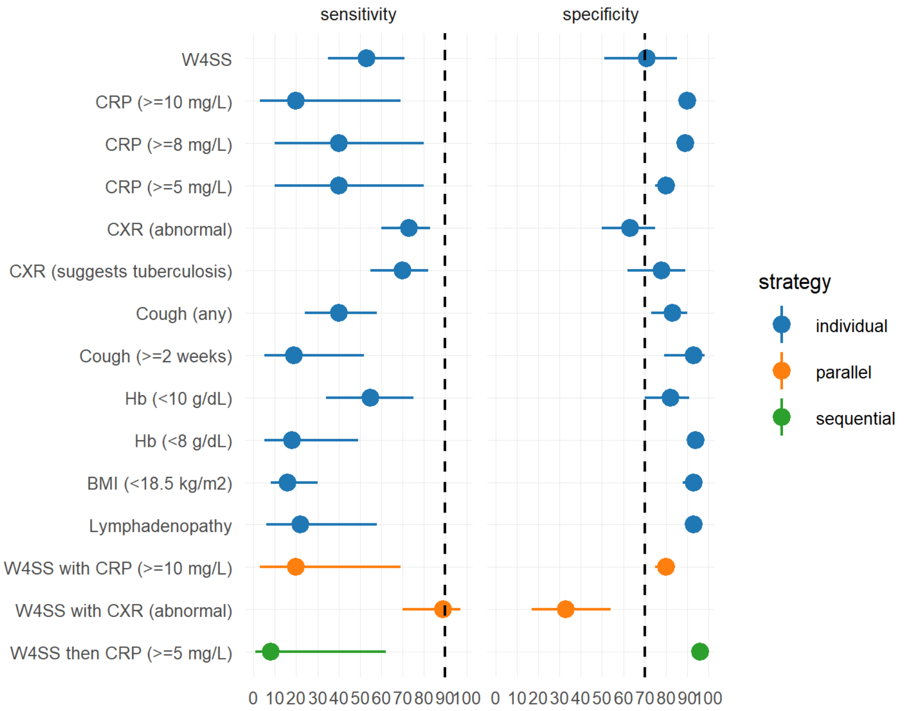

†Dashed lines indicate WHO's minimum requirements for a tuberculosis screening test (90% sensitivity and 70% specificity)
Definition of abbreviations: ART = antiretroviral therapy, BMI = body mass index, CRP = C-reactive protein, CXR = chest X-ray, Hb = haemoglobin, W4SS = WHO four-symptom screen

Figure S2C - Plots of sensitivity and specificity for each screening test for the detection of tuberculosis in outpatients (not on ART)†

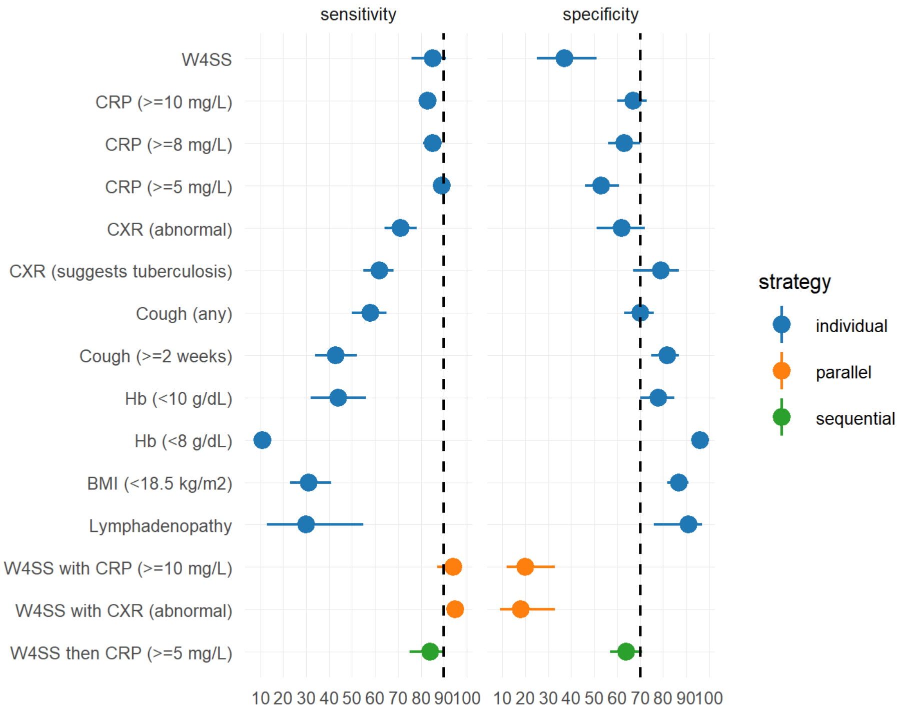

†Dashed lines indicate WHO's minimum requirements for a tuberculosis screening test (90% sensitivity and 70% specificity)
Definition of abbreviations: ART = antiretroviral therapy, BMI = body mass index, CRP = C-reactive protein, CXR = chest X-ray, Hb = haemoglobin, W4SS = WHO four-symptom screen

Figure S2D - Plots of sensitivity and specificity for each screening test for the detection of tuberculosis in participants with CD4 cell count <=200 cells/μL†

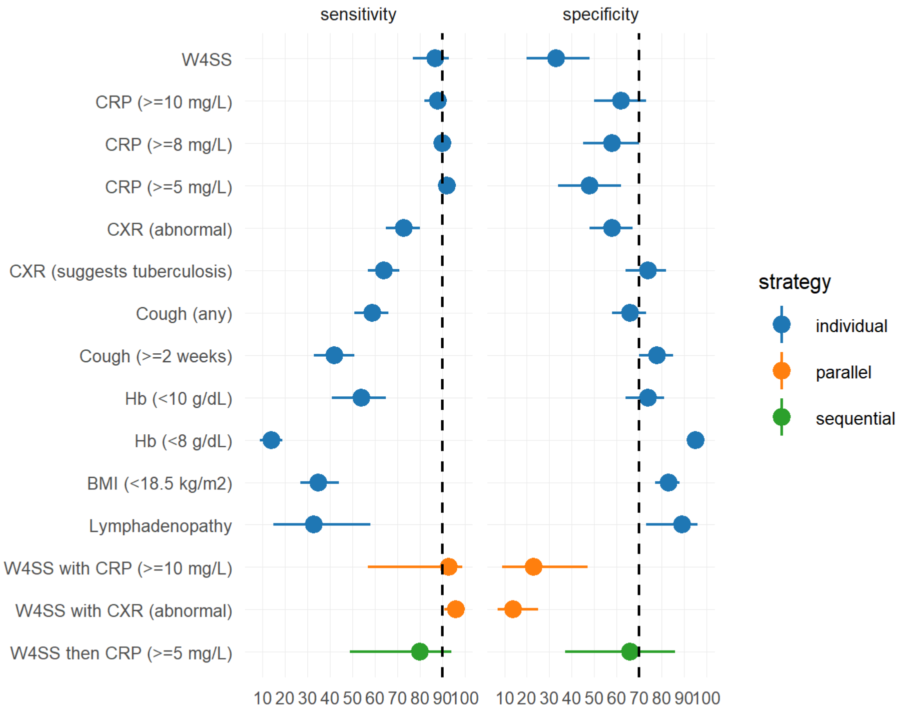

†Dashed lines indicate WHO's minimum requirements for a tuberculosis screening test (90% sensitivity and 70% specificity)
Definition of abbreviations: BMI = body mass index, CRP = C-reactive protein, CXR = chest X-ray, Hb = haemoglobin, W4SS = WHO four-symptom screen

Figure S2E - Plots of sensitivity and specificity for each screening test for the detection of tuberculosis in participants with CD4 cell count >200 cells/μL†

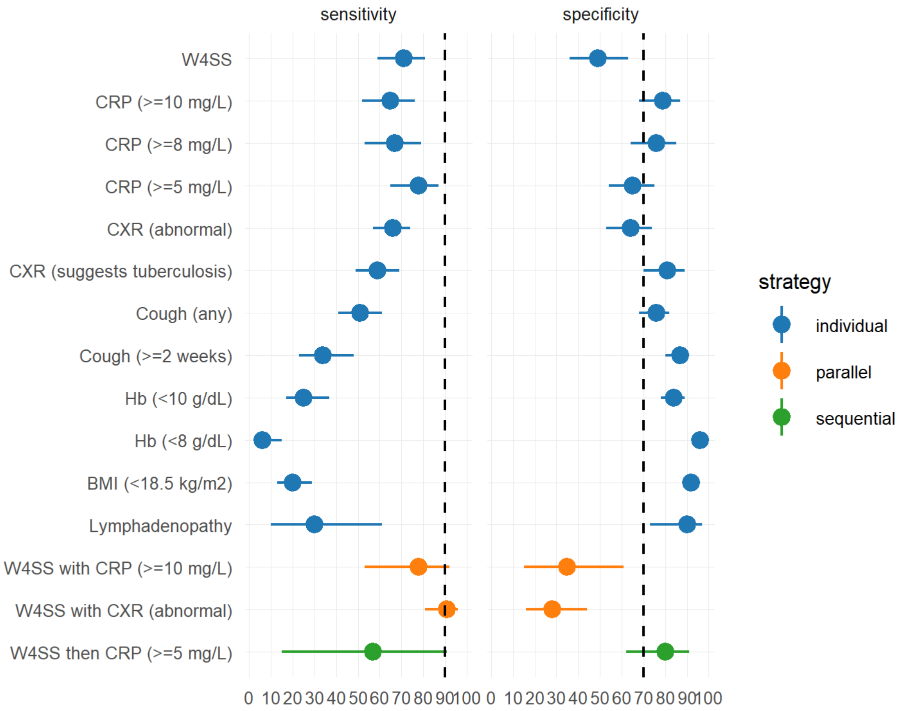

†Dashed lines indicate WHO's minimum requirements for a tuberculosis screening test (90% sensitivity and 70% specificity)
Definition of abbreviations: BMI = body mass index, CRP = C-reactive protein, CXR = chest X-ray, Hb = haemoglobin, W4SS = WHO four-symptom screen

Figure S2F - Plots of sensitivity and specificity for each screening test for the detection of tuberculosis in in pregnant participants†*

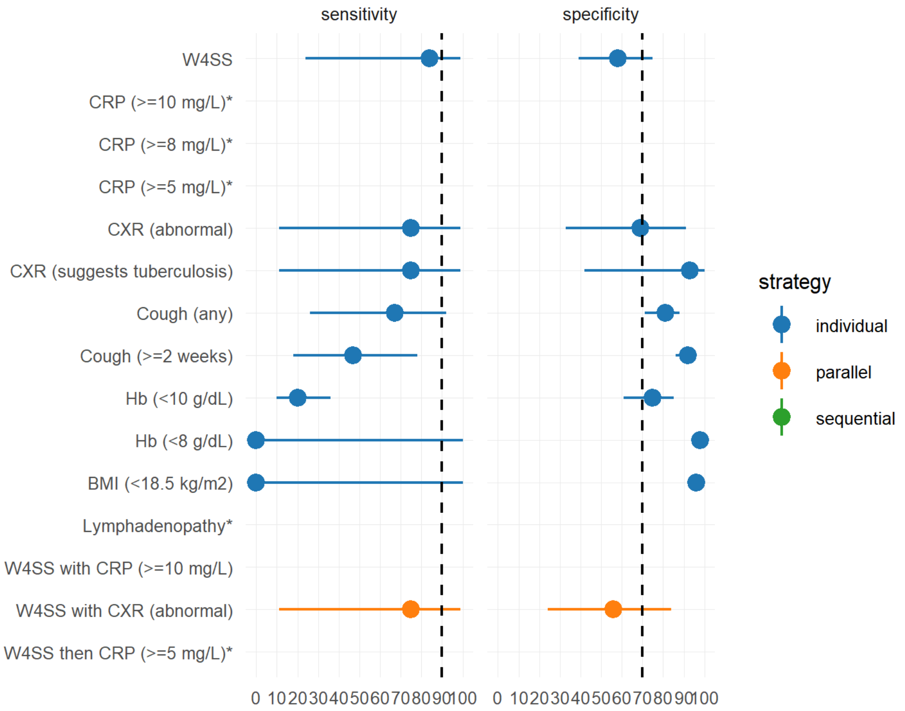

†Dashed lines indicate WHO's minimum requirements for a tuberculosis screening test (90% sensitivity and 70% specificity)
*Insufficient data to perform meta-analysis
Definition of abbreviations: BMI = body mass index, CRP = C-reactive protein, CXR = chest X-ray, Hb = haemoglobin, W4SS = WHO four-symptom screen

Figure S3 - Forest plots of sensitivity and specificity estimates in all participants and subgroups

Figure S3A - Forest plots of sensitivity and specificity estimates in all participants

All - Forest plot for
W4SS

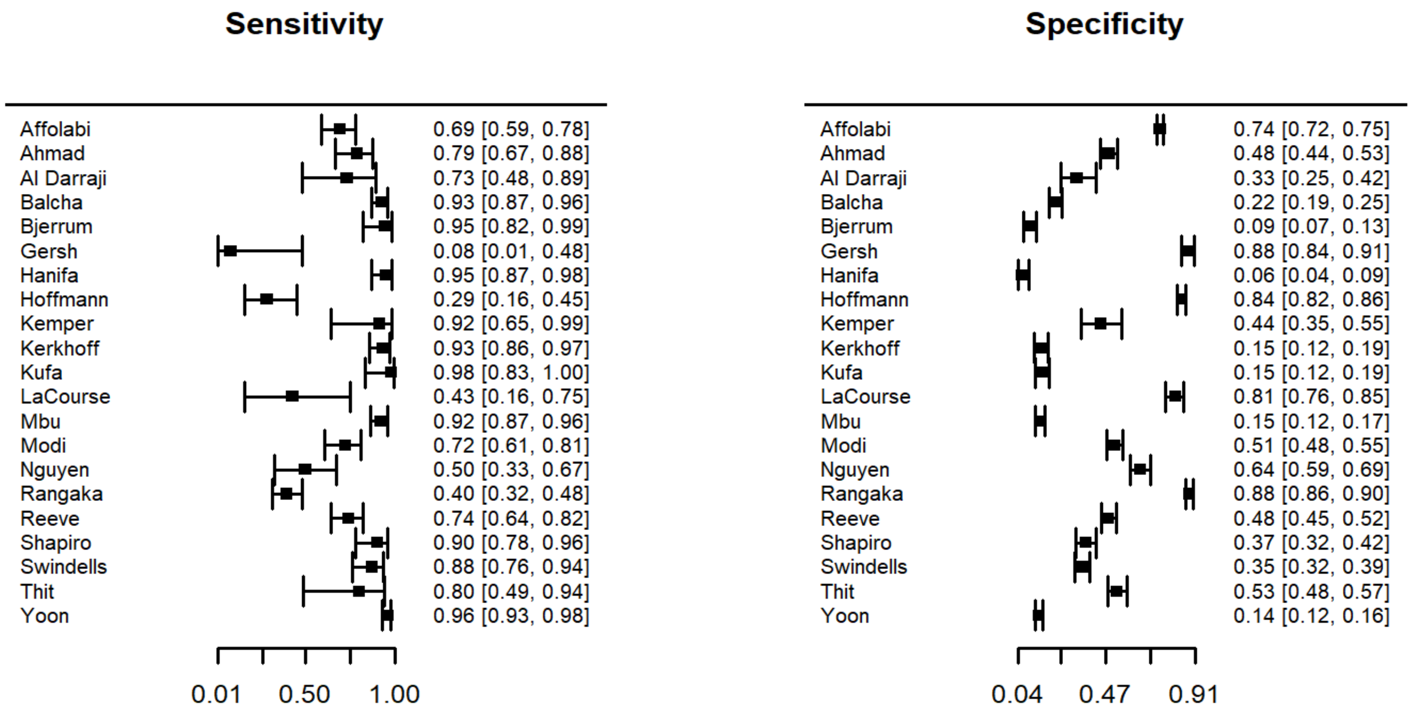

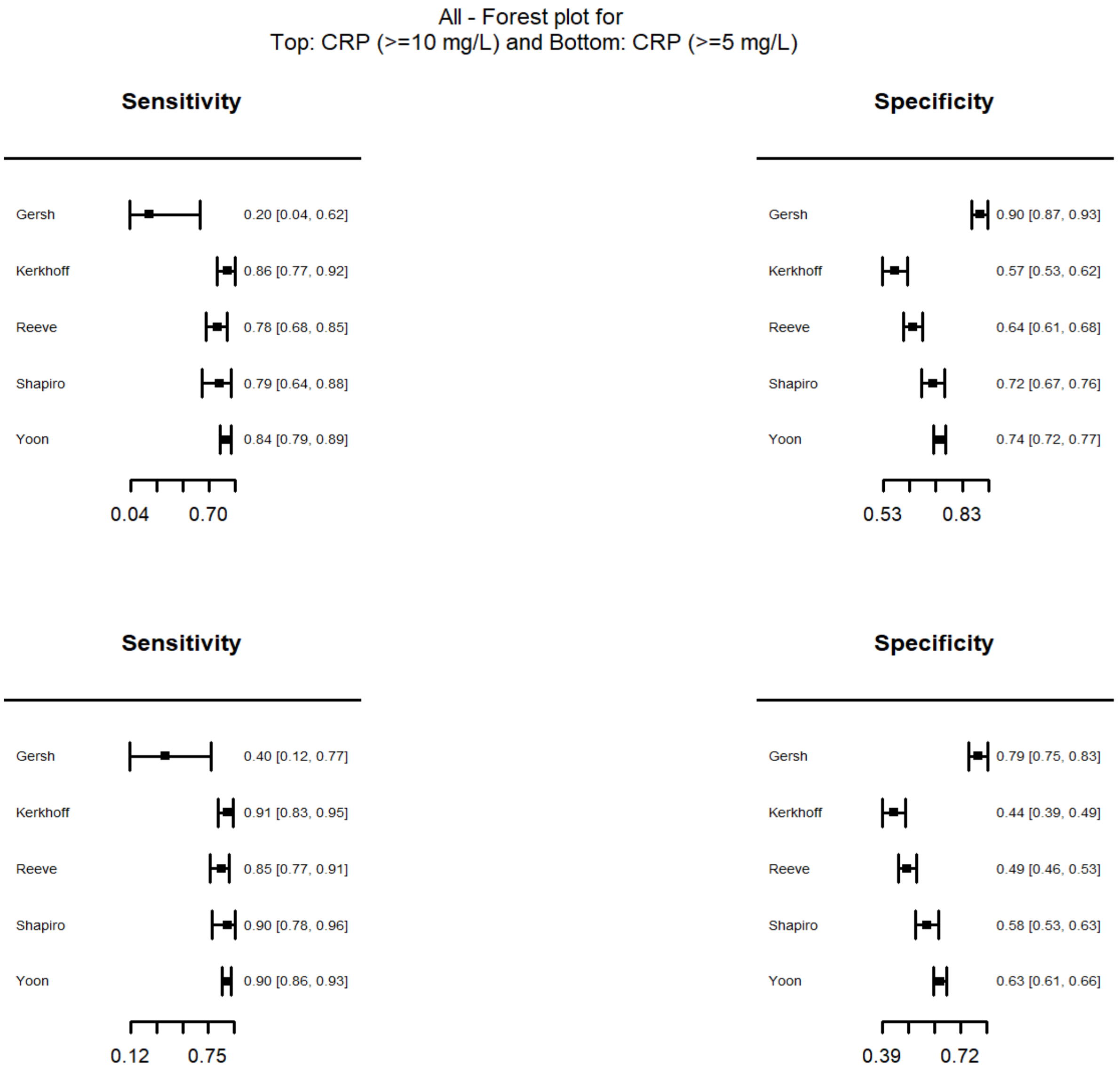
All - Forest plot for
Top: CRP (>=10 mg/L) and Bottom: CRP (>=5 mg/L)
Sensitivity
Gersh 0.20 [0.04, 0.62]
Kerkhoff 0.86 [0.77, 0.92]
Reeve 0.78 [0.68, 0.85]
Shapiro 0.79 [0.64, 0.88]
Yoon 0.84 [0.79, 0.89]
0.04 0.70
Specificity
Gersh 0.90 [0.87, 0.93]
Kerkhoff 0.57 [0.53, 0.62]
Reeve 0.64 [0.61, 0.68]
Shapiro 0.72 [0.67, 0.76]
Yoon 0.74 [0.72, 0.77]
0.53 0.83
Sensitivity
Gersh 0.40 [0.12, 0.77]
Kerkhoff 0.91 [0.83, 0.95]
Reeve 0.85 [0.77, 0.91]
Shapiro 0.90 [0.78, 0.96]
Yoon 0.90 [0.86, 0.93]
0.12 0.75
Specificity
Gersh 0.79 [0.75, 0.83]
Kerkhoff 0.44 [0.39, 0.49]
Reeve 0.49 [0.46, 0.53]
Shapiro 0.58 [0.53, 0.63]
Yoon 0.63 [0.61, 0.66]
0.39 0.72

All - Forest plot for
Top: CXR (abnormal) and Bottom: CXR (suggests tuberculosis)

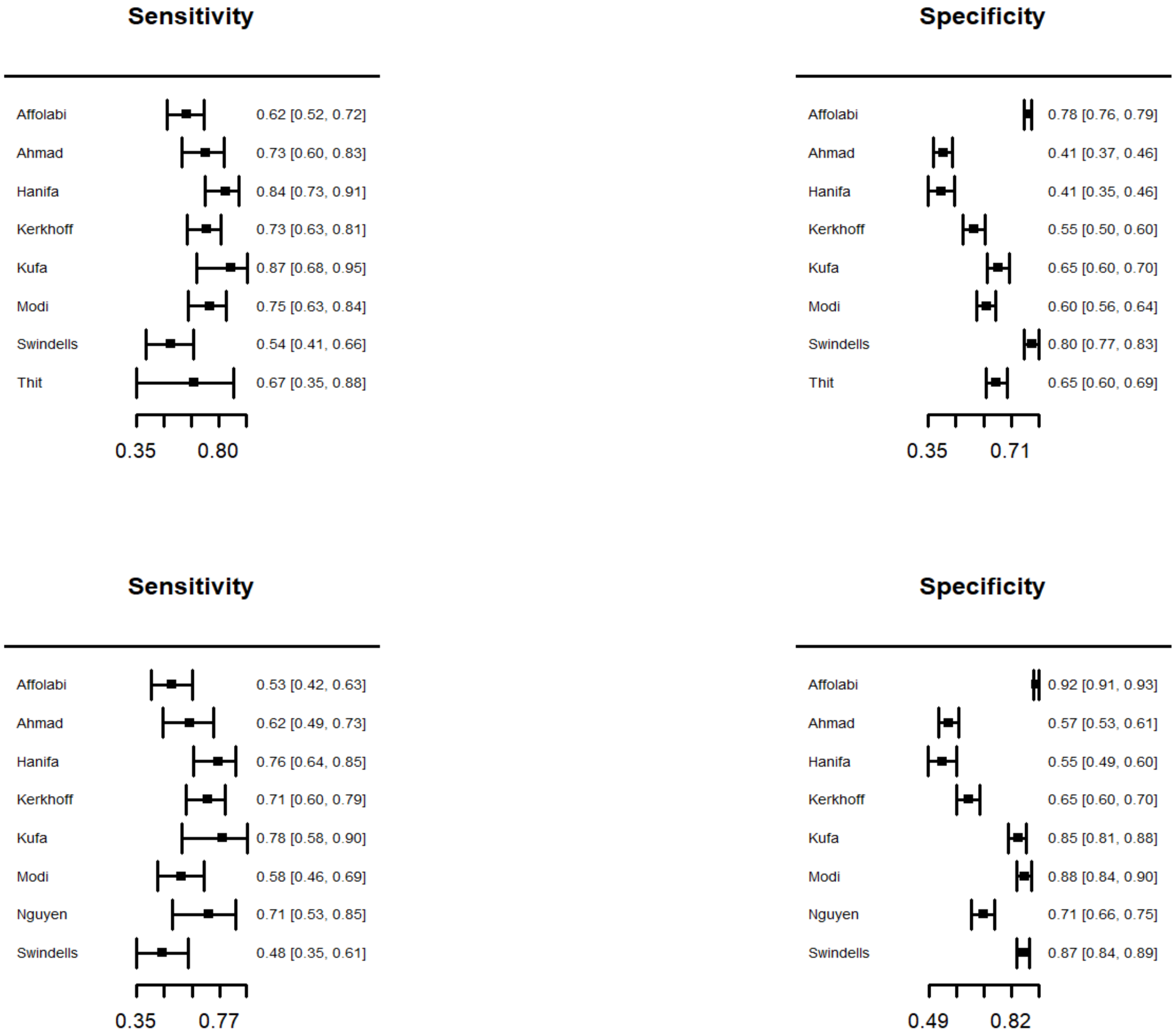

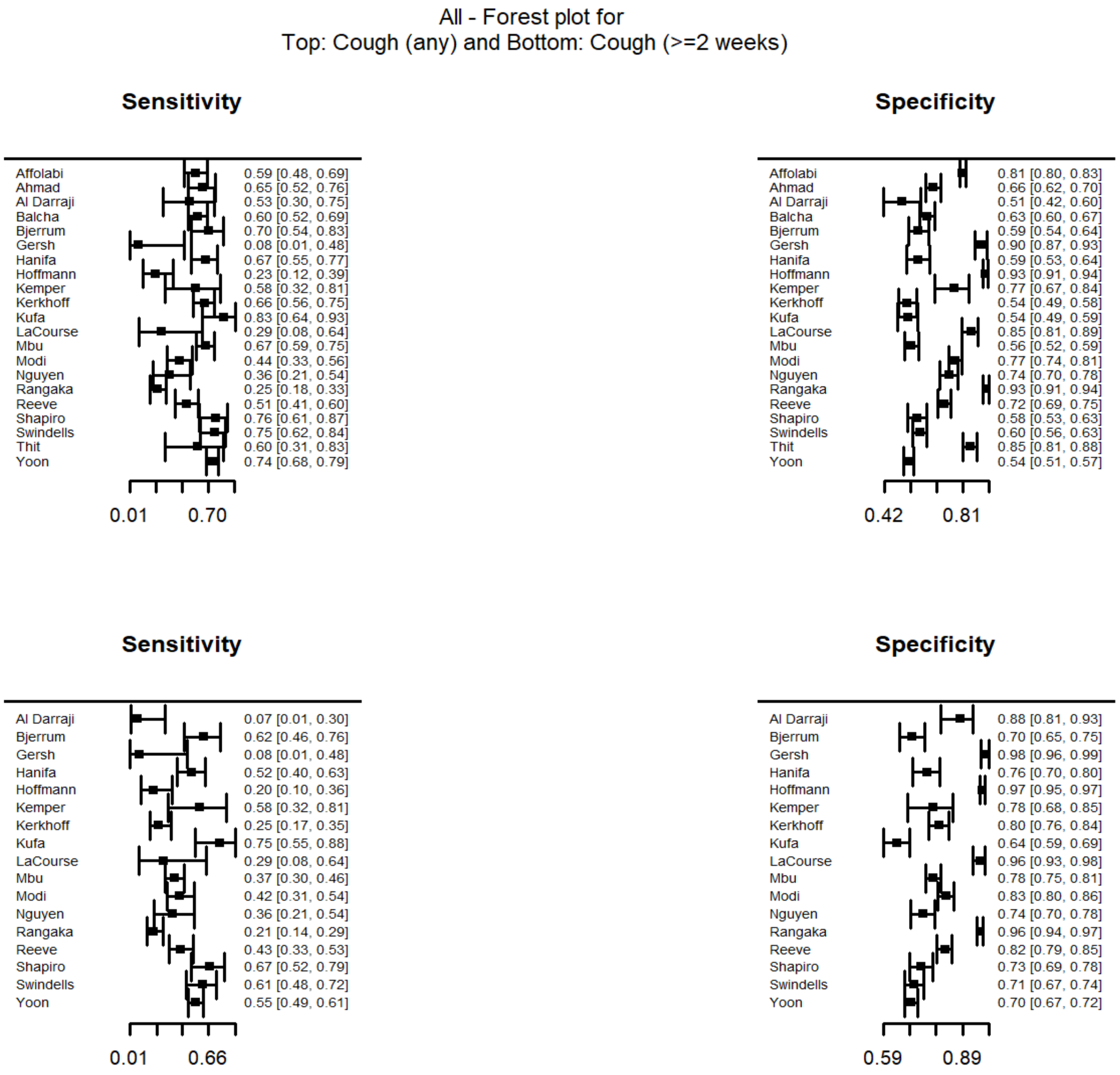

All - Forest plot for
Top: Cough (any) and Bottom: Cough (>=2 weeks)
Sensitivity
Affolabi 0.59 [0.48, 0.69]
Ahmad 0.65 [0.52, 0.76]
Al Darraji 0.53 [0.30, 0.75]
Balcha 0.60 [0.52, 0.69]
Bjerrum 0.70 [0.54, 0.83]
Gersh 0.08 [0.01, 0.48]
Hanifa 0.67 [0.55, 0.77]
Hoffmann 0.23 [0.12, 0.39]
Kemper 0.58 [0.32, 0.81]
Kerkhoff 0.66 [0.56, 0.75]
Kufa 0.83 [0.64, 0.93]
LaCourse 0.29 [0.08, 0.64]
Mbu 0.67 [0.59, 0.75]
Modi 0.44 [0.33, 0.56]
Nguyen 0.36 [0.21, 0.54]
Rangaka 0.25 [0.18, 0.33]
Reeve 0.51 [0.41, 0.60]
Shapiro 0.76 [0.61, 0.87]
Swindells 0.75 [0.62, 0.84]
Thit 0.60 [0.31, 0.83]
Yoon 0.74 [0.68, 0.79]
0.01 0.70
Specificity
Affolabi 0.81 [0.80, 0.83]
Ahmad 0.66 [0.62, 0.70]
Al Darraji 0.51 [0.42, 0.60]
Balcha 0.63 [0.60, 0.67]
Bjerrum 0.59 [0.54, 0.64]
Gersh 0.90 [0.87, 0.93]
Hanifa 0.59 [0.53, 0.64]
Hoffmann 0.93 [0.91, 0.94]
Kemper 0.77 [0.67, 0.84]
Kerkhoff 0.54 [0.49, 0.58]
Kufa 0.54 [0.49, 0.59]
LaCourse 0.85 [0.81, 0.89]
Mbu 0.56 [0.52, 0.59]
Modi 0.77 [0.74, 0.81]
Nguyen 0.74 [0.70, 0.78]
Rangaka 0.93 [0.91, 0.94]
Reeve 0.72 [0.69, 0.75]
Shapiro 0.58 [0.53, 0.63]
Swindells 0.60 [0.56, 0.63]
Thit 0.85 [0.81, 0.88]
Yoon 0.54 [0.51, 0.57]
0.42 0.81
Sensitivity
Al Darraji 0.07 [0.01, 0.30]
Bjerrum 0.62 [0.46, 0.76]
Gersh 0.08 [0.01, 0.48]
Hanifa 0.52 [0.40, 0.63]
Hoffmann 0.20 [0.10, 0.36]
Kemper 0.58 [0.32, 0.81]
Kerkhoff 0.25 [0.17, 0.35]
Kufa 0.75 [0.55, 0.88]
LaCourse 0.29 [0.08, 0.64]
Mbu 0.37 [0.30, 0.46]
Modi 0.42 [0.31, 0.54]
Nguyen 0.36 [0.21, 0.54]
Rangaka 0.21 [0.14, 0.29]
Reeve 0.43 [0.33, 0.53]
Shapiro 0.67 [0.52, 0.79]
Swindells 0.61 [0.48, 0.72]
Yoon 0.55 [0.49, 0.61]
0.01 0.66
Specificity
Al Darraji 0.88 [0.81, 0.93]
Bjerrum 0.70 [0.65, 0.75]
Gersh 0.98 [0.96, 0.99]
Hanifa 0.76 [0.70, 0.80]
Hoffmann 0.97 [0.95, 0.97]
Kemper 0.78 [0.68, 0.85]
Kerkhoff 0.80 [0.76, 0.84]
Kufa 0.64 [0.59, 0.69]
LaCourse 0.96 [0.93, 0.98]
Mbu 0.78 [0.75, 0.81]
Modi 0.83 [0.80, 0.86]
Nguyen 0.74 [0.70, 0.78]
Rangaka 0.96 [0.94, 0.97]
Reeve 0.82 [0.79, 0.85]
Shapiro 0.73 [0.69, 0.78]
Swindells 0.71 [0.67, 0.74]
Yoon 0.70 [0.67, 0.72]
0.59 0.89

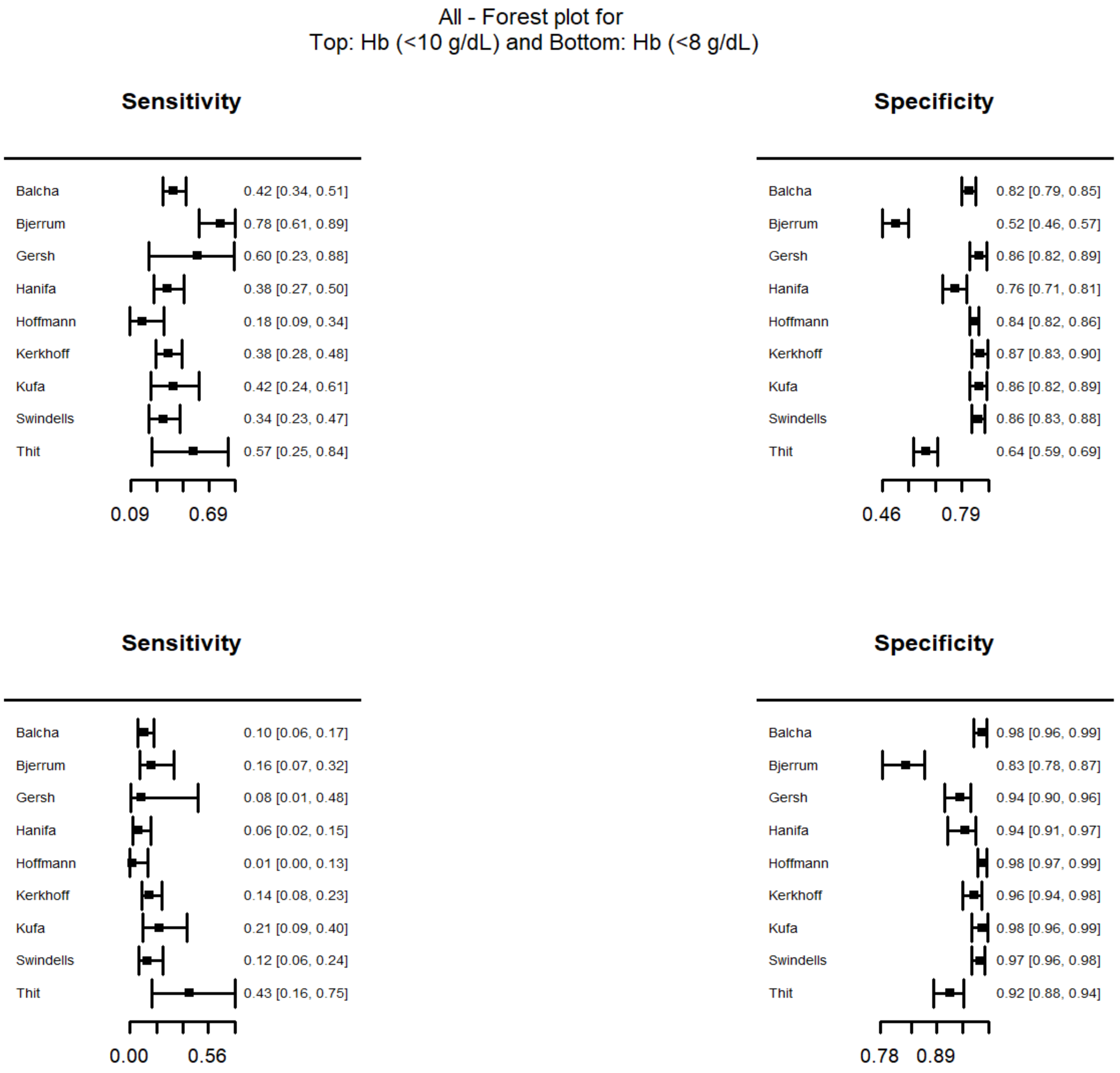
All - Forest plot for
Top: Hb (<10 g/dL) and Bottom: Hb (<8 g/dL)
Sensitivity
Balcha 0.42 [0.34, 0.51]
Bjerrum 0.78 [0.61, 0.89]
Gersh 0.60 [0.23, 0.88]
Hanifa 0.38 [0.27, 0.50]
Hoffmann 0.18 [0.09, 0.34]
Kerkhoff 0.38 [0.28, 0.48]
Kufa 0.42 [0.24, 0.61]
Swindells 0.34 [0.23, 0.47]
Thit 0.57 [0.25, 0.84]
0.09 0.69
Specificity
Balcha 0.82 [0.79, 0.85]
Bjerrum 0.52 [0.46, 0.57]
Gersh 0.86 [0.82, 0.89]
Hanifa 0.76 [0.71, 0.81]
Hoffmann 0.84 [0.82, 0.86]
Kerkhoff 0.87 [0.83, 0.90]
Kufa 0.86 [0.82, 0.89]
Swindells 0.86 [0.83, 0.88]
Thit 0.64 [0.59, 0.69]
0.46 0.79
Sensitivity
Balcha 0.10 [0.06, 0.17]
Bjerrum 0.16 [0.07, 0.32]
Gersh 0.08 [0.01, 0.48]
Hanifa 0.06 [0.02, 0.15]
Hoffmann 0.01 [0.00, 0.13]
Kerkhoff 0.14 [0.08, 0.23]
Kufa 0.21 [0.09, 0.40]
Swindells 0.12 [0.06, 0.24]
Thit 0.43 [0.16, 0.75]
0.00 0.56
Specificity
Balcha 0.98 [0.96, 0.99]
Bjerrum 0.83 [0.78, 0.87]
Gersh 0.94 [0.90, 0.96]
Hanifa 0.94 [0.91, 0.97]
Hoffmann 0.98 [0.97, 0.99]
Kerkhoff 0.96 [0.94, 0.98]
Kufa 0.98 [0.96, 0.99]
Swindells 0.97 [0.96, 0.98]
Thit 0.92 [0.88, 0.94]
0.78 0.89

All - Forest plot for
BMI (<18.5 kg/m^2)

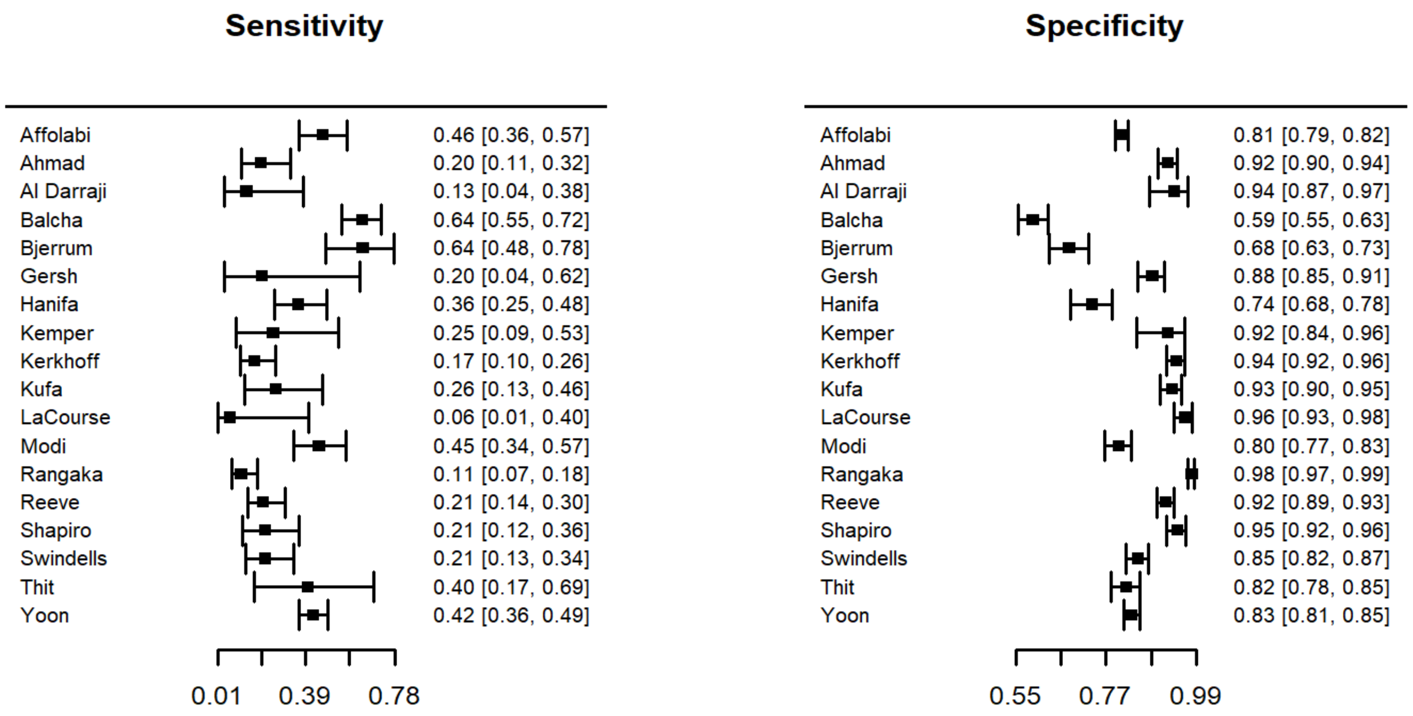

All - Forest plot for
Lymphadenopathy

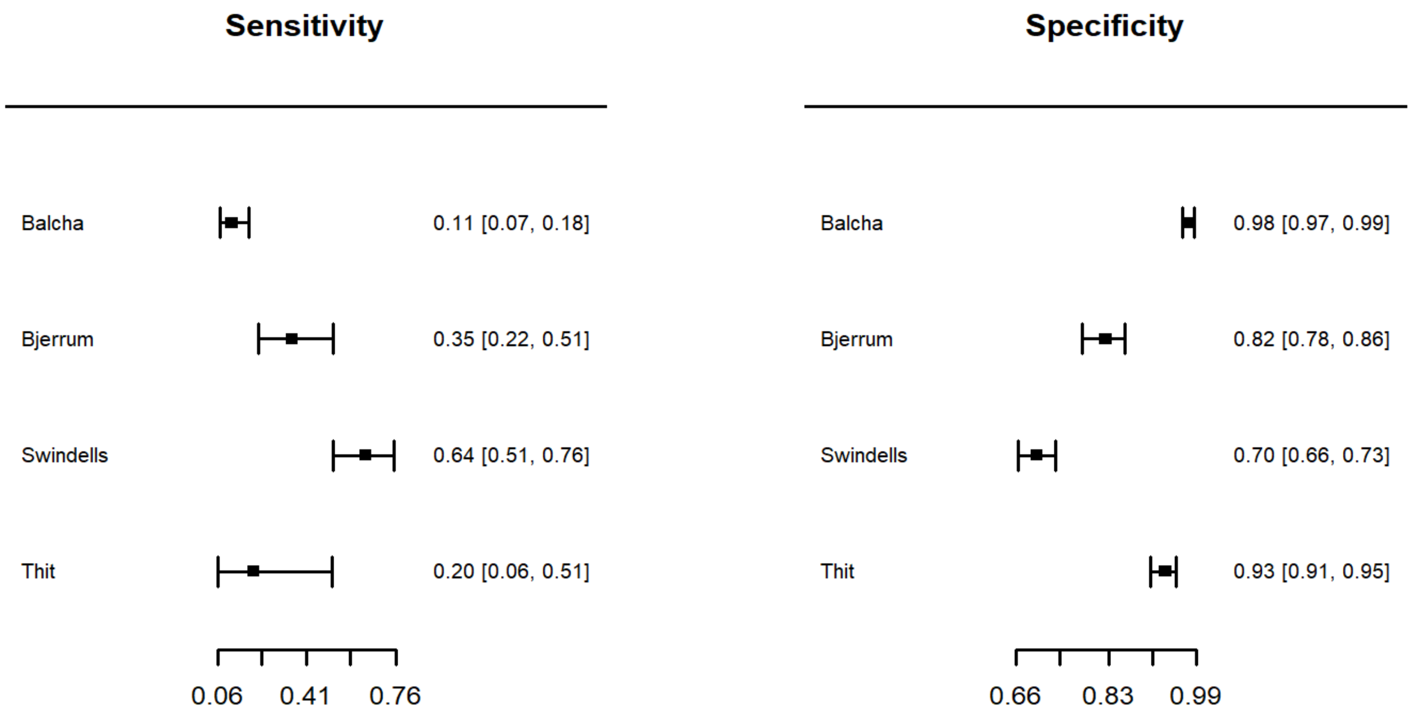

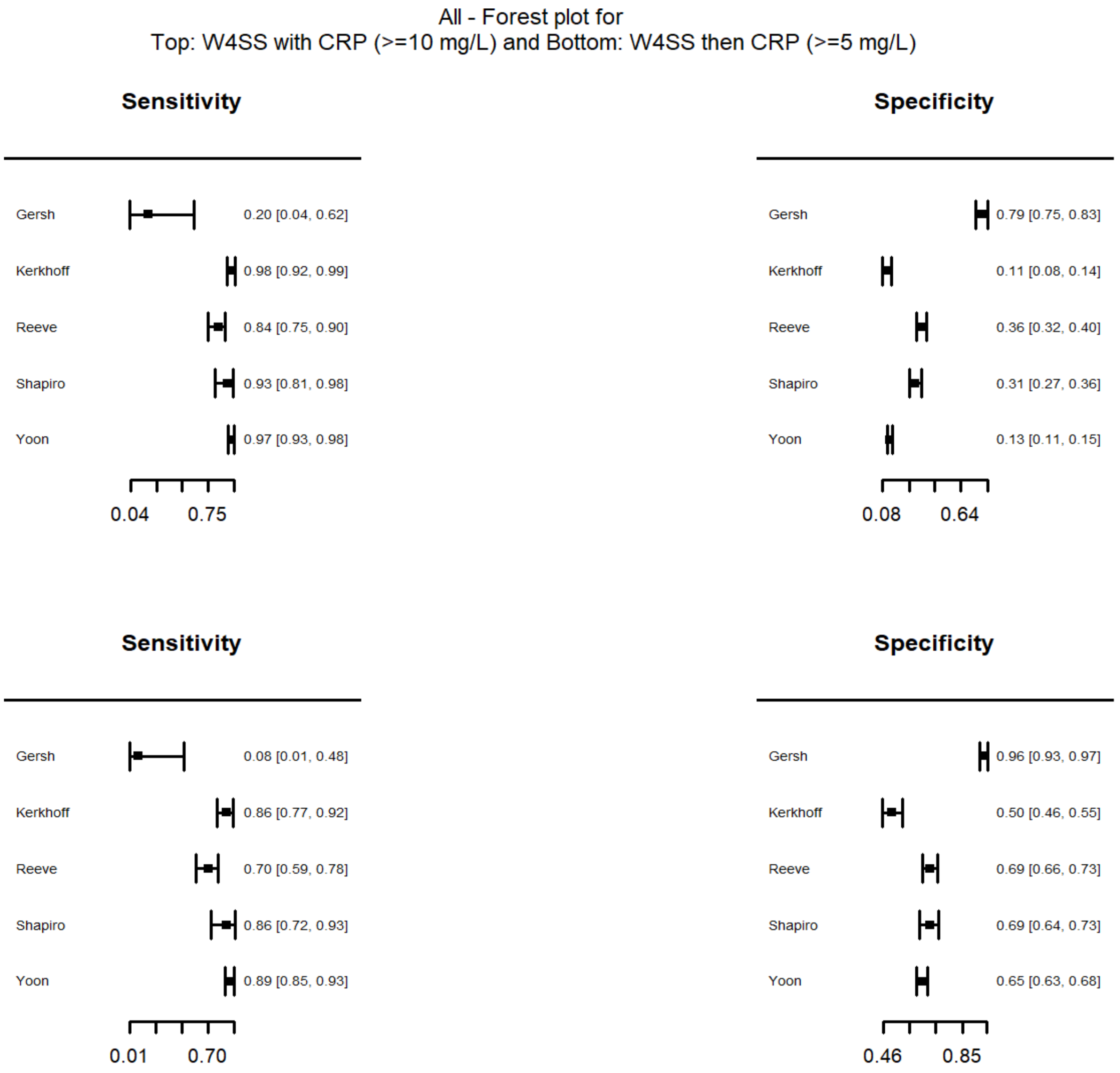
All - Forest plot for
Top: W4SS with CRP (>=10 mg/L) and Bottom: W4SS then CRP (>=5 mg/L)
Sensitivity
Gersh 0.20 [0.04, 0.62]
Kerkhoff 0.98 [0.92, 0.99]
Reeve 0.84 [0.75, 0.90]
Shapiro 0.93 [0.81, 0.98]
Yoon 0.97 [0.93, 0.98]
0.04 0.75
Specificity
Gersh 0.79 [0.75, 0.83]
Kerkhoff 0.11 [0.08, 0.14]
Reeve 0.36 [0.32, 0.40]
Shapiro 0.31 [0.27, 0.36]
Yoon 0.13 [0.11, 0.15]
0.08 0.64
Sensitivity
Gersh 0.08 [0.01, 0.48]
Kerkhoff 0.86 [0.77, 0.92]
Reeve 0.70 [0.59, 0.78]
Shapiro 0.86 [0.72, 0.93]
Yoon 0.89 [0.85, 0.93]
0.01 0.70
Specificity
Gersh 0.96 [0.93, 0.97]
Kerkhoff 0.50 [0.46, 0.55]
Reeve 0.69 [0.66, 0.73]
Shapiro 0.69 [0.64, 0.73]
Yoon 0.65 [0.63, 0.68]
0.46 0.85

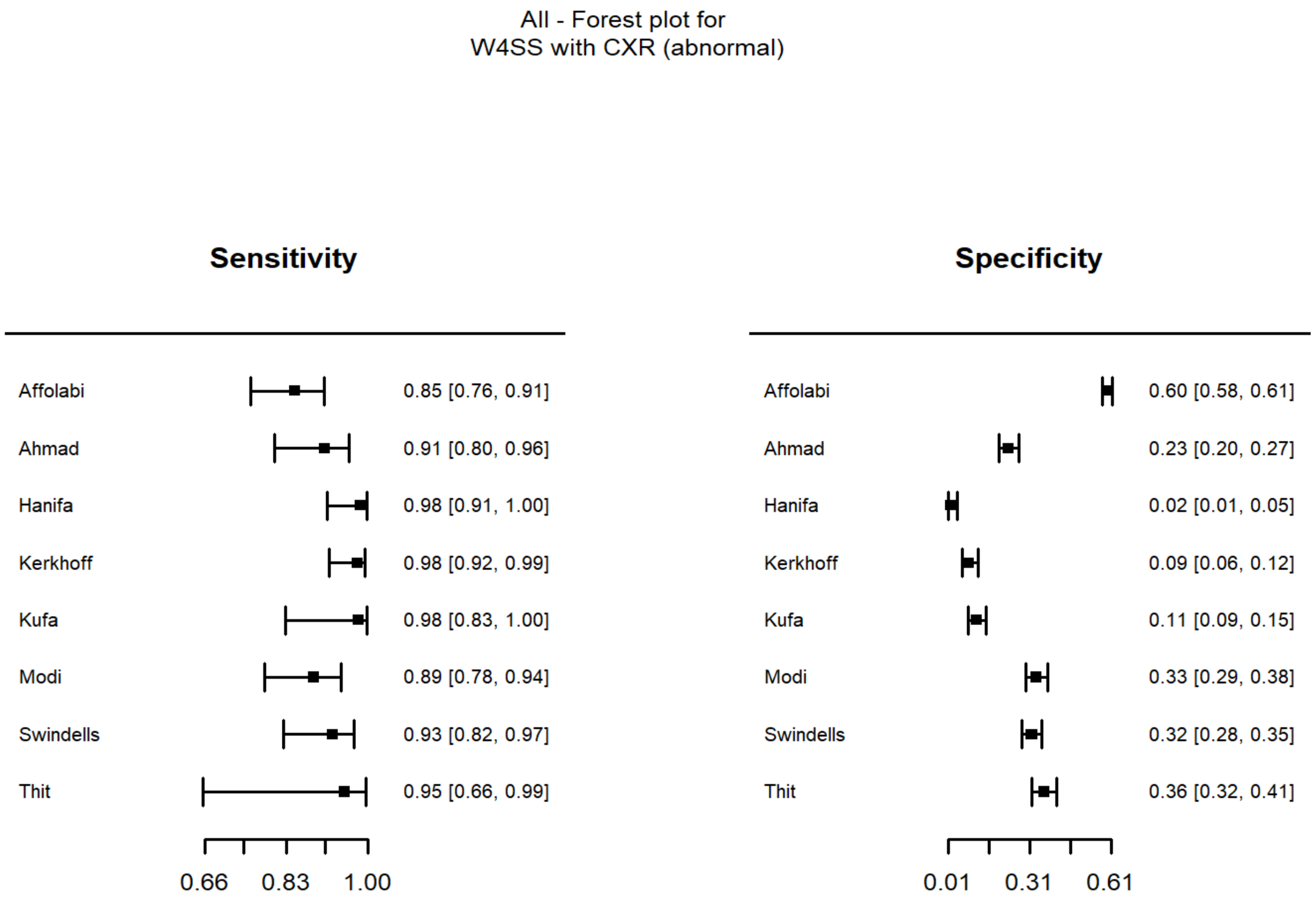

All - Forest plot for
W4SS with CXR (abnormal)
Sensitivity
Affolabi 0.85 [0.76, 0.91]
Ahmad 0.91 [0.80, 0.96]
Hanifa 0.98 [0.91, 1.00]
Kerkhoff 0.98 [0.92, 0.99]
Kufa 0.98 [0.83, 1.00]
Modi 0.89 [0.78, 0.94]
Swindells 0.93 [0.82, 0.97]
Thit 0.95 [0.66, 0.99]
0.66
0.83
1.00
Specificity
Affolabi 0.60 [0.58, 0.61]
Ahmad 0.23 [0.20, 0.27]
Hanifa 0.02 [0.01, 0.05]
Kerkhoff 0.09 [0.06, 0.12]
Kufa 0.11 [0.09, 0.15]
Modi 0.33 [0.29, 0.38]
Swindells 0.32 [0.28, 0.35]
Thit 0.36 [0.32, 0.41]
0.01
0.31
0.61

All - Forest plot for
Top: W4SS then Xpert and Bottom: Xpert alone

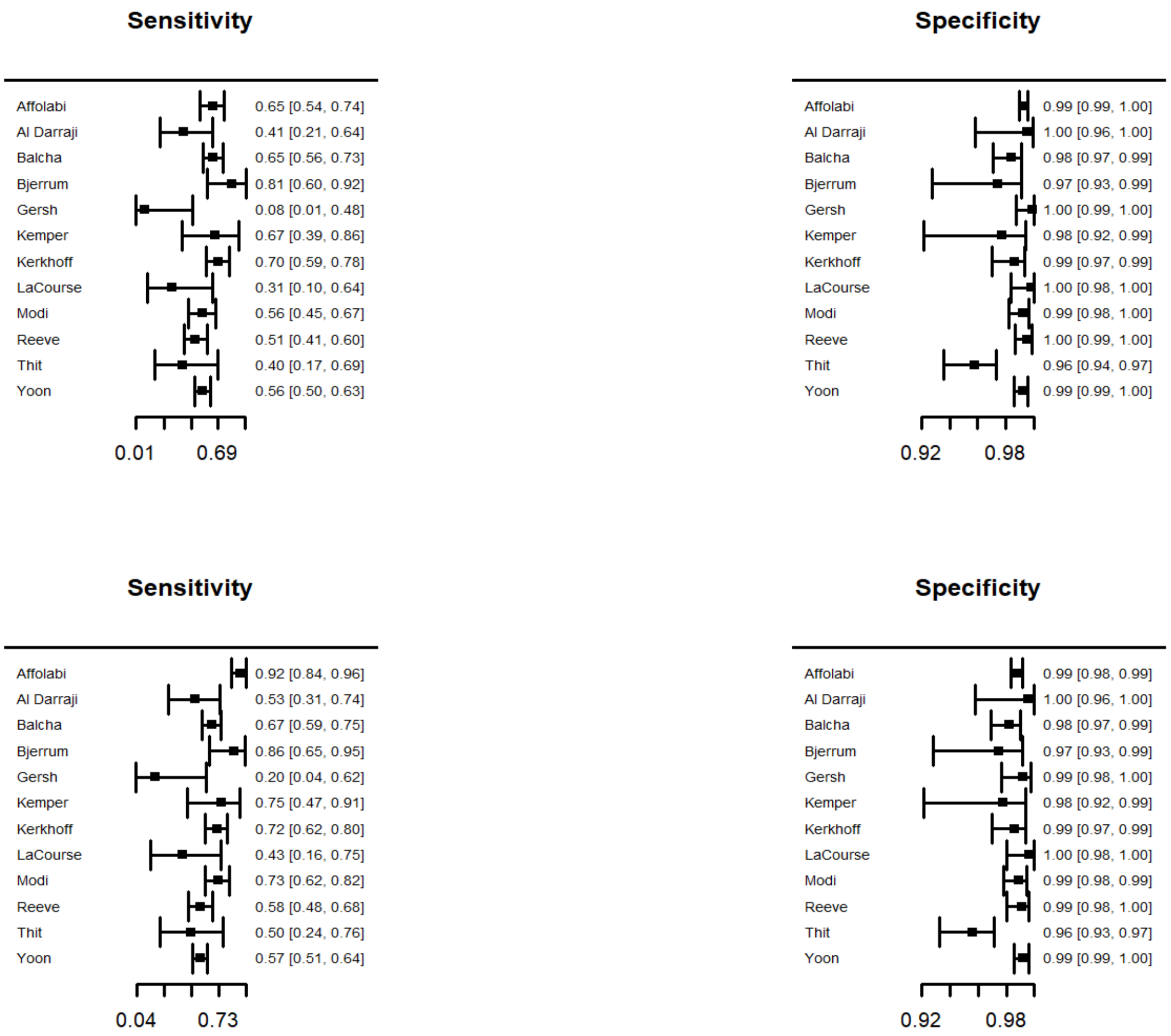

Figure S3B - Forest plots of sensitivity and specificity estimates in outpatients (on ART)

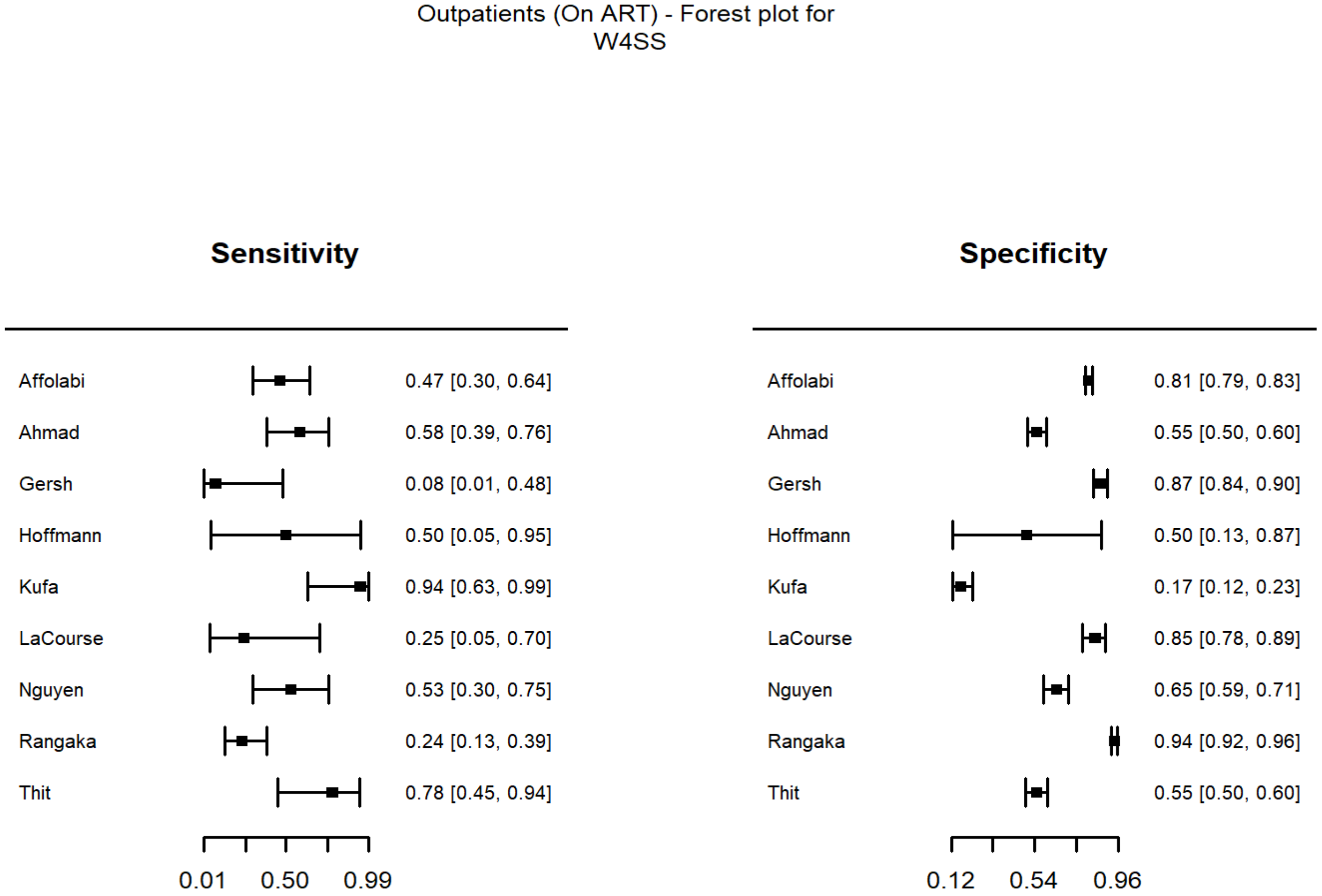

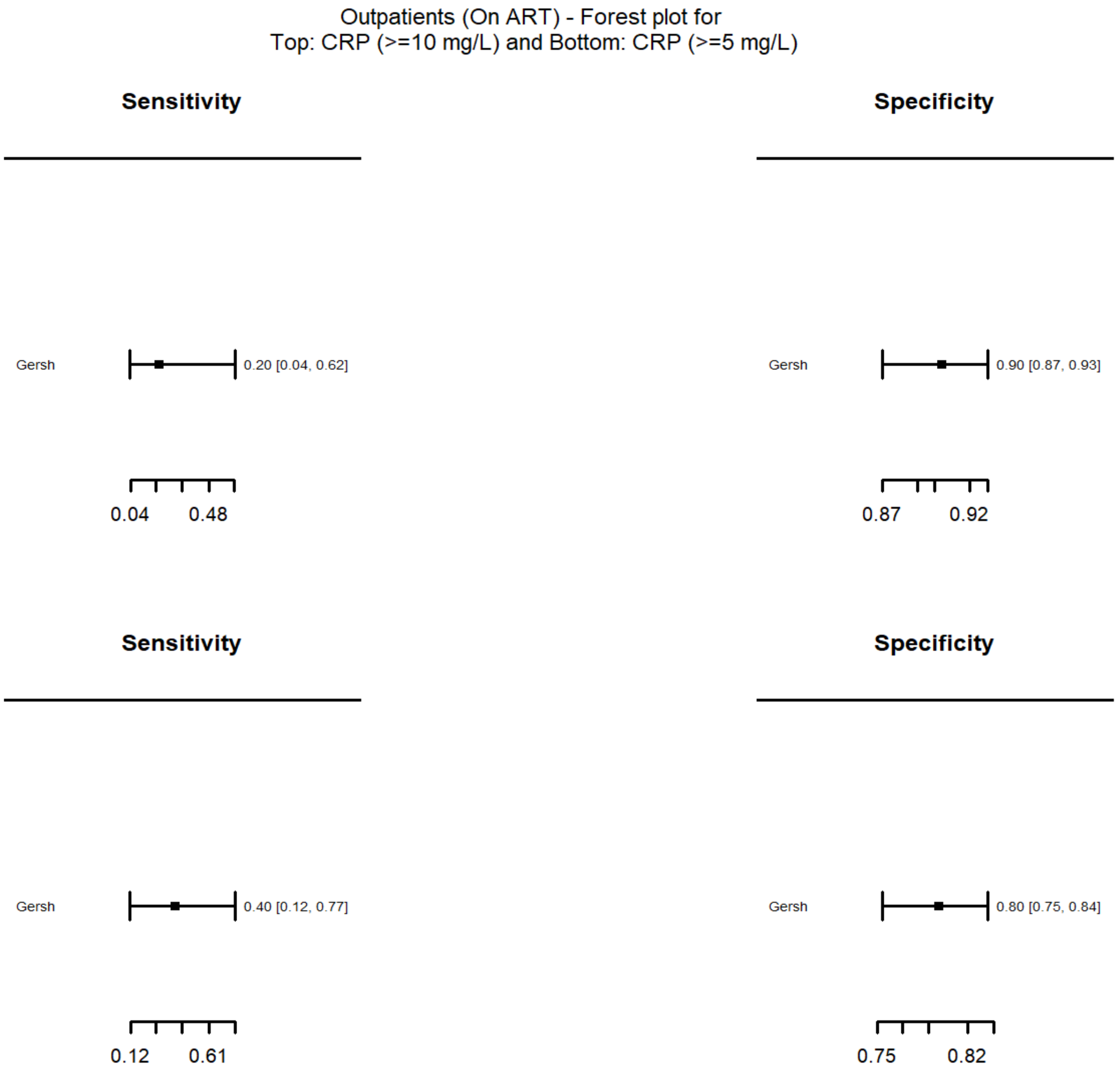
Outpatients (On ART) - Forest plot for
Top: CRP (>=10 mg/L) and Bottom: CRP (>=5 mg/L)
Sensitivity
Specificity
Gersh
0.20 [0.04, 0.62]
Gersh
0.90 [0.87, 0.93]
0.04
0.48
0.87
0.92
Sensitivity
Specificity
Gersh
0.40 [0.12, 0.77]
Gersh
0.80 [0.75, 0.84]
0.12
0.61
0.75
0.82

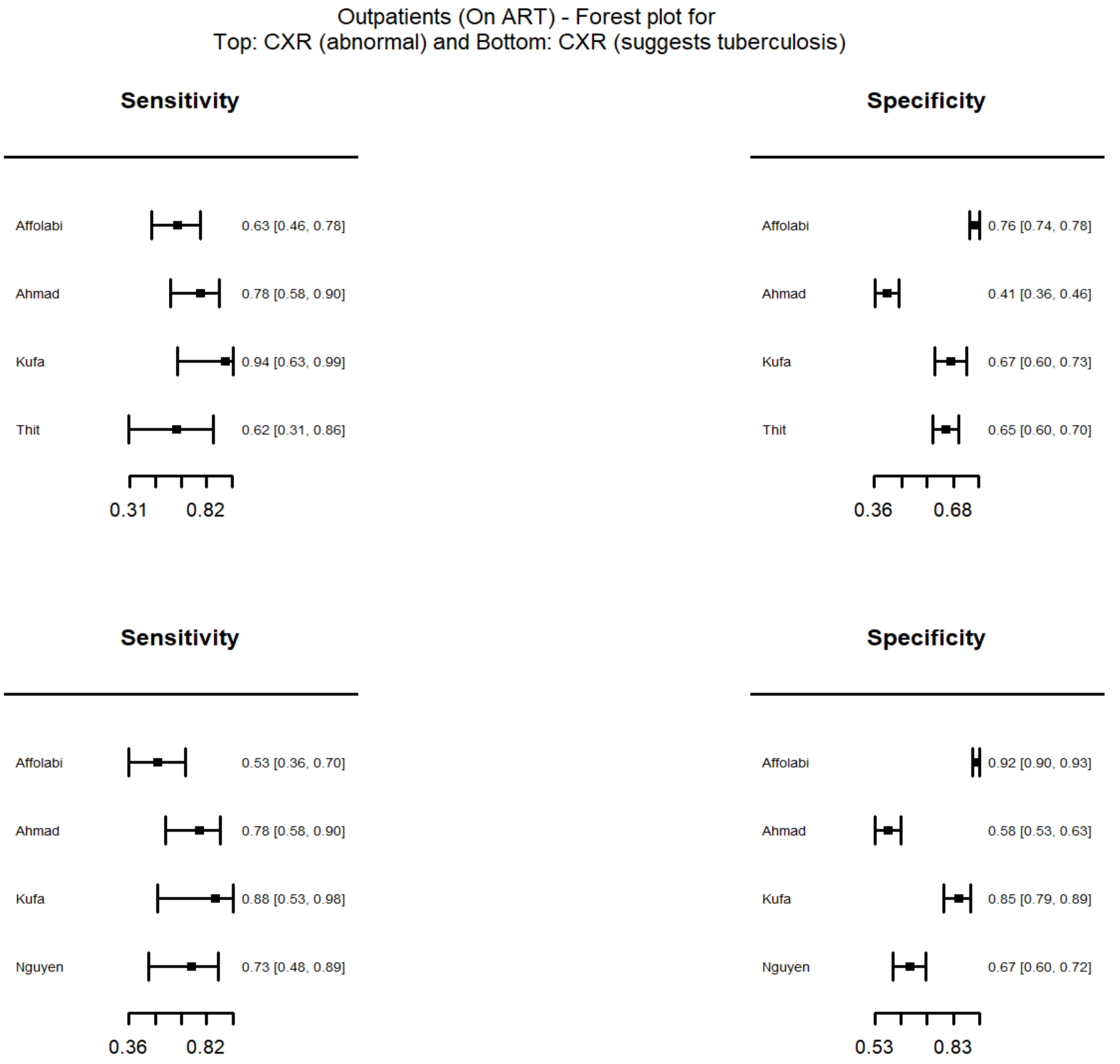
Outpatients (On ART) - Forest plot for
Top: CXR (abnormal) and Bottom: CXR (suggests tuberculosis)
Sensitivity
Specificity
Affolabi 0.63 [0.46, 0.78]
Ahmad 0.78 [0.58, 0.90]
Kufa 0.94 [0.63, 0.99]
Thit 0.62 [0.31, 0.86]
0.31 0.82
Affolabi 0.76 [0.74, 0.78]
Ahmad 0.41 [0.36, 0.46]
Kufa 0.67 [0.60, 0.73]
Thit 0.65 [0.60, 0.70]
0.36 0.68
Sensitivity
Specificity
Affolabi 0.53 [0.36, 0.70]
Ahmad 0.78 [0.58, 0.90]
Kufa 0.88 [0.53, 0.98]
Nguyen 0.73 [0.48, 0.89]
0.36 0.82
Affolabi 0.92 [0.90, 0.93]
Ahmad 0.58 [0.53, 0.63]
Kufa 0.85 [0.79, 0.89]
Nguyen 0.67 [0.60, 0.72]
0.53 0.83

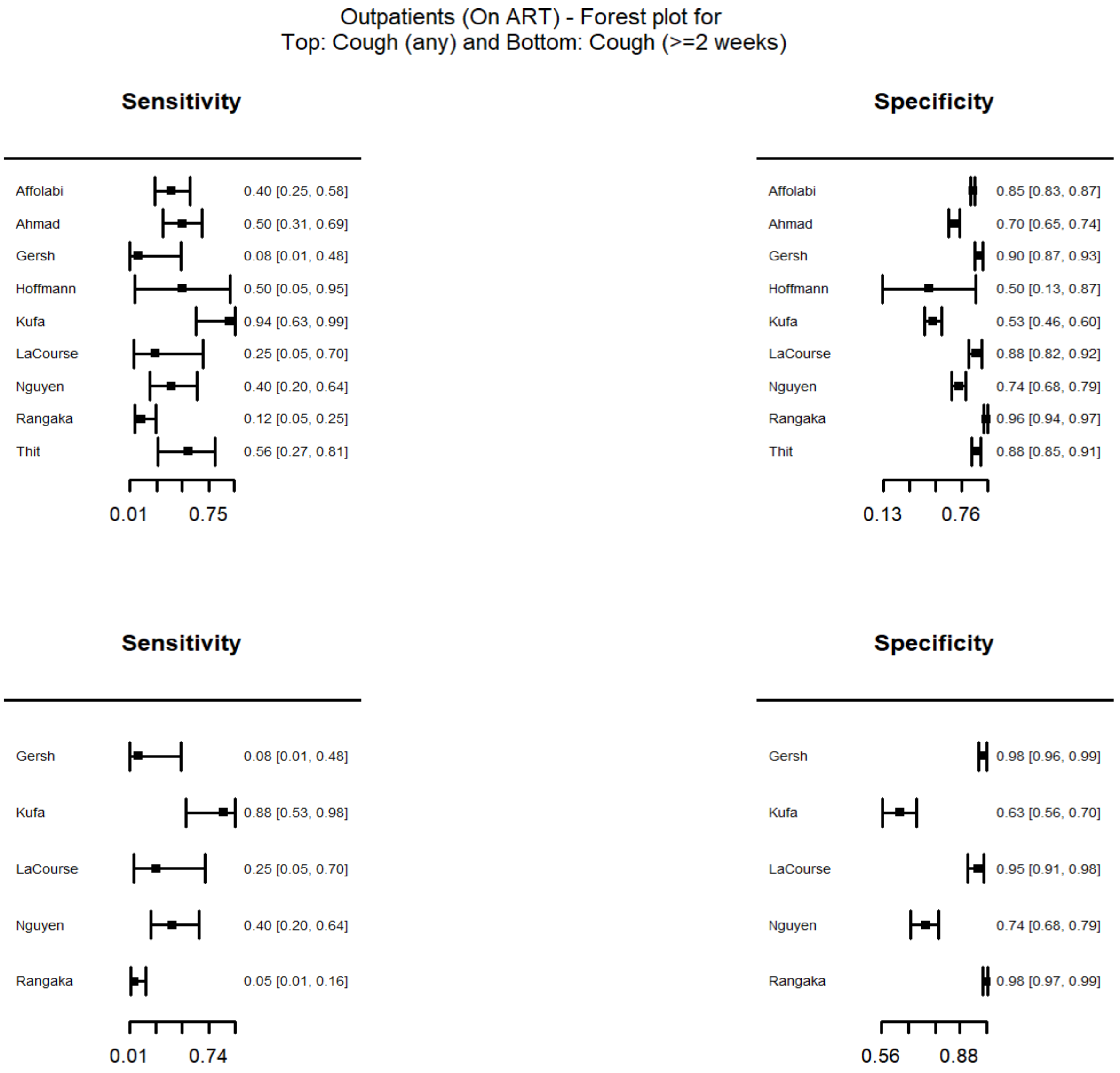

Outpatients (On ART) - Forest plot for
Top: Cough (any) and Bottom: Cough (>=2 weeks)
Sensitivity
Affolabi 0.40 [0.25, 0.58]
Ahmad 0.50 [0.31, 0.69]
Gersh 0.08 [0.01, 0.48]
Hoffmann 0.50 [0.05, 0.95]
Kufa 0.94 [0.63, 0.99]
LaCourse 0.25 [0.05, 0.70]
Nguyen 0.40 [0.20, 0.64]
Rangaka 0.12 [0.05, 0.25]
Thit 0.56 [0.27, 0.81]
0.01
0.75
Specificity
Affolabi 0.85 [0.83, 0.87]
Ahmad 0.70 [0.65, 0.74]
Gersh 0.90 [0.87, 0.93]
Hoffmann 0.50 [0.13, 0.87]
Kufa 0.53 [0.46, 0.60]
LaCourse 0.88 [0.82, 0.92]
Nguyen 0.74 [0.68, 0.79]
Rangaka 0.96 [0.94, 0.97]
Thit 0.88 [0.85, 0.91]
0.13
0.76
Sensitivity
Gersh 0.08 [0.01, 0.48]
Kufa 0.88 [0.53, 0.98]
LaCourse 0.25 [0.05, 0.70]
Nguyen 0.40 [0.20, 0.64]
Rangaka 0.05 [0.01, 0.16]
0.01
0.74
Specificity
Gersh 0.98 [0.96, 0.99]
Kufa 0.63 [0.56, 0.70]
LaCourse 0.95 [0.91, 0.98]
Nguyen 0.74 [0.68, 0.79]
Rangaka 0.98 [0.97, 0.99]
0.56
0.88

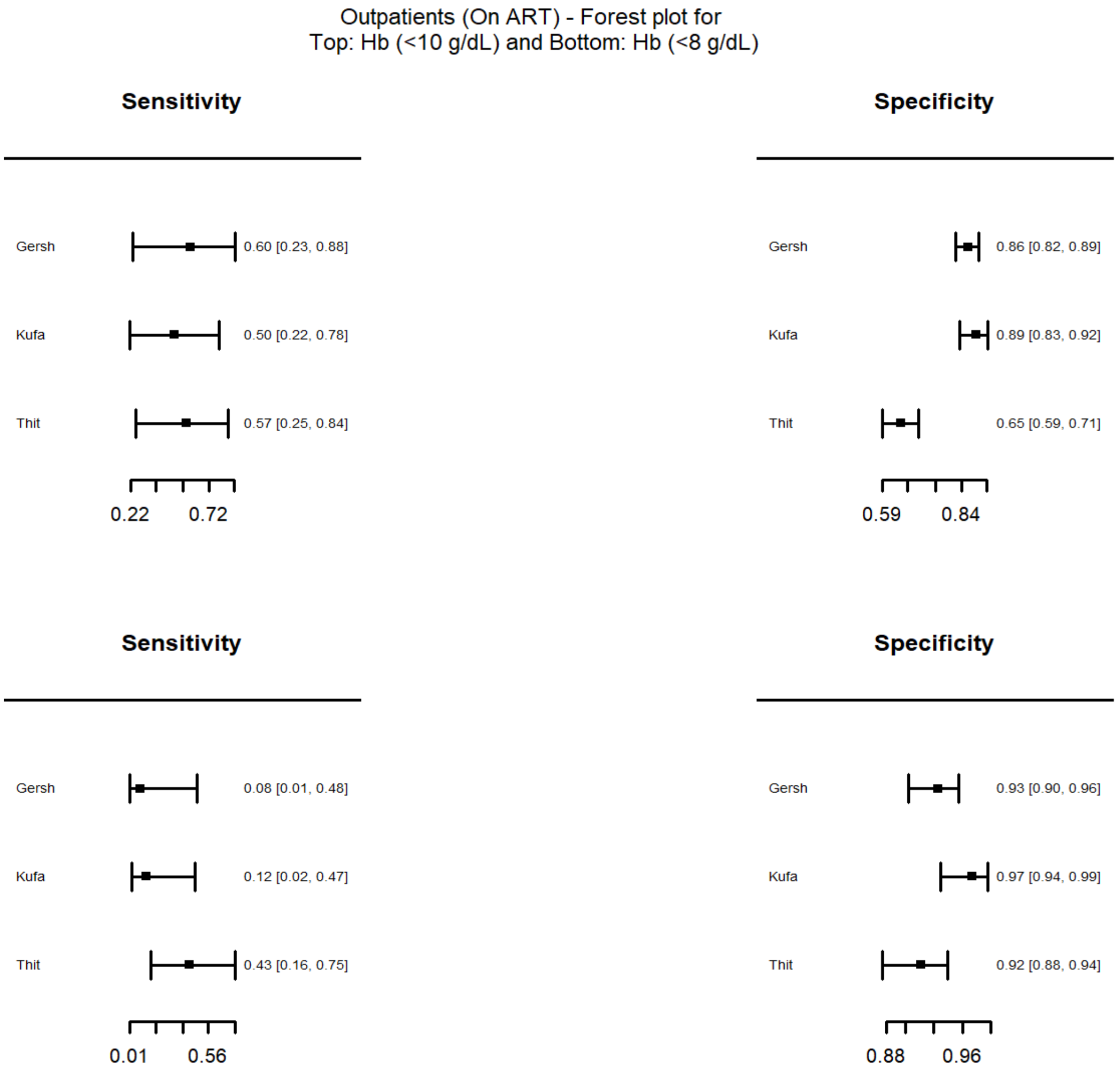
Outpatients (On ART) - Forest plot for
Top: Hb (<10 g/dL) and Bottom: Hb (<8 g/dL)
Sensitivity
Specificity
Gersh
0.60 [0.23, 0.88]
Gersh
0.86 [0.82, 0.89]
Kufa
0.50 [0.22, 0.78]
Kufa
0.89 [0.83, 0.92]
Thit
0.57 [0.25, 0.84]
Thit
0.65 [0.59, 0.71]
0.22
0.72
0.59
0.84
Sensitivity
Specificity
Gersh
0.08 [0.01, 0.48]
Gersh
0.93 [0.90, 0.96]
Kufa
0.12 [0.02, 0.47]
Kufa
0.97 [0.94, 0.99]
Thit
0.43 [0.16, 0.75]
Thit
0.92 [0.88, 0.94]
0.01
0.56
0.88
0.96

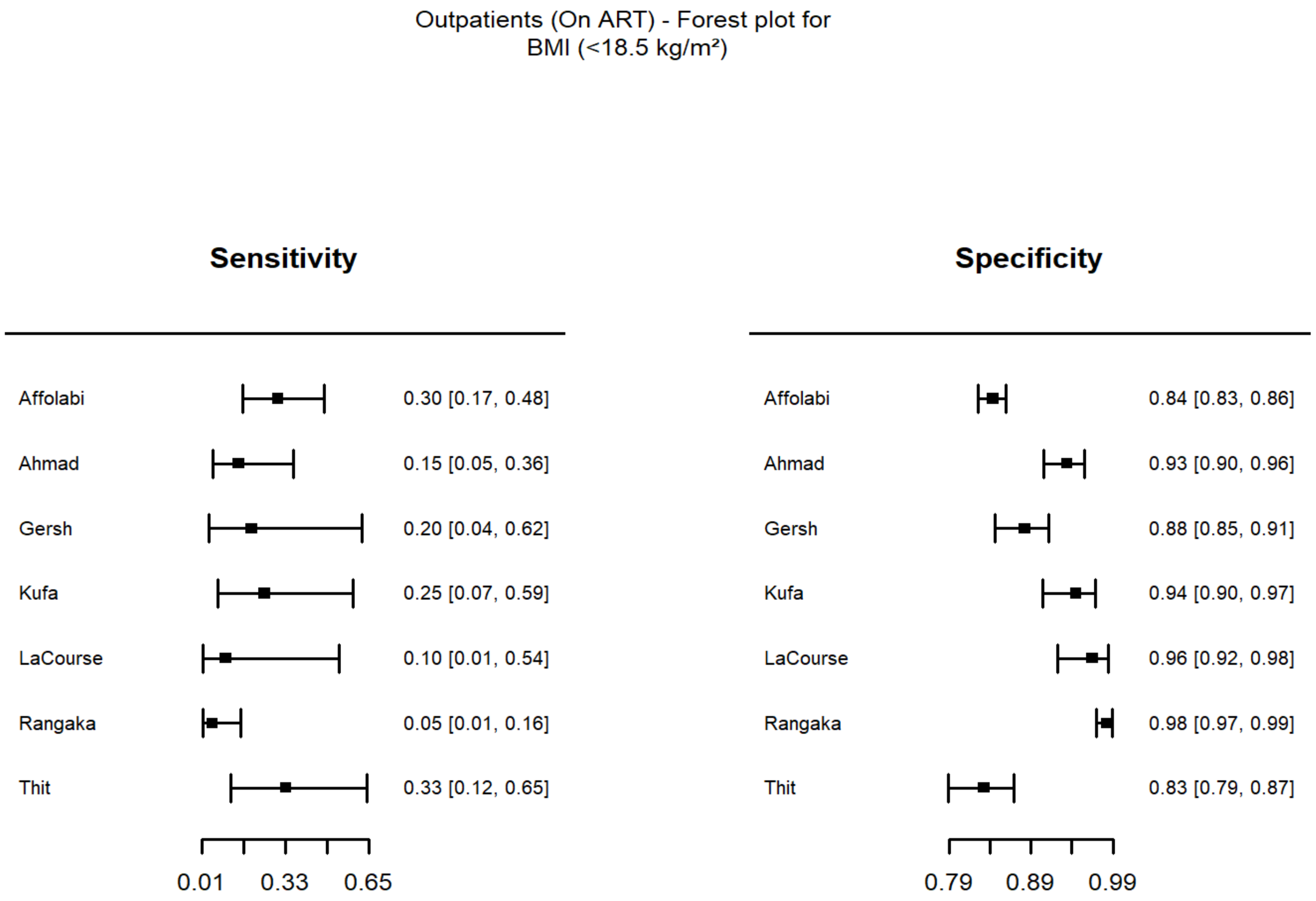
Outpatients (On ART) - Forest plot for
BMI (<18.5 kg/m²)
Sensitivity
Specificity
Affolabi
0.30 [0.17, 0.48]
Ahmad
0.15 [0.05, 0.36]
Gersh
0.20 [0.04, 0.62]
Kufa
0.25 [0.07, 0.59]
LaCourse
0.10 [0.01, 0.54]
Rangaka
0.05 [0.01, 0.16]
Thit
0.33 [0.12, 0.65]
0.01
0.33
0.65
Affolabi
0.84 [0.83, 0.86]
Ahmad
0.93 [0.90, 0.96]
Gersh
0.88 [0.85, 0.91]
Kufa
0.94 [0.90, 0.97]
LaCourse
0.96 [0.92, 0.98]
Rangaka
0.98 [0.97, 0.99]
Thit
0.83 [0.79, 0.87]
0.79
0.89
0.99

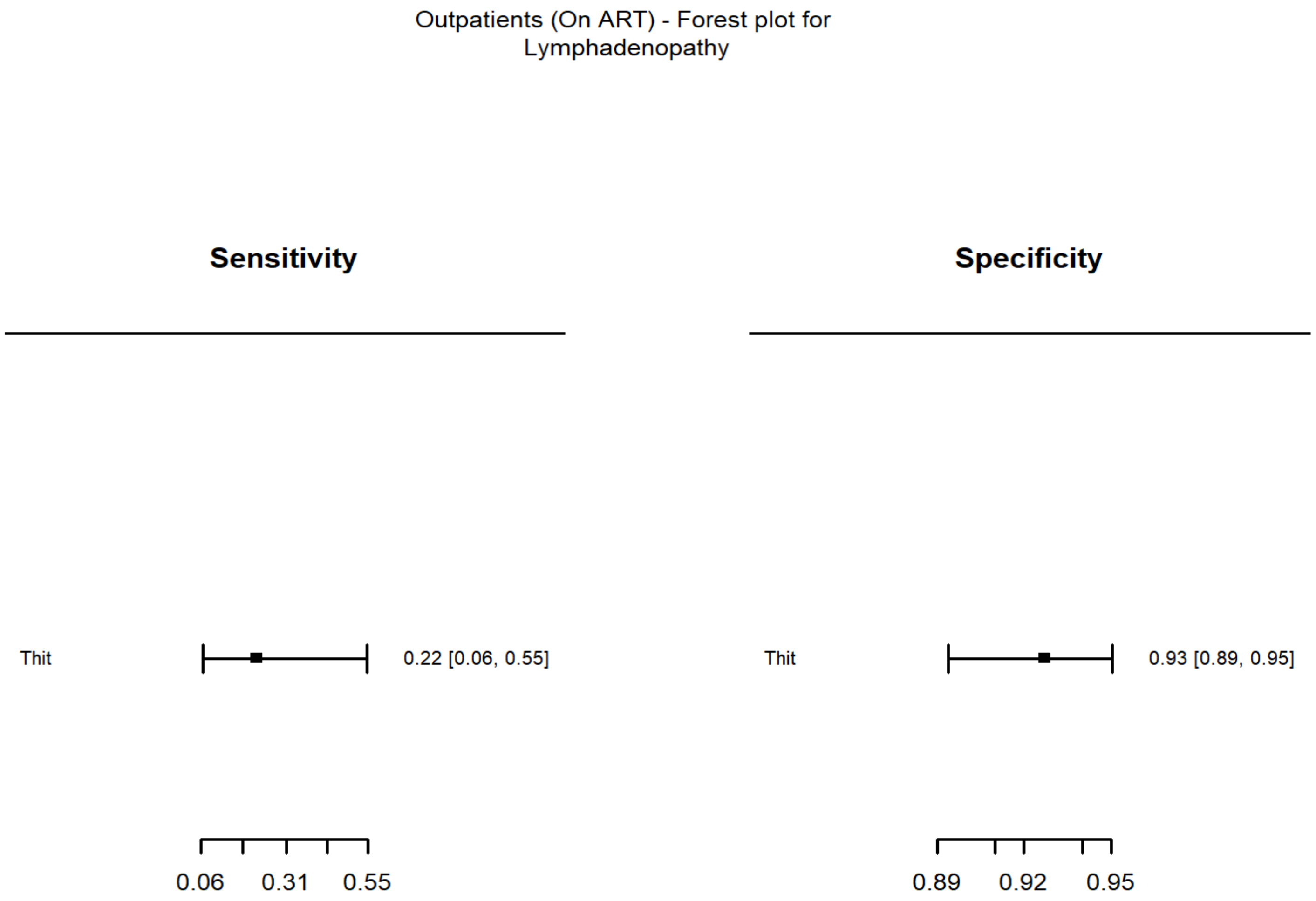
Outpatients (On ART) - Forest plot for Lymphadenopathy
Sensitivity
Specificity
Thit
0.22 [0.06, 0.55]
Thit
0.93 [0.89, 0.95]
0.06
0.31
0.55
0.89
0.92
0.95

Outpatients (On ART) - Forest plot for
Top: W4SS with CRP (>=10 mg/L) and Bottom: W4SS then CRP (>=5 mg/L)

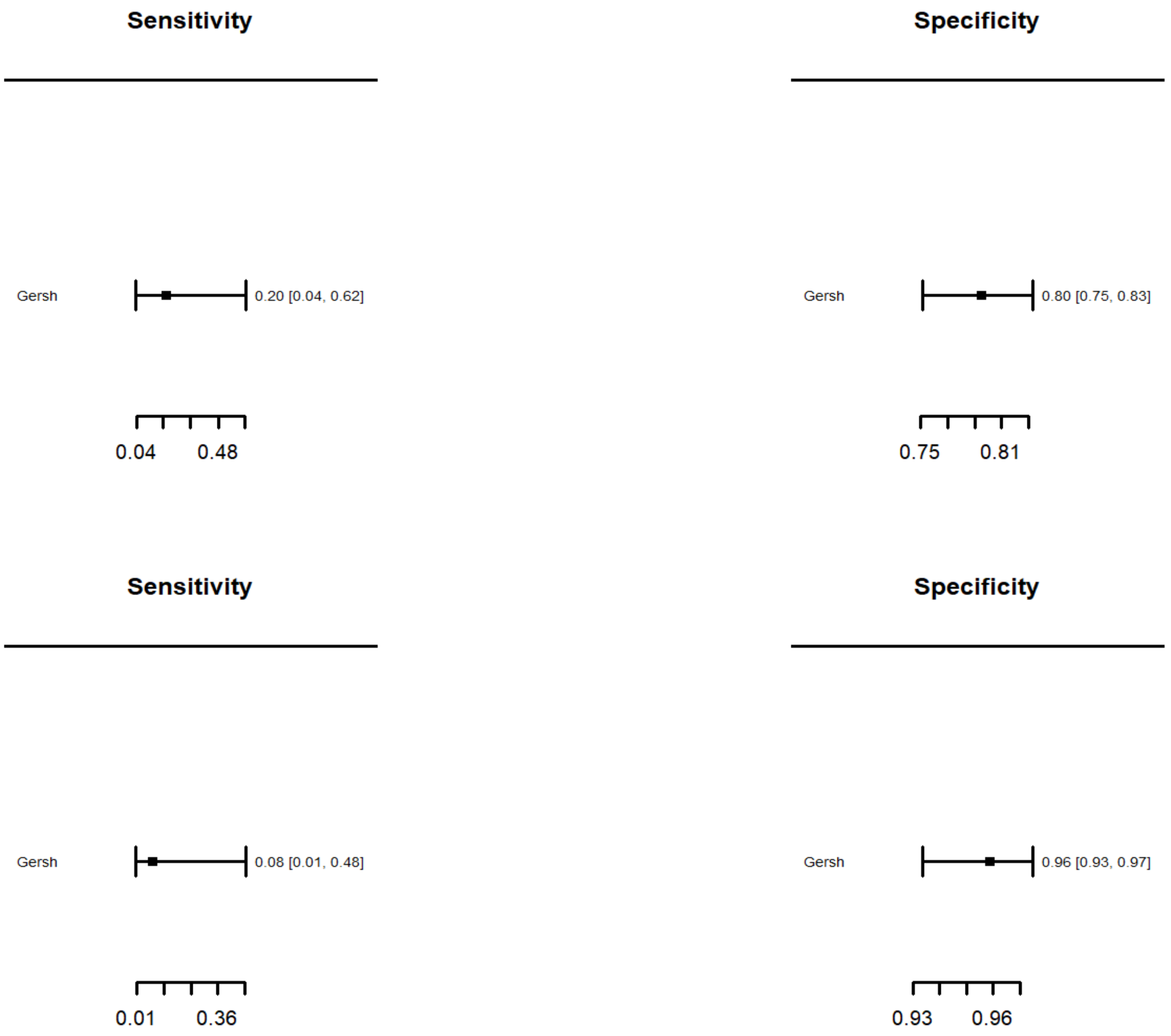

Outpatients (On ART) - Forest plot for
W4SS with CXR (abnormal)

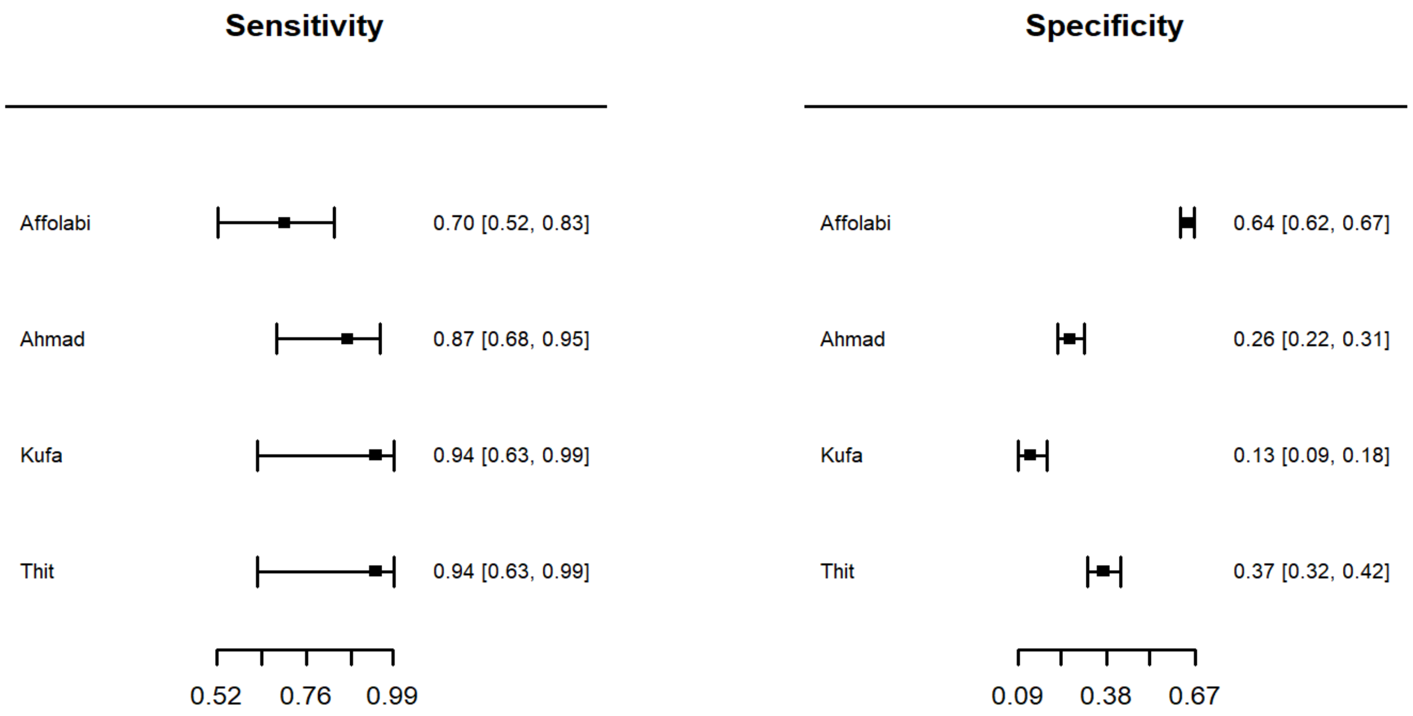

Outpatients (On ART) - Forest plot for
Top: W4SS then Xpert and Bottom: Xpert alone

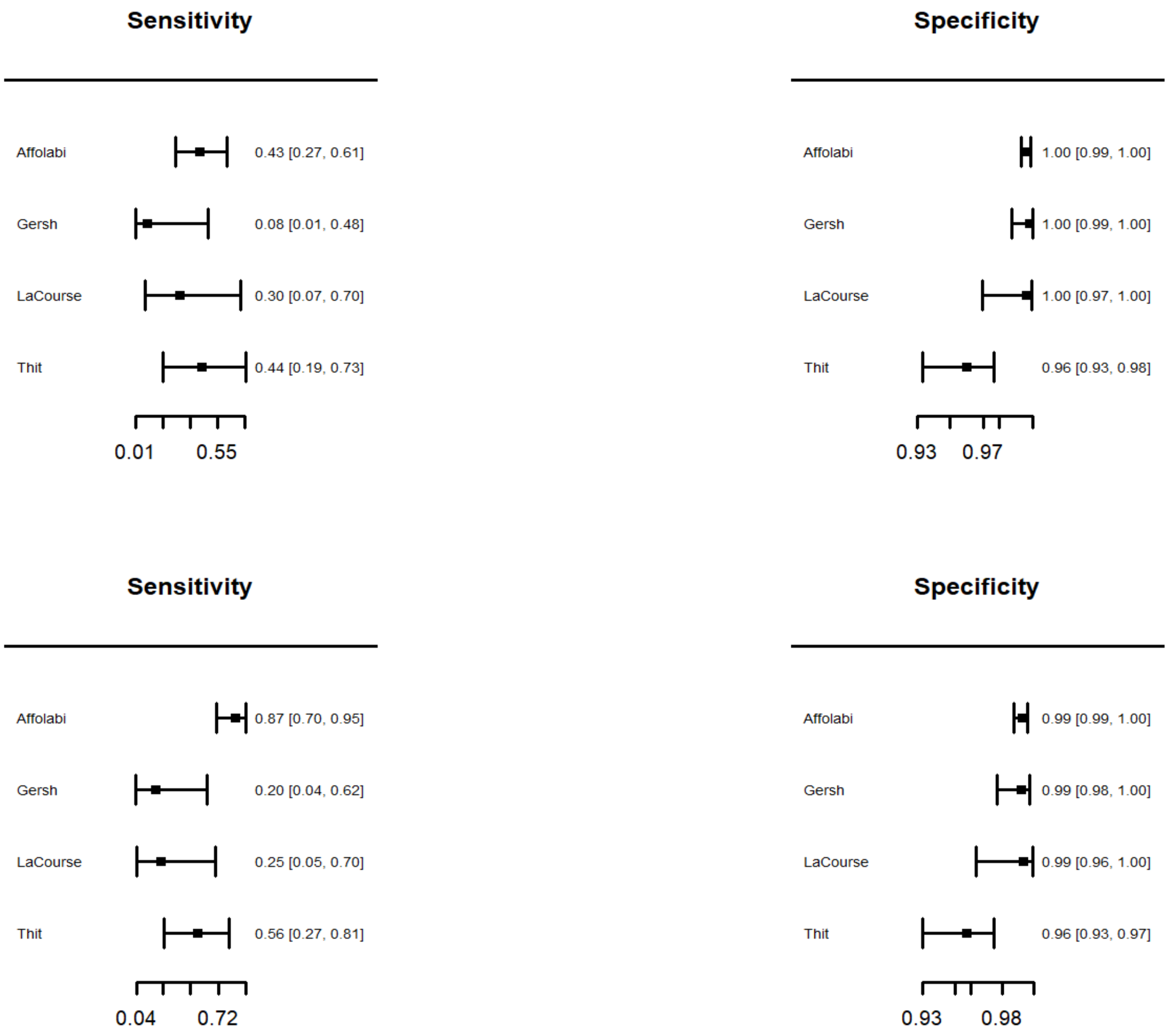

Figure S3C - Forest plots of sensitivity and specificity estimates in outpatients (not on ART)

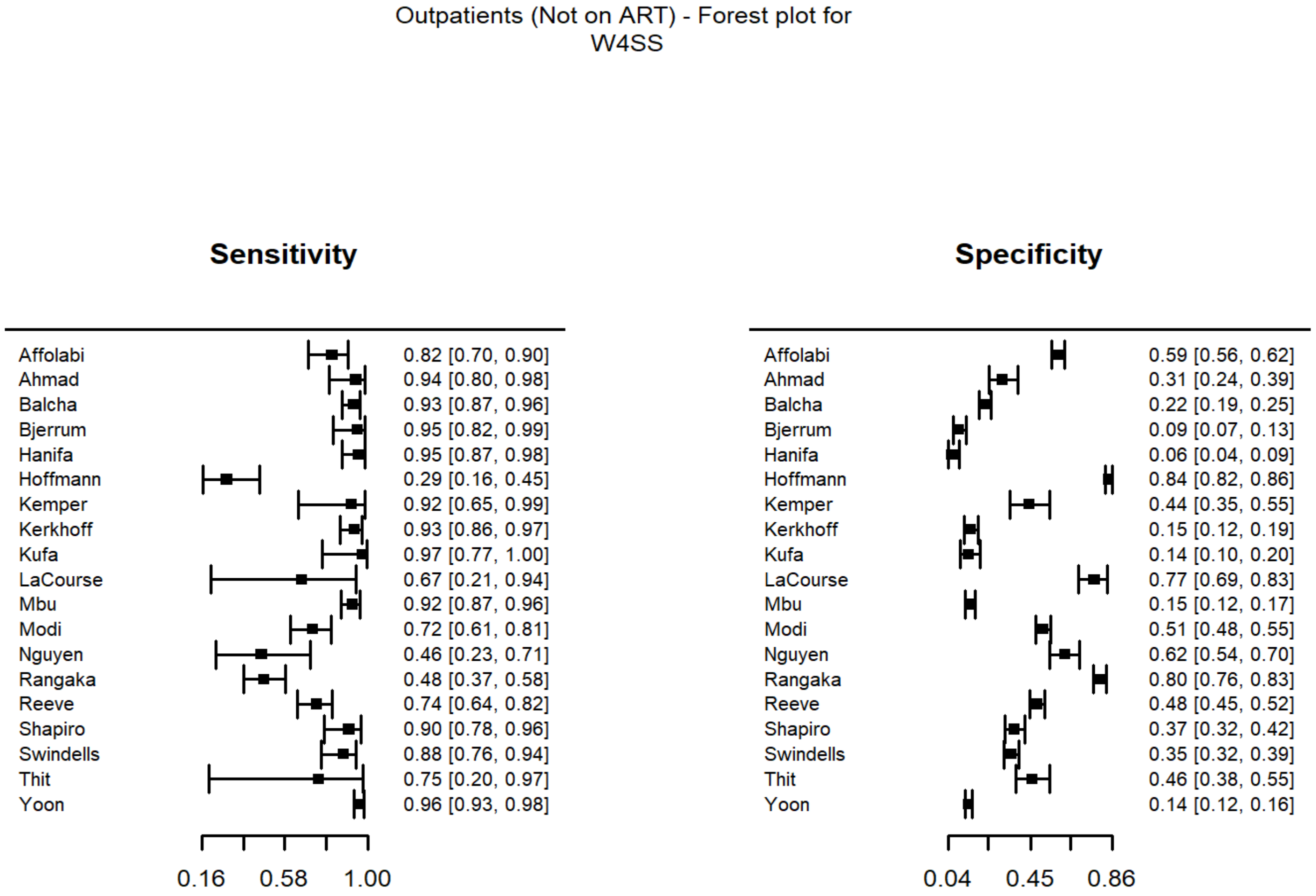

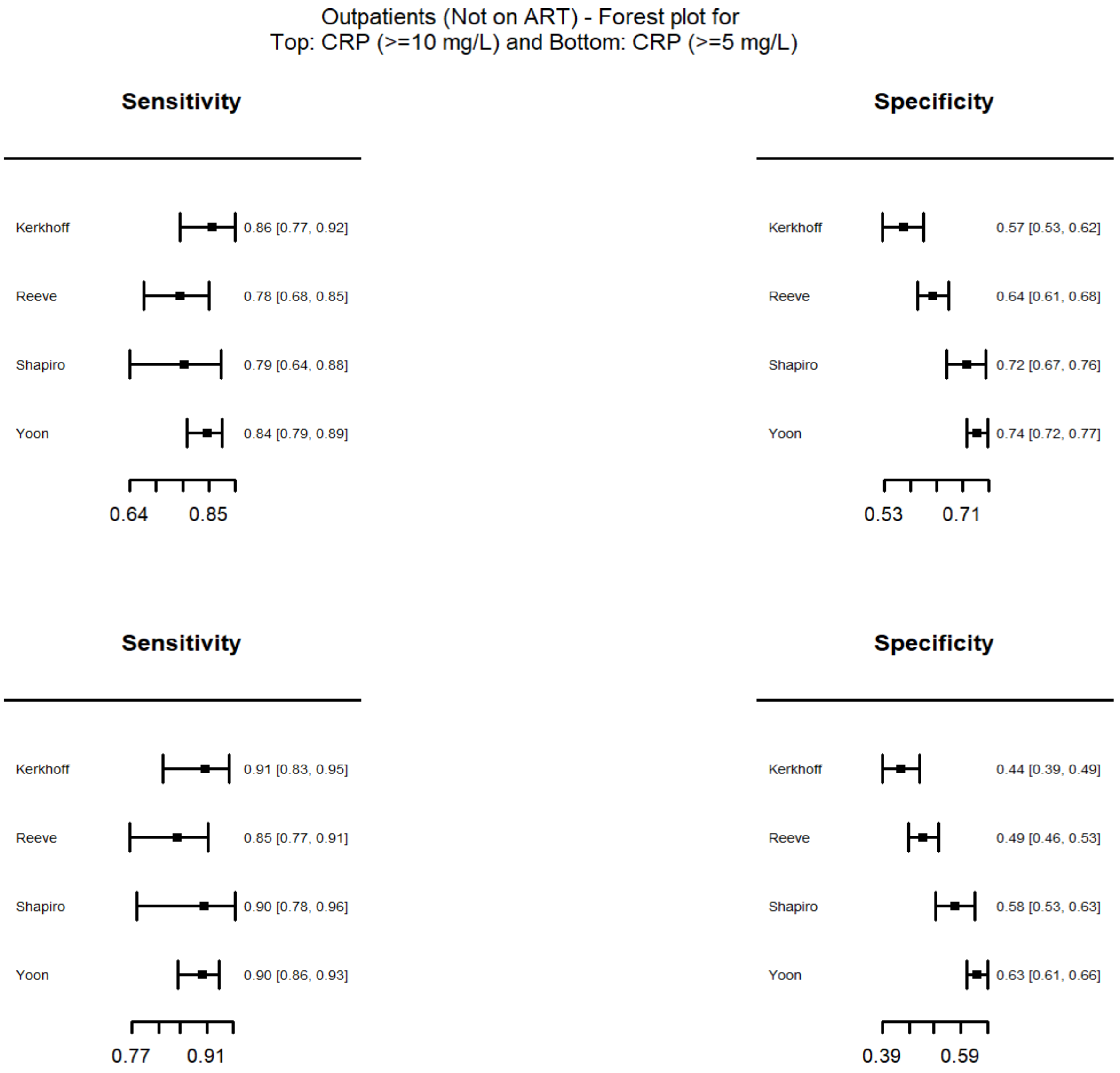
Outpatients (Not on ART) - Forest plot for
Top: CRP (>=10 mg/L) and Bottom: CRP (>=5 mg/L)
Sensitivity
Kerkhoff 0.86 [0.77, 0.92]
Reeve 0.78 [0.68, 0.85]
Shapiro 0.79 [0.64, 0.88]
Yoon 0.84 [0.79, 0.89]
0.64 0.85
Specificity
Kerkhoff 0.57 [0.53, 0.62]
Reeve 0.64 [0.61, 0.68]
Shapiro 0.72 [0.67, 0.76]
Yoon 0.74 [0.72, 0.77]
0.53 0.71
Sensitivity
Kerkhoff 0.91 [0.83, 0.95]
Reeve 0.85 [0.77, 0.91]
Shapiro 0.90 [0.78, 0.96]
Yoon 0.90 [0.86, 0.93]
0.77 0.91
Specificity
Kerkhoff 0.44 [0.39, 0.49]
Reeve 0.49 [0.46, 0.53]
Shapiro 0.58 [0.53, 0.63]
Yoon 0.63 [0.61, 0.66]
0.39 0.59

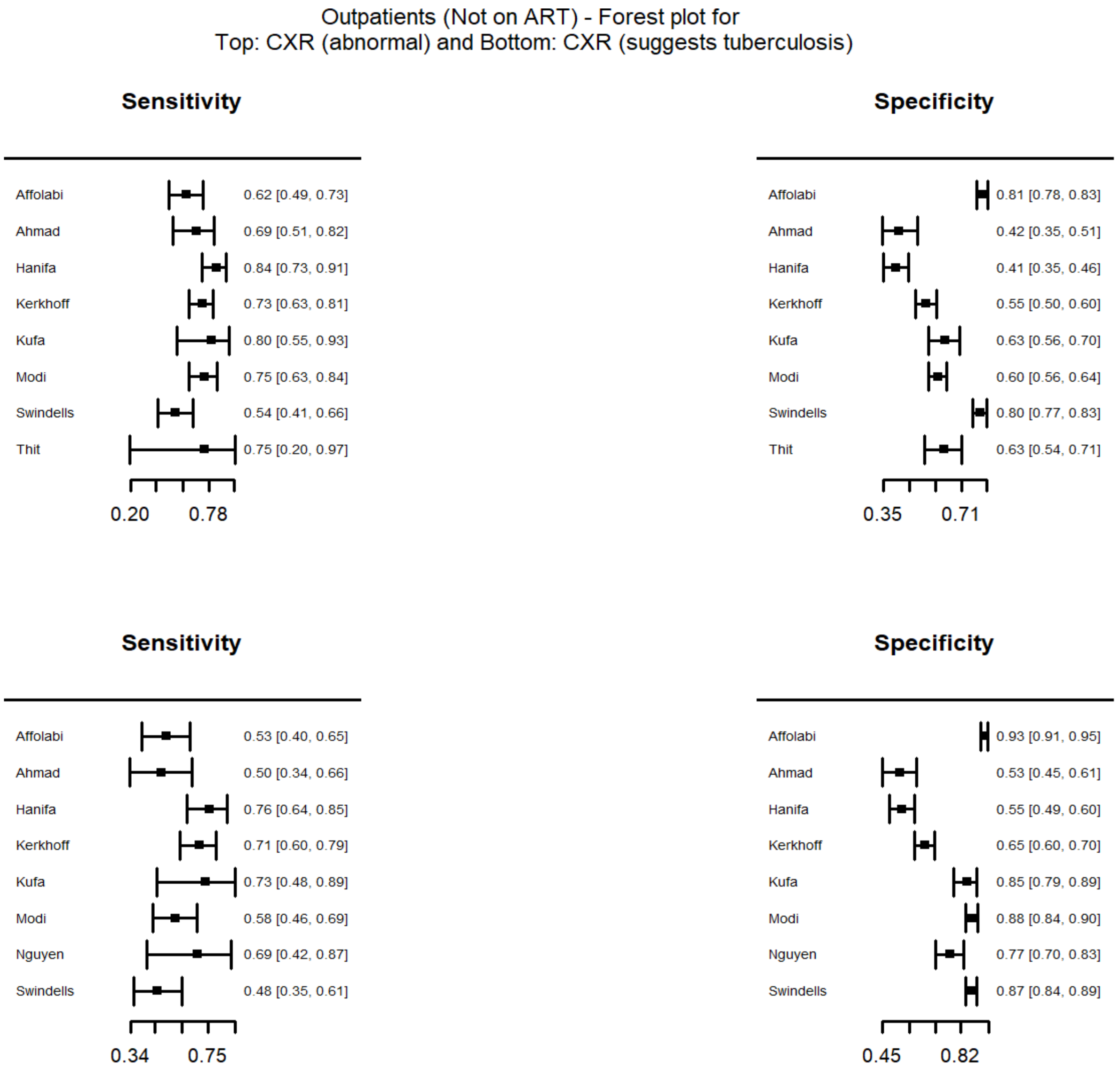
Outpatients (Not on ART) - Forest plot for
Top: CXR (abnormal) and Bottom: CXR (suggests tuberculosis)
Sensitivity
Affolabi 0.62 [0.49, 0.73]
Ahmad 0.69 [0.51, 0.82]
Hanifa 0.84 [0.73, 0.91]
Kerkhoff 0.73 [0.63, 0.81]
Kufa 0.80 [0.55, 0.93]
Modi 0.75 [0.63, 0.84]
Swindells 0.54 [0.41, 0.66]
Thit 0.75 [0.20, 0.97]
0.20 0.78
Specificity
Affolabi 0.81 [0.78, 0.83]
Ahmad 0.42 [0.35, 0.51]
Hanifa 0.41 [0.35, 0.46]
Kerkhoff 0.55 [0.50, 0.60]
Kufa 0.63 [0.56, 0.70]
Modi 0.60 [0.56, 0.64]
Swindells 0.80 [0.77, 0.83]
Thit 0.63 [0.54, 0.71]
0.35 0.71
Sensitivity
Affolabi 0.53 [0.40, 0.65]
Ahmad 0.50 [0.34, 0.66]
Hanifa 0.76 [0.64, 0.85]
Kerkhoff 0.71 [0.60, 0.79]
Kufa 0.73 [0.48, 0.89]
Modi 0.58 [0.46, 0.69]
Nguyen 0.69 [0.42, 0.87]
Swindells 0.48 [0.35, 0.61]
0.34 0.75
Specificity
Affolabi 0.93 [0.91, 0.95]
Ahmad 0.53 [0.45, 0.61]
Hanifa 0.55 [0.49, 0.60]
Kerkhoff 0.65 [0.60, 0.70]
Kufa 0.85 [0.79, 0.89]
Modi 0.88 [0.84, 0.90]
Nguyen 0.77 [0.70, 0.83]
Swindells 0.87 [0.84, 0.89]
0.45 0.82

Outpatients (Not on ART) - Forest plot for
Top: Cough (any) and Bottom: Cough (>=2 weeks)

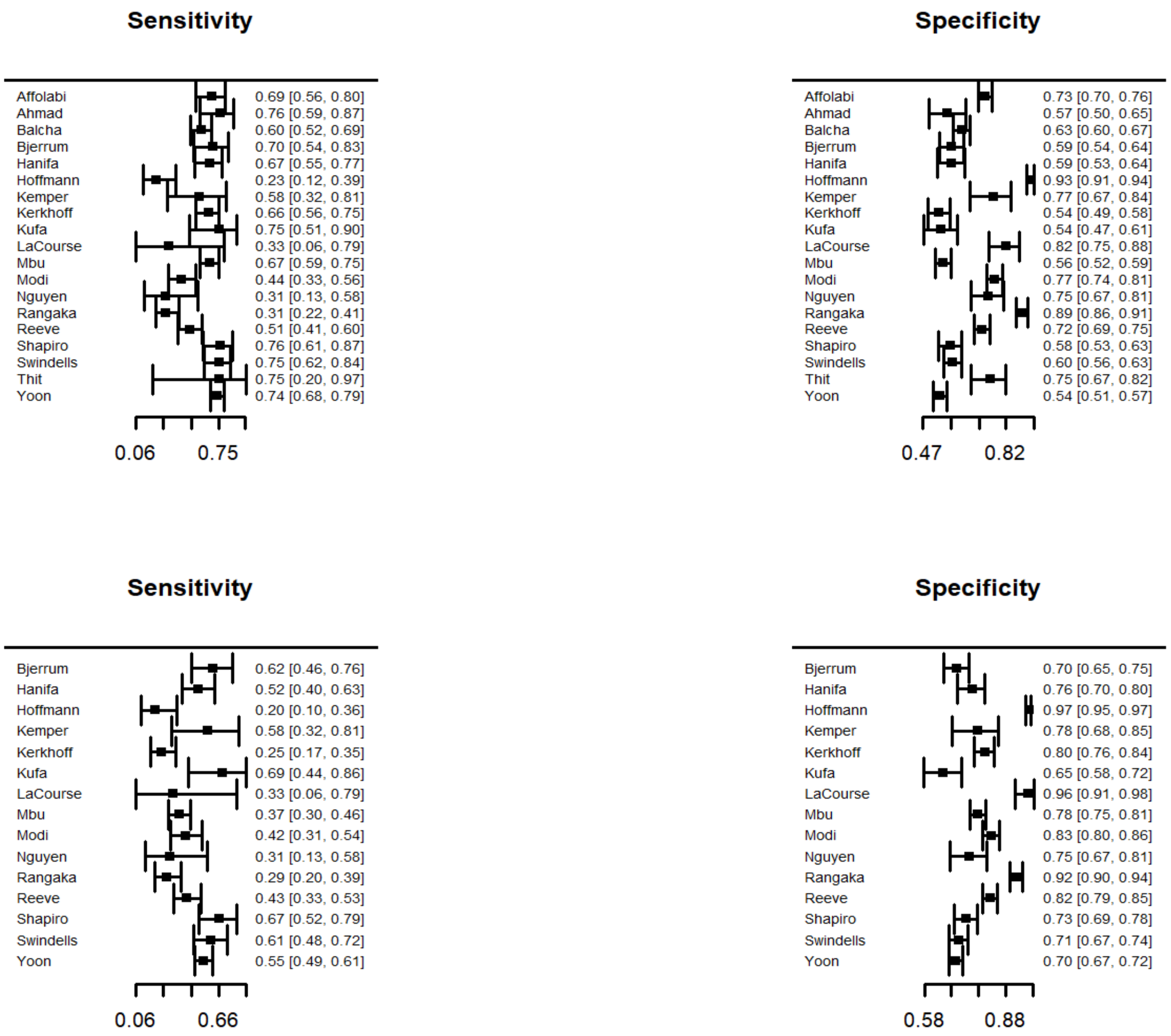

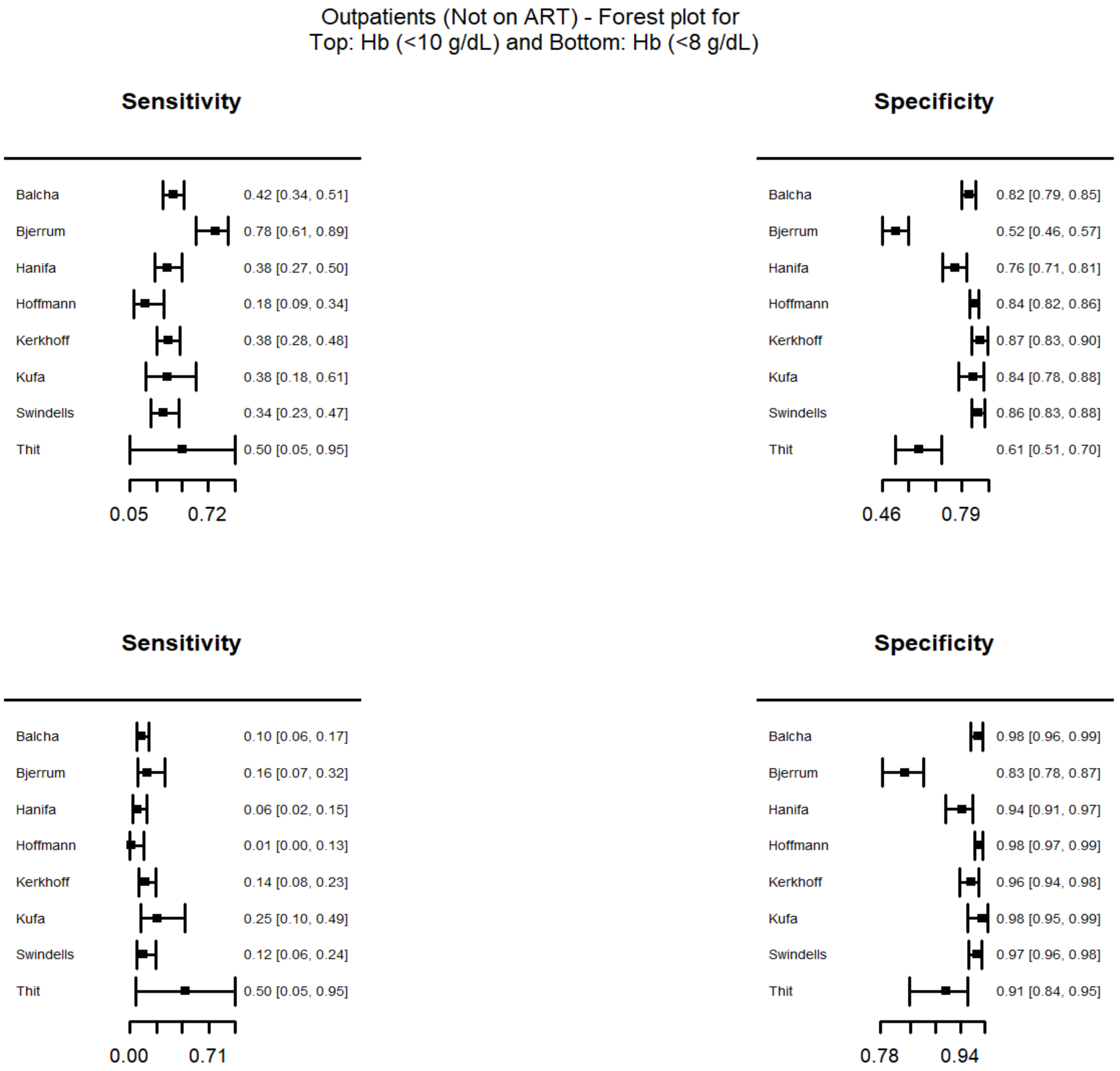
Outpatients (Not on ART) - Forest plot for
Top: Hb (<10 g/dL) and Bottom: Hb (<8 g/dL)
Sensitivity
Balcha 0.42 [0.34, 0.51]
Bjerrum 0.78 [0.61, 0.89]
Hanifa 0.38 [0.27, 0.50]
Hoffmann 0.18 [0.09, 0.34]
Kerkhoff 0.38 [0.28, 0.48]
Kufa 0.38 [0.18, 0.61]
Swindells 0.34 [0.23, 0.47]
Thit 0.50 [0.05, 0.95]
0.05 0.72
Specificity
Balcha 0.82 [0.79, 0.85]
Bjerrum 0.52 [0.46, 0.57]
Hanifa 0.76 [0.71, 0.81]
Hoffmann 0.84 [0.82, 0.86]
Kerkhoff 0.87 [0.83, 0.90]
Kufa 0.84 [0.78, 0.88]
Swindells 0.86 [0.83, 0.88]
Thit 0.61 [0.51, 0.70]
0.46 0.79
Sensitivity
Balcha 0.10 [0.06, 0.17]
Bjerrum 0.16 [0.07, 0.32]
Hanifa 0.06 [0.02, 0.15]
Hoffmann 0.01 [0.00, 0.13]
Kerkhoff 0.14 [0.08, 0.23]
Kufa 0.25 [0.10, 0.49]
Swindells 0.12 [0.06, 0.24]
Thit 0.50 [0.05, 0.95]
0.00 0.71
Specificity
Balcha 0.98 [0.96, 0.99]
Bjerrum 0.83 [0.78, 0.87]
Hanifa 0.94 [0.91, 0.97]
Hoffmann 0.98 [0.97, 0.99]
Kerkhoff 0.96 [0.94, 0.98]
Kufa 0.98 [0.95, 0.99]
Swindells 0.97 [0.96, 0.98]
Thit 0.91 [0.84, 0.95]
0.78 0.94

Outpatients (Not on ART) - Forest plot for
BMI (<18.5 kg/m²)

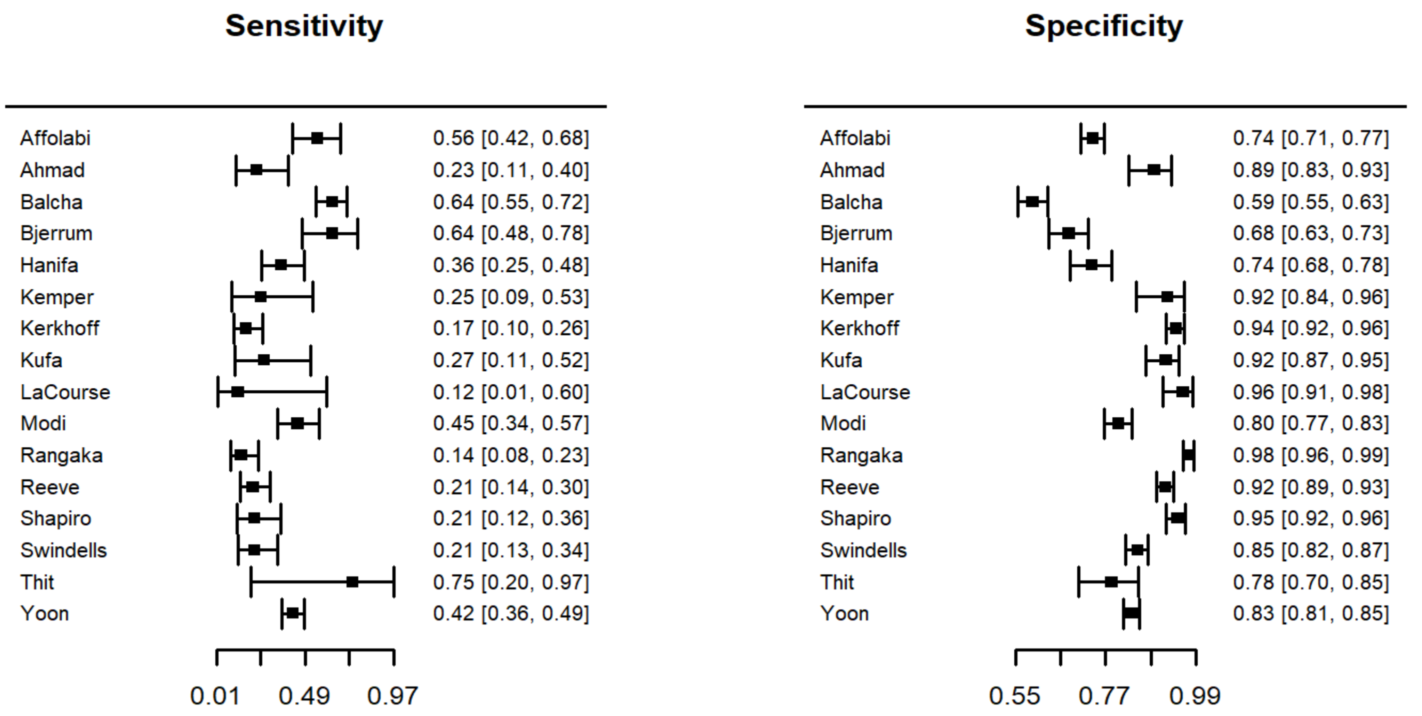

Outpatients (Not on ART) - Forest plot for Lymphadenopathy

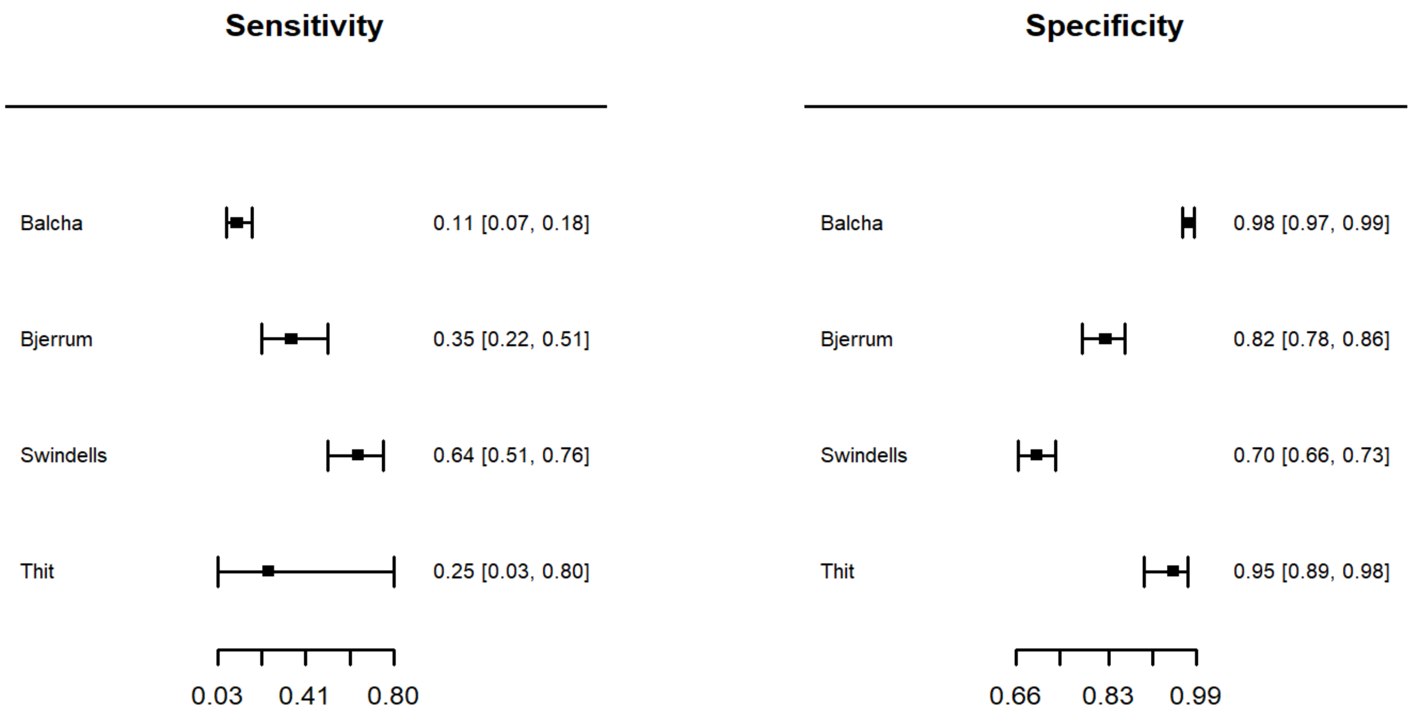

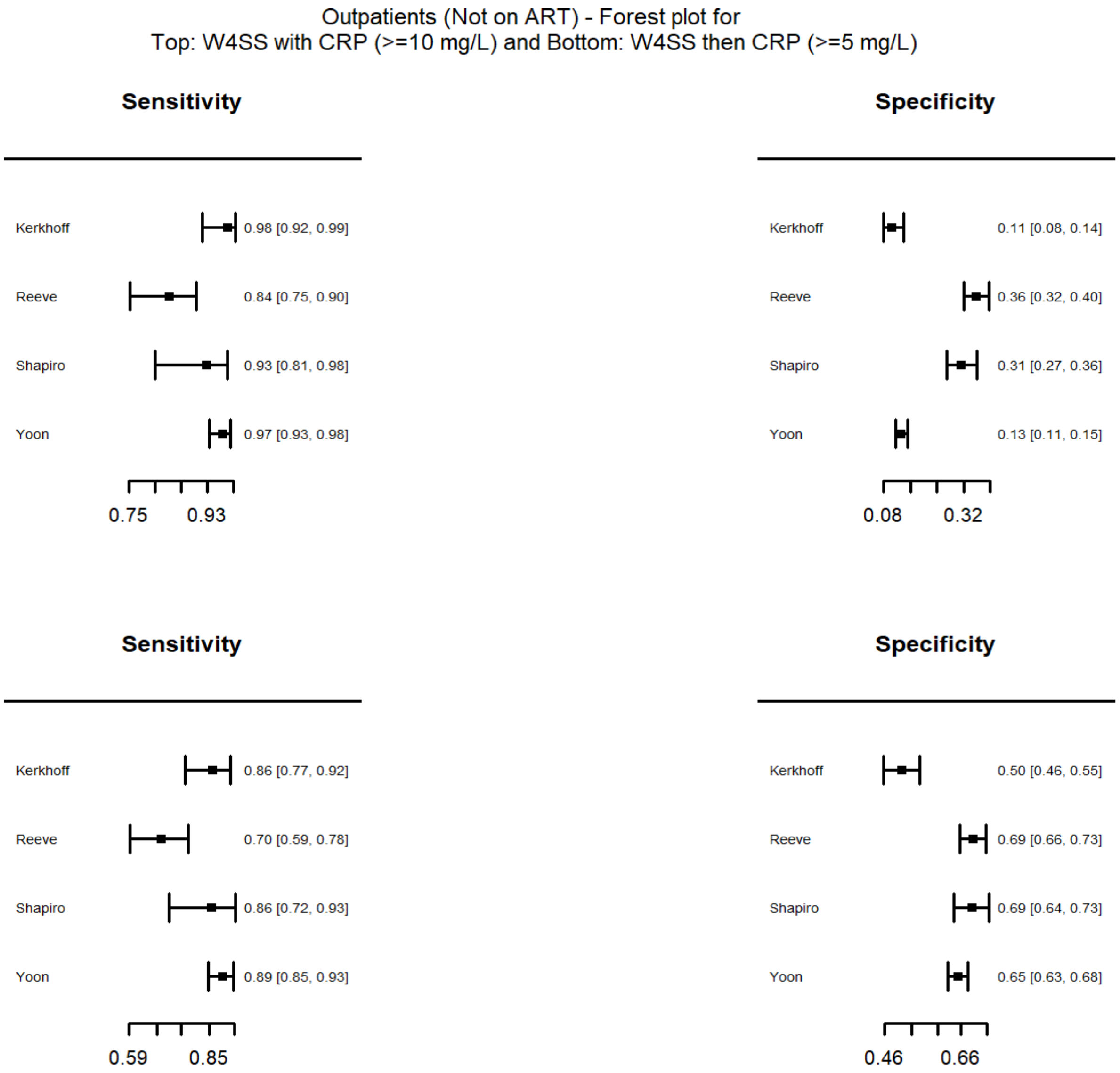
Outpatients (Not on ART) - Forest plot for
Top: W4SS with CRP (>=10 mg/L) and Bottom: W4SS then CRP (>=5 mg/L)
Sensitivity
Kerkhoff 0.98 [0.92, 0.99]
Reeve 0.84 [0.75, 0.90]
Shapiro 0.93 [0.81, 0.98]
Yoon 0.97 [0.93, 0.98]
0.75 0.93
Specificity
Kerkhoff 0.11 [0.08, 0.14]
Reeve 0.36 [0.32, 0.40]
Shapiro 0.31 [0.27, 0.36]
Yoon 0.13 [0.11, 0.15]
0.08 0.32
Sensitivity
Kerkhoff 0.86 [0.77, 0.92]
Reeve 0.70 [0.59, 0.78]
Shapiro 0.86 [0.72, 0.93]
Yoon 0.89 [0.85, 0.93]
0.59 0.85
Specificity
Kerkhoff 0.50 [0.46, 0.55]
Reeve 0.69 [0.66, 0.73]
Shapiro 0.69 [0.64, 0.73]
Yoon 0.65 [0.63, 0.68]
0.46 0.66

Outpatients (Not on ART) - Forest plot for
W4SS with CXR (abnormal)

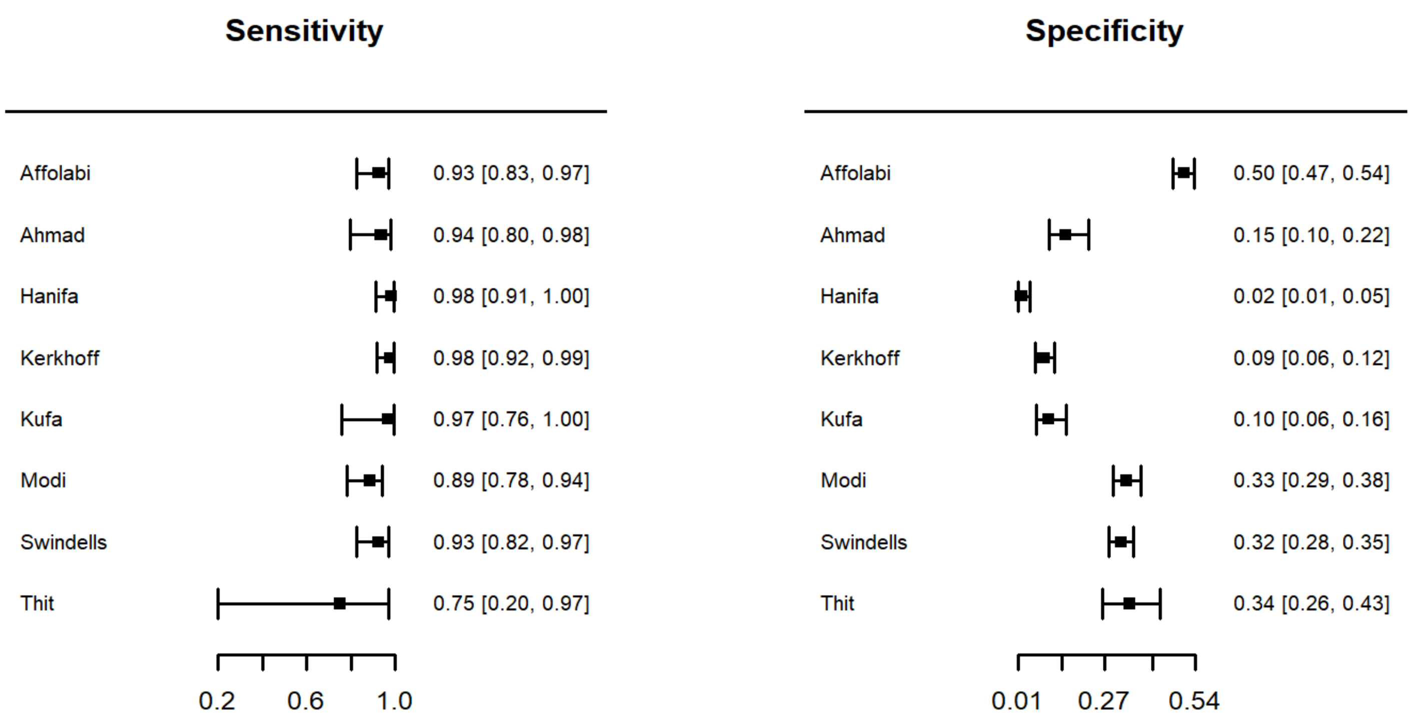

Outpatients (Not on ART) - Forest plot for
Top: W4SS then Xpert and Bottom: Xpert alone

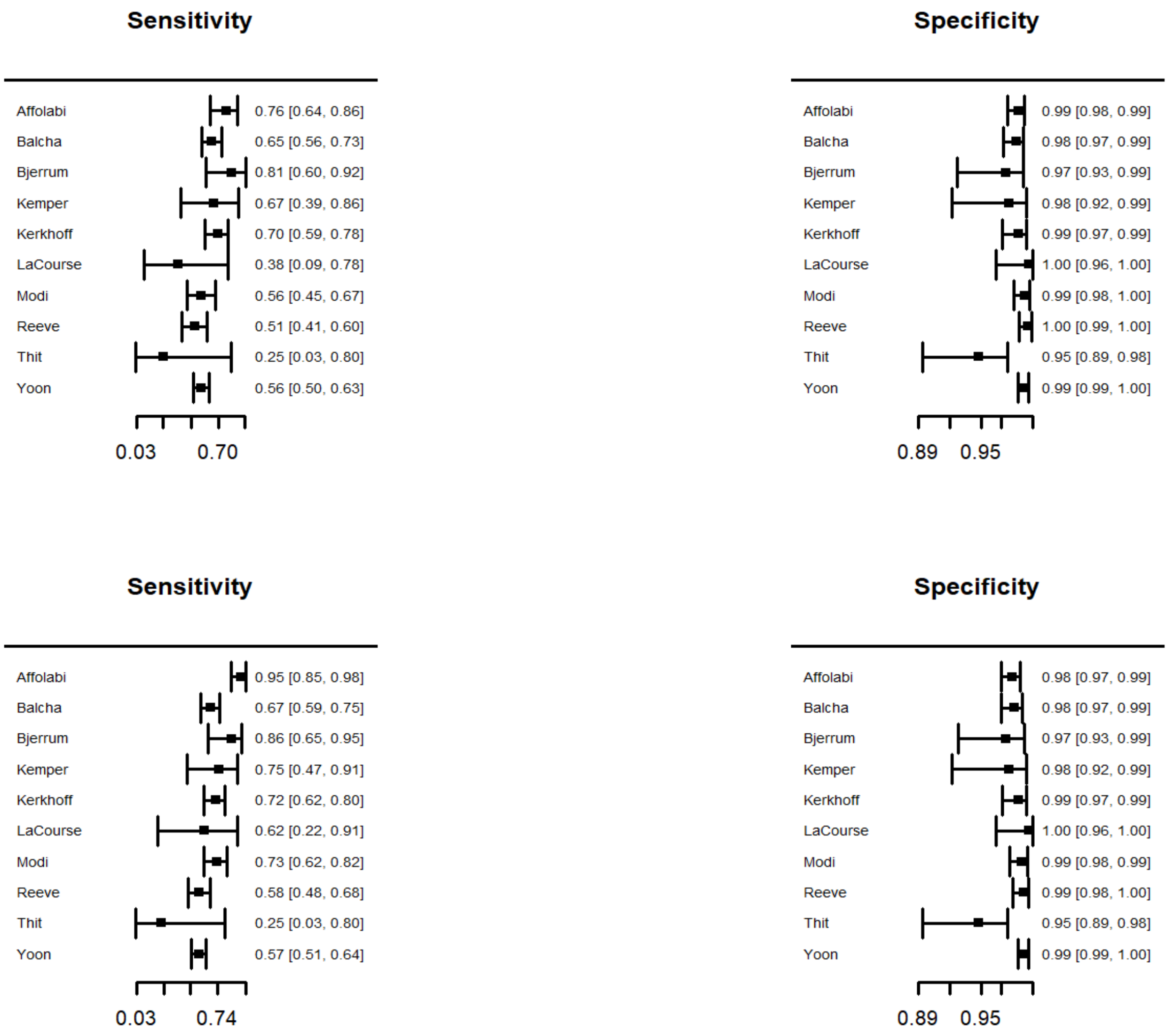

Figure S3D - Forest plots of sensitivity and specificity estimates in participants with a CD4 cell count <=200 cells/µL

CD4 <=200 cells/µL - Forest plot for
W4SS

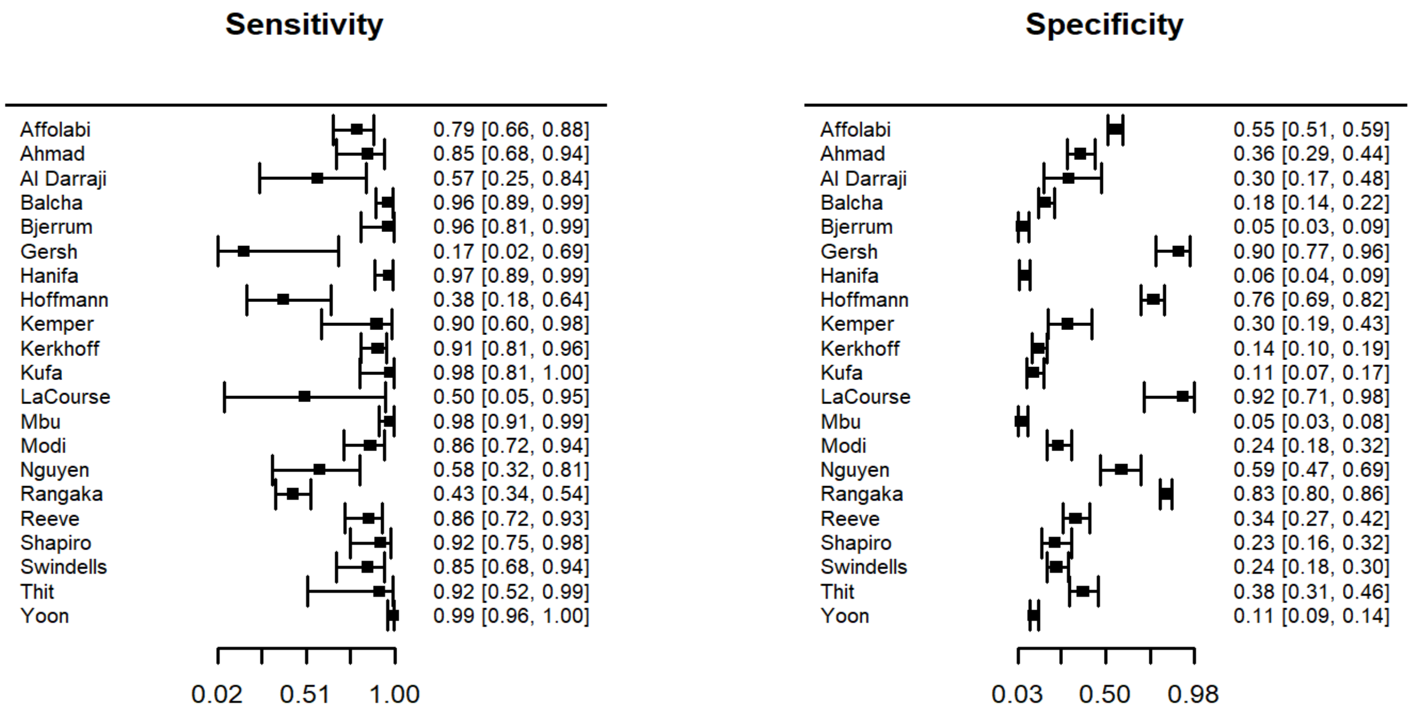

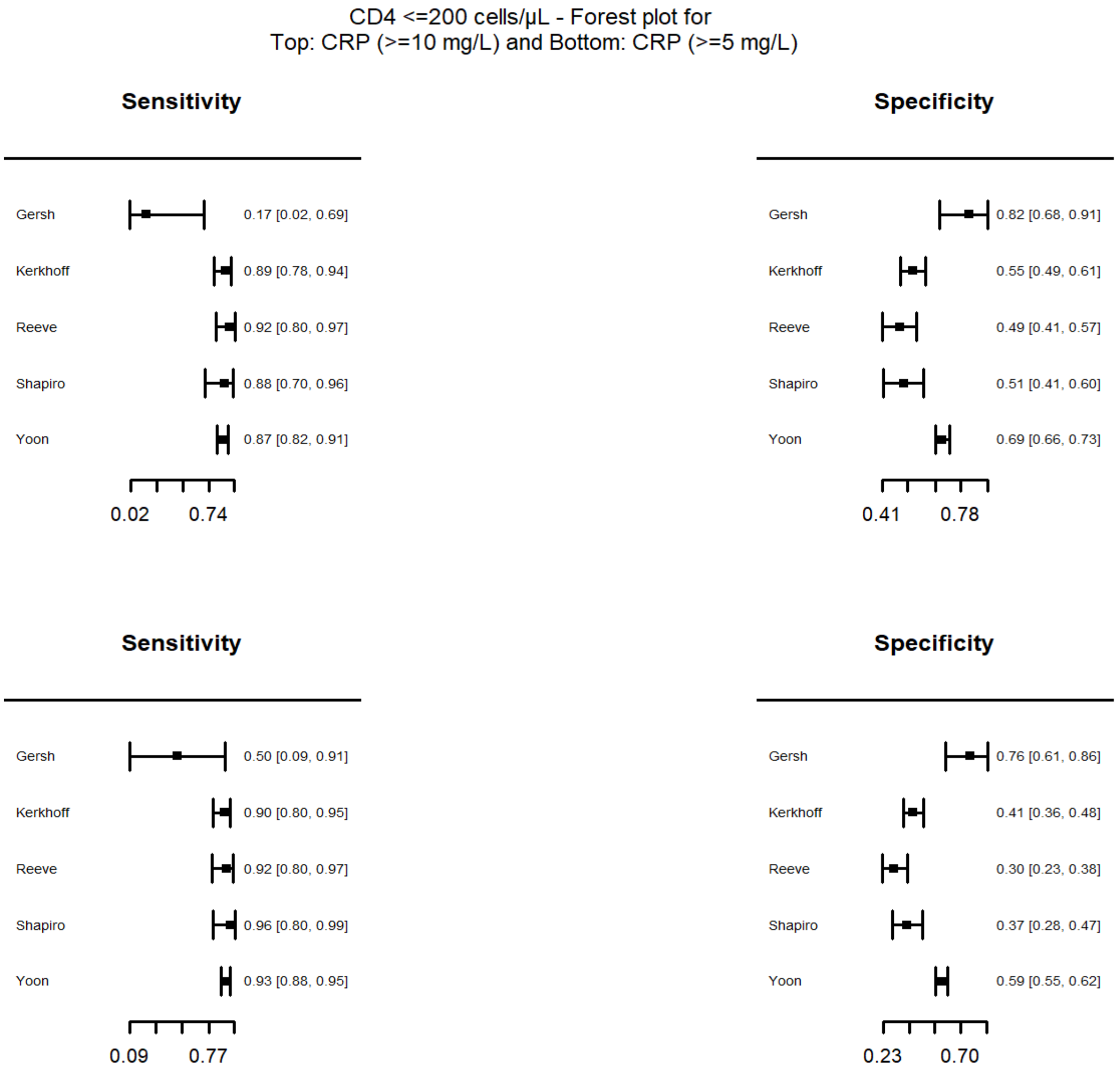
CD4 <=200 cells/μL - Forest plot for
Top: CRP (>=10 mg/L) and Bottom: CRP (>=5 mg/L)
Sensitivity
Gersh 0.17 [0.02, 0.69]
Kerkhoff 0.89 [0.78, 0.94]
Reeve 0.92 [0.80, 0.97]
Shapiro 0.88 [0.70, 0.96]
Yoon 0.87 [0.82, 0.91]
0.02 0.74
Specificity
Gersh 0.82 [0.68, 0.91]
Kerkhoff 0.55 [0.49, 0.61]
Reeve 0.49 [0.41, 0.57]
Shapiro 0.51 [0.41, 0.60]
Yoon 0.69 [0.66, 0.73]
0.41 0.78
Sensitivity
Gersh 0.50 [0.09, 0.91]
Kerkhoff 0.90 [0.80, 0.95]
Reeve 0.92 [0.80, 0.97]
Shapiro 0.96 [0.80, 0.99]
Yoon 0.93 [0.88, 0.95]
0.09 0.77
Specificity
Gersh 0.76 [0.61, 0.86]
Kerkhoff 0.41 [0.36, 0.48]
Reeve 0.30 [0.23, 0.38]
Shapiro 0.37 [0.28, 0.47]
Yoon 0.59 [0.55, 0.62]
0.23 0.70

CD4 <=200 cells/µL - Forest plot for
Top: CXR (abnormal) and Bottom: CXR (suggests tuberculosis)

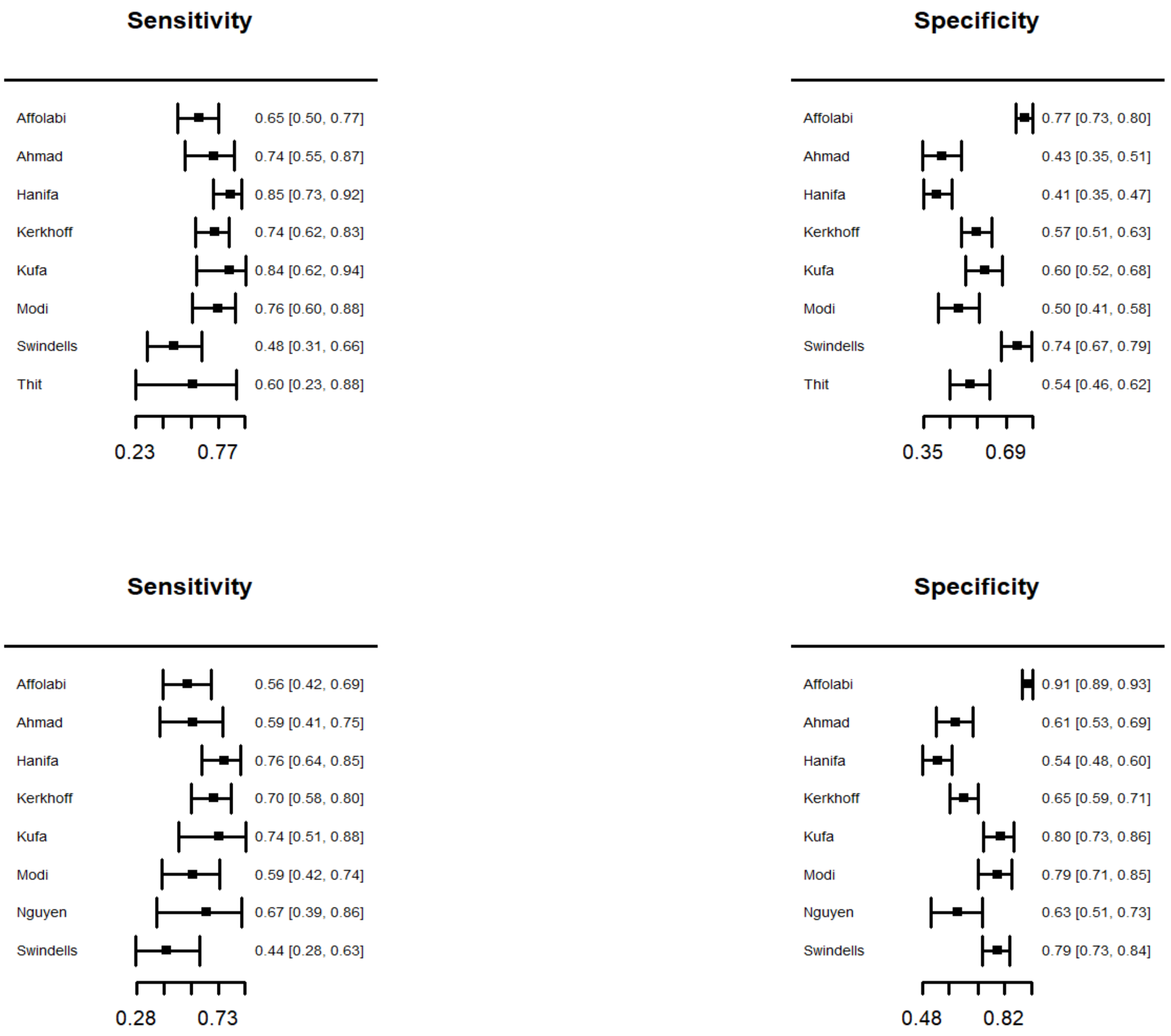

CD4 <=200 cells/µL - Forest plot for
Top: Cough (any) and Bottom: Cough (>=2 weeks)

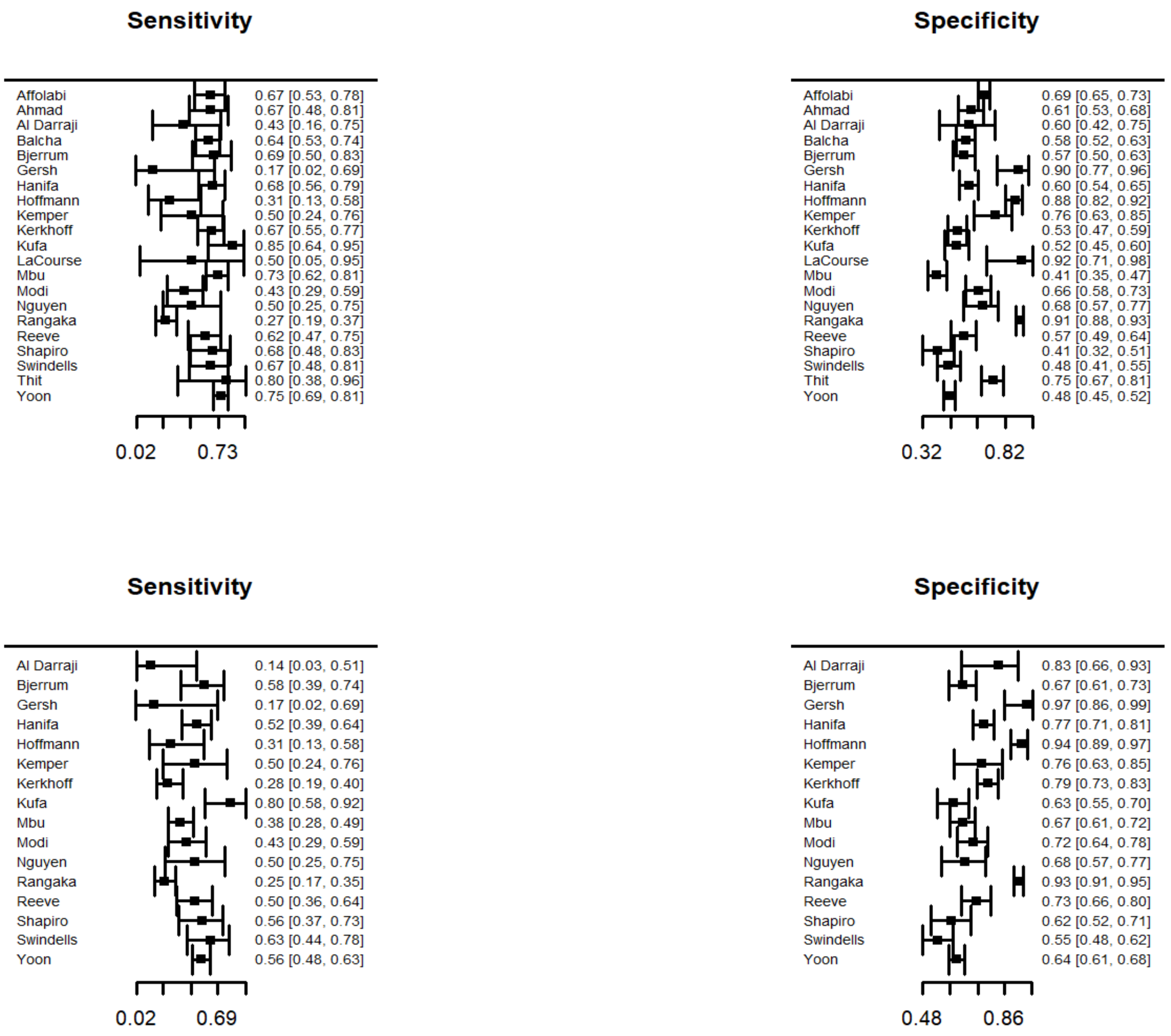

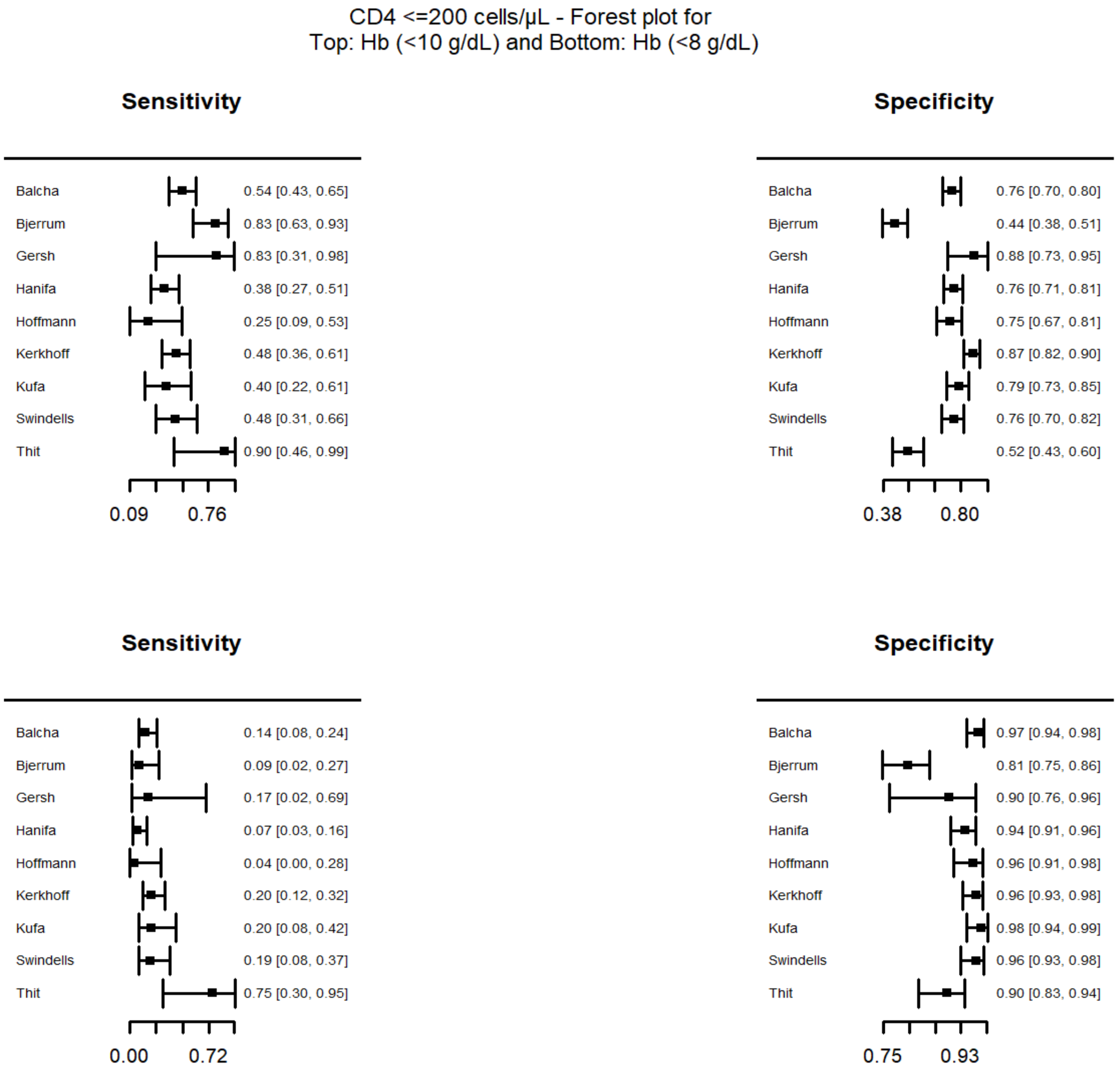

CD4 <=200 cells/µL - Forest plot for
Top: Hb (<10 g/dL) and Bottom: Hb (<8 g/dL)
Sensitivity
Balcha 0.54 [0.43, 0.65]
Bjerrum 0.83 [0.63, 0.93]
Gersh 0.83 [0.31, 0.98]
Hanifa 0.38 [0.27, 0.51]
Hoffmann 0.25 [0.09, 0.53]
Kerkhoff 0.48 [0.36, 0.61]
Kufa 0.40 [0.22, 0.61]
Swindells 0.48 [0.31, 0.66]
Thit 0.90 [0.46, 0.99]
0.09
0.76
Specificity
Balcha 0.76 [0.70, 0.80]
Bjerrum 0.44 [0.38, 0.51]
Gersh 0.88 [0.73, 0.95]
Hanifa 0.76 [0.71, 0.81]
Hoffmann 0.75 [0.67, 0.81]
Kerkhoff 0.87 [0.82, 0.90]
Kufa 0.79 [0.73, 0.85]
Swindells 0.76 [0.70, 0.82]
Thit 0.52 [0.43, 0.60]
0.38
0.80
Sensitivity
Balcha 0.14 [0.08, 0.24]
Bjerrum 0.09 [0.02, 0.27]
Gersh 0.17 [0.02, 0.69]
Hanifa 0.07 [0.03, 0.16]
Hoffmann 0.04 [0.00, 0.28]
Kerkhoff 0.20 [0.12, 0.32]
Kufa 0.20 [0.08, 0.42]
Swindells 0.19 [0.08, 0.37]
Thit 0.75 [0.30, 0.95]
0.00
0.72
Specificity
Balcha 0.97 [0.94, 0.98]
Bjerrum 0.81 [0.75, 0.86]
Gersh 0.90 [0.76, 0.96]
Hanifa 0.94 [0.91, 0.96]
Hoffmann 0.96 [0.91, 0.98]
Kerkhoff 0.96 [0.93, 0.98]
Kufa 0.98 [0.94, 0.99]
Swindells 0.96 [0.93, 0.98]
Thit 0.90 [0.83, 0.94]
0.75
0.93

CD4 <=200 cells/μL - Forest plot for
BMI (<18.5 kg/m²)

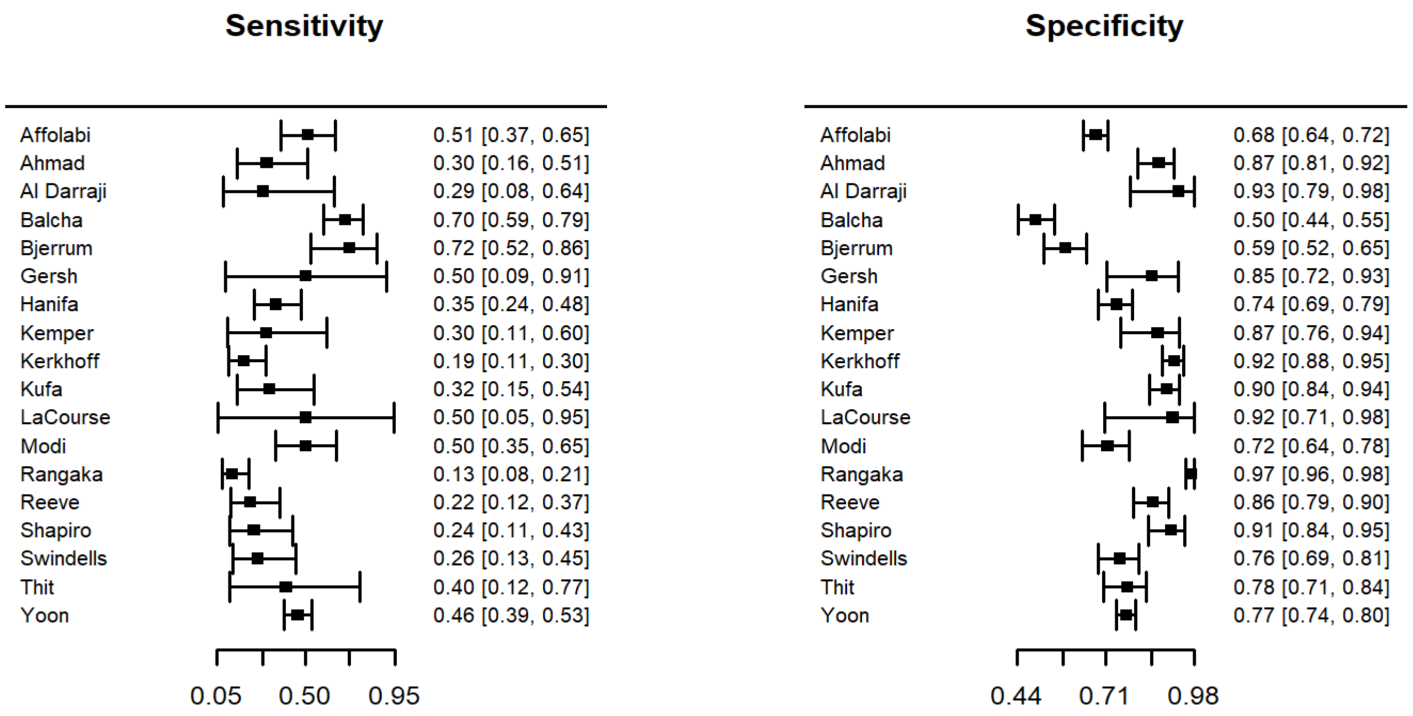

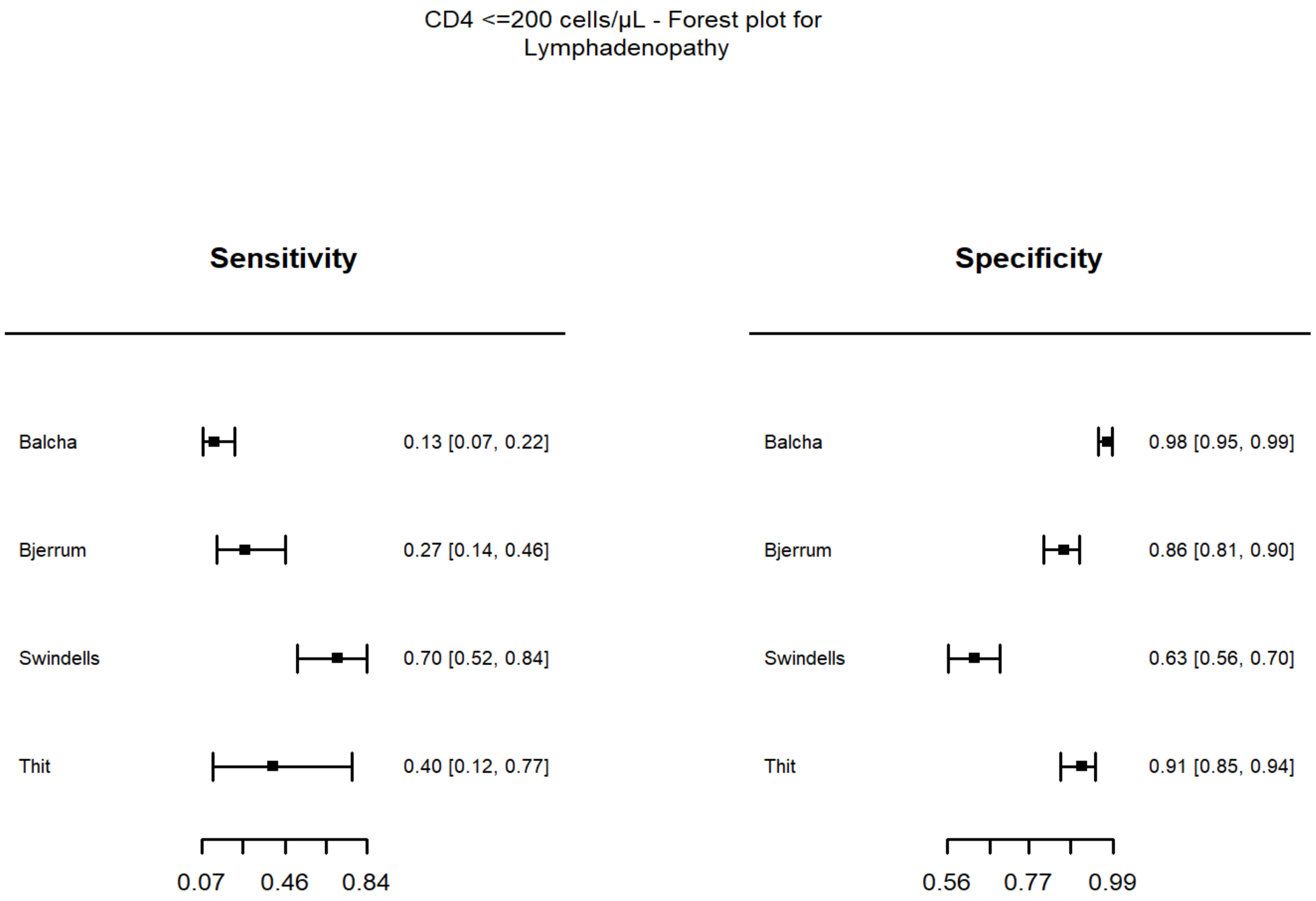
CD4 <=200 cells/μL - Forest plot for Lymphadenopathy
Sensitivity
Specificity
Balcha
0.13 [0.07, 0.22]
0.98 [0.95, 0.99]
Bjerrum
0.27 [0.14, 0.46]
0.86 [0.81, 0.90]
Swindells
0.70 [0.52, 0.84]
0.63 [0.56, 0.70]
Thit
0.40 [0.12, 0.77]
0.91 [0.85, 0.94]
0.07
0.46
0.84
0.56
0.77
0.99

CD4 <=200 cells/μL - Forest plot for
Top: W4SS with CRP (>=10 mg/L) and Bottom: W4SS then CRP (>=5 mg/L)

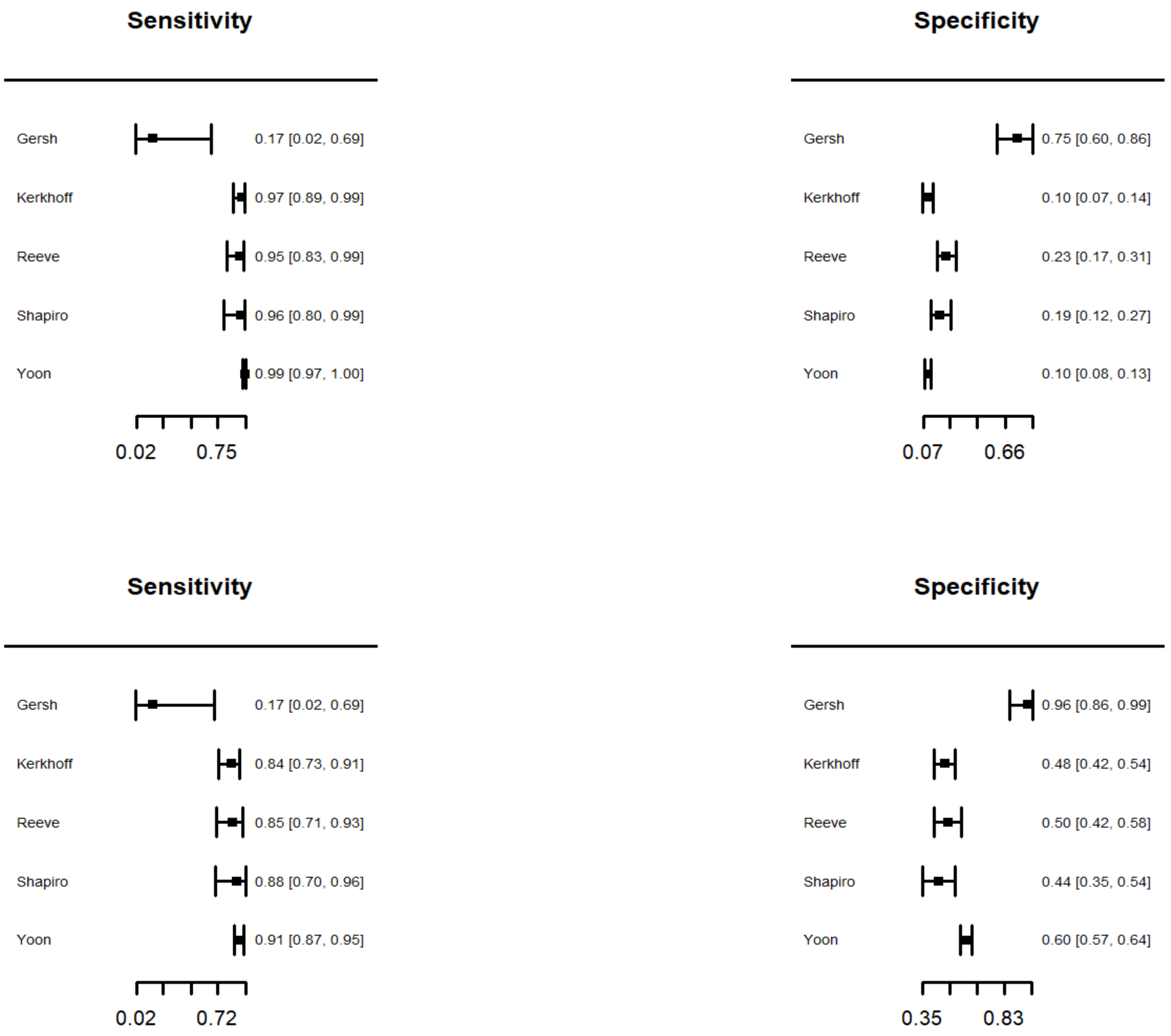

CD4 <=200 cells/µL - Forest plot for
W4SS with CXR (abnormal)

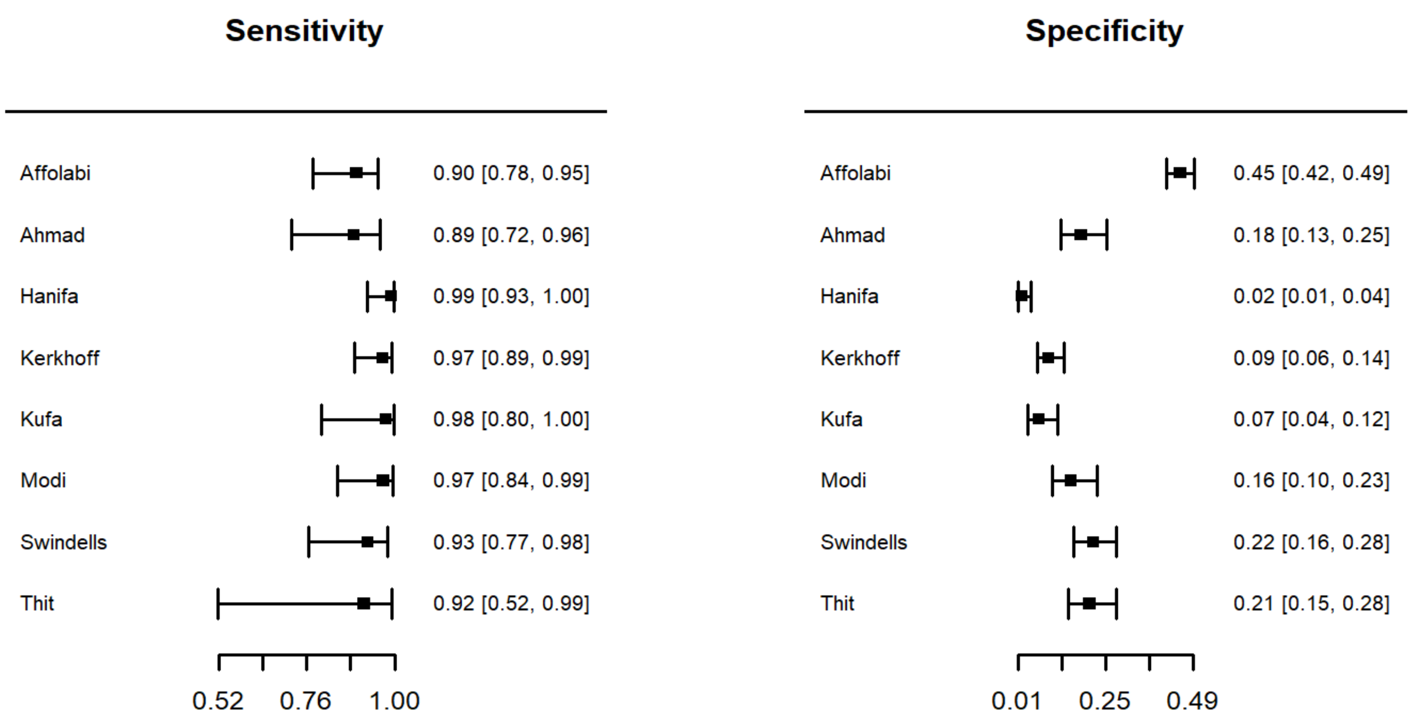

CD4 <=200 cells/μL - Forest plot for
Top: W4SS then Xpert and Bottom: Xpert alone

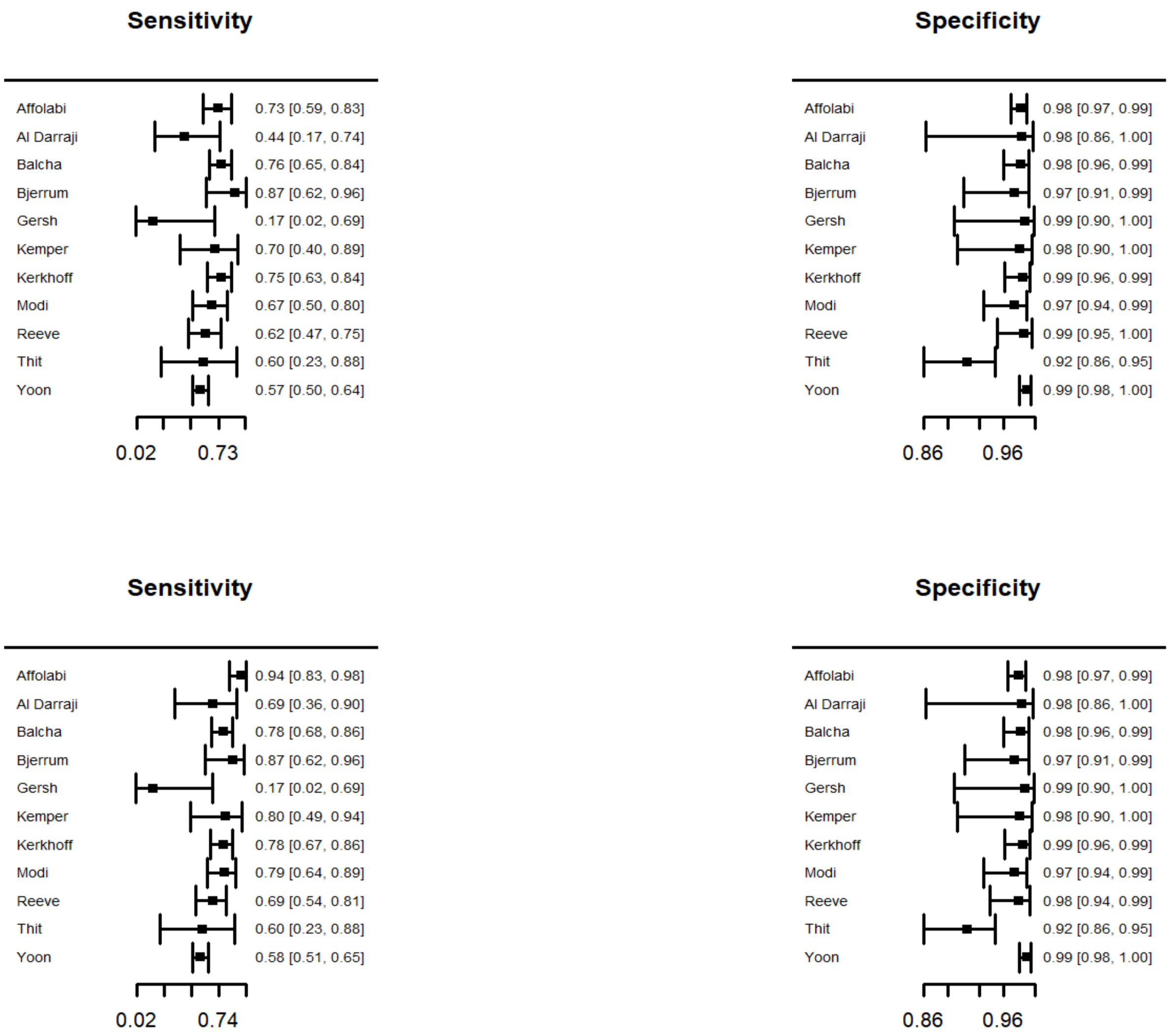

Figure S3E - Forest plots of sensitivity and specificity estimates in participants with a CD4 cell count >200 cells/µL

CD4 >200 cells/µL - Forest plot for W4SS

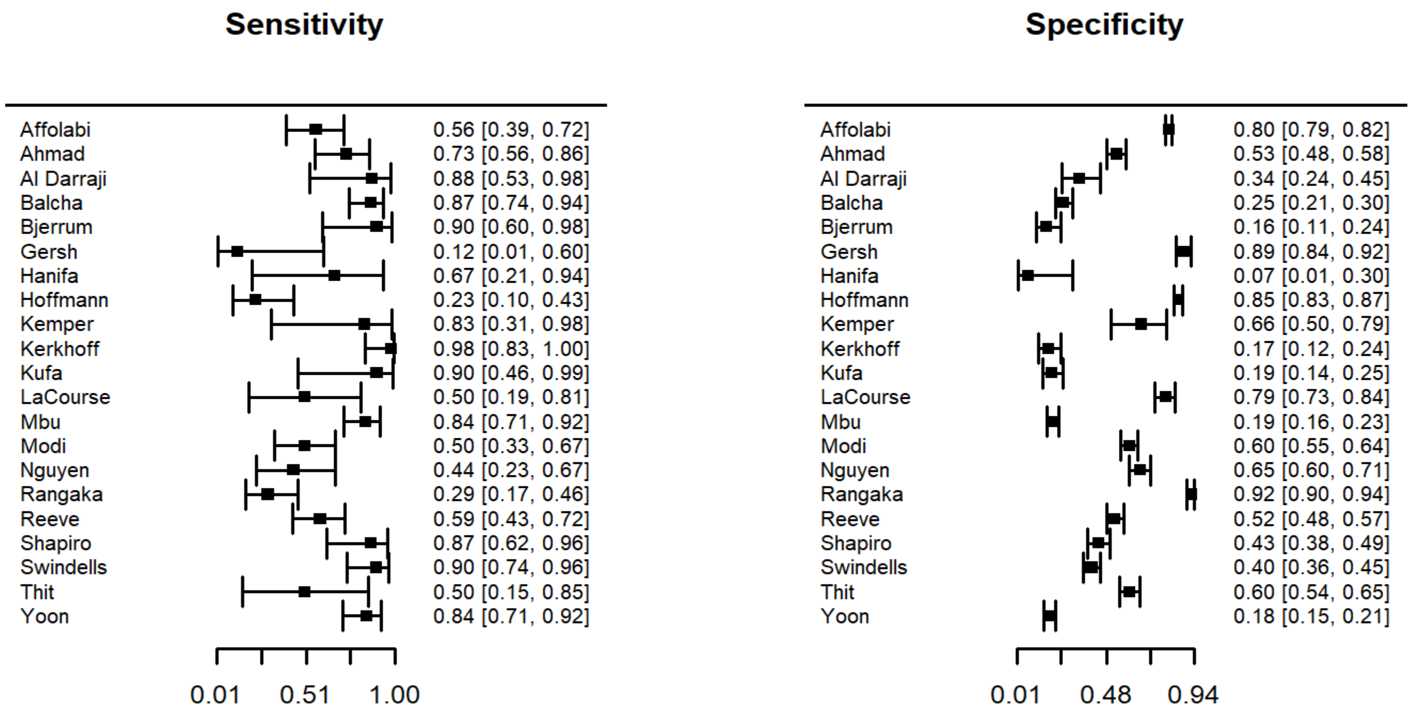

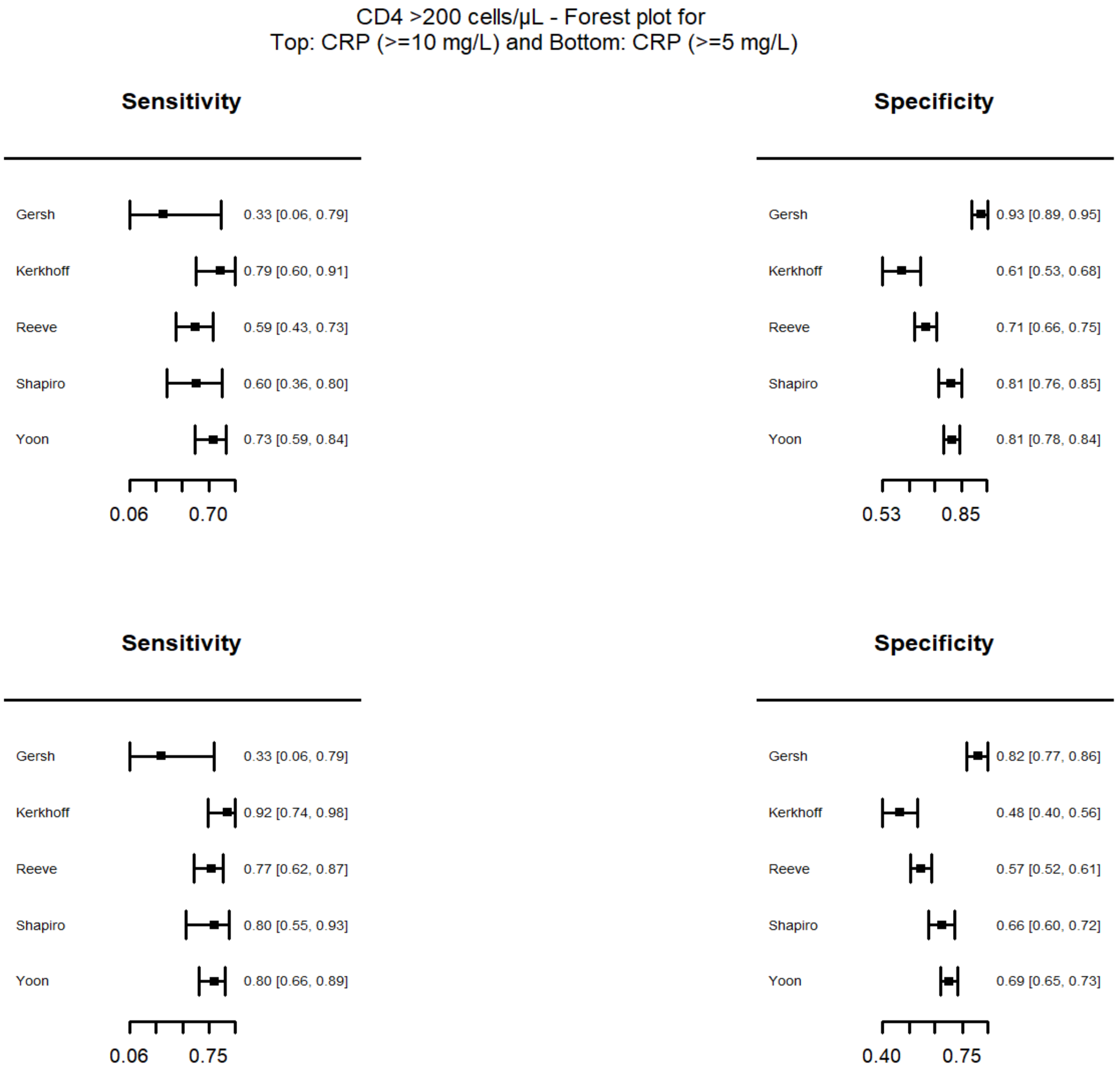
CD4 >200 cells/µL - Forest plot for
Top: CRP (>=10 mg/L) and Bottom: CRP (>=5 mg/L)
Sensitivity
Specificity
Gersh
0.33 [0.06, 0.79]
0.93 [0.89, 0.95]
Kerkhoff
0.79 [0.60, 0.91]
0.61 [0.53, 0.68]
Reeve
0.59 [0.43, 0.73]
0.71 [0.66, 0.75]
Shapiro
0.60 [0.36, 0.80]
0.81 [0.76, 0.85]
Yoon
0.73 [0.59, 0.84]
0.81 [0.78, 0.84]
0.06
0.70
0.53
0.85
Sensitivity
Specificity
Gersh
0.33 [0.06, 0.79]
0.82 [0.77, 0.86]
Kerkhoff
0.92 [0.74, 0.98]
0.48 [0.40, 0.56]
Reeve
0.77 [0.62, 0.87]
0.57 [0.52, 0.61]
Shapiro
0.80 [0.55, 0.93]
0.66 [0.60, 0.72]
Yoon
0.80 [0.66, 0.89]
0.69 [0.65, 0.73]
0.06
0.75
0.40
0.75

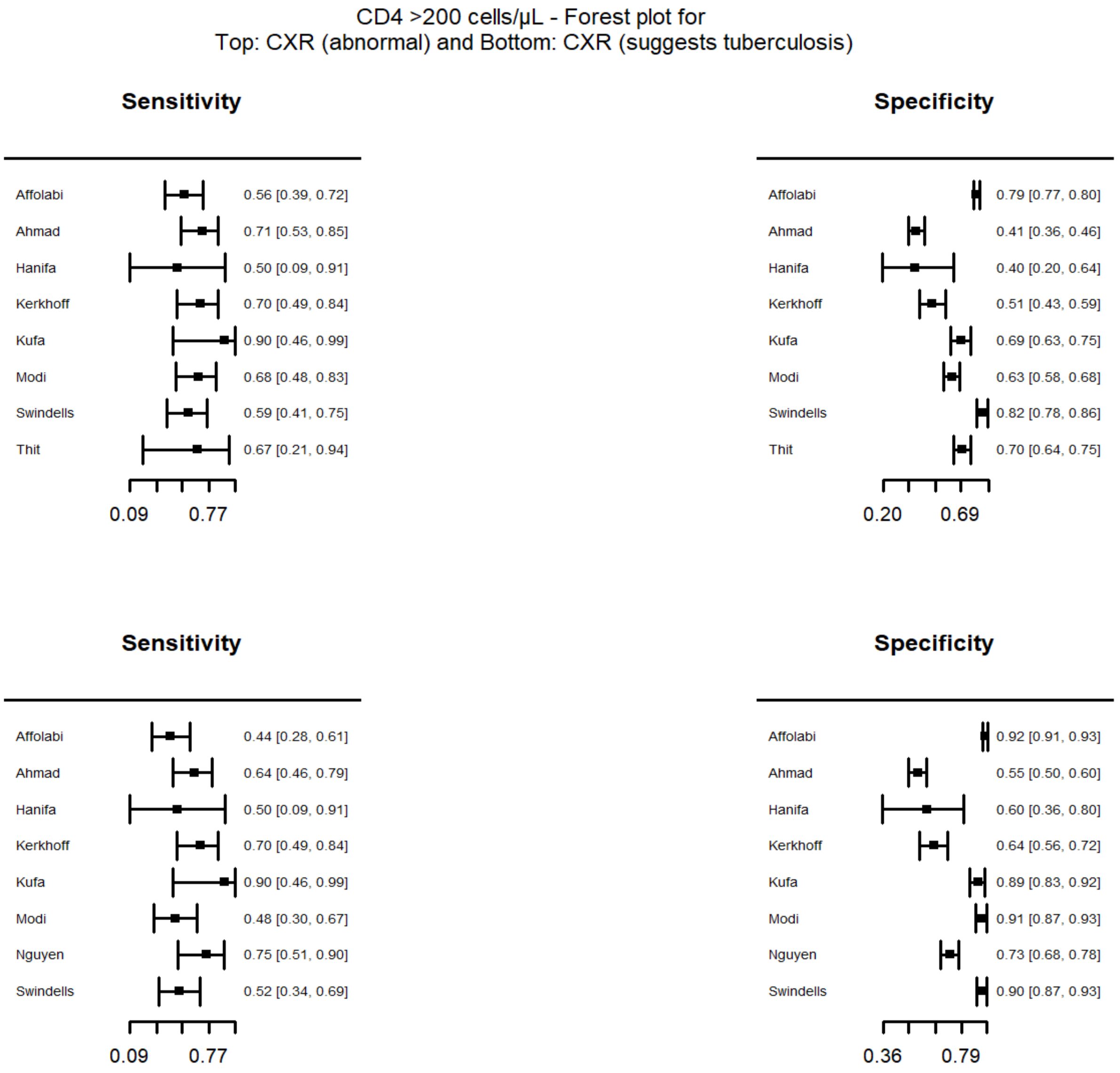

CD4 >200 cells/μL - Forest plot for
Top: CXR (abnormal) and Bottom: CXR (suggests tuberculosis)
Sensitivity
Affolabi 0.56 [0.39, 0.72]
Ahmad 0.71 [0.53, 0.85]
Hanifa 0.50 [0.09, 0.91]
Kerkhoff 0.70 [0.49, 0.84]
Kufa 0.90 [0.46, 0.99]
Modi 0.68 [0.48, 0.83]
Swindells 0.59 [0.41, 0.75]
Thit 0.67 [0.21, 0.94]
0.09 0.77
Specificity
Affolabi 0.79 [0.77, 0.80]
Ahmad 0.41 [0.36, 0.46]
Hanifa 0.40 [0.20, 0.64]
Kerkhoff 0.51 [0.43, 0.59]
Kufa 0.69 [0.63, 0.75]
Modi 0.63 [0.58, 0.68]
Swindells 0.82 [0.78, 0.86]
Thit 0.70 [0.64, 0.75]
0.20 0.69
Sensitivity
Affolabi 0.44 [0.28, 0.61]
Ahmad 0.64 [0.46, 0.79]
Hanifa 0.50 [0.09, 0.91]
Kerkhoff 0.70 [0.49, 0.84]
Kufa 0.90 [0.46, 0.99]
Modi 0.48 [0.30, 0.67]
Nguyen 0.75 [0.51, 0.90]
Swindells 0.52 [0.34, 0.69]
0.09 0.77
Specificity
Affolabi 0.92 [0.91, 0.93]
Ahmad 0.55 [0.50, 0.60]
Hanifa 0.60 [0.36, 0.80]
Kerkhoff 0.64 [0.56, 0.72]
Kufa 0.89 [0.83, 0.92]
Modi 0.91 [0.87, 0.93]
Nguyen 0.73 [0.68, 0.78]
Swindells 0.90 [0.87, 0.93]
0.36 0.79

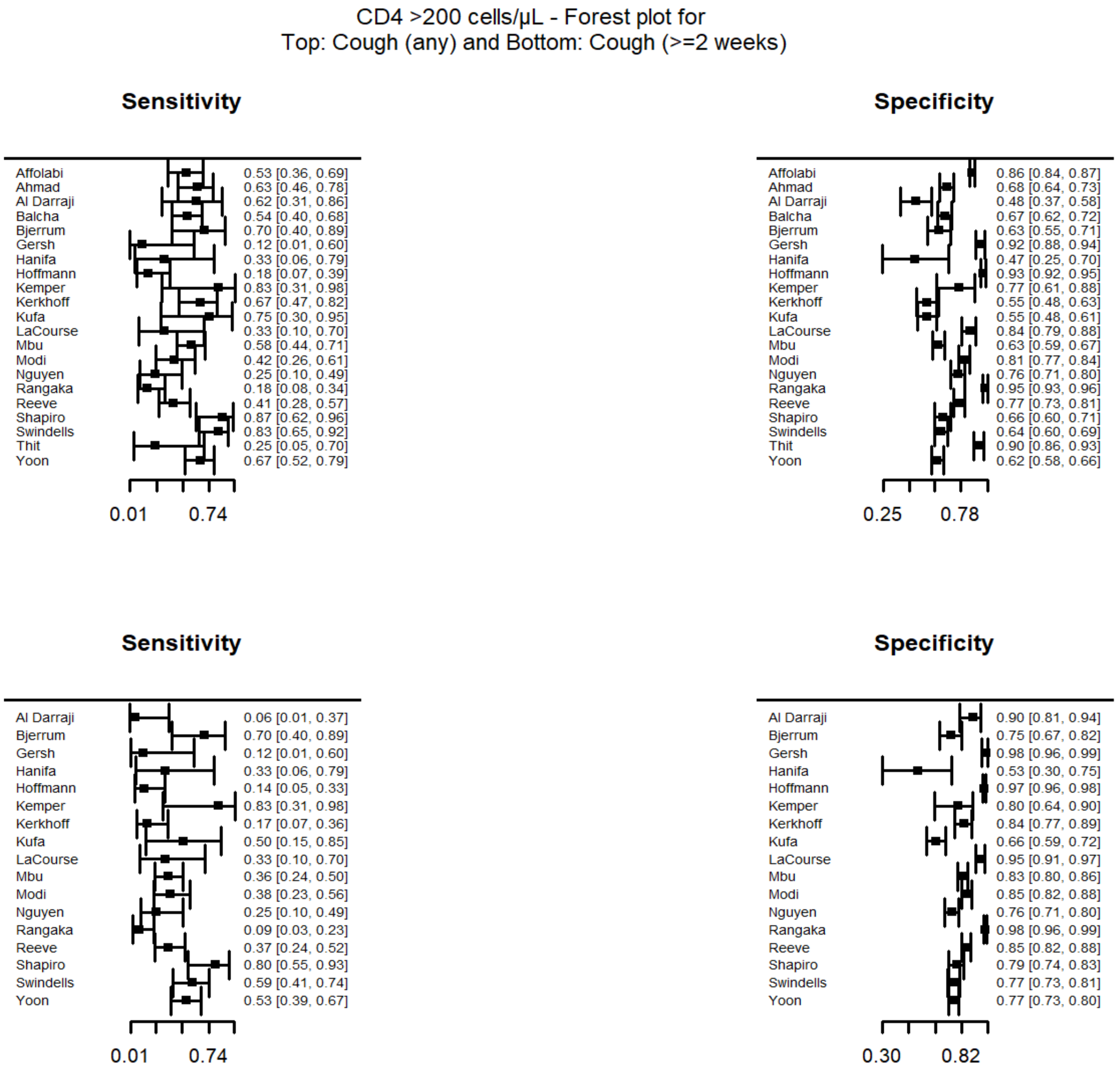
CD4 >200 cells/µL - Forest plot for
Top: Cough (any) and Bottom: Cough (>=2 weeks)
Sensitivity
Affolabi 0.53 [0.36, 0.69]
Ahmad 0.63 [0.46, 0.78]
Al Darraji 0.62 [0.31, 0.86]
Balcha 0.54 [0.40, 0.68]
Bjerrum 0.70 [0.40, 0.89]
Gersh 0.12 [0.01, 0.60]
Hanifa 0.33 [0.06, 0.79]
Hoffmann 0.18 [0.07, 0.39]
Kemper 0.83 [0.31, 0.98]
Kerkhoff 0.67 [0.47, 0.82]
Kufa 0.75 [0.30, 0.95]
LaCourse 0.33 [0.10, 0.70]
Mbu 0.58 [0.44, 0.71]
Modi 0.42 [0.26, 0.61]
Nguyen 0.25 [0.10, 0.49]
Rangaka 0.18 [0.08, 0.34]
Reeve 0.41 [0.28, 0.57]
Shapiro 0.87 [0.62, 0.96]
Swindells 0.83 [0.65, 0.92]
Thit 0.25 [0.05, 0.70]
Yoon 0.67 [0.52, 0.79]
0.01 0.74
Specificity
Affolabi 0.86 [0.84, 0.87]
Ahmad 0.68 [0.64, 0.73]
Al Darraji 0.48 [0.37, 0.58]
Balcha 0.67 [0.62, 0.72]
Bjerrum 0.63 [0.55, 0.71]
Gersh 0.92 [0.88, 0.94]
Hanifa 0.47 [0.25, 0.70]
Hoffmann 0.93 [0.92, 0.95]
Kemper 0.77 [0.61, 0.88]
Kerkhoff 0.55 [0.48, 0.63]
Kufa 0.55 [0.48, 0.61]
LaCourse 0.84 [0.79, 0.88]
Mbu 0.63 [0.59, 0.67]
Modi 0.81 [0.77, 0.84]
Nguyen 0.76 [0.71, 0.80]
Rangaka 0.95 [0.93, 0.96]
Reeve 0.77 [0.73, 0.81]
Shapiro 0.66 [0.60, 0.71]
Swindells 0.64 [0.60, 0.69]
Thit 0.90 [0.86, 0.93]
Yoon 0.62 [0.58, 0.66]
0.25 0.78
Sensitivity
Al Darraji 0.06 [0.01, 0.37]
Bjerrum 0.70 [0.40, 0.89]
Gersh 0.12 [0.01, 0.60]
Hanifa 0.33 [0.06, 0.79]
Hoffmann 0.14 [0.05, 0.33]
Kemper 0.83 [0.31, 0.98]
Kerkhoff 0.17 [0.07, 0.36]
Kufa 0.50 [0.15, 0.85]
LaCourse 0.33 [0.10, 0.70]
Mbu 0.36 [0.24, 0.50]
Modi 0.38 [0.23, 0.56]
Nguyen 0.25 [0.10, 0.49]
Rangaka 0.09 [0.03, 0.23]
Reeve 0.37 [0.24, 0.52]
Shapiro 0.80 [0.55, 0.93]
Swindells 0.59 [0.41, 0.74]
Yoon 0.53 [0.39, 0.67]
0.01 0.74
Specificity
Al Darraji 0.90 [0.81, 0.94]
Bjerrum 0.75 [0.67, 0.82]
Gersh 0.98 [0.96, 0.99]
Hanifa 0.53 [0.30, 0.75]
Hoffmann 0.97 [0.96, 0.98]
Kemper 0.80 [0.64, 0.90]
Kerkhoff 0.84 [0.77, 0.89]
Kufa 0.66 [0.59, 0.72]
LaCourse 0.95 [0.91, 0.97]
Mbu 0.83 [0.80, 0.86]
Modi 0.85 [0.82, 0.88]
Nguyen 0.76 [0.71, 0.80]
Rangaka 0.98 [0.96, 0.99]
Reeve 0.85 [0.82, 0.88]
Shapiro 0.79 [0.74, 0.83]
Swindells 0.77 [0.73, 0.81]
Yoon 0.77 [0.73, 0.80]
0.30 0.82

CD4 >200 cells/μL - Forest plot for
Top: Hb (<10 g/dL) and Bottom: Hb (<8 g/dL)

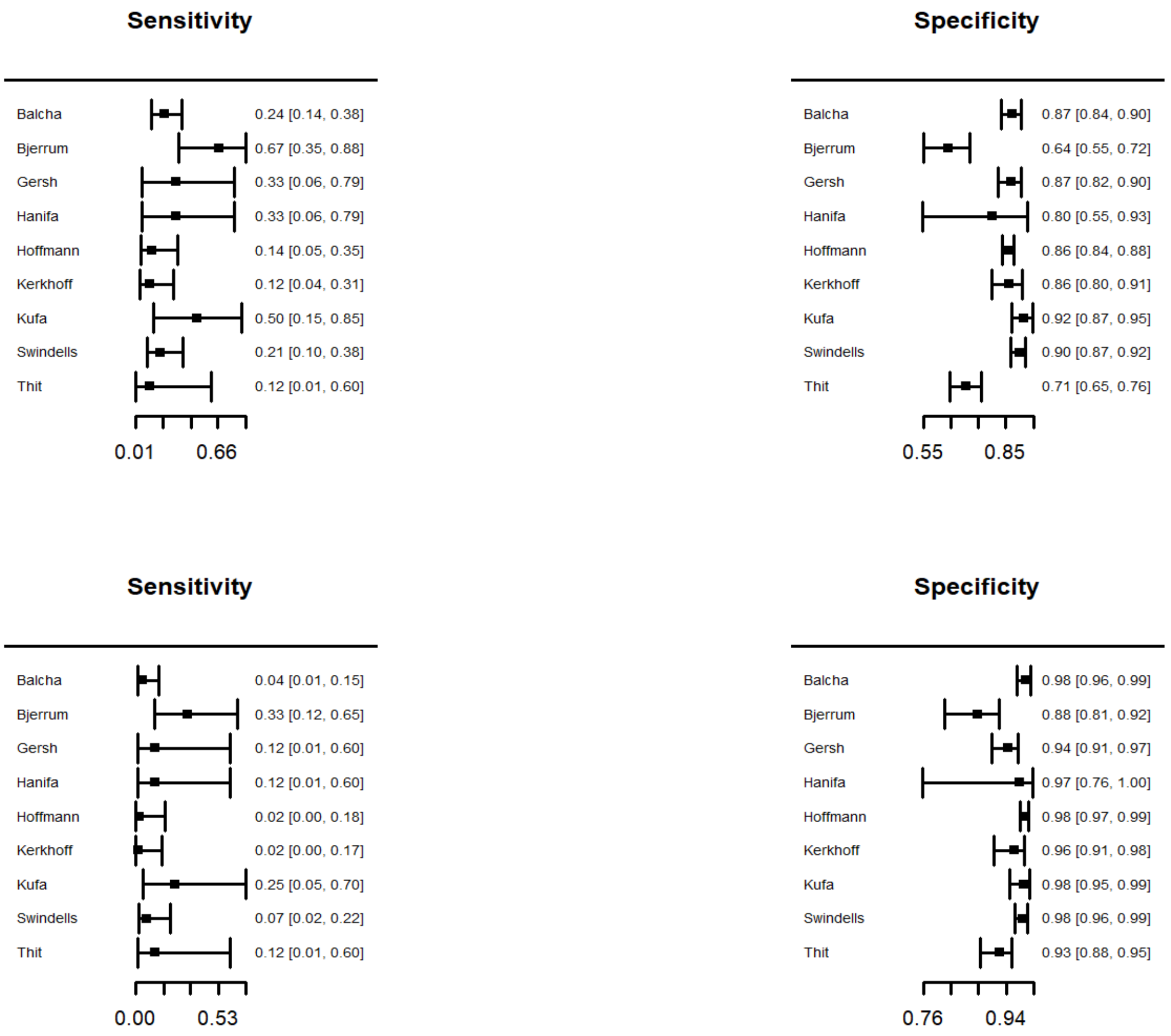

CD4 >200 cells/μL - Forest plot for
BMI (<18.5 kg/m^2)

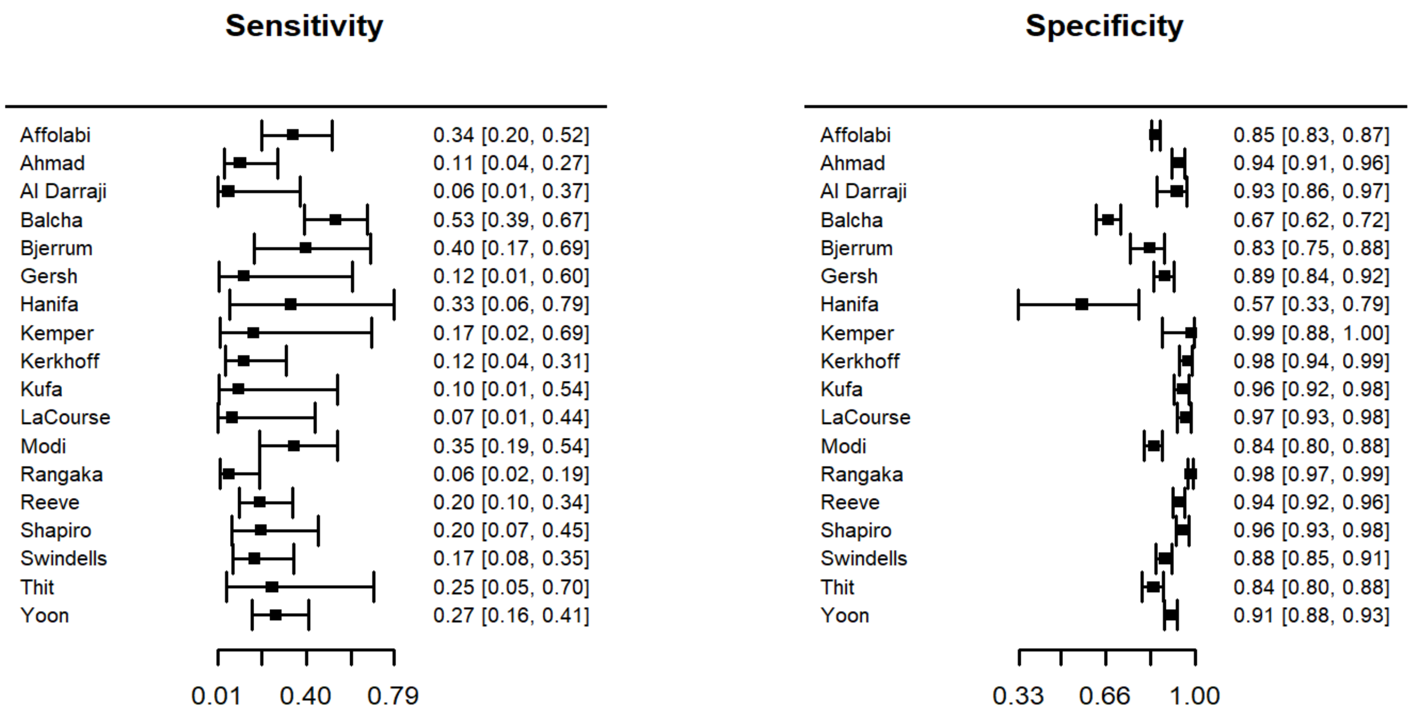

CD4 >200 cells/μL - Forest plot for Lymphadenopathy

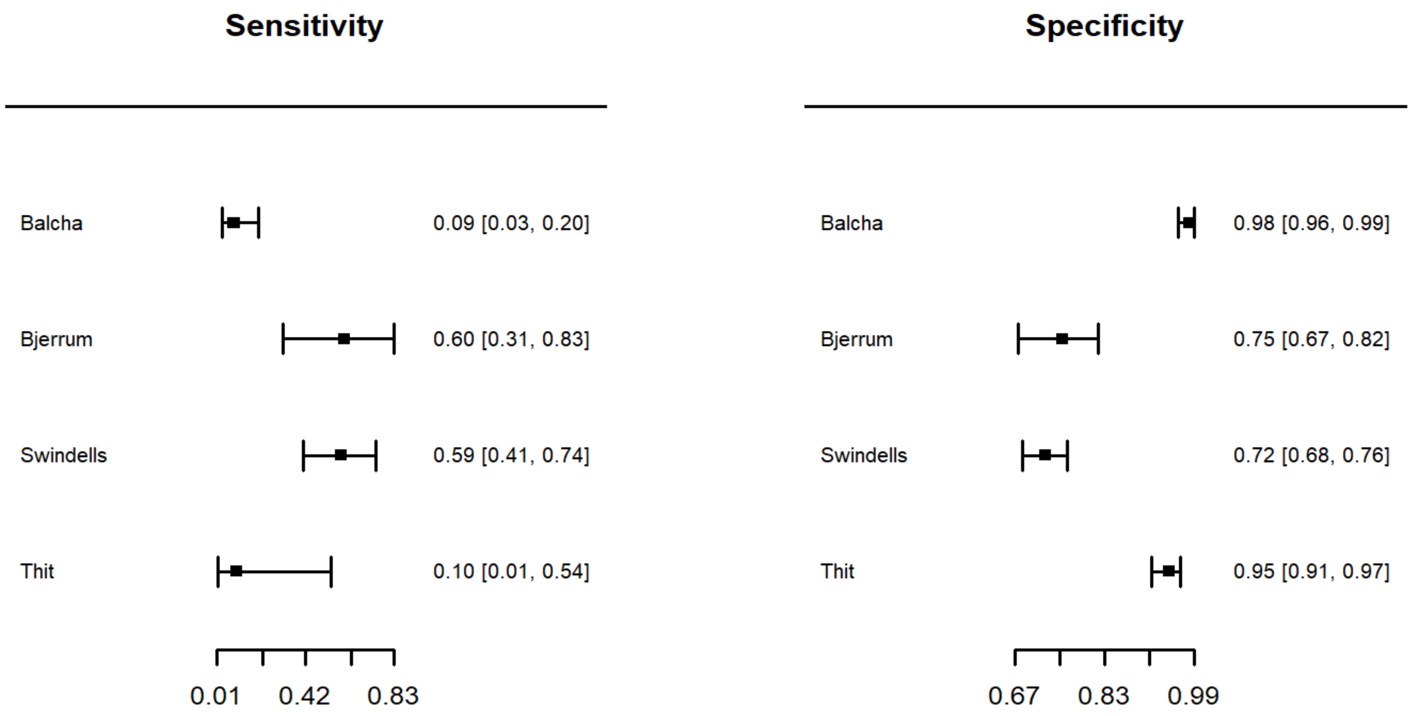

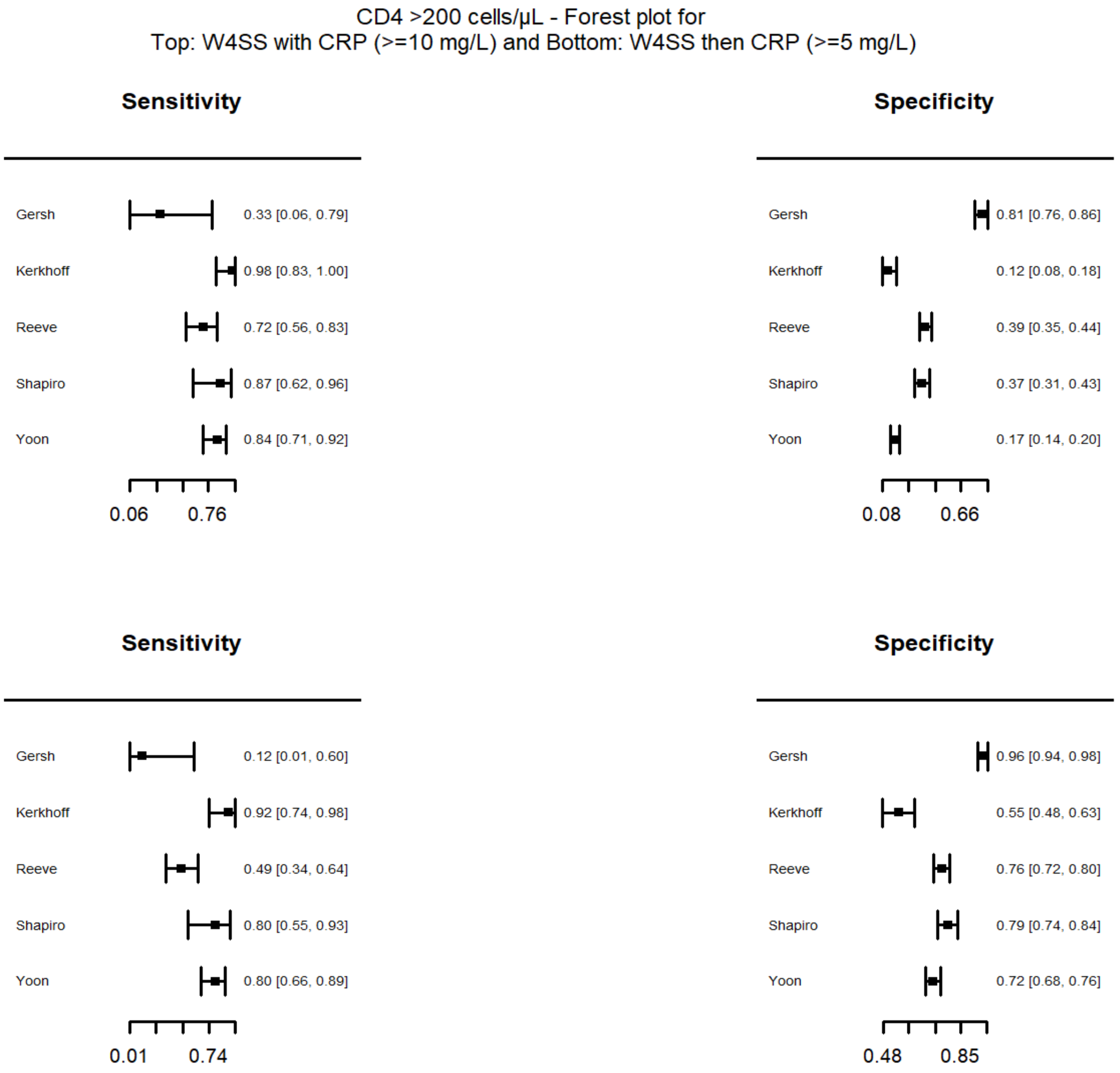
CD4 >200 cells/µL - Forest plot for
Top: W4SS with CRP (>=10 mg/L) and Bottom: W4SS then CRP (>=5 mg/L)
Sensitivity
Gersh 0.33 [0.06, 0.79]
Kerkhoff 0.98 [0.83, 1.00]
Reeve 0.72 [0.56, 0.83]
Shapiro 0.87 [0.62, 0.96]
Yoon 0.84 [0.71, 0.92]
0.06 0.76
Specificity
Gersh 0.81 [0.76, 0.86]
Kerkhoff 0.12 [0.08, 0.18]
Reeve 0.39 [0.35, 0.44]
Shapiro 0.37 [0.31, 0.43]
Yoon 0.17 [0.14, 0.20]
0.08 0.66
Sensitivity
Gersh 0.12 [0.01, 0.60]
Kerkhoff 0.92 [0.74, 0.98]
Reeve 0.49 [0.34, 0.64]
Shapiro 0.80 [0.55, 0.93]
Yoon 0.80 [0.66, 0.89]
0.01 0.74
Specificity
Gersh 0.96 [0.94, 0.98]
Kerkhoff 0.55 [0.48, 0.63]
Reeve 0.76 [0.72, 0.80]
Shapiro 0.79 [0.74, 0.84]
Yoon 0.72 [0.68, 0.76]
0.48 0.85

CD4 >200 cells/μL - Forest plot for
W4SS with CXR (abnormal)

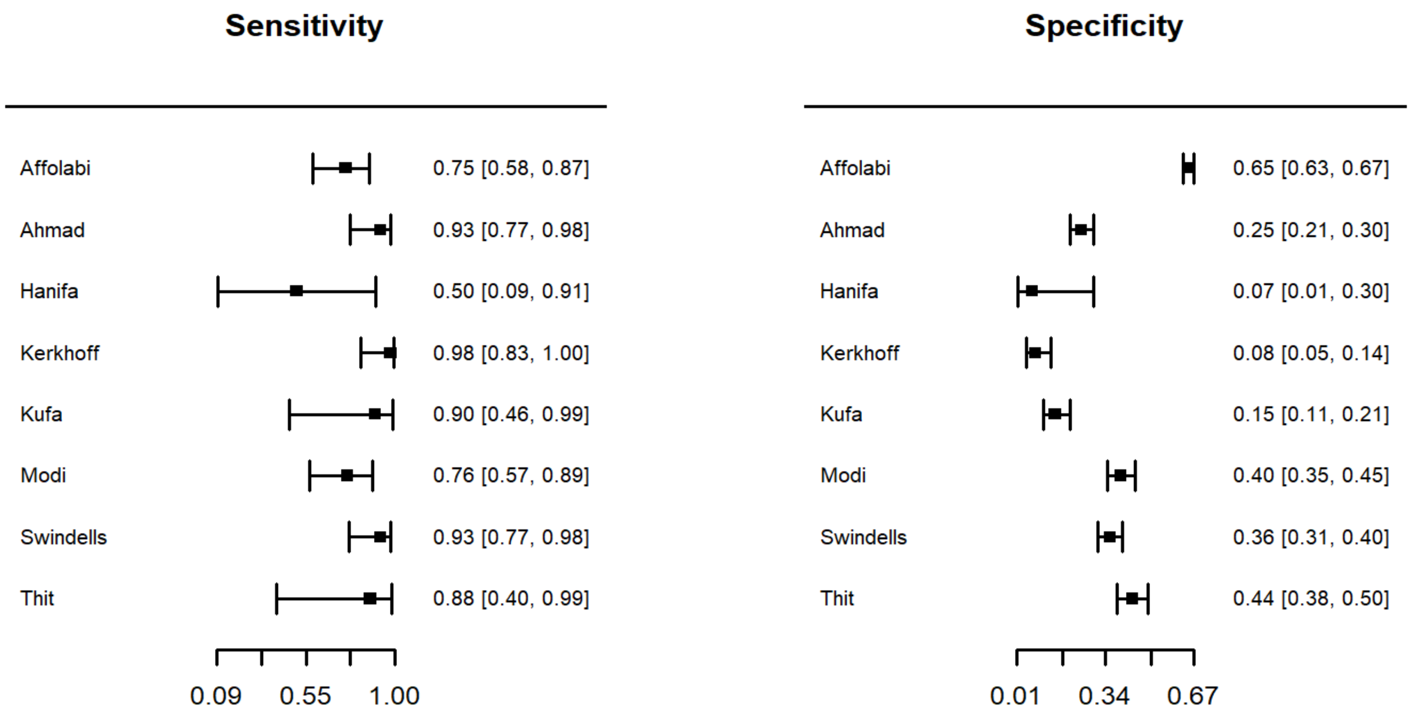

CD4 >200 cells/μL - Forest plot for
Top: W4SS then Xpert and Bottom: Xpert alone

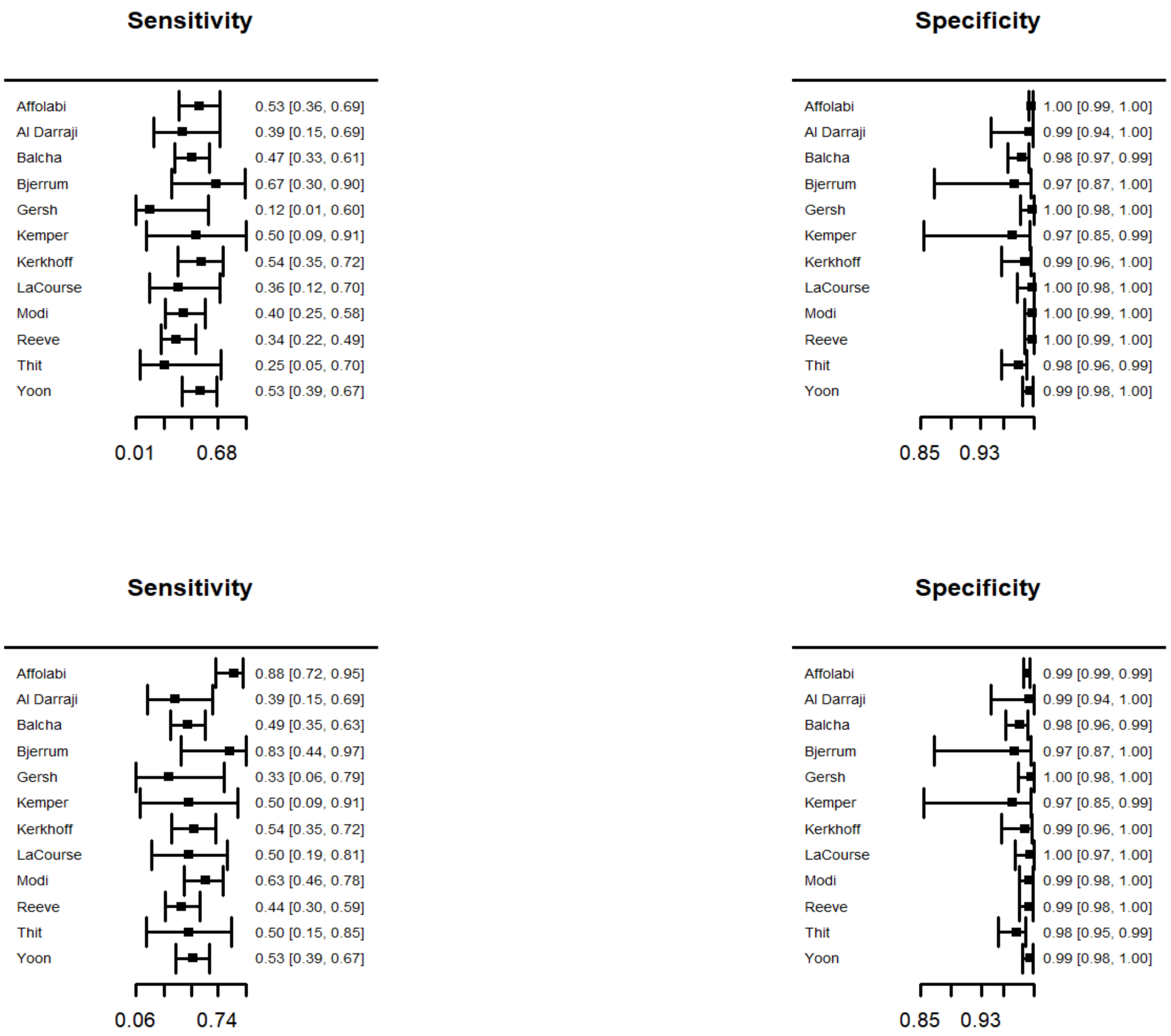

Figure S3F - Forest plots of sensitivity and specificity estimates in pregnant participants

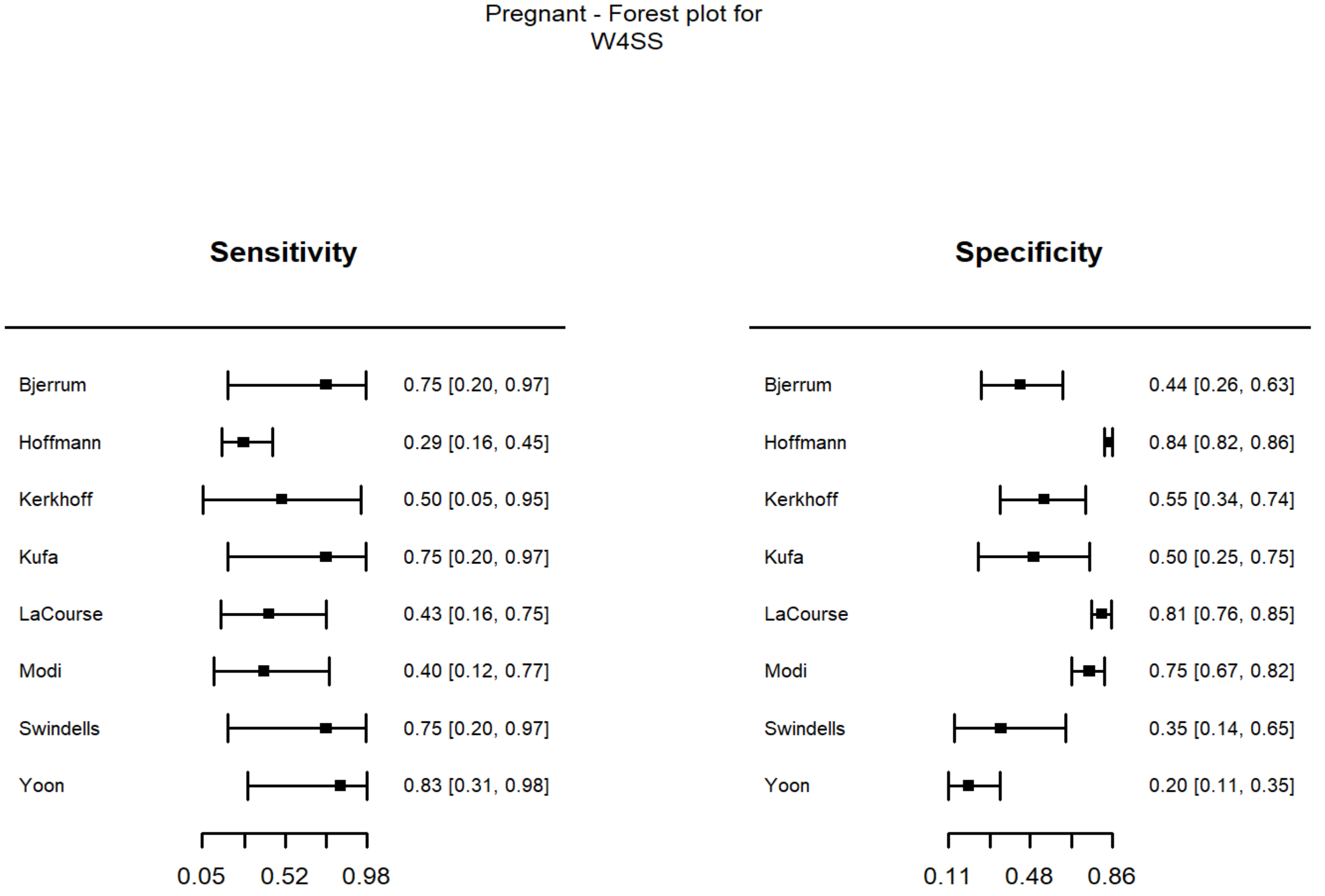

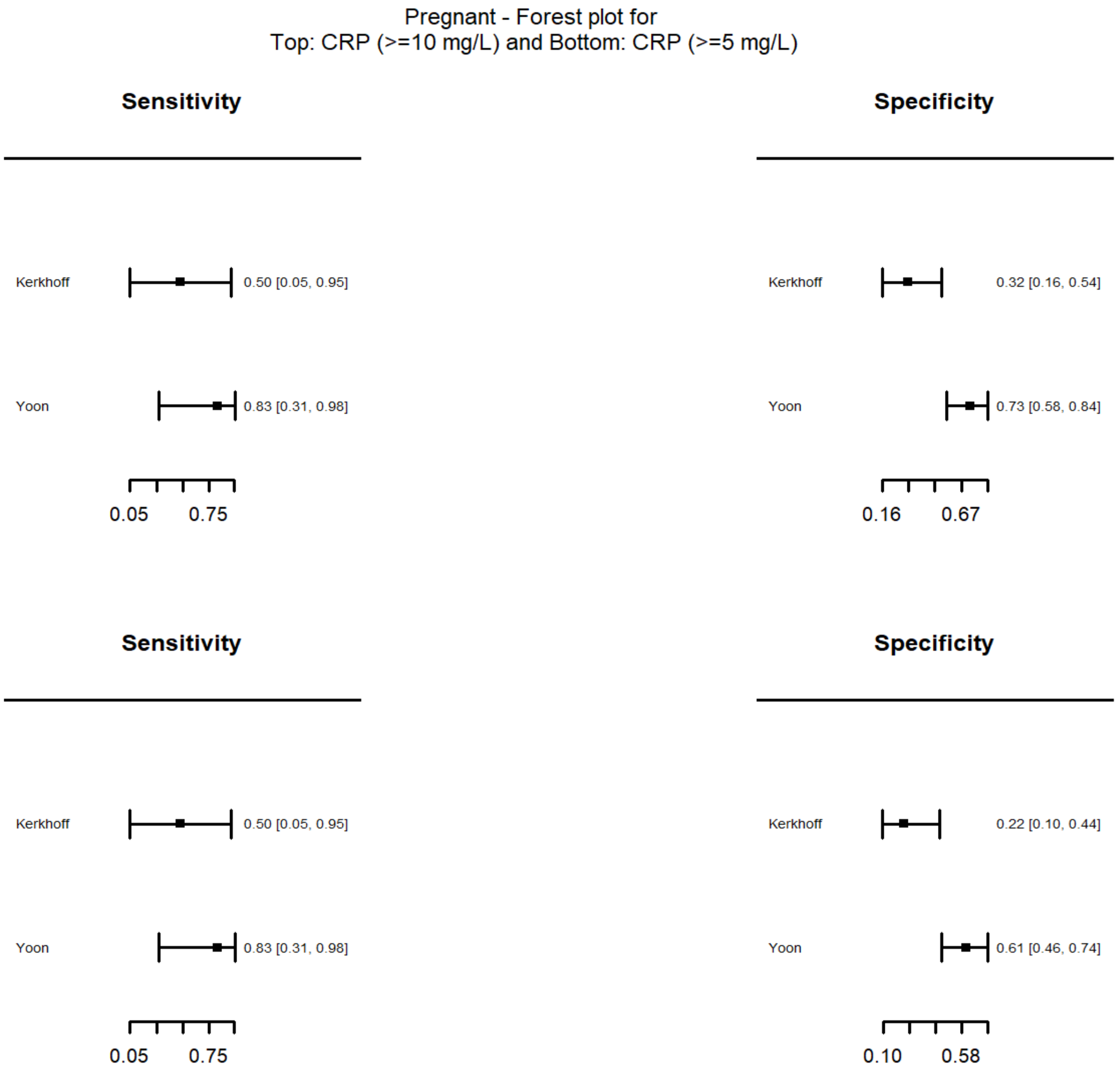
Pregnant - Forest plot for
Top: CRP (>=10 mg/L) and Bottom: CRP (>=5 mg/L)
Sensitivity
Kerkhoff 0.50 [0.05, 0.95]
Yoon 0.83 [0.31, 0.98]
0.05 0.75
Specificity
Kerkhoff 0.32 [0.16, 0.54]
Yoon 0.73 [0.58, 0.84]
0.16 0.67
Sensitivity
Kerkhoff 0.50 [0.05, 0.95]
Yoon 0.83 [0.31, 0.98]
0.05 0.75
Specificity
Kerkhoff 0.22 [0.10, 0.44]
Yoon 0.61 [0.46, 0.74]
0.10 0.58

Pregnant - Forest plot for
Top: CXR (abnormal) and Bottom: CXR (suggests tuberculosis)

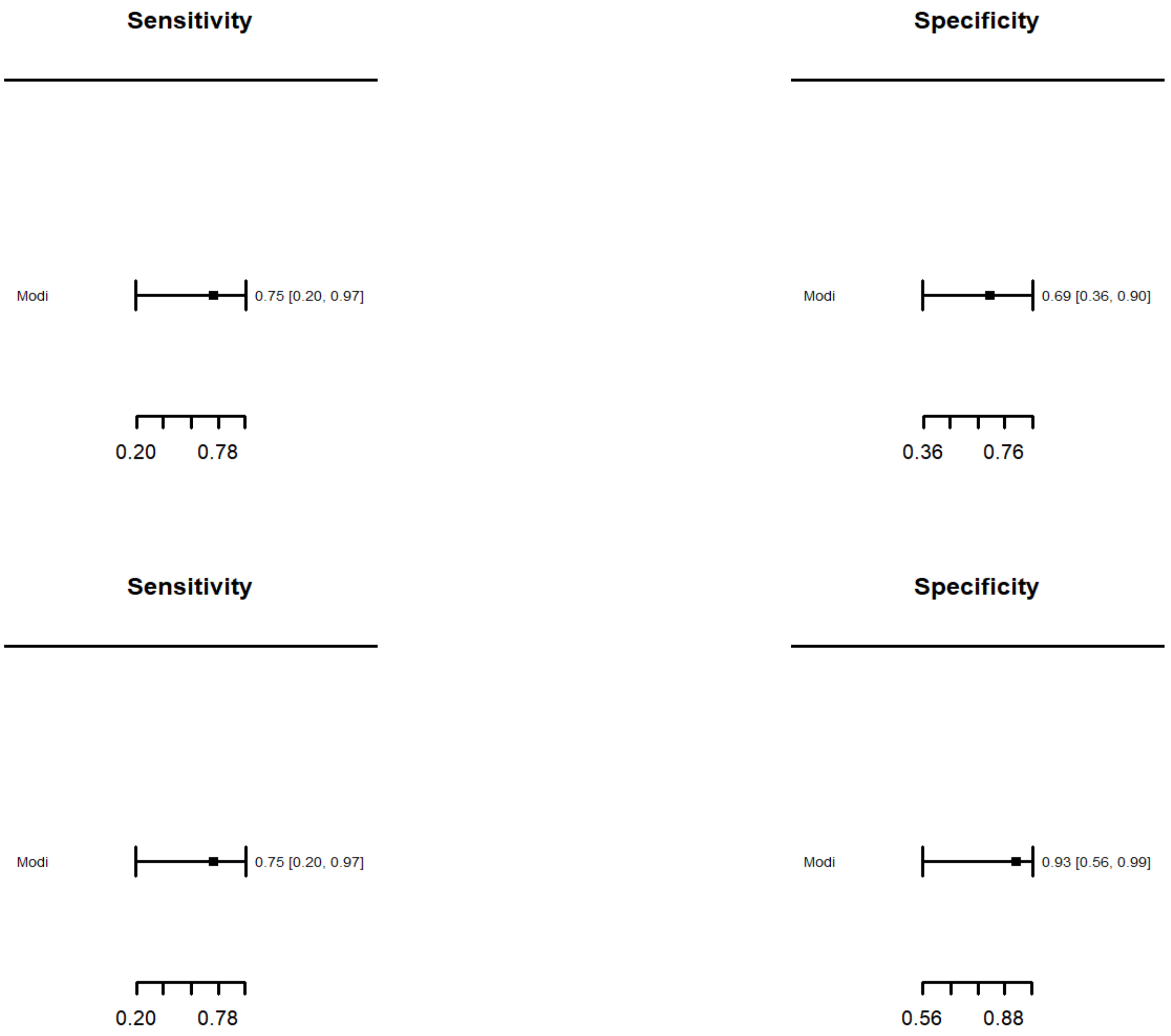

Pregnant - Forest plot for
Top: Cough (any) and Bottom: Cough (>=2 weeks)

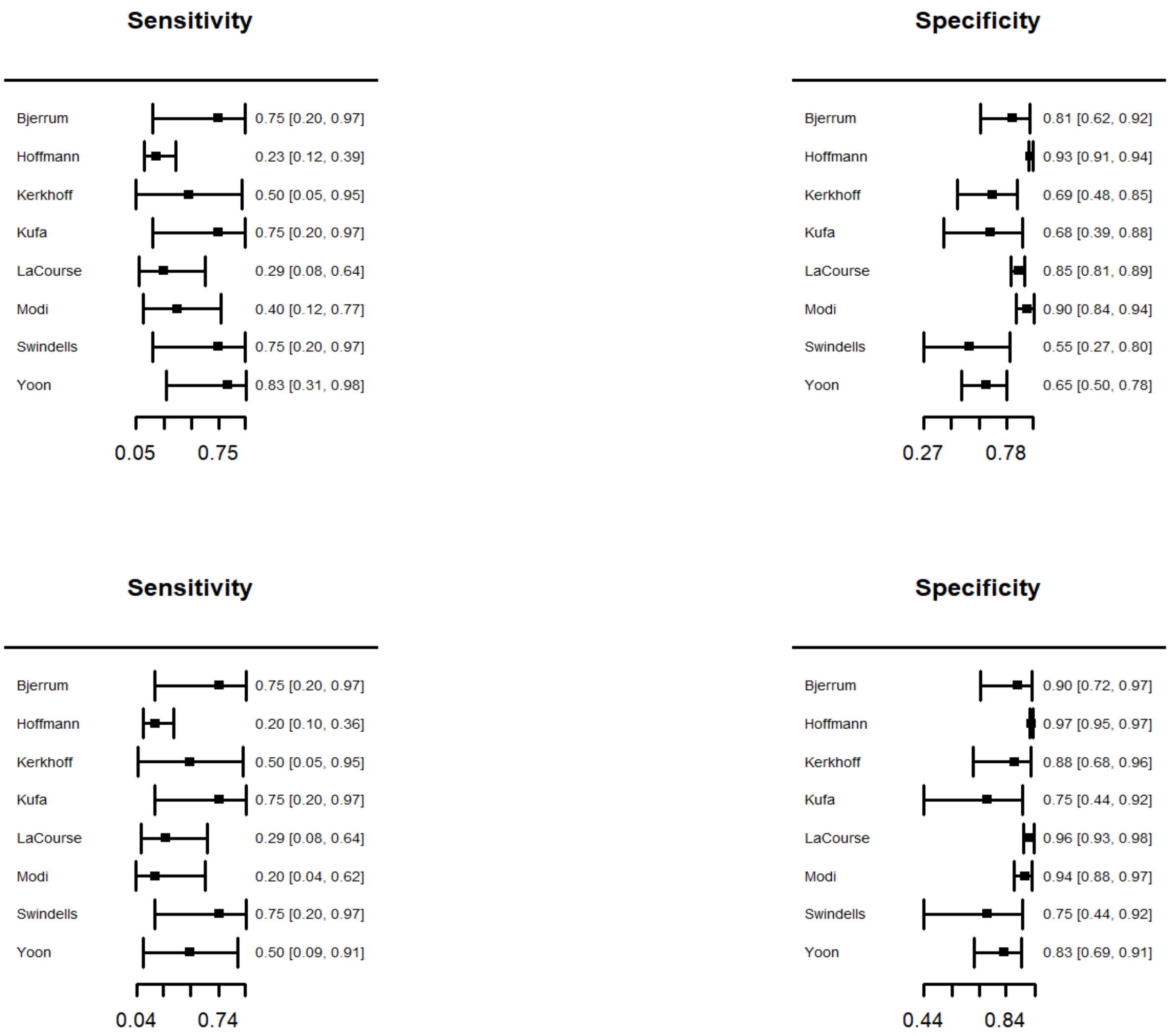

Pregnant - Forest plot for
Top: Hb (<10 g/dL) and Bottom: Hb (<8 g/dL)

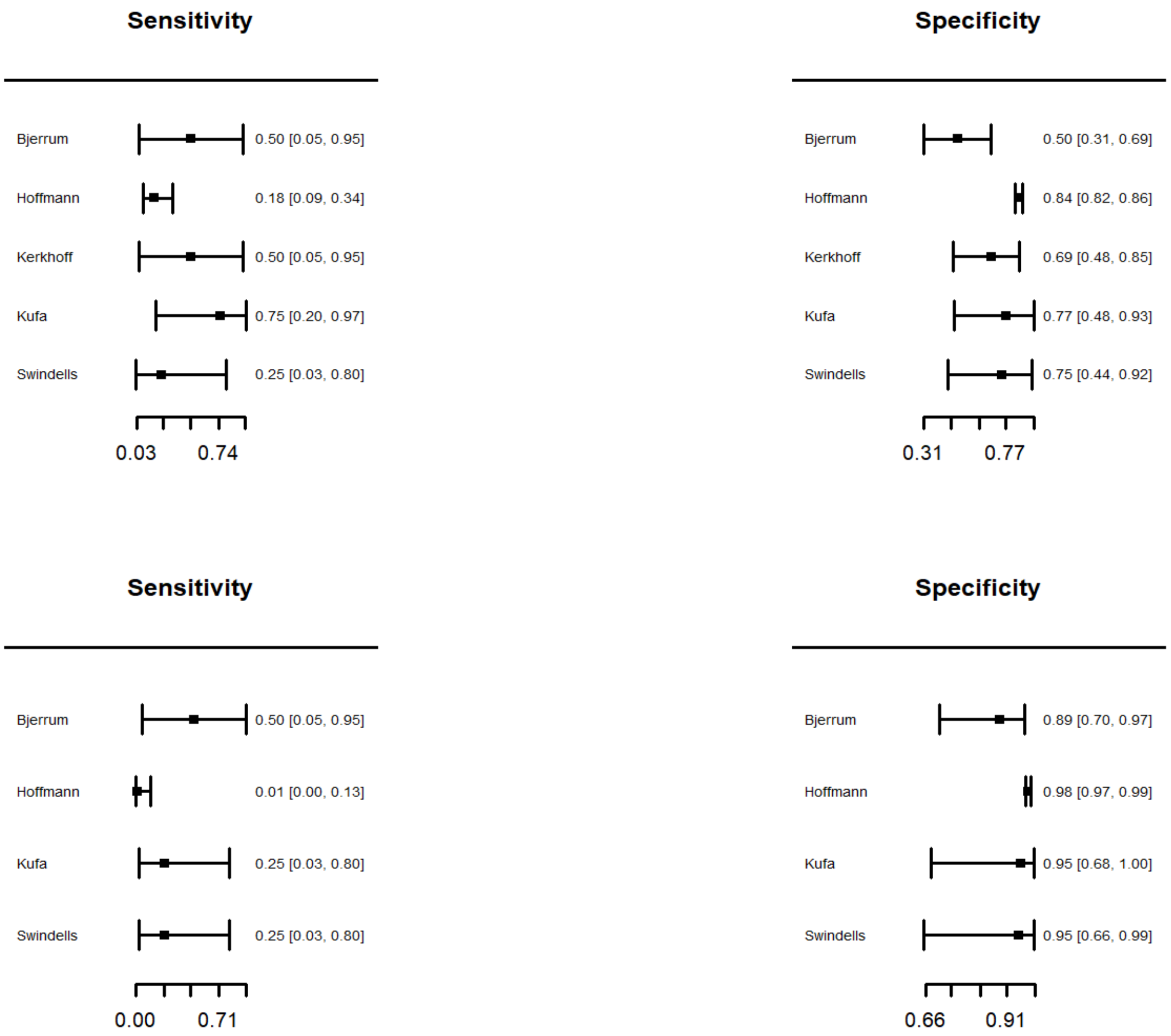

Pregnant - Forest plot for
BMI (<18.5 kg/m^2)

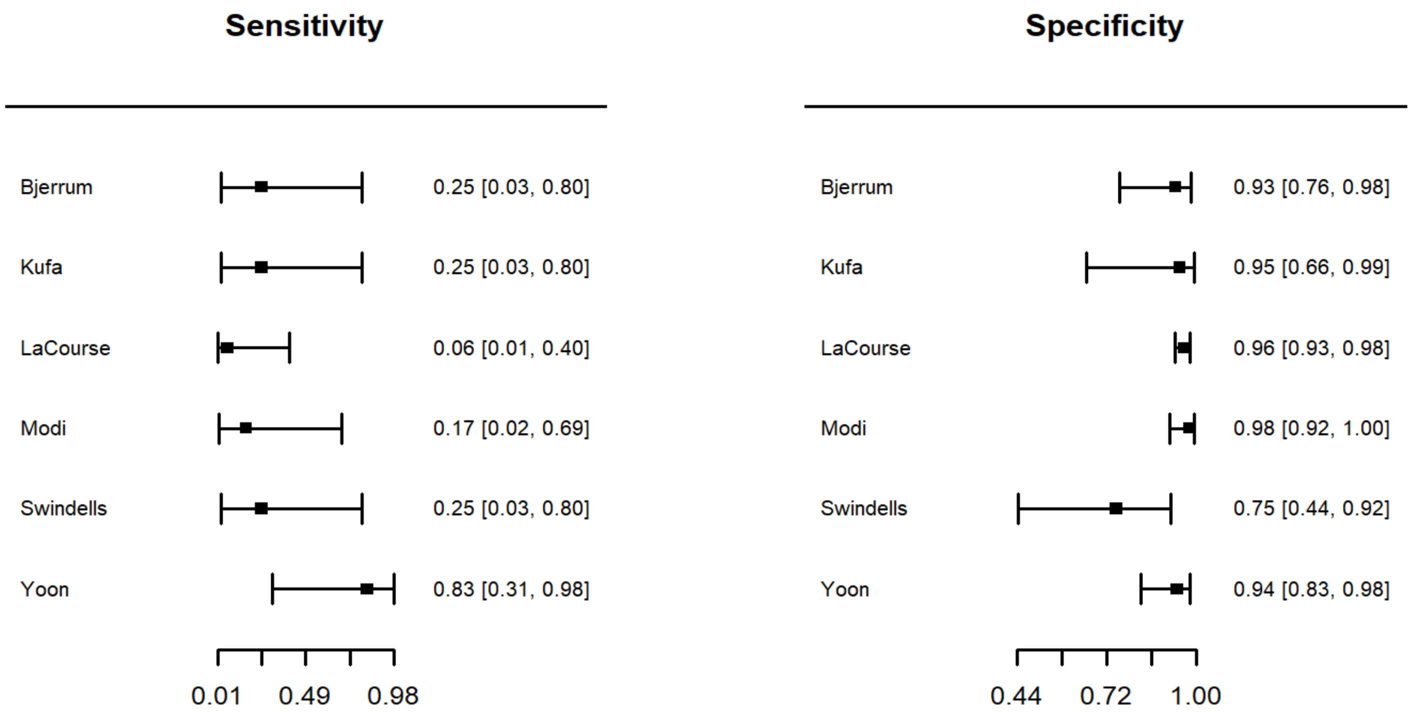

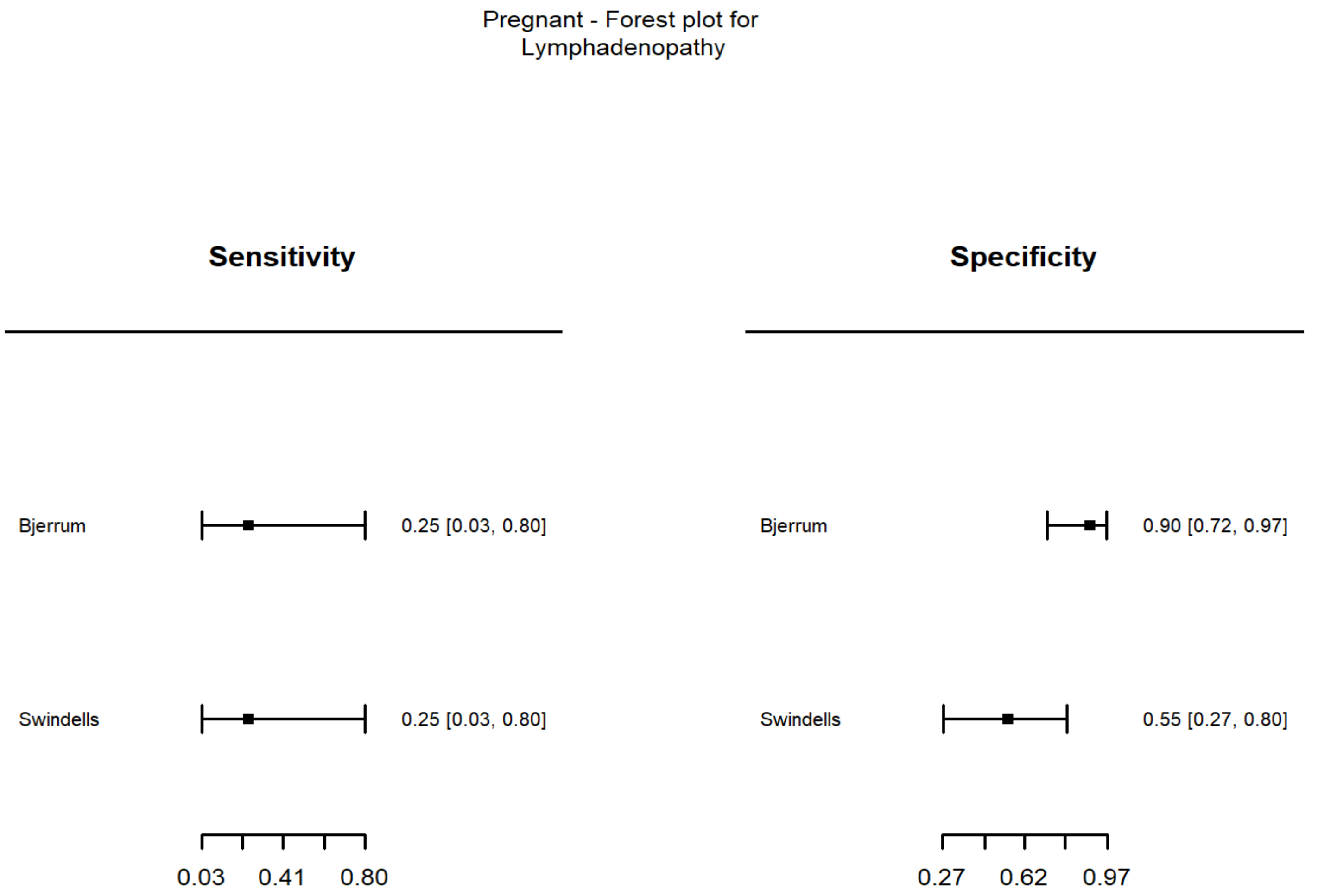
Pregnant - Forest plot for
Lymphadenopathy
Sensitivity
Specificity
Bjerrum
0.25 [0.03, 0.80]
Bjerrum
0.90 [0.72, 0.97]
Swindells
0.25 [0.03, 0.80]
Swindells
0.55 [0.27, 0.80]
0.03
0.41
0.80
0.27
0.62
0.97

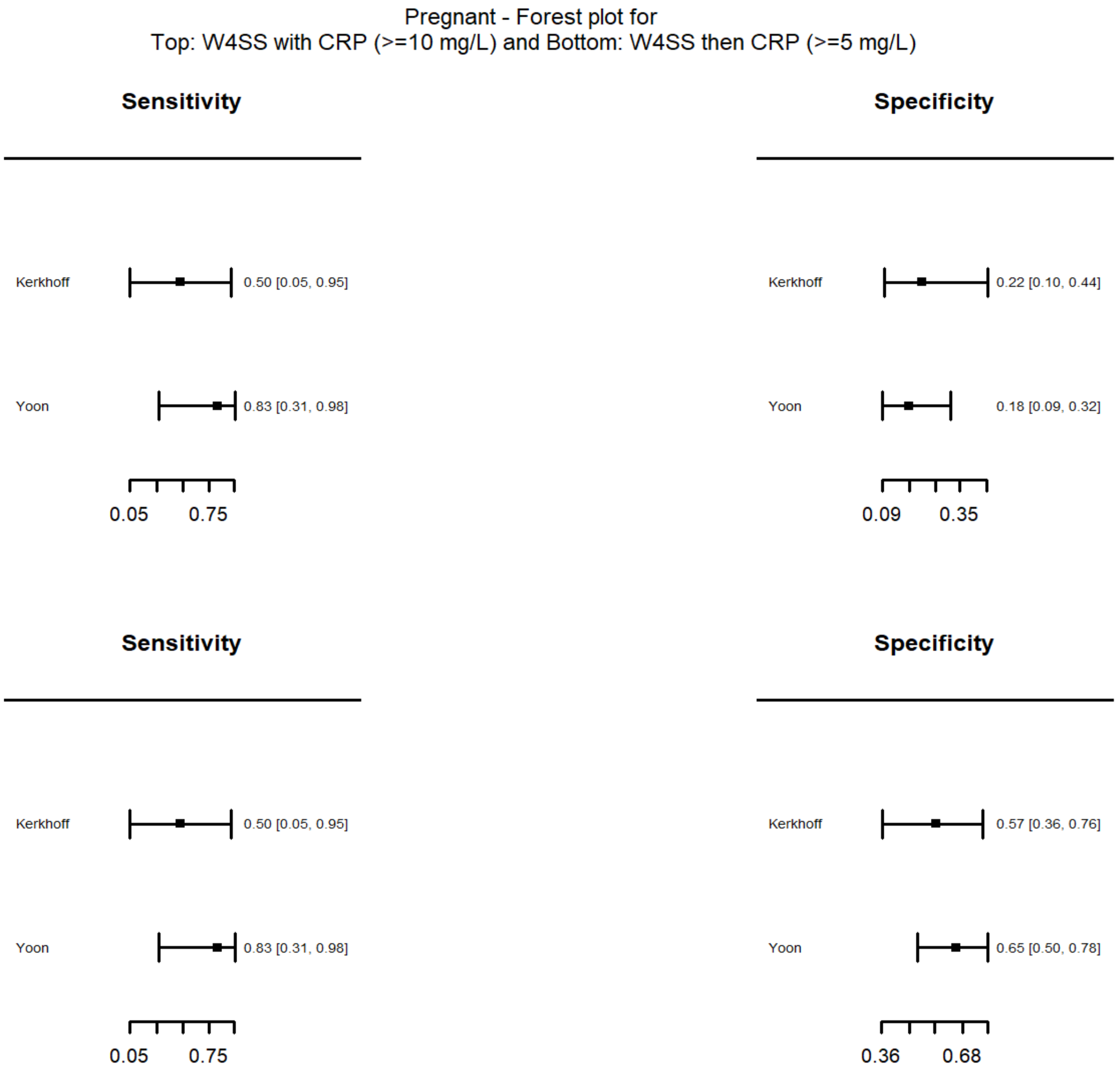
Pregnant - Forest plot for
Top: W4SS with CRP (>=10 mg/L) and Bottom: W4SS then CRP (>=5 mg/L)
Sensitivity
Kerkhoff
0.50 [0.05, 0.95]
Yoon
0.83 [0.31, 0.98]
0.05
0.75
Specificity
Kerkhoff
0.22 [0.10, 0.44]
Yoon
0.18 [0.09, 0.32]
0.09
0.35
Sensitivity
Kerkhoff
0.50 [0.05, 0.95]
Yoon
0.83 [0.31, 0.98]
0.05
0.75
Specificity
Kerkhoff
0.57 [0.36, 0.76]
Yoon
0.65 [0.50, 0.78]
0.36
0.68

Pregnant - Forest plot for
W4SS with CXR (abnormal)

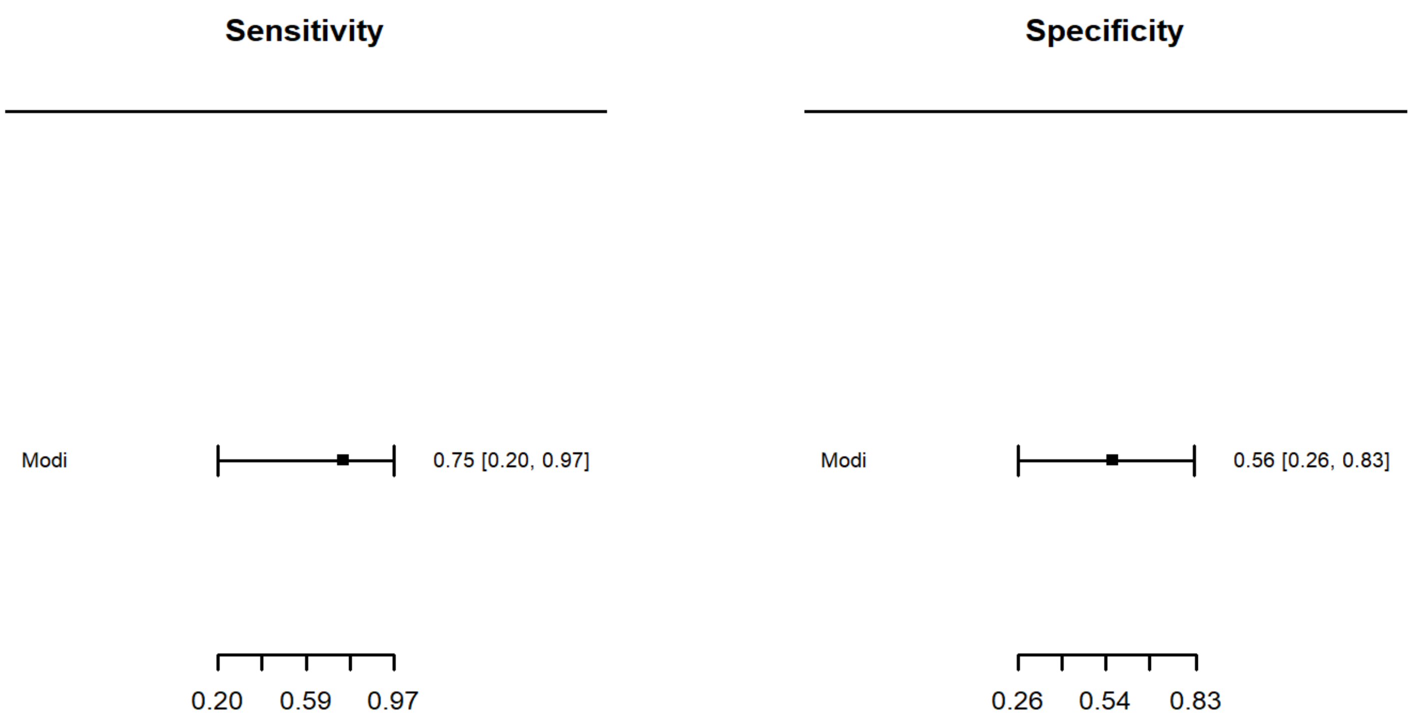

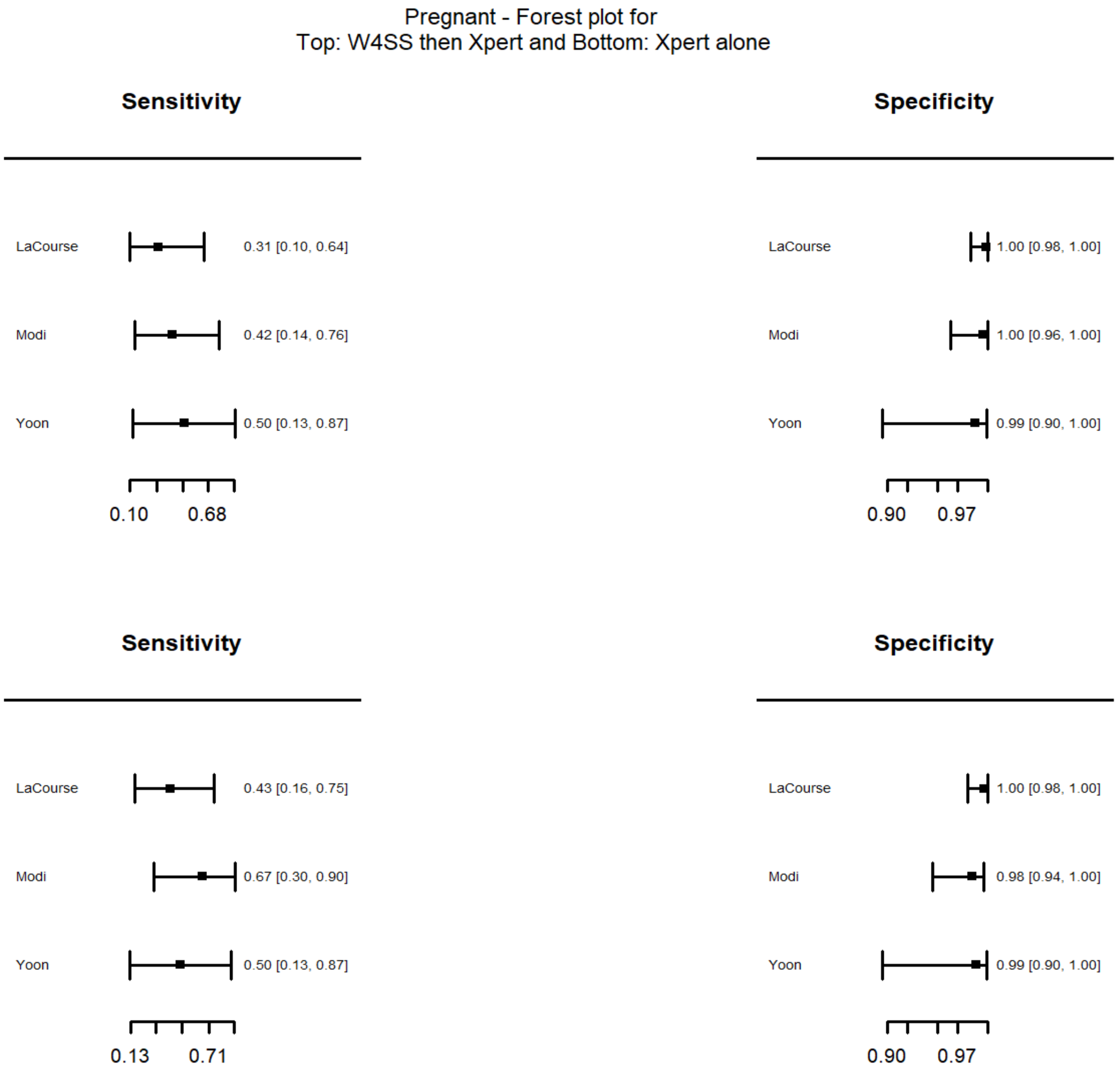
Pregnant - Forest plot for
Top: W4SS then Xpert and Bottom: Xpert alone
Sensitivity
LaCourse
0.31 [0.10, 0.64]
Modi
0.42 [0.14, 0.76]
Yoon
0.50 [0.13, 0.87]
0.10
0.68
Specificity
LaCourse
1.00 [0.98, 1.00]
Modi
1.00 [0.96, 1.00]
Yoon
0.99 [0.90, 1.00]
0.90
0.97
Sensitivity
LaCourse
0.43 [0.16, 0.75]
Modi
0.67 [0.30, 0.90]
Yoon
0.50 [0.13, 0.87]
0.13
0.71
Specificity
LaCourse
1.00 [0.98, 1.00]
Modi
0.98 [0.94, 1.00]
Yoon
0.99 [0.90, 1.00]
0.90
0.97

Figure S4 - Summary receiver operating characteristics curves in all participants and subgroups (for tests/strategies with >=2 studies available)

Figure S4A - Summary receiver operating characteristics curves comparing each test and WHO four-symptom screen for the detection of tuberculosis in all participants

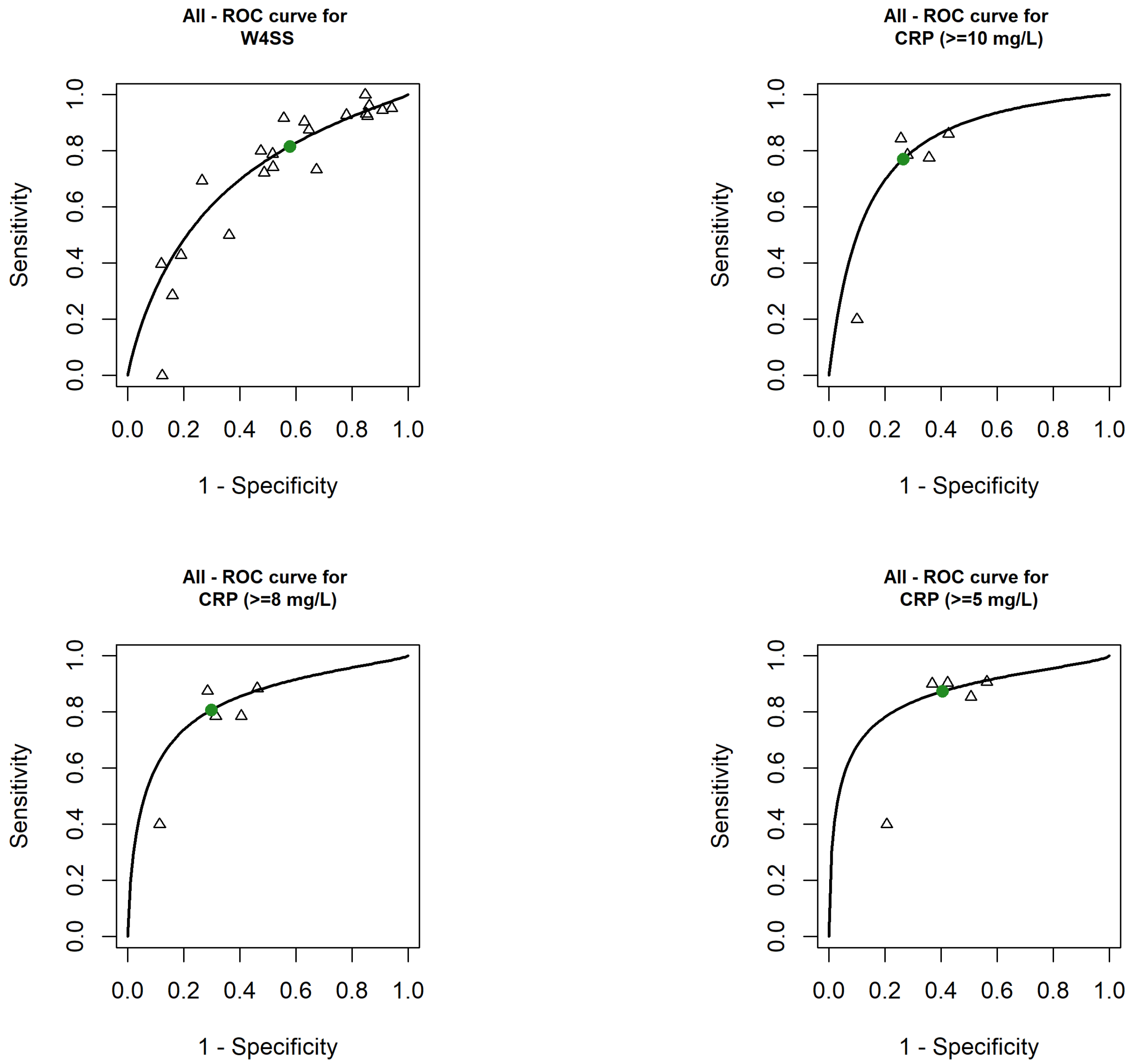

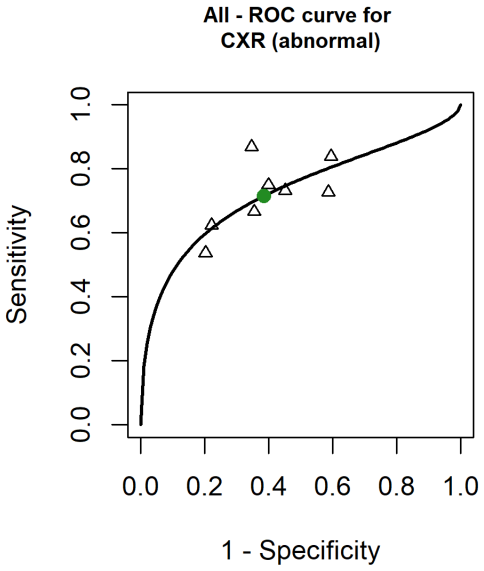
All - ROC curve for
CXR (abnormal)
Sensitivity
1 - Specificity
0.0 0.2 0.4 0.6 0.8 1.0

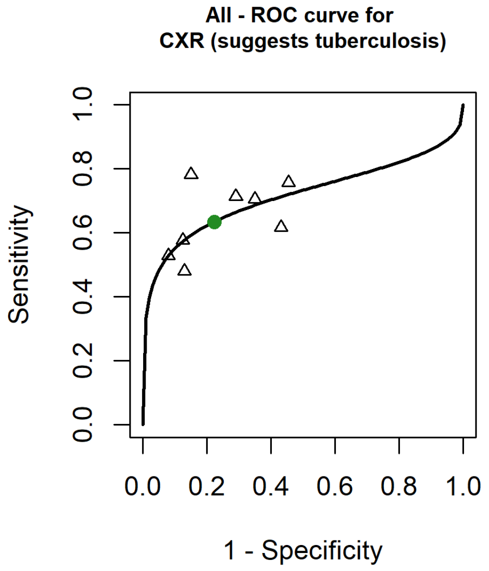
All - ROC curve for
CXR (suggests tuberculosis)
Sensitivity
1 - Specificity
0.0 0.2 0.4 0.6 0.8 1.0

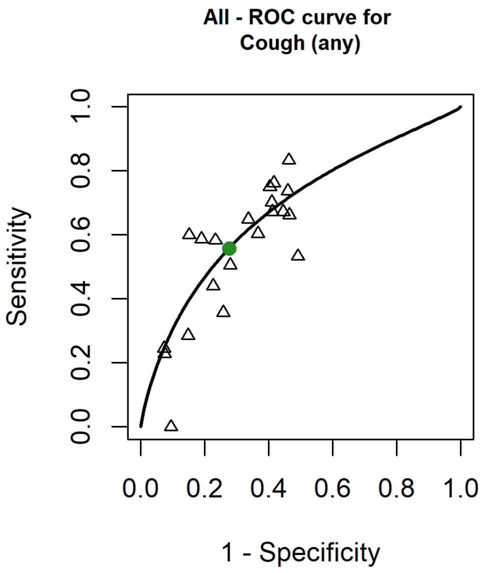
All - ROC curve for
Cough (any)
Sensitivity
1 - Specificity
0.0 0.2 0.4 0.6 0.8 1.0

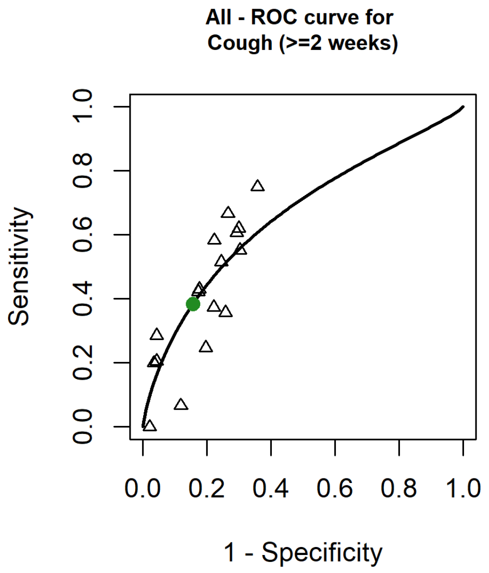
All - ROC curve for
Cough (>=2 weeks)
Sensitivity
1 - Specificity
0.0 0.2 0.4 0.6 0.8 1.0

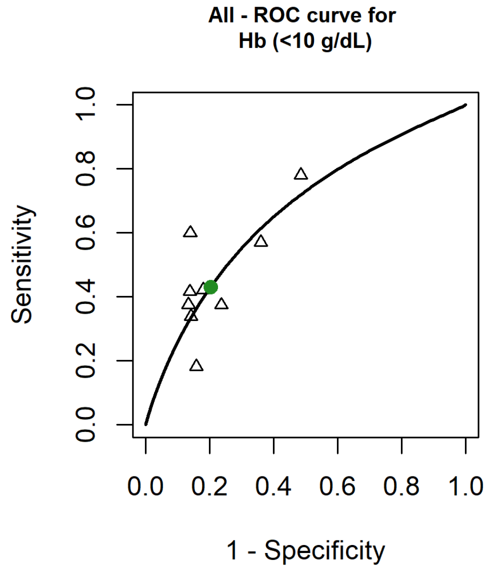
All - ROC curve for
Hb (<10 g/dL)
Sensitivity
0.0 0.2 0.4 0.6 0.8 1.0
1 - Specificity
0.0 0.2 0.4 0.6 0.8 1.0

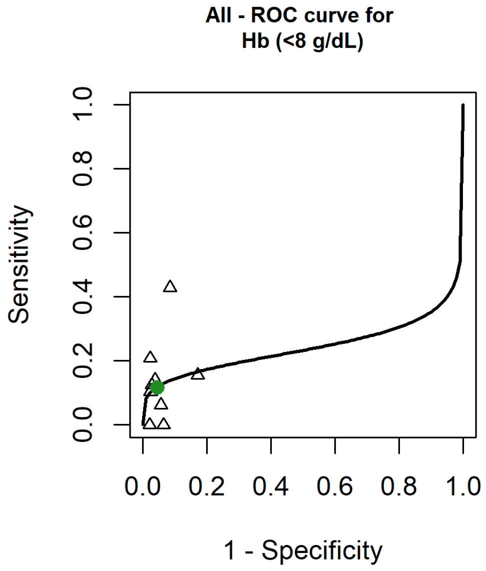
All - ROC curve for
Hb (<8 g/dL)
Sensitivity
0.0 0.2 0.4 0.6 0.8 1.0
1 - Specificity
0.0 0.2 0.4 0.6 0.8 1.0

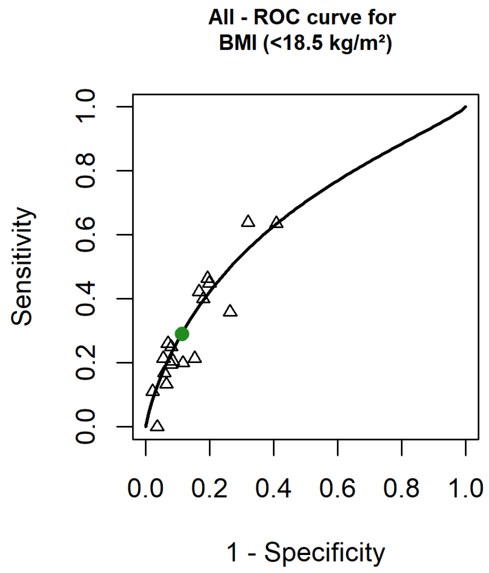
All - ROC curve for
BMI (<18.5 kg/m²)
Sensitivity
0.0 0.2 0.4 0.6 0.8 1.0
1 - Specificity
0.0 0.2 0.4 0.6 0.8 1.0

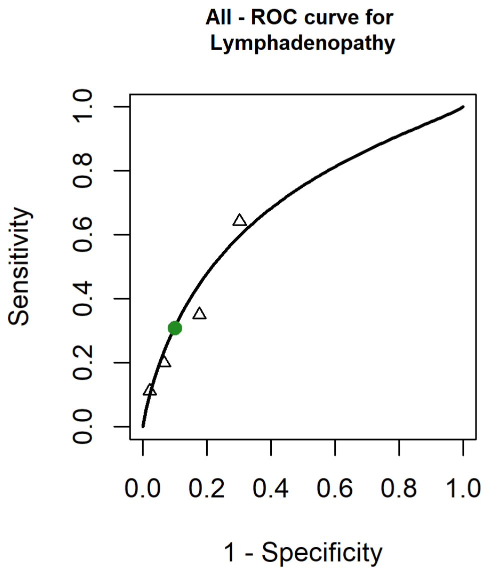
All - ROC curve for
Lymphadenopathy
Sensitivity
0.0 0.2 0.4 0.6 0.8 1.0
1 - Specificity
0.0 0.2 0.4 0.6 0.8 1.0

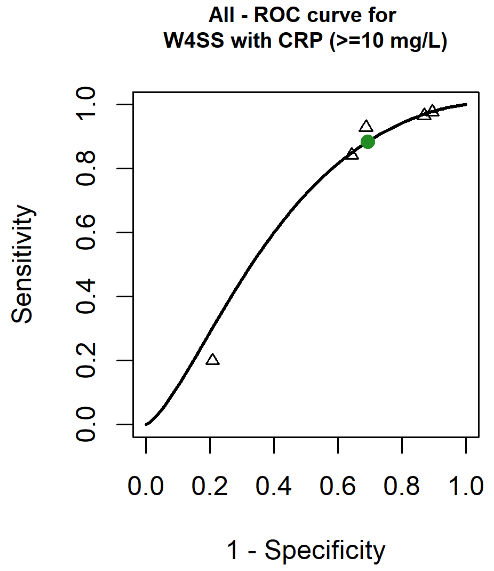
All - ROC curve for
W4SS with CRP (>=10 mg/L)
Sensitivity
1 - Specificity
0.0
0.2
0.4
0.6
0.8
1.0

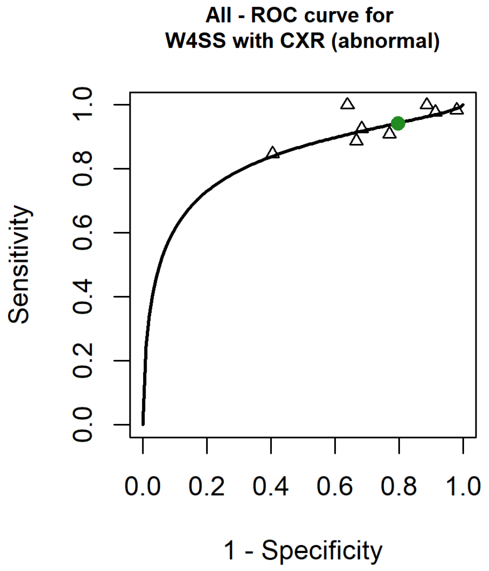
All - ROC curve for
W4SS with CXR (abnormal)
Sensitivity
1 - Specificity
0.0
0.2
0.4
0.6
0.8
1.0

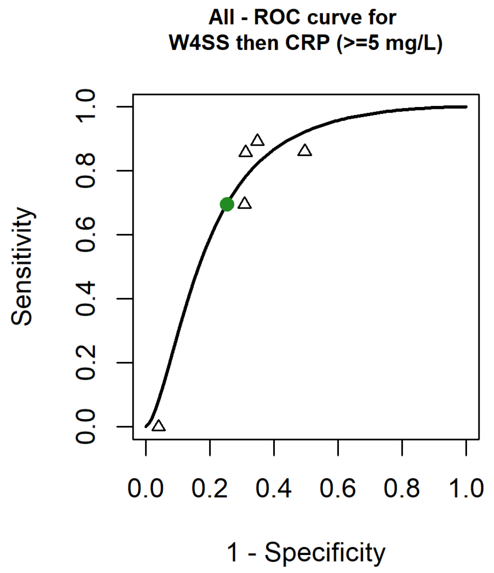
All - ROC curve for
W4SS then CRP (>=5 mg/L)
Sensitivity
1 - Specificity
0.0
0.2
0.4
0.6
0.8
1.0

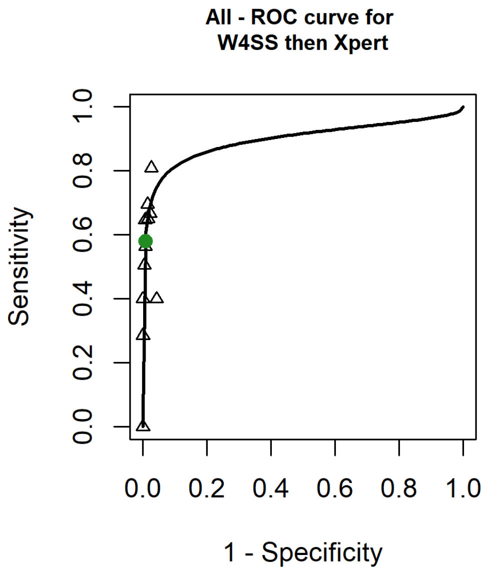
All - ROC curve for
W4SS then Xpert
Sensitivity
1 - Specificity
0.0
0.2
0.4
0.6
0.8
1.0

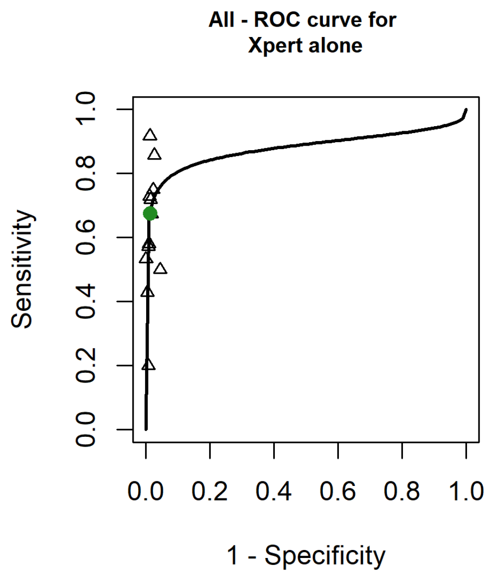
All - ROC curve for
Xpert alone
Sensitivity
0.0
0.2
0.4
0.6
0.8
1.0
0.0
0.2
0.4
0.6
0.8
1.0
1 - Specificity

Figure S4B - Summary receiver operating characteristics curves comparing each test and WHO four-symptom screen for the detection of tuberculosis in outpatients (on ART)

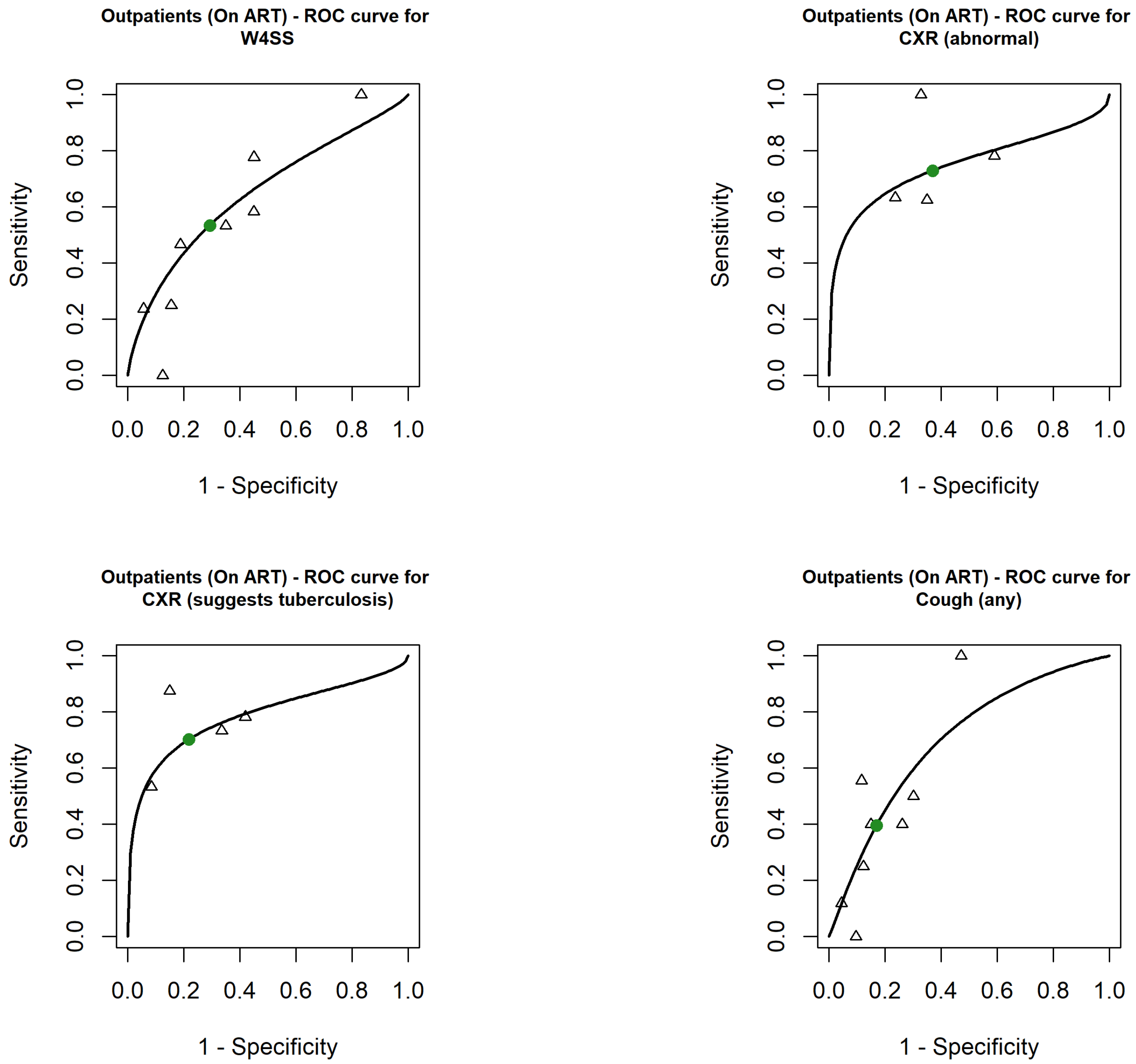

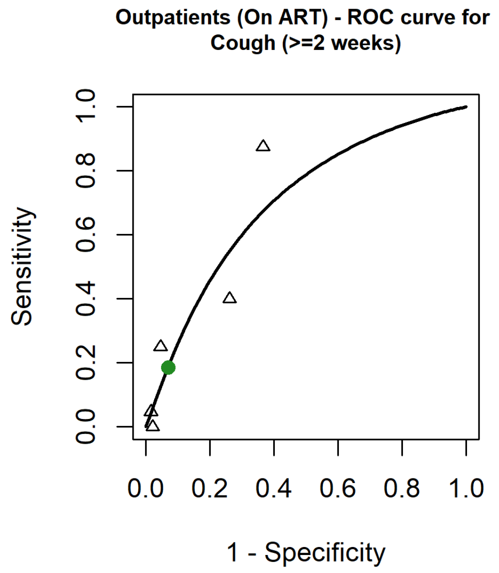
Outpatients (On ART) - ROC curve for
Cough (>=2 weeks)
Sensitivity
1 - Specificity
0.0
0.2
0.4
0.6
0.8
1.0

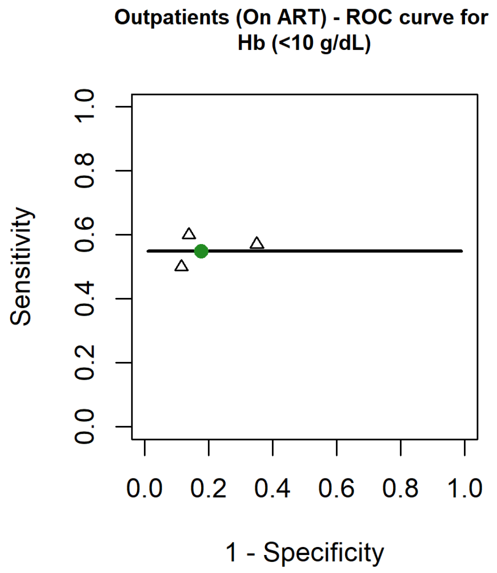
Outpatients (On ART) - ROC curve for
Hb (<10 g/dL)
Sensitivity
1 - Specificity
0.0
0.2
0.4
0.6
0.8
1.0

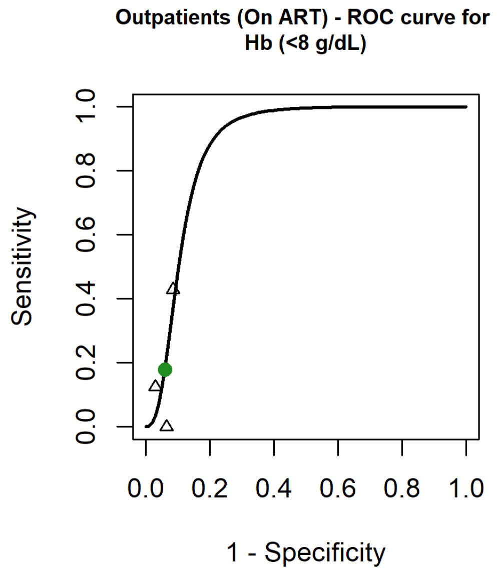
Outpatients (On ART) - ROC curve for
Hb (<8 g/dL)
Sensitivity
1 - Specificity
0.0
0.2
0.4
0.6
0.8
1.0

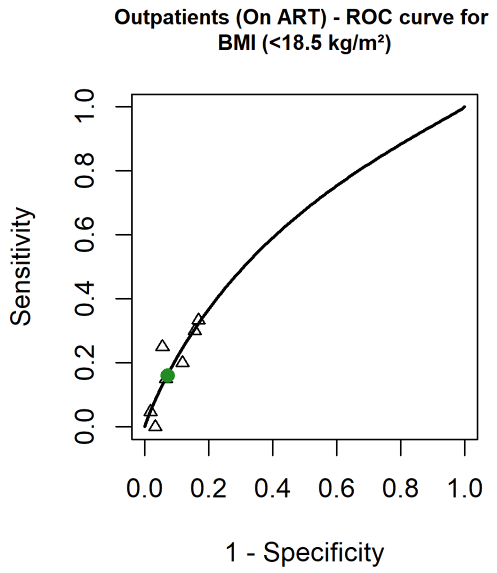
Outpatients (On ART) - ROC curve for
BMI (<18.5 kg/m²)
Sensitivity
1 - Specificity
0.0
0.2
0.4
0.6
0.8
1.0

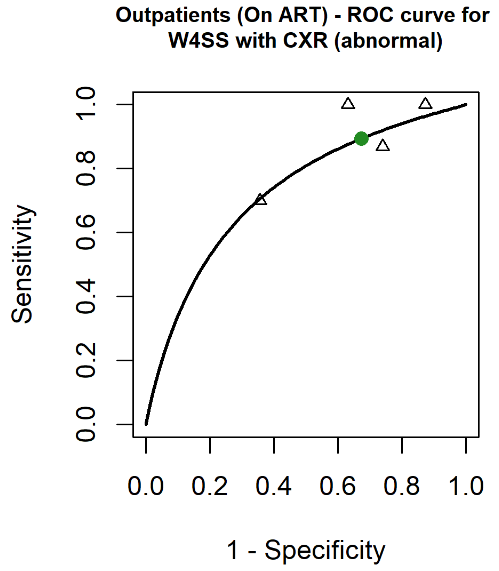
Outpatients (On ART) - ROC curve for
W4SS with CXR (abnormal)
Sensitivity
0.0
0.2
0.4
0.6
0.8
1.0
1 - Specificity

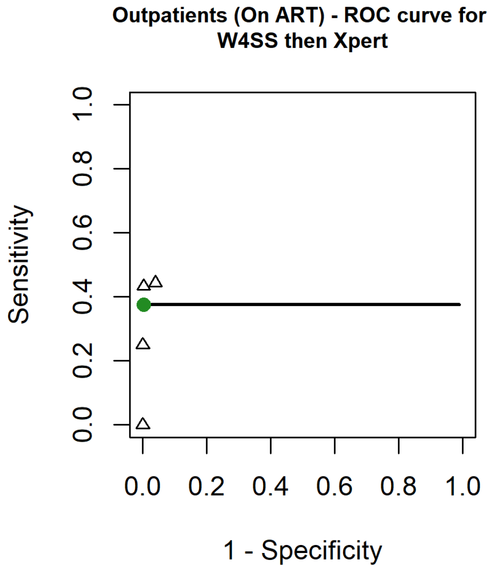
Outpatients (On ART) - ROC curve for
W4SS then Xpert
Sensitivity
0.0
0.2
0.4
0.6
0.8
1.0
1 - Specificity

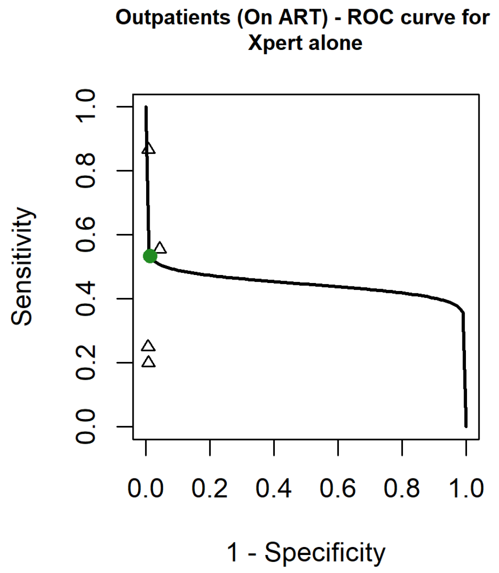
Outpatients (On ART) - ROC curve for
Xpert alone
Sensitivity
0.0
0.2
0.4
0.6
0.8
1.0
1 - Specificity

Figure S4C - Summary receiver operating characteristics curves comparing each test and WHO four-symptom screen for the detection of tuberculosis in outpatients (not on ART)

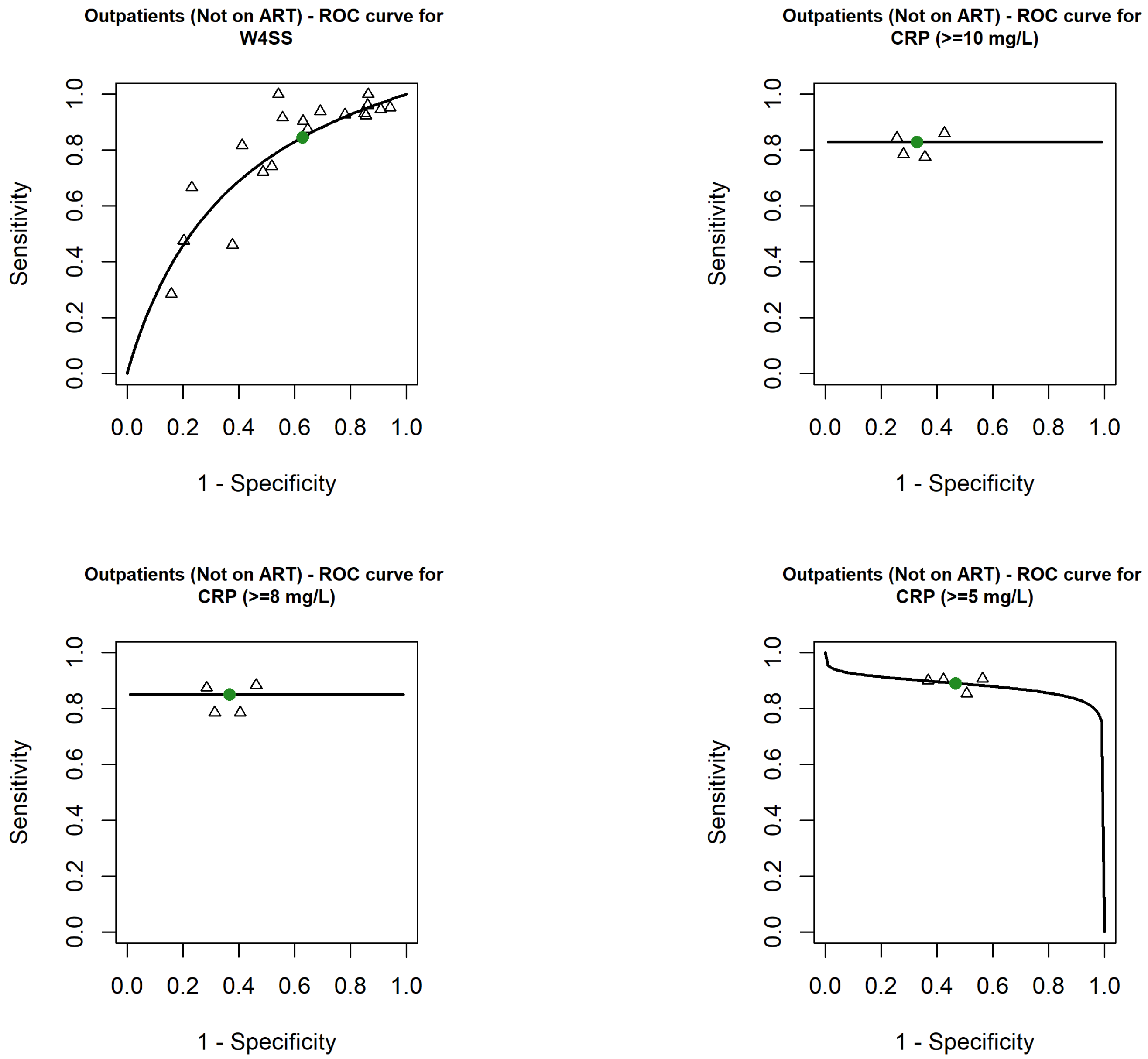

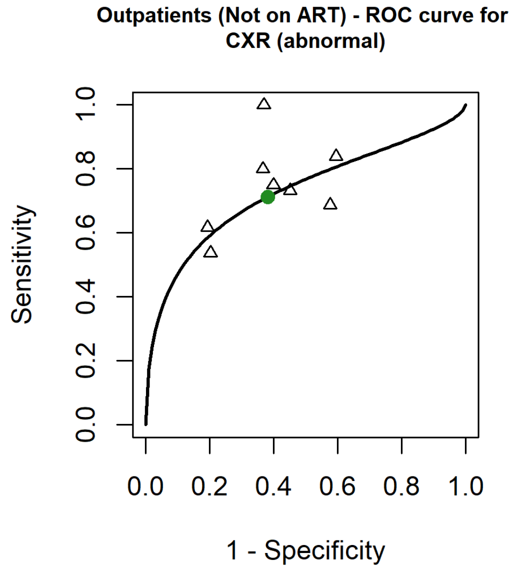
Outpatients (Not on ART) - ROC curve for
CXR (abnormal)
Sensitivity
0.0 0.2 0.4 0.6 0.8 1.0
1 - Specificity

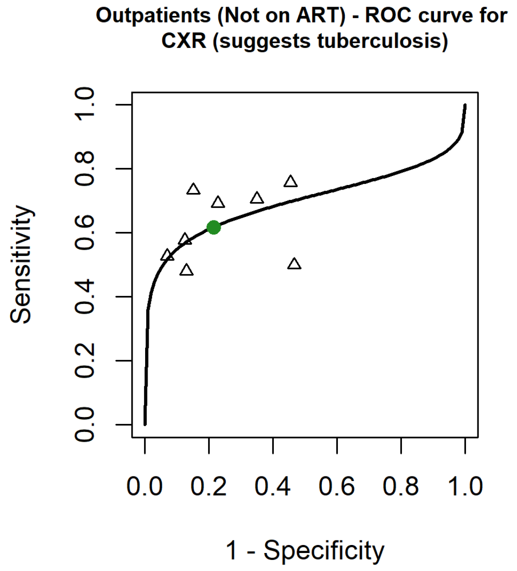
Outpatients (Not on ART) - ROC curve for
CXR (suggests tuberculosis)
Sensitivity
0.0 0.2 0.4 0.6 0.8 1.0
1 - Specificity

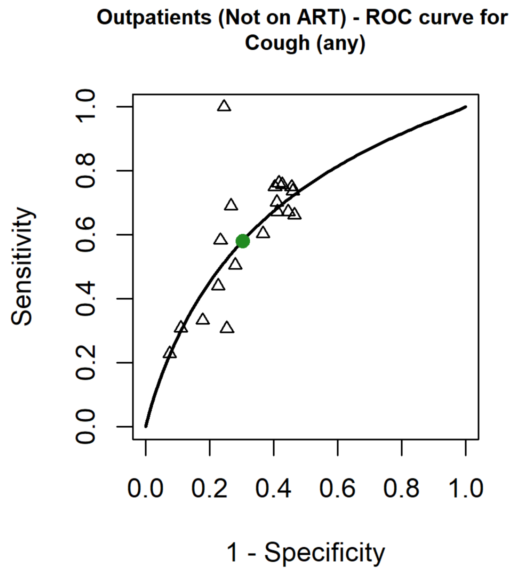
Outpatients (Not on ART) - ROC curve for
Cough (any)
Sensitivity
0.0 0.2 0.4 0.6 0.8 1.0
1 - Specificity

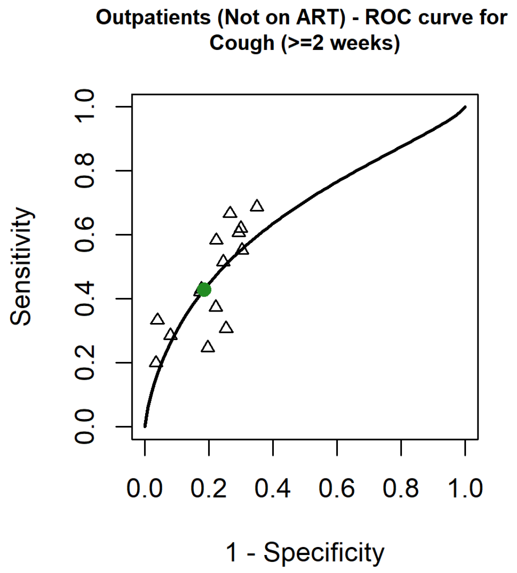
Outpatients (Not on ART) - ROC curve for
Cough (>=2 weeks)
Sensitivity
0.0 0.2 0.4 0.6 0.8 1.0
1 - Specificity

Outpatients (Not on ART) - ROC curve for Hb (<10 g/dL)

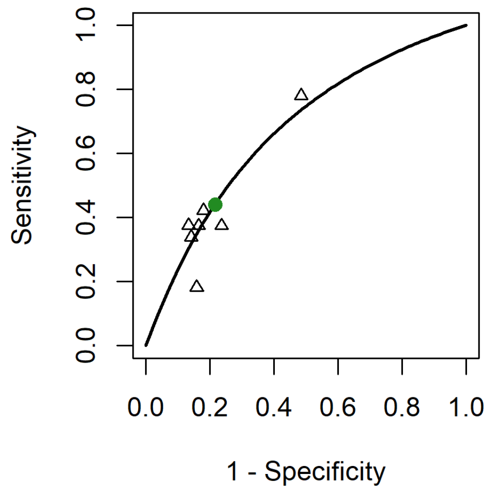

Outpatients (Not on ART) - ROC curve for Hb (<8 g/dL)

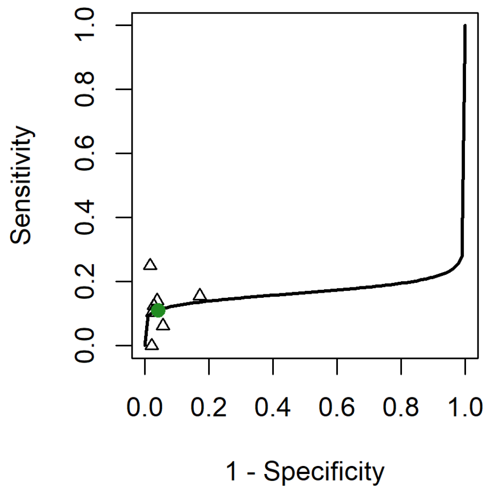

Outpatients (Not on ART) - ROC curve for BMI (<18.5 kg/m²)

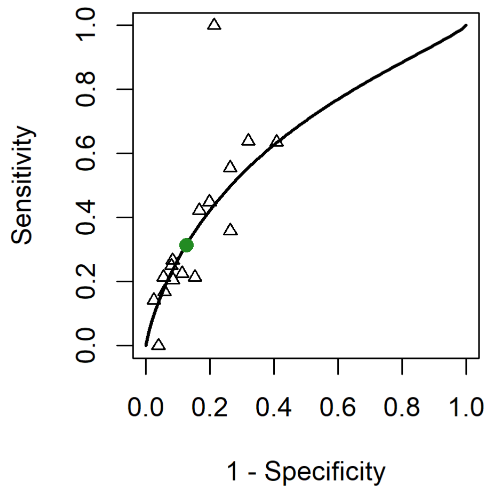

Outpatients (Not on ART) - ROC curve for Lymphadenopathy

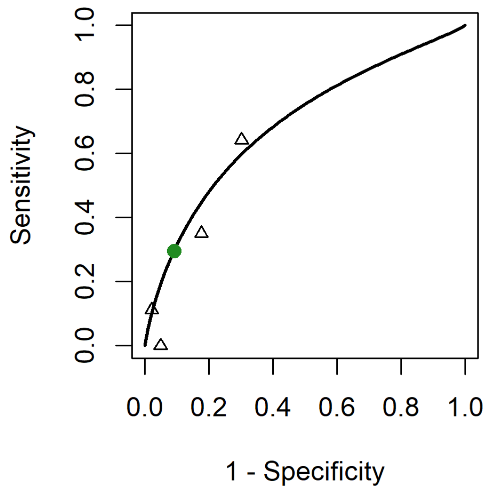

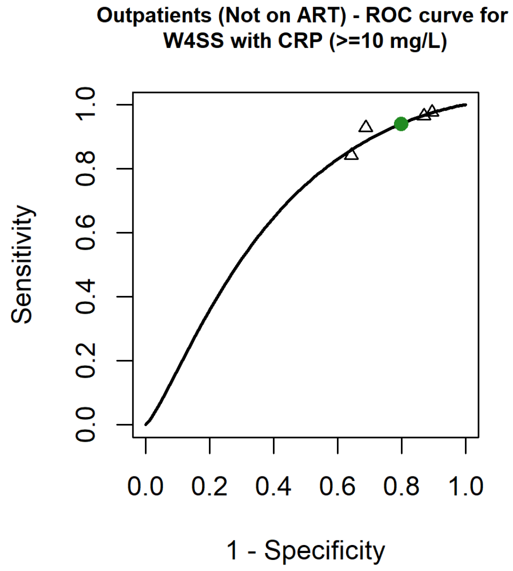
Outpatients (Not on ART) - ROC curve for
W4SS with CRP (>=10 mg/L)
Sensitivity
1 - Specificity
0.0
0.2
0.4
0.6
0.8
1.0

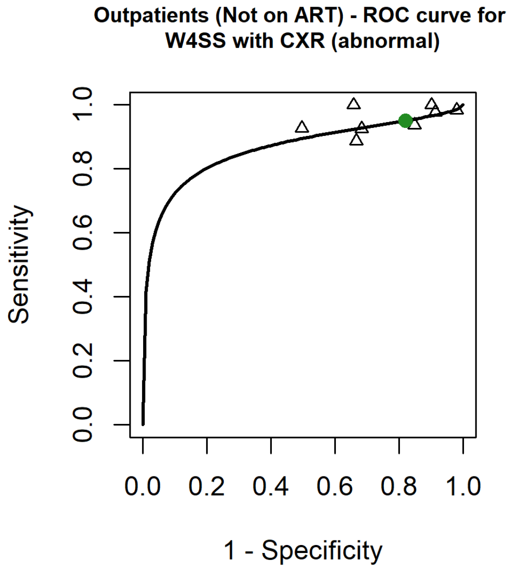
Outpatients (Not on ART) - ROC curve for
W4SS with CXR (abnormal)
Sensitivity
1 - Specificity
0.0
0.2
0.4
0.6
0.8
1.0

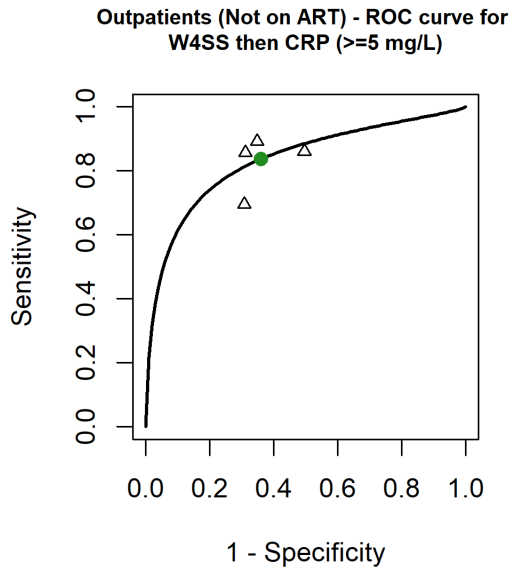
Outpatients (Not on ART) - ROC curve for
W4SS then CRP (>=5 mg/L)
Sensitivity
1 - Specificity
0.0
0.2
0.4
0.6
0.8
1.0

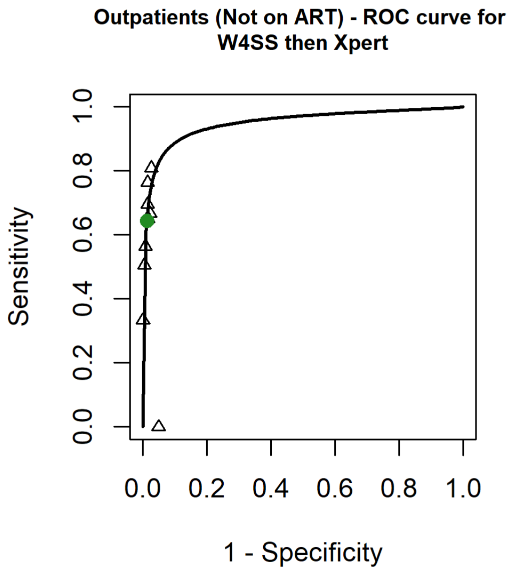
Outpatients (Not on ART) - ROC curve for
W4SS then Xpert
Sensitivity
1 - Specificity
0.0
0.2
0.4
0.6
0.8
1.0

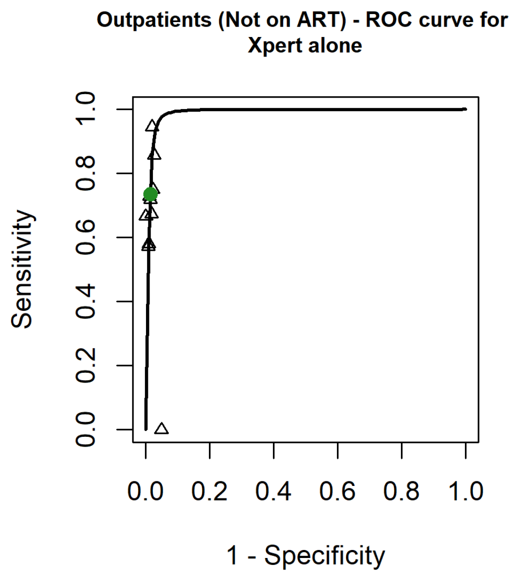

Outpatients (Not on ART) - ROC curve for
Xpert alone
Sensitivity
0.0
0.2
0.4
0.6
0.8
1.0
1 - Specificity

Figure S4D - Summary receiver operating characteristics curves comparing each test and WHO four-symptom screen for the detection of tuberculosis in participants with a CD4 cell count <=200 cells/µL

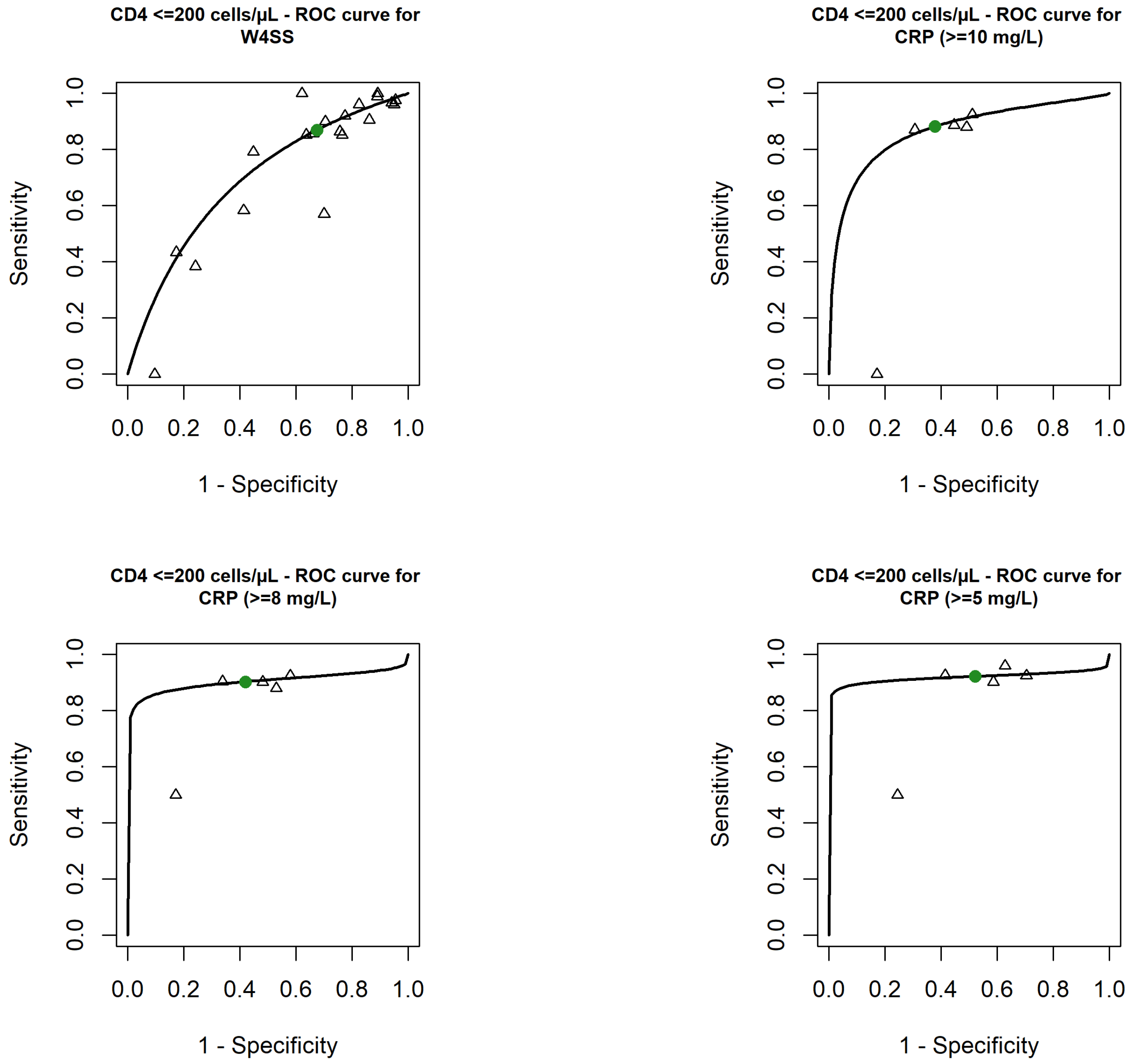

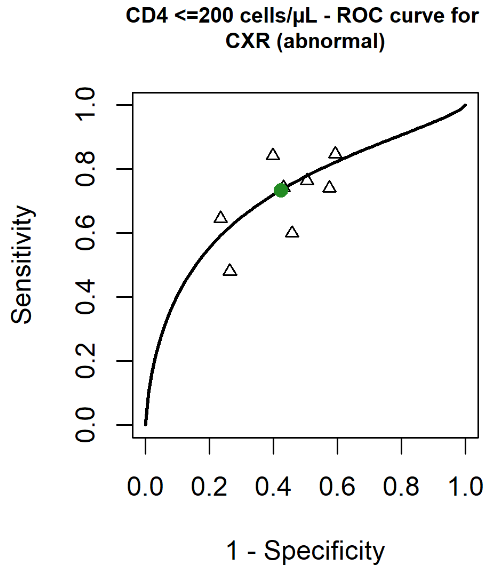
CD4 <=200 cells/µL - ROC curve for
CXR (abnormal)
Sensitivity
1 - Specificity
0.0
0.2
0.4
0.6
0.8
1.0

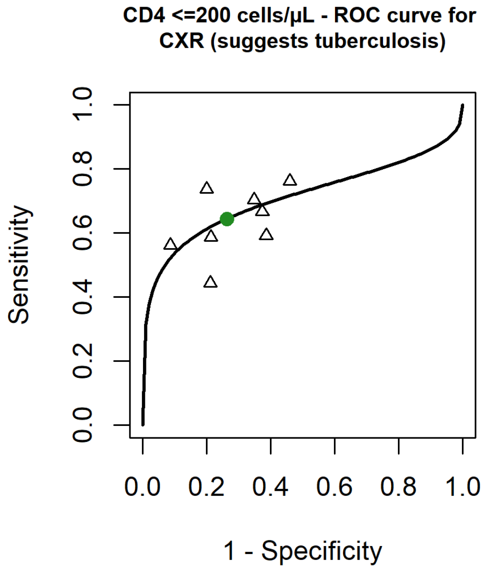
CD4 <=200 cells/µL - ROC curve for
CXR (suggests tuberculosis)
Sensitivity
1 - Specificity
0.0
0.2
0.4
0.6
0.8
1.0

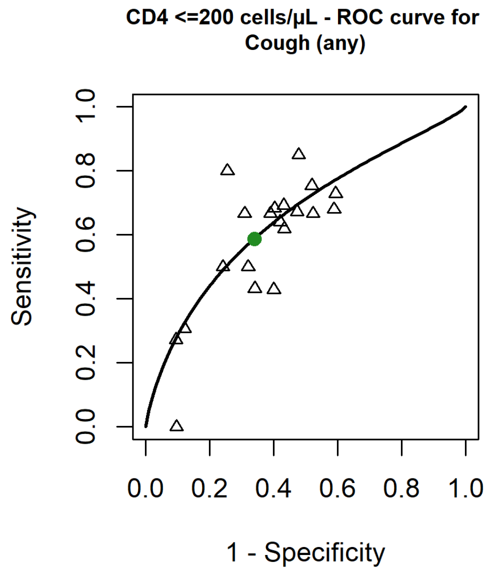
CD4 <=200 cells/µL - ROC curve for
Cough (any)
Sensitivity
1 - Specificity
0.0
0.2
0.4
0.6
0.8
1.0

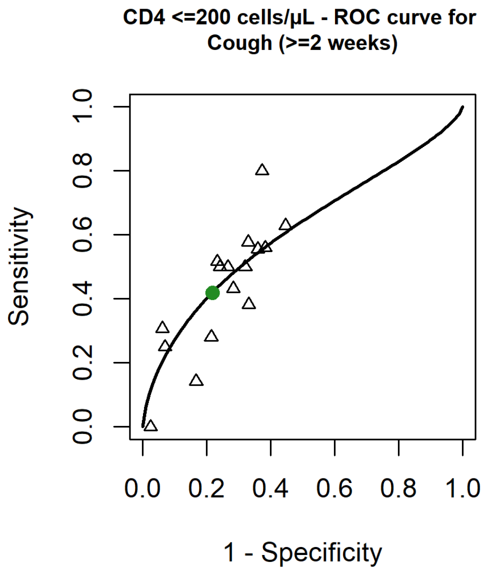
CD4 <=200 cells/µL - ROC curve for
Cough (>=2 weeks)
Sensitivity
1 - Specificity
0.0
0.2
0.4
0.6
0.8
1.0

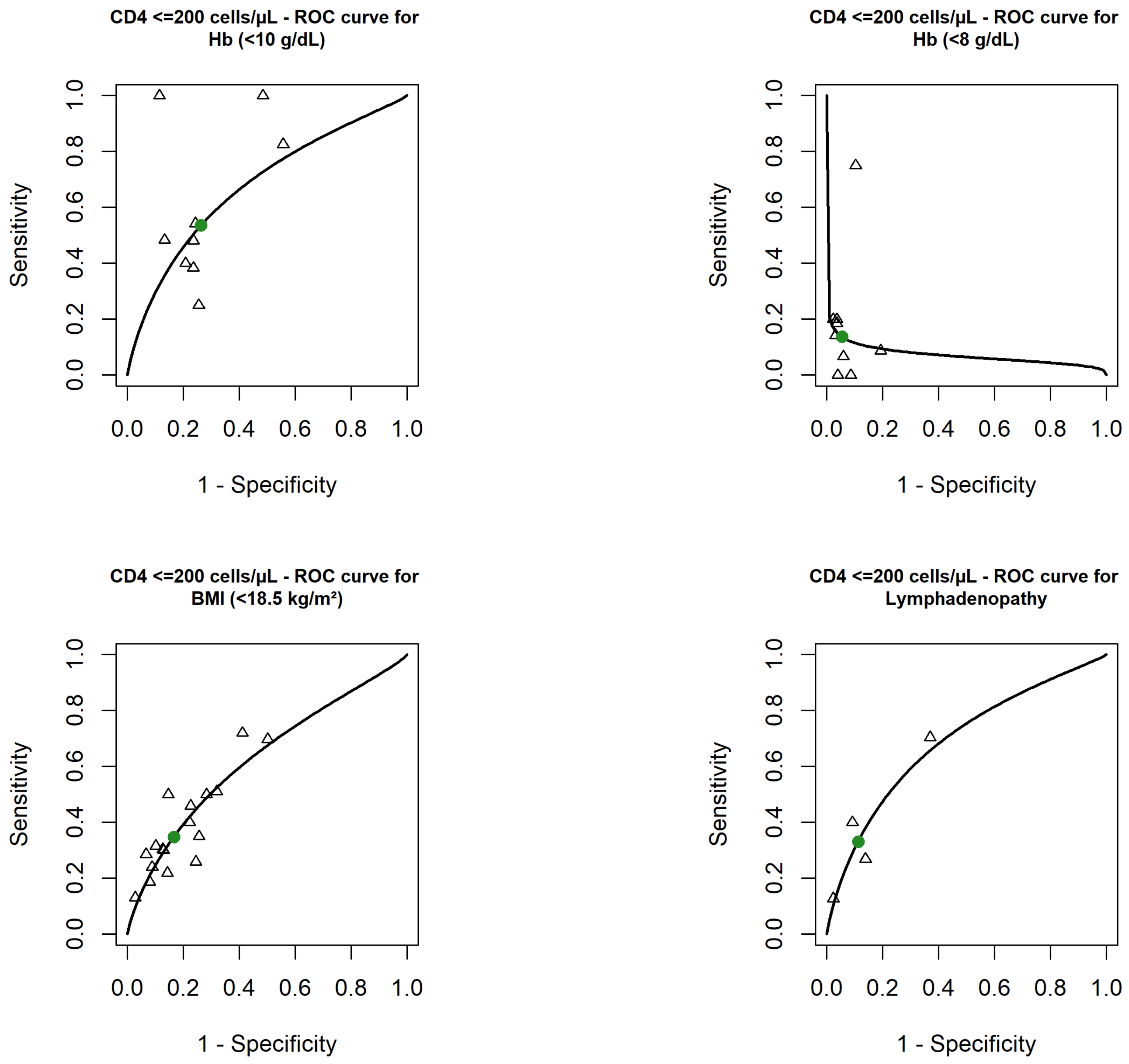
CD4 <=200 cells/µL - ROC curve for Hb (<10 g/dL)
Sensitivity
1 - Specificity
0.0 0.2 0.4 0.6 0.8 1.0
CD4 <=200 cells/µL - ROC curve for Hb (<8 g/dL)
Sensitivity
1 - Specificity
0.0 0.2 0.4 0.6 0.8 1.0
CD4 <=200 cells/µL - ROC curve for BMI (<18.5 kg/m²)
Sensitivity
1 - Specificity
0.0 0.2 0.4 0.6 0.8 1.0
CD4 <=200 cells/µL - ROC curve for Lymphadenopathy
Sensitivity
1 - Specificity
0.0 0.2 0.4 0.6 0.8 1.0

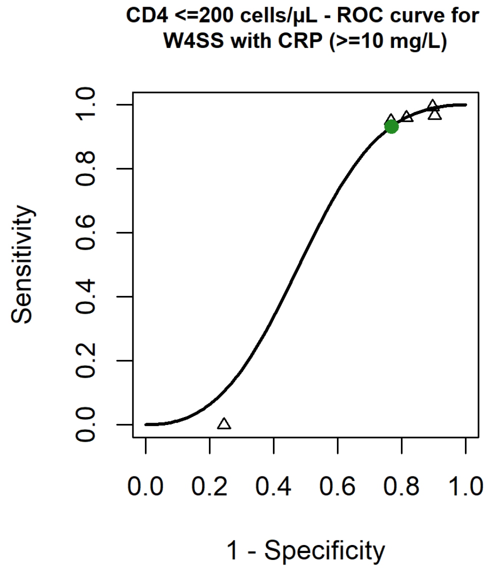
CD4 <=200 cells/µL - ROC curve for
W4SS with CRP (>=10 mg/L)
Sensitivity
1 - Specificity
0.0
0.2
0.4
0.6
0.8
1.0

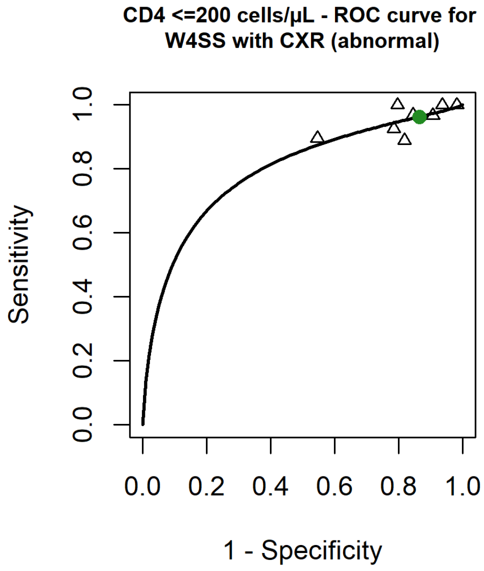
CD4 <=200 cells/µL - ROC curve for
W4SS with CXR (abnormal)
Sensitivity
1 - Specificity
0.0
0.2
0.4
0.6
0.8
1.0

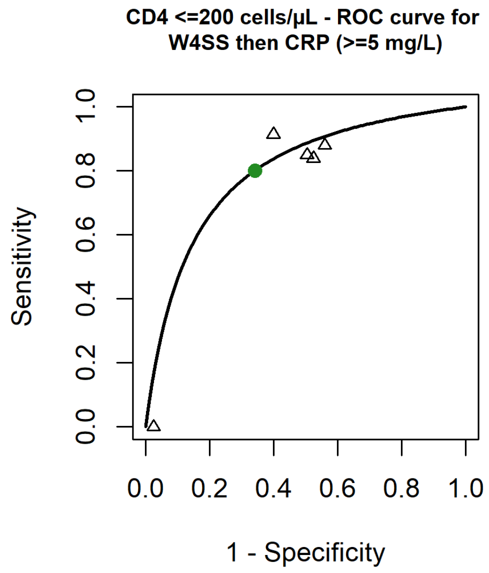
CD4 <=200 cells/µL - ROC curve for
W4SS then CRP (>=5 mg/L)
Sensitivity
1 - Specificity
0.0
0.2
0.4
0.6
0.8
1.0

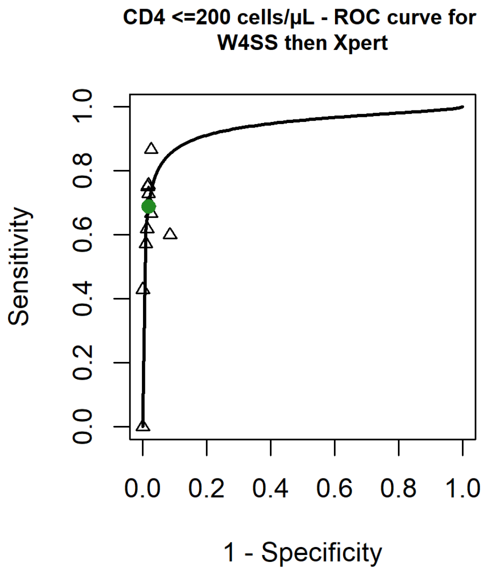
CD4 <=200 cells/µL - ROC curve for
W4SS then Xpert
Sensitivity
1 - Specificity
0.0
0.2
0.4
0.6
0.8
1.0

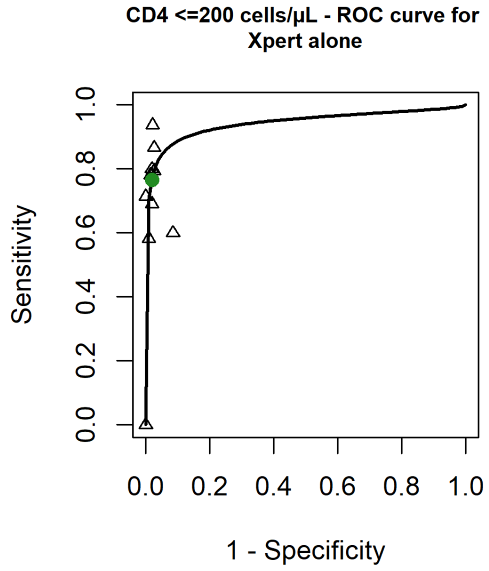
CD4 <=200 cells/µL - ROC curve for
Xpert alone
Sensitivity
0.0
0.2
0.4
0.6
0.8
1.0
0.0
0.2
0.4
0.6
0.8
1.0
1 - Specificity

Figure S4E - Summary receiver operating characteristics curves comparing each test and WHO four-symptom screen for the detection of tuberculosis in in participants with a CD4 cell count >200 cells/μL

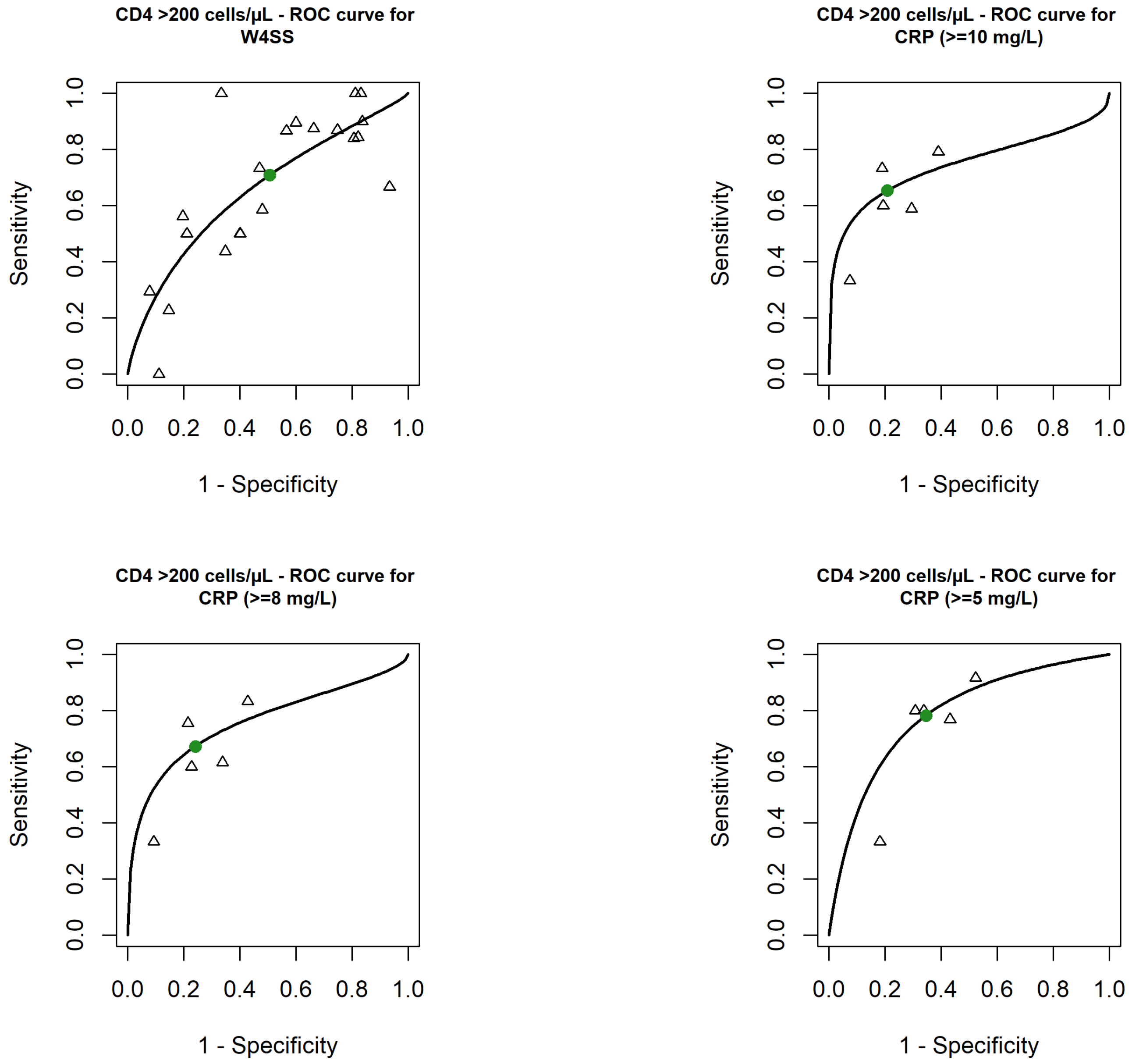

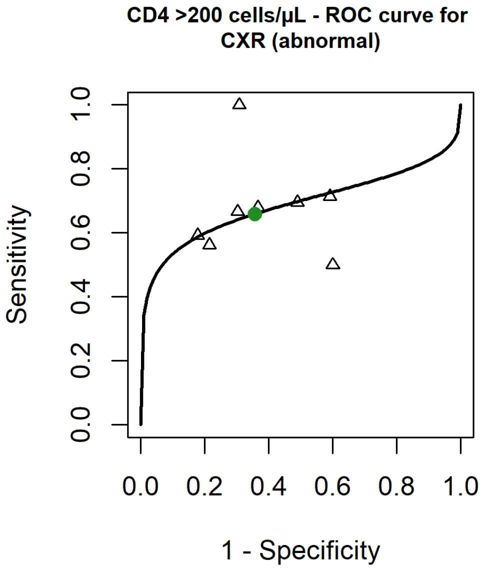
CD4 >200 cells/µL - ROC curve for
CXR (abnormal)
Sensitivity
0.0 0.2 0.4 0.6 0.8 1.0
1 - Specificity

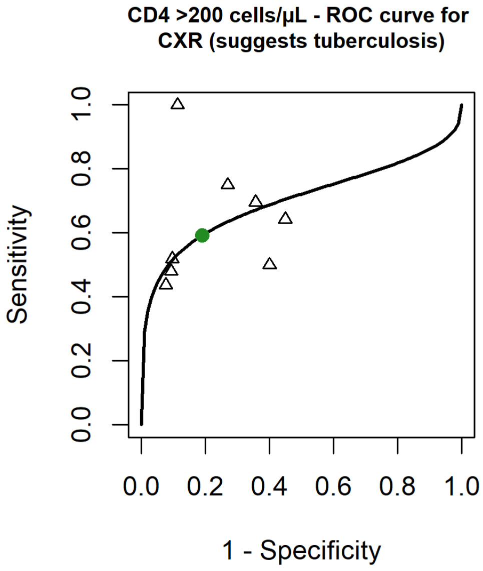
CD4 >200 cells/µL - ROC curve for
CXR (suggests tuberculosis)
Sensitivity
0.0 0.2 0.4 0.6 0.8 1.0
1 - Specificity

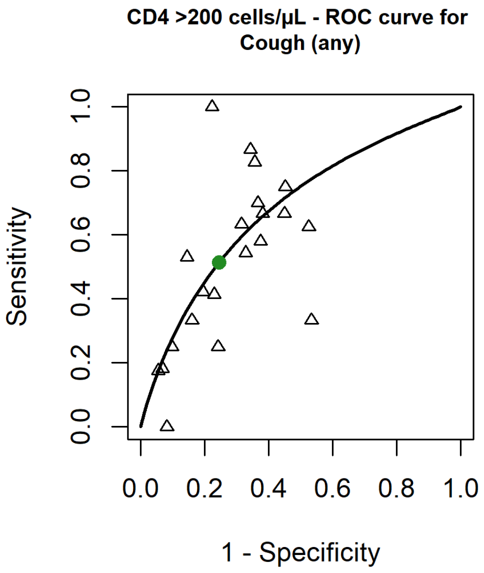
CD4 >200 cells/µL - ROC curve for
Cough (any)
Sensitivity
0.0 0.2 0.4 0.6 0.8 1.0
1 - Specificity

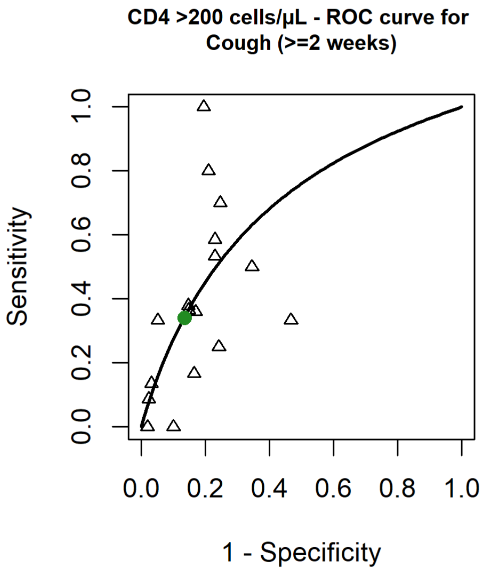
CD4 >200 cells/µL - ROC curve for
Cough (>=2 weeks)
Sensitivity
0.0 0.2 0.4 0.6 0.8 1.0
1 - Specificity

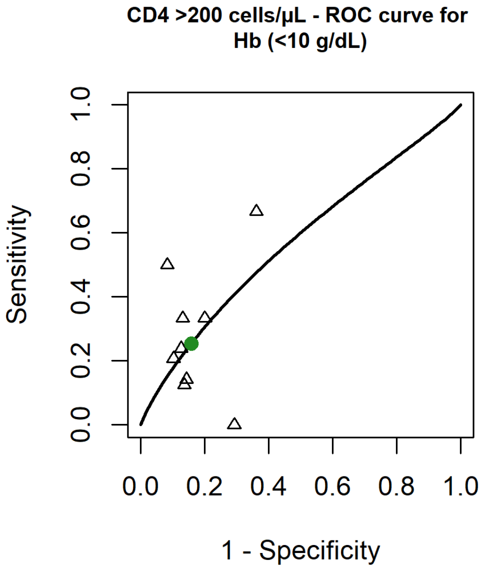
CD4 >200 cells/µL - ROC curve for
Hb (<10 g/dL)
Sensitivity
1 - Specificity
0.0 0.2 0.4 0.6 0.8 1.0

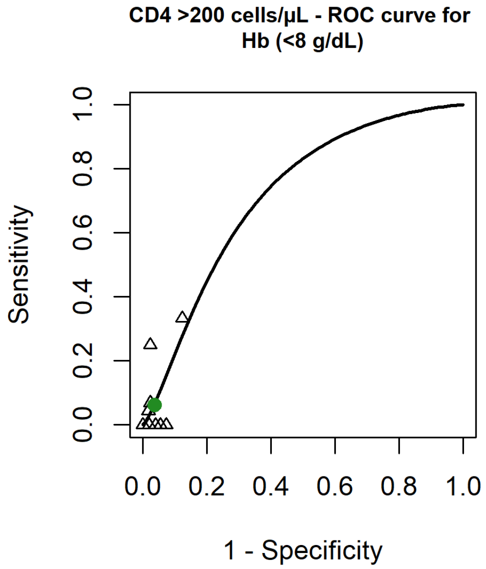
CD4 >200 cells/µL - ROC curve for
Hb (<8 g/dL)
Sensitivity
1 - Specificity
0.0 0.2 0.4 0.6 0.8 1.0

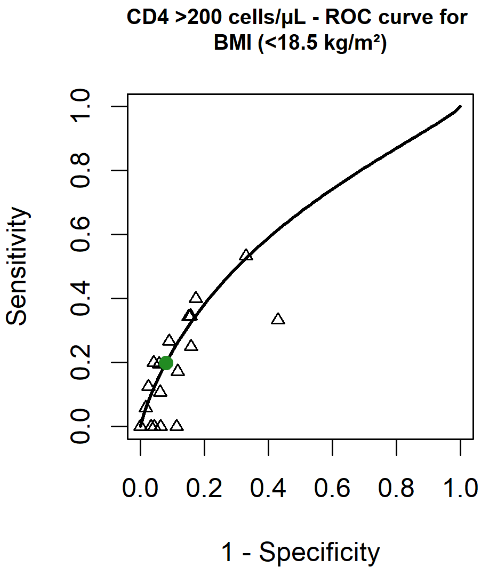
CD4 >200 cells/µL - ROC curve for
BMI (<18.5 kg/m²)
Sensitivity
1 - Specificity
0.0 0.2 0.4 0.6 0.8 1.0

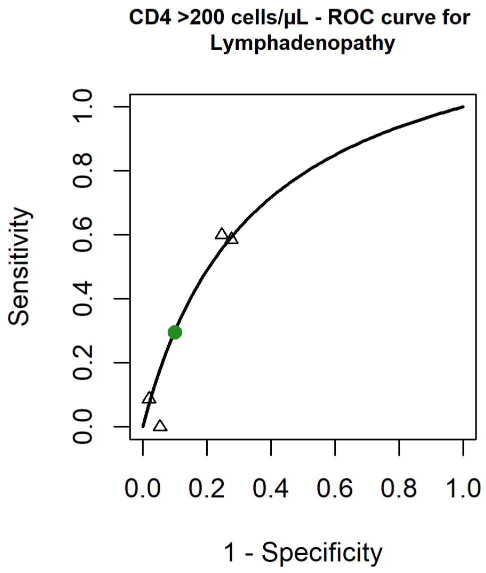
CD4 >200 cells/µL - ROC curve for
Lymphadenopathy
Sensitivity
1 - Specificity
0.0 0.2 0.4 0.6 0.8 1.0

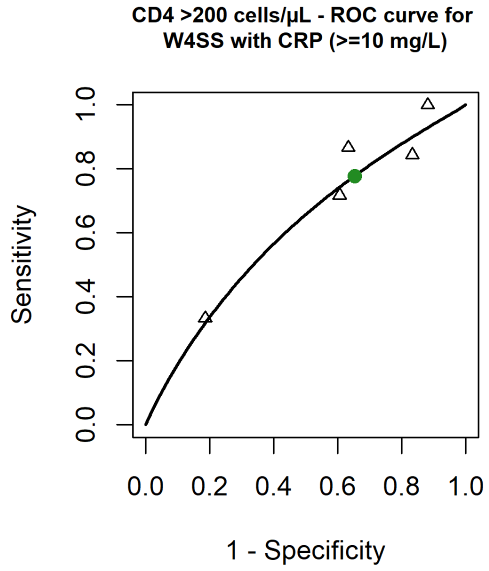
CD4 >200 cells/µL - ROC curve for
W4SS with CRP (>=10 mg/L)
Sensitivity
0.0
0.2
0.4
0.6
0.8
1.0
1 - Specificity

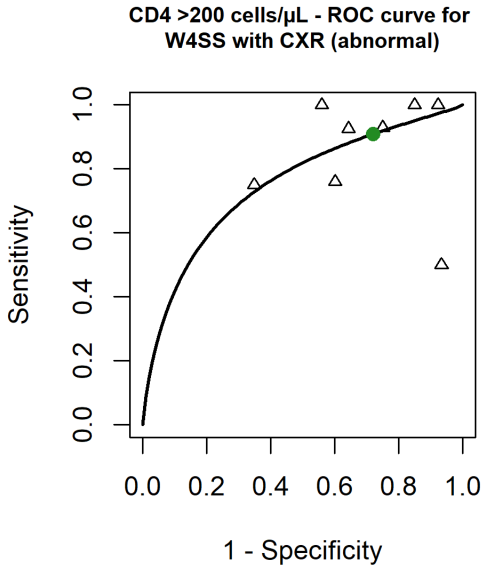
CD4 >200 cells/µL - ROC curve for
W4SS with CXR (abnormal)
Sensitivity
0.0
0.2
0.4
0.6
0.8
1.0
1 - Specificity

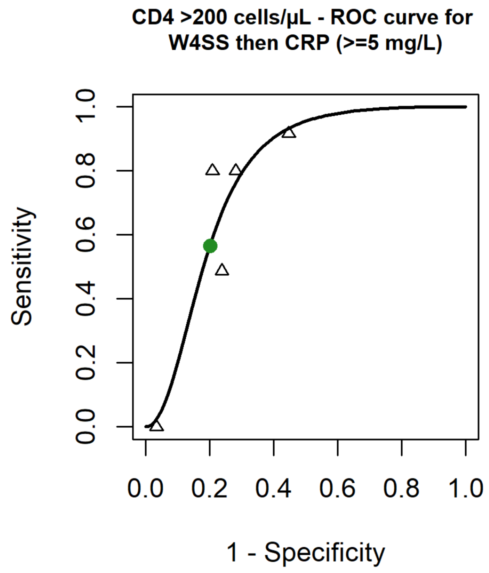
CD4 >200 cells/µL - ROC curve for
W4SS then CRP (>=5 mg/L)
Sensitivity
0.0
0.2
0.4
0.6
0.8
1.0
1 - Specificity

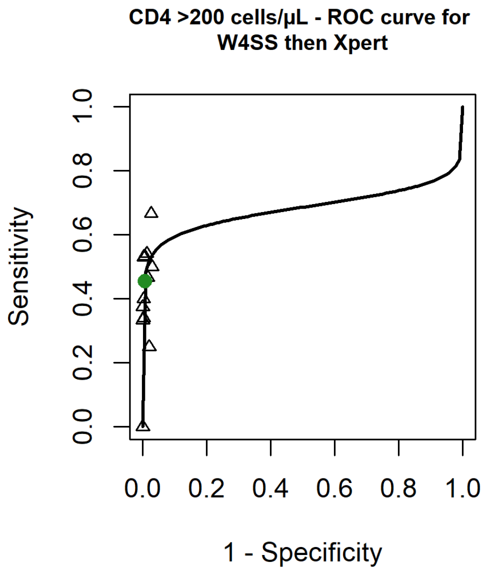
CD4 >200 cells/µL - ROC curve for
W4SS then Xpert
Sensitivity
0.0
0.2
0.4
0.6
0.8
1.0
1 - Specificity

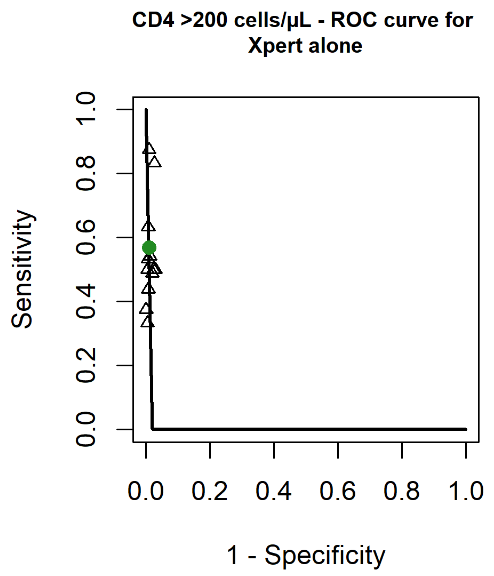
CD4 >200 cells/μL - ROC curve for
Xpert alone
Sensitivity
0.0
0.2
0.4
0.6
0.8
1.0
0.0
0.2
0.4
0.6
0.8
1.0
1 - Specificity

Figure S4F - Summary receiver operating characteristics curves comparing each test and WHO four-symptom screen for the detection of tuberculosis in pregnant participants

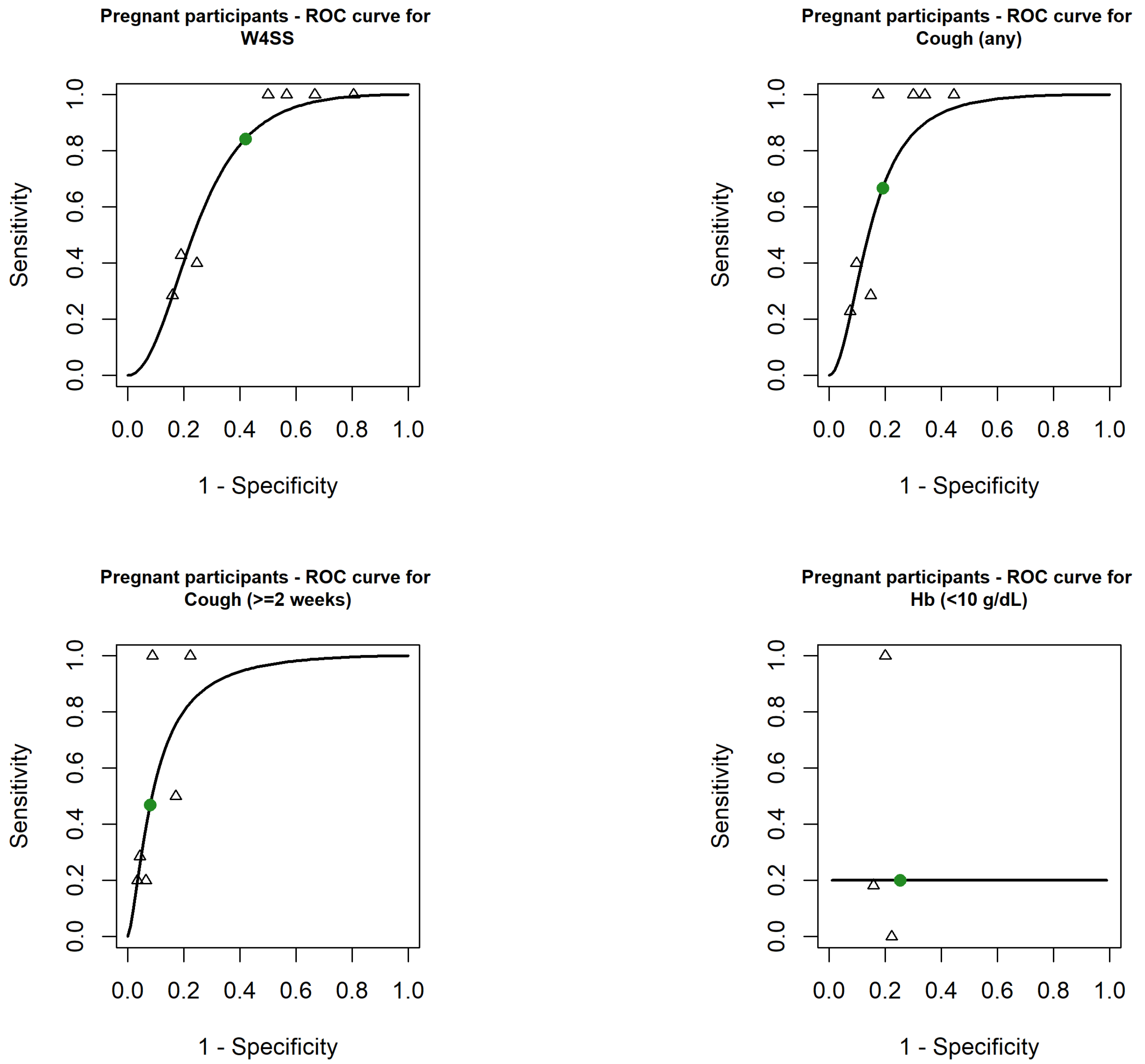

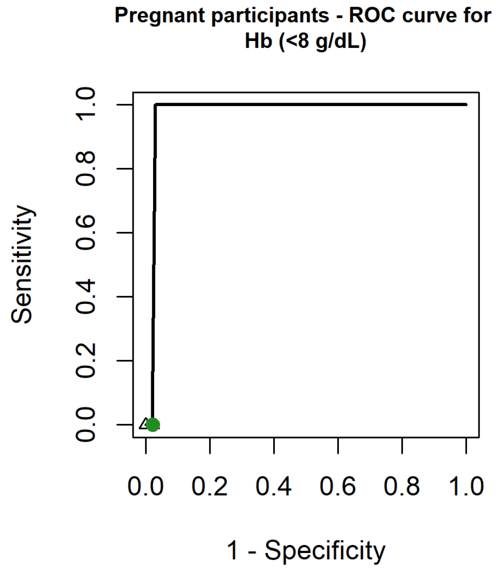
Pregnant participants - ROC curve for
Hb (<8 g/dL)
Sensitivity
0.0
0.2
0.4
0.6
0.8
1.0
1 - Specificity

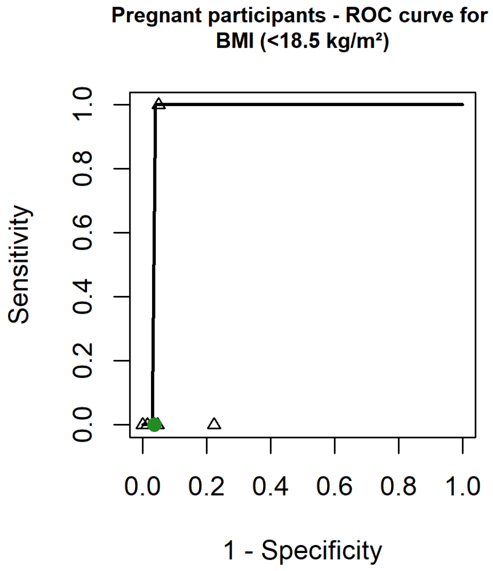
Pregnant participants - ROC curve for
BMI (<18.5 kg/m²)
Sensitivity
0.0
0.2
0.4
0.6
0.8
1.0
1 - Specificity

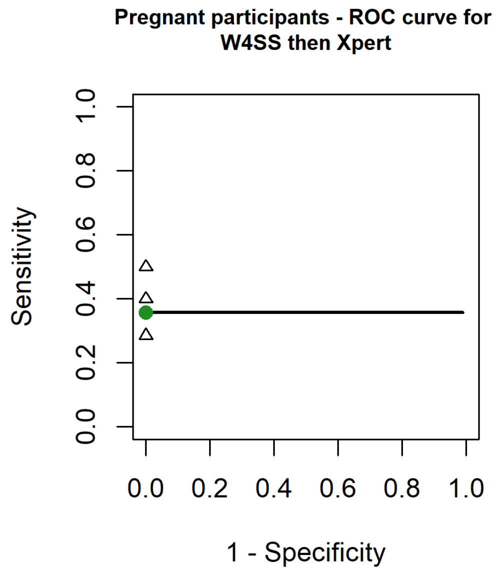
Pregnant participants - ROC curve for
W4SS then Xpert
Sensitivity
0.0
0.2
0.4
0.6
0.8
1.0
1 - Specificity

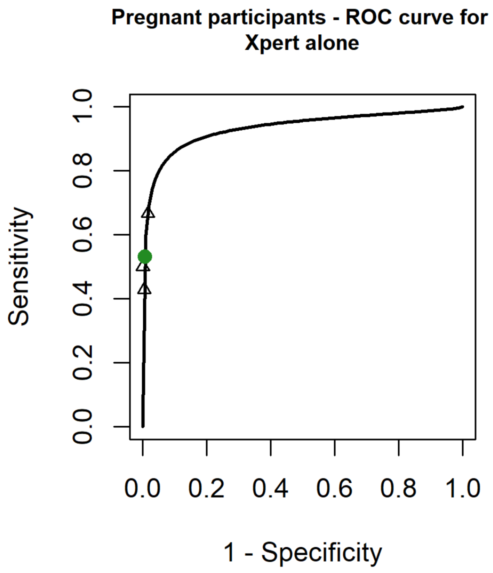
Pregnant participants - ROC curve for
Xpert alone
Sensitivity
0.0
0.2
0.4
0.6
0.8
1.0
1 - Specificity

Figure S5 - Funnel plots (for tests/strategies with >=10 studies available)

Figure S5A - Funnel plots in all participants

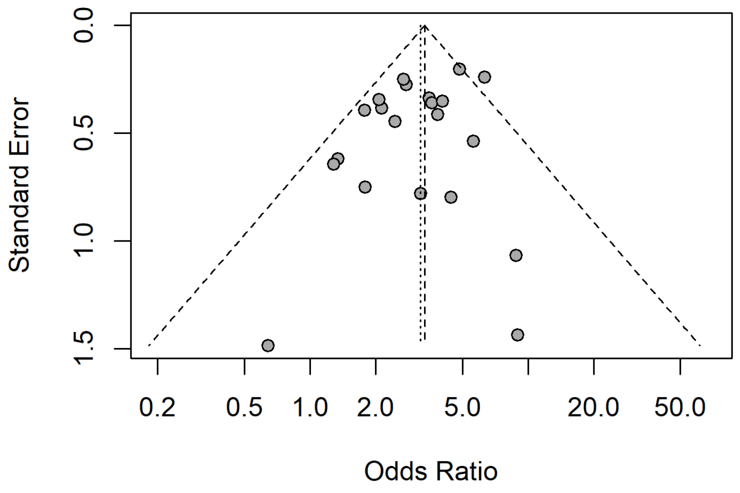

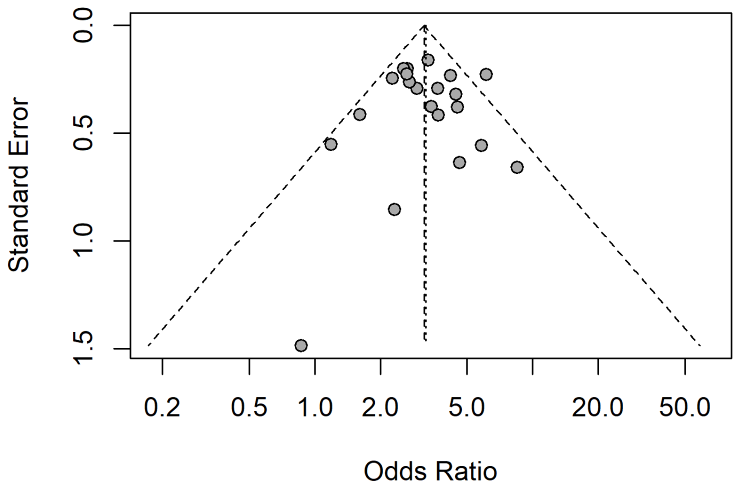

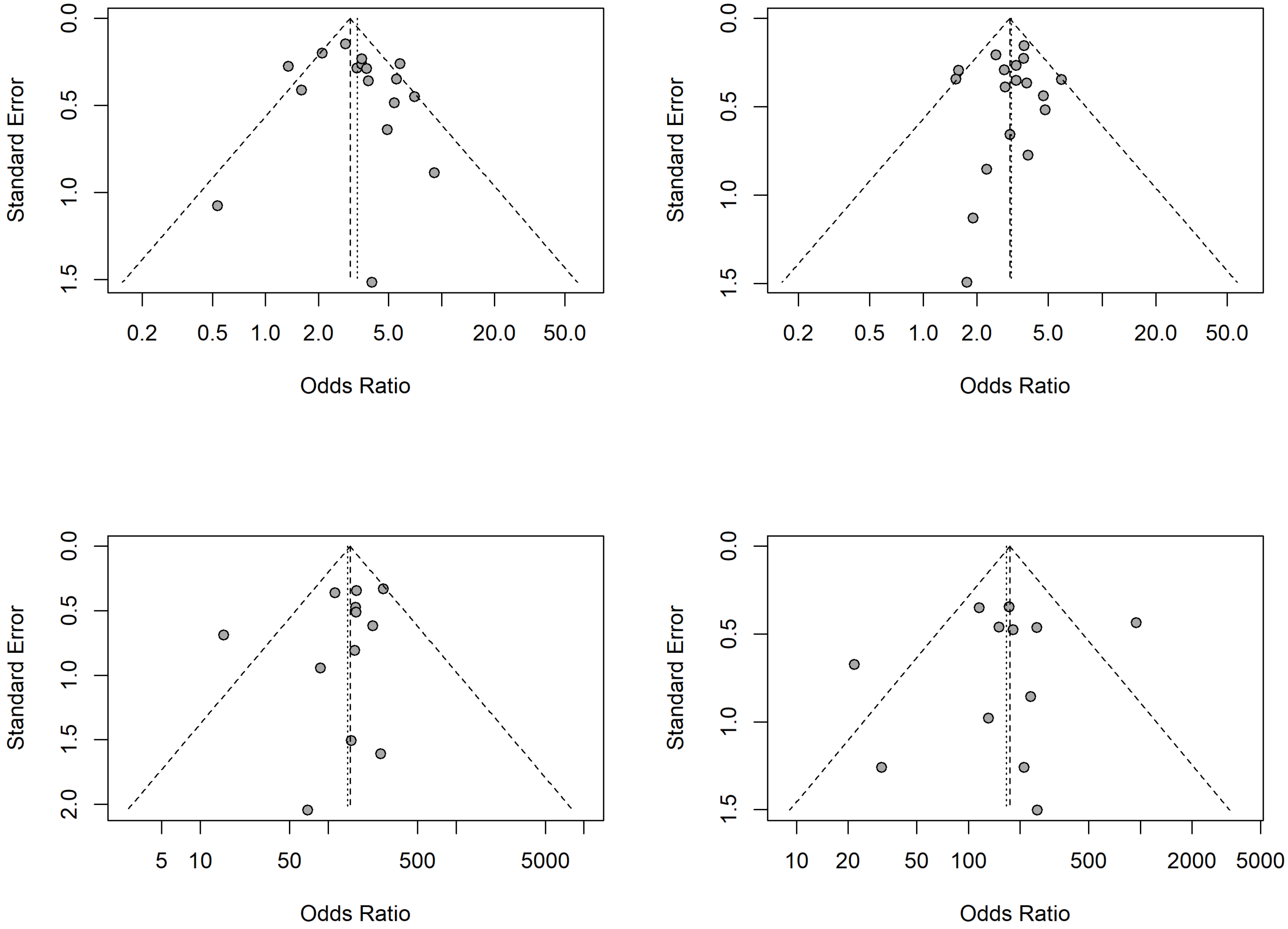
Standard Error
0.0
0.5
1.0
1.5
0.2
0.5
1.0
2.0
5.0
20.0
50.0
Odds Ratio
Standard Error
0.0
0.5
1.0
1.5
0.2
0.5
1.0
2.0
5.0
20.0
50.0
Odds Ratio
Standard Error
0.0
0.5
1.0
1.5
2.0
5
10
50
500
5000
Odds Ratio
Standard Error
0.0
0.5
1.0
1.5
10
20
50
100
500
2000
5000
Odds Ratio

Figure S5B - Funnel plots in outpatients (on ART)
Figure S5C - Funnel plots in outpatients (not on ART)

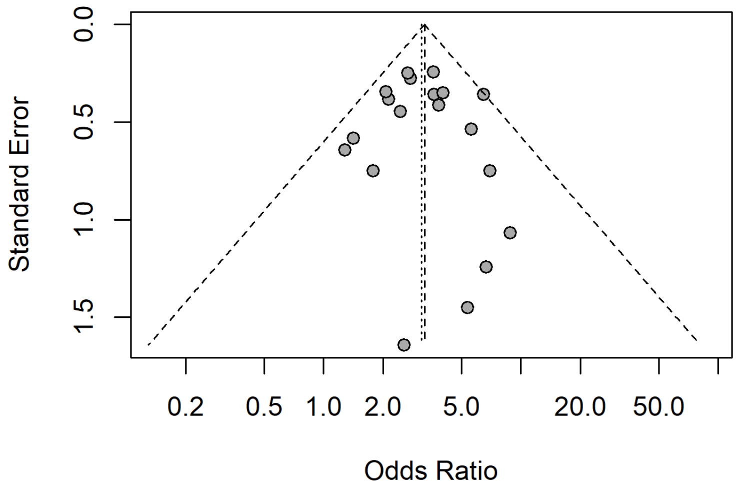

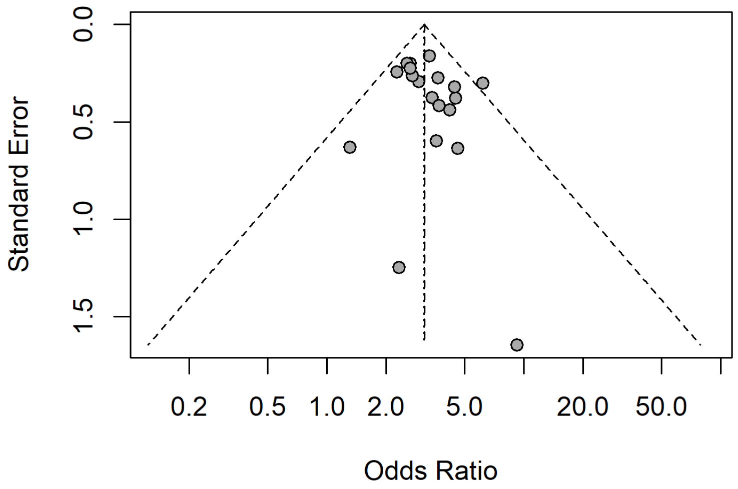

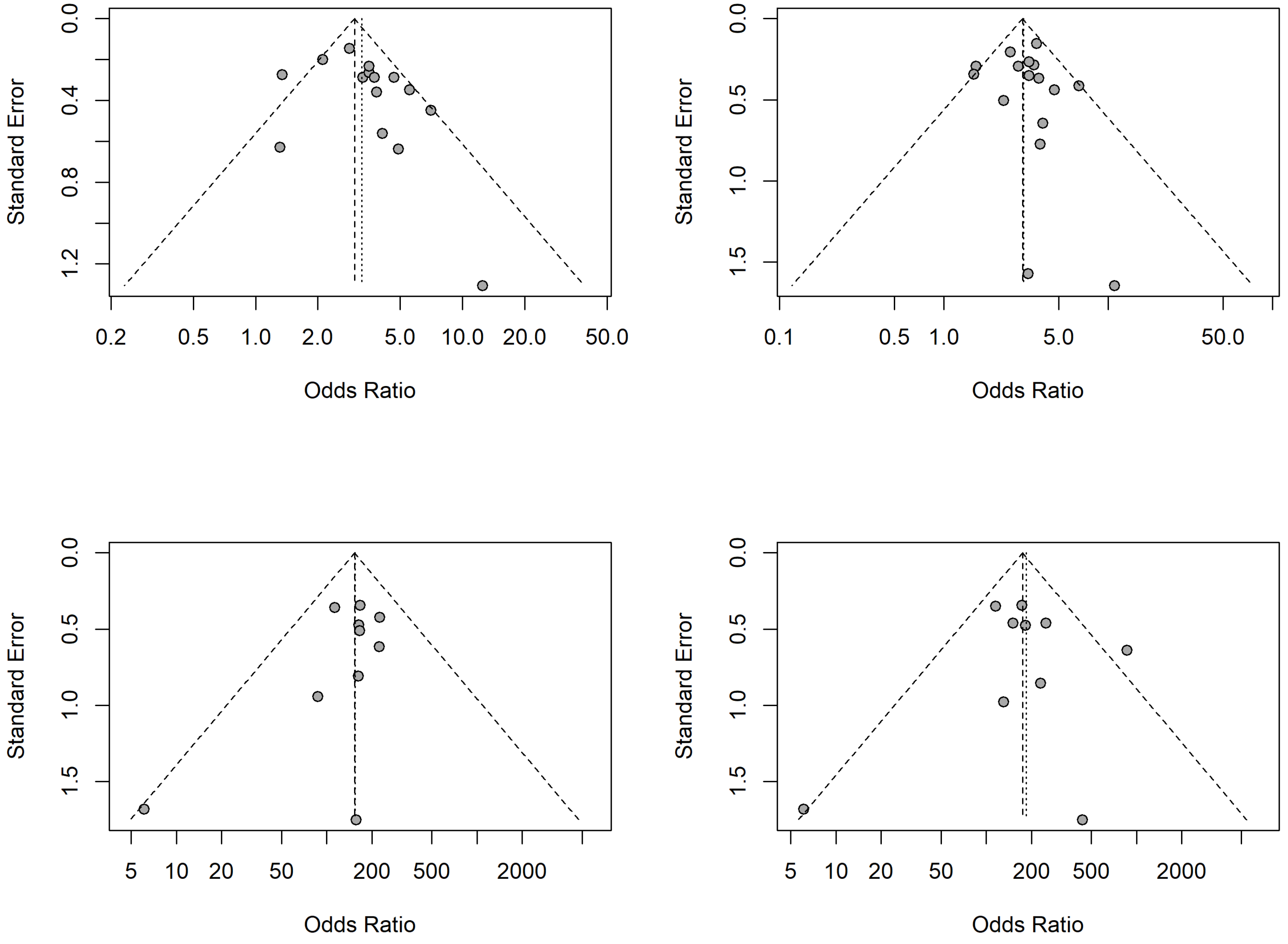

Standard Error
0.0
0.4
0.8
1.2
0.2
0.5
1.0
2.0
5.0
10.0
20.0
50.0
Odds Ratio
Standard Error
0.0
0.5
1.0
1.5
0.1
0.5
1.0
5.0
50.0
Odds Ratio
Standard Error
0.0
0.5
1.0
1.5
5
10
20
50
200
500
2000
Odds Ratio
Standard Error
0.0
0.5
1.0
1.5
5
10
20
50
200
500
2000
Odds Ratio

Figure S5D - Funnel plots in participants with a CD4 cell count <=200 cells/µL

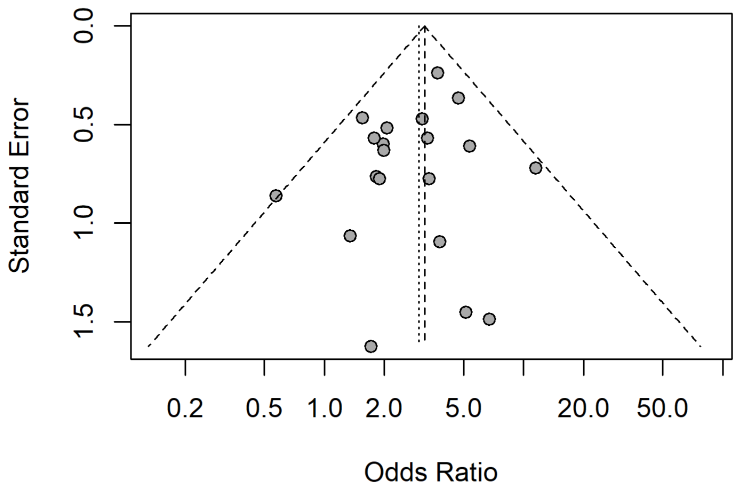

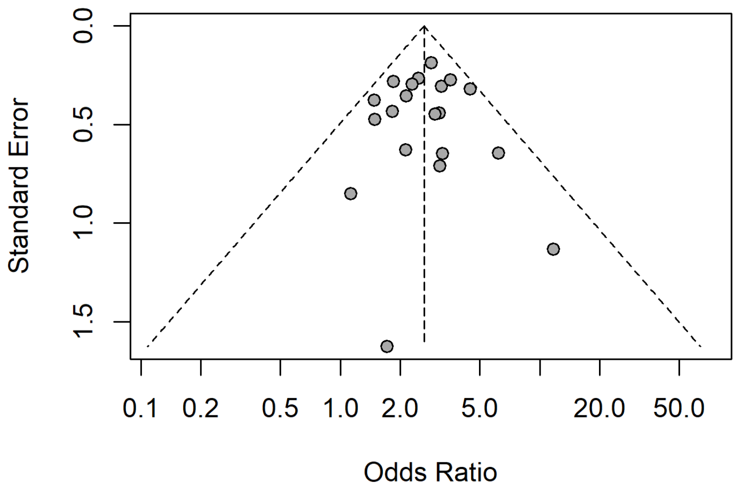

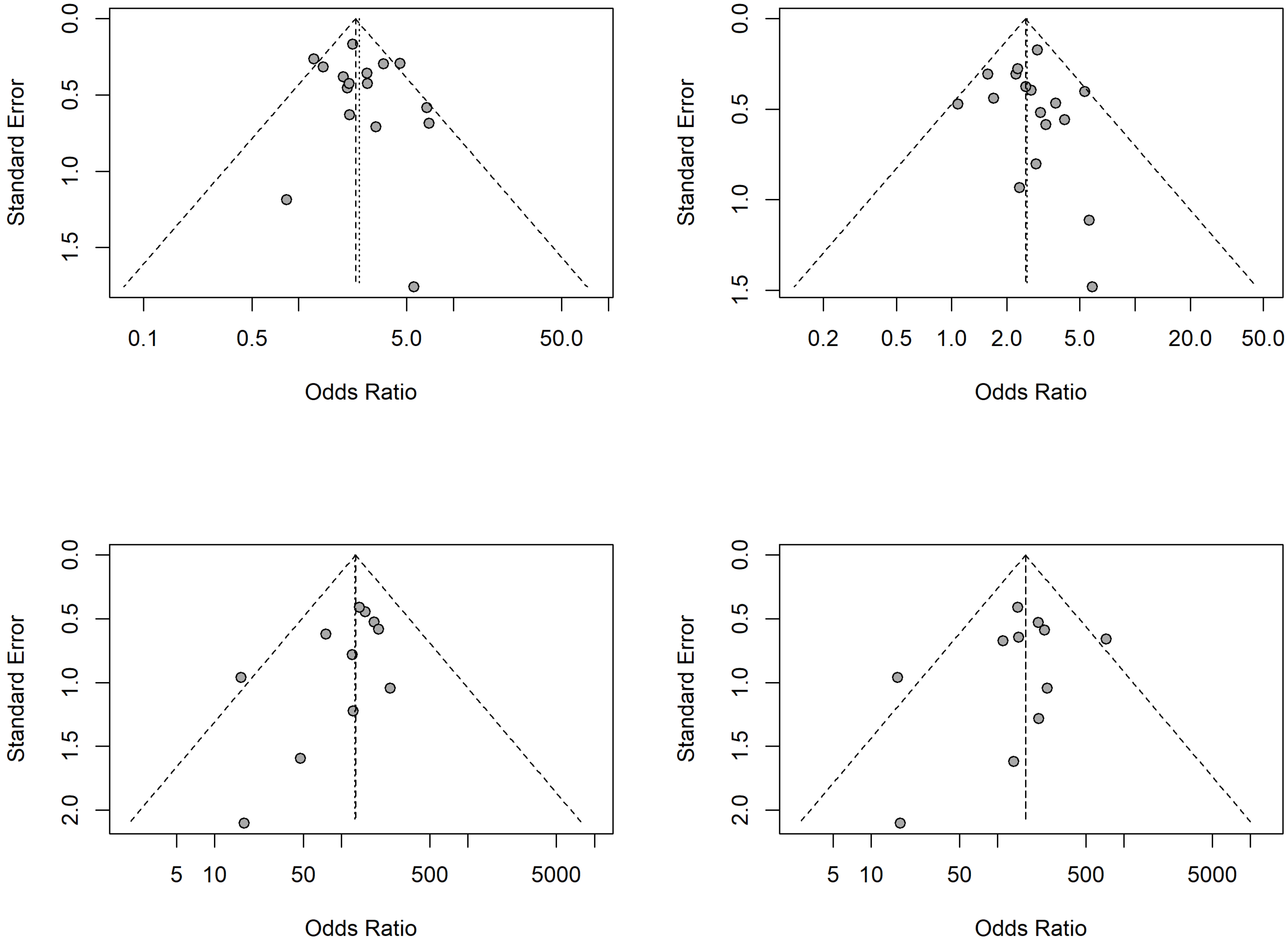
Standard Error
0.0
0.5
1.0
1.5
0.1
0.5
5.0
50.0
Odds Ratio
Standard Error
0.0
0.5
1.0
1.5
0.2
0.5
1.0
2.0
5.0
20.0
50.0
Odds Ratio
Standard Error
0.0
0.5
1.0
1.5
2.0
5
10
50
500
5000
Odds Ratio
Standard Error
0.0
0.5
1.0
1.5
2.0
5
10
50
500
5000
Odds Ratio

Figure S5E - Funnel plots in participants with a CD4 cell count >200 cells/µL

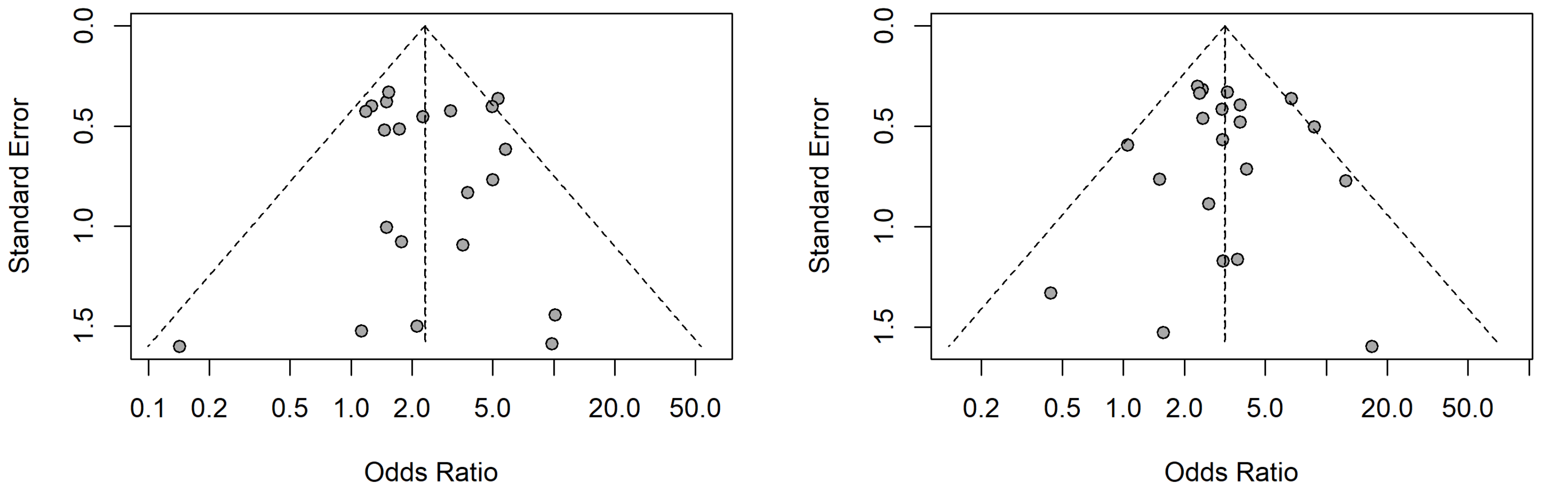

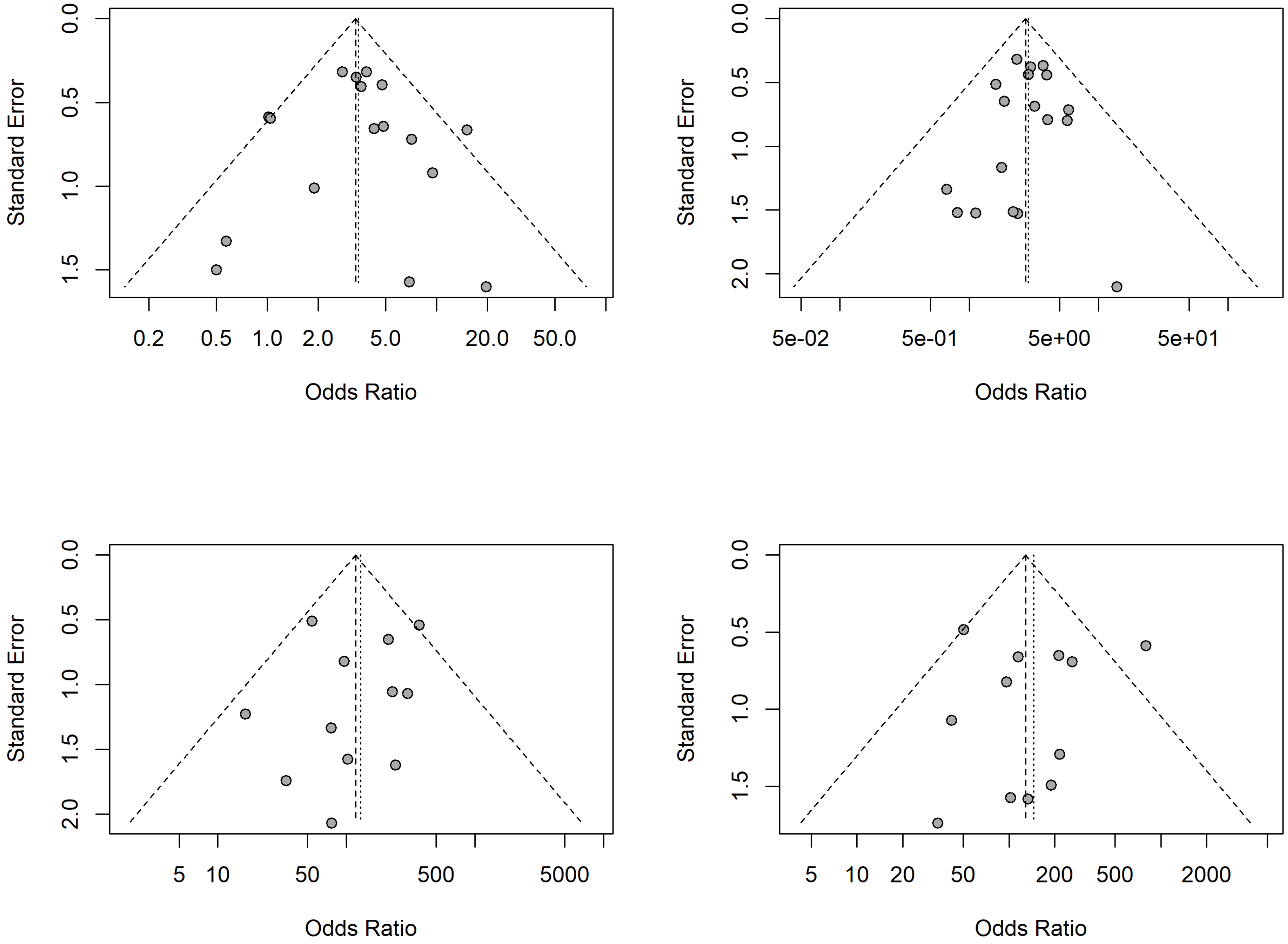
Standard Error
0.0
0.5
1.0
1.5
0.2
0.5
1.0
2.0
5.0
20.0
50.0
Odds Ratio
Standard Error
0.0
0.5
1.0
1.5
2.0
5e-02
5e-01
5e+00
5e+01
Odds Ratio
Standard Error
0.0
0.5
1.0
1.5
2.0
5
10
50
500
5000
Odds Ratio
Standard Error
0.0
0.5
1.0
1.5
5
10
20
50
200
500
2000
Odds Ratio

References

1. Al-Darraji HA, Abd Razak H, Ng KP, Altice FL, Kamarulzaman A. The diagnostic performance of a single GeneXpert MTB/RIF assay in an intensified tuberculosis case finding survey among HIV-infected prisoners in Malaysia. *PLoS One* 2013; **8**(9): e73717.
2. Affolabi D, Wachinou AP, Bekou W, et al. Screening tuberculosis in HIV infected patients: which algorithms work best? A multicountry survey in Benin, Guinea and Senegal (RAFAscreen project). 49th World Conference On Lung Health Of The International Union Against Tuberculosis And Lung Disease. The Hague, The Netherlands; 24–27 October, 2018.
3. Ahmad Khan F, Verkuijl S, Parrish A, et al. Performance of symptom-based tuberculosis screening among people living with HIV: not as great as hoped. *AIDS (London, England)* 2014; **28**(10): 1463-72.
4. Balcha TT, Skogmar S, Sturegard E, et al. A Clinical Scoring Algorithm for Determination of the Risk of Tuberculosis in HIV-Infected Adults: A Cohort Study Performed at Ethiopian Health Centers. *Open Forum Infect Dis* 2014; **1**(3): ofu095.
5. Bjerrum S, Kenu E, Lartey M, et al. Diagnostic accuracy of the rapid urine lipoarabinomannan test for pulmonary tuberculosis among HIV-infected adults in Ghana-findings from the DETECT HIV-TB study. *BMC Infect Dis* 2015; **15**: 407.
6. Gersh JK, Barnabas RV, Matemo D, et al. Pulmonary tuberculosis screening in anti-retroviral treated adults living with HIV in Kenya. *BMC Infect Dis* 2021; **21**(1): 218.
7. Hanifa Y, Fielding KL, Charalambous S, et al. Tuberculosis among adults starting antiretroviral therapy in South Africa: the need for routine case finding. *The international journal of tuberculosis and lung disease : the official journal of the International Union against Tuberculosis and Lung Disease* 2012; **16**(9): 1252-9.
8. Heidebrecht CL, Podewils LJ, Pym AS, Cohen T, Mthiyane T, Wilson D. Assessing the utility of Xpert((R)) MTB/RIF as a screening tool for patients admitted to medical wards in South Africa. *Scientific reports* 2016; **6**: 19391.
9. Hoffmann CJ, Variava E, Rakgokong M, et al. High prevalence of pulmonary tuberculosis but low sensitivity of symptom screening among HIV-infected pregnant women in South Africa. *PLoS One* 2013; **8**(4): e62211.
10. Kempker RR, Chkhartishvili N, Kinkladze I, et al. High Yield of Active Tuberculosis Case Finding Among HIV-Infected Patients Using Xpert MTB/RIF Testing. *Open Forum Infect Dis* 2019; **6**(6): ofz233.
11. Kerkhoff AD, Wood R, Lowe DM, Vogt M, Lawn SD. Blood neutrophil counts in HIV-infected patients with pulmonary tuberculosis: association with sputum mycobacterial load. *PLoS One* 2013; **8**(7): e67956.
12. Kufa T, Mngomezulu V, Charalambous S, et al. Undiagnosed tuberculosis among HIV clinic attendees: association with antiretroviral therapy and implications for intensified case finding, isoniazid preventive therapy, and infection control. *J Acquir Immune Defic Syndr* 2012; **60**(2): e22-8.
13. LaCourse SM, Cranmer LM, Matemo D, et al. Tuberculosis Case Finding in HIV-Infected Pregnant Women in Kenya Reveals Poor Performance of Symptom Screening and Rapid Diagnostic Tests. *J Acquir Immune Defic Syndr* 2016; **71**(2): 219-27.
14. Mbu ET, Sauter F, Zoufaly A, et al. Tuberculosis in people newly diagnosed with HIV at a large HIV care and treatment center in Northwest Cameroon: Burden, comparative screening and diagnostic yields, and patient outcomes. *PLoS One* 2018; **13**(6): e0199634.
15. Modi S, Cavanaugh JS, Shiraishi RW, et al. Performance of Clinical Screening Algorithms for Tuberculosis Intensified Case Finding among People Living with HIV in Western Kenya. *PLoS One* 2016; **11**(12): e0167685.
16. Nguyen DT, Bang ND, Hung NQ, Beasley RP, Hwang LY, Graviss EA. Yield of chest radiograph in tuberculosis screening for HIV-infected persons at a district-level HIV clinic. *The international journal of tuberculosis and lung disease : the official journal of the International Union against Tuberculosis and Lung Disease* 2016; **20**(2): 211-7.
17. Rangaka MX, Wilkinson RJ, Glynn JR, et al. Effect of antiretroviral therapy on the diagnostic accuracy of symptom screening for intensified tuberculosis case finding in a South African HIV clinic. *Clinical infectious diseases : an official publication of the Infectious Diseases Society of America* 2012; **55**(12): 1698-706.
18. Reeve B, Ndlangalavu G, Palmer Z, et al. Accuracy of Xpert Ultra and Xpert MTB/RIF in people living with HIV initiating antiretroviral treatment who have minimal TB symptoms. 50th World Conference On Lung Health Of The International Union Against Tuberculosis And Lung Disease. Hyderabad, India; Oct 30–Nov 2, 2019. p. S115.
19. Shapiro AE, Hong T, Govere S, et al. C-reactive protein as a screening test for HIV-associated pulmonary tuberculosis prior to antiretroviral therapy in South Africa. *AIDS (London, England)* 2018; **32**(13): 1811-20.
20. Swindells S, Komarow L, Tripathy S, et al. Screening for pulmonary tuberculosis in HIV-infected individuals: AIDS Clinical Trials Group Protocol A5253. *The international journal of tuberculosis and lung disease : the official journal of the International Union against Tuberculosis and Lung Disease* 2013; **17**(4): 532-9.
21. Thit SS, Aung NM, Htet ZW, et al. The clinical utility of the urine-based lateral flow lipoarabinomannan assay in HIV-infected adults in Myanmar: an observational study. *BMC Med* 2017; **15**(1): 145.
22. Yoon C, Semitala FC, Atuhumuza E, et al. Point-of-care C-reactive protein-based tuberculosis screening for people living with HIV: a diagnostic accuracy study. *Lancet Infect Dis* 2017; **17**(12): 1285-92.
